# Metabolic modeling of energy balances in *Mycoplasma hyopneumoniae* shows that pyruvate addition increases growth rate

**DOI:** 10.1002/bit.26347

**Published:** 2017-07-27

**Authors:** Tjerko Kamminga, Simen‐Jan Slagman, Jetta J. E. Bijlsma, Vitor A. P. Martins dos Santos, Maria Suarez‐Diez, Peter J. Schaap

**Affiliations:** ^1^ Laboratory of Systems and Synthetic Biology, Department of Agrotechnology and Food Sciences Wageningen University and Research Stippeneng 4 6708 Wageningen The Netherlands; ^2^ Bioprocess Technology and Support MSD Animal Health Boxmeer The Netherlands; ^3^ Discovery and Technology MSD Animal Health Boxmeer The Netherlands

**Keywords:** *Mycoplasma hyopneumoniae*, metabolic networks, constraint‐based metabolic modeling, energy balances, process optimization

## Abstract

*Mycoplasma hyopneumoniae* is cultured on large‐scale to produce antigen for inactivated whole‐cell vaccines against respiratory disease in pigs. However, the fastidious nutrient requirements of this minimal bacterium and the low growth rate make it challenging to reach sufficient biomass yield for antigen production. In this study, we sequenced the genome of *M. hyopneumoniae* strain 11 and constructed a high quality constraint‐based genome‐scale metabolic model of 284 chemical reactions and 298 metabolites. We validated the model with time‐series data of duplicate fermentation cultures to aim for an integrated model describing the dynamic profiles measured in fermentations. The model predicted that 84% of cellular energy in a standard *M. hyopneumoniae* cultivation was used for non‐growth associated maintenance and only 16% of cellular energy was used for growth and growth associated maintenance. Following a cycle of model‐driven experimentation in dedicated fermentation experiments, we were able to increase the fraction of cellular energy used for growth through pyruvate addition to the medium. This increase in turn led to an increase in growth rate and a 2.3 times increase in the total biomass concentration reached after 3–4 days of fermentation, enhancing the productivity of the overall process. The model presented provides a solid basis to understand and further improve *M. hyopneumoniae* fermentation processes. Biotechnol. Bioeng. 2017;114: 2339–2347. © 2017 The Authors. Biotechnology and Bioengineering published by Wiley Periodicals, Inc.

## Introduction

*M. hyopneumoniae* causes enzootic pneumoniae in pigs. Multiple vaccines are available that provide protection against *M. hyopneumoniae* infection; all contain the inactivated whole‐cell bacterium as active component. Manufacturing of these vaccines is done in large‐scale fermenter systems in which a sufficiently high biomass concentration should be reached to meet production requirements. However, reaching sufficiently high biomass concentrations in *M. hyopneumoniae* fermentations is challenging due to the fastidious growth requirements of this organism, which remain largely unknown.

Only for a small number of mycoplasma species a chemically defined medium enabling growth is available (Rodwell, 1969; Tourtelotte et al., 1964; Yus et al., 2009). As a result, growth media for production of mycoplasma vaccines are undefined and often contain components from animal origin for which the exact chemical composition is not known such as serum or animal‐derived peptones. Moreover, the concentration of critical components such as glucose, lipids and vitamins, varies largely thus challenging the development of a robust production process. In addition, the intrinsic growth rates of mycoplasma species are often low and possibly related to limitations in protein biosynthesis capacity (Yus et al., 2009), which poses an additional hurdle for the production process.

A constraint‐based metabolic model (CBM) provides a list of biochemical reactions, describes the network topology of metabolic pathways and provides a modeling framework to predict and understand biological processes (Thiele and Palsson, 2010). *Haemophilus influenzae* (Schilling and Palsson, 2000) was the first bacterium for which a CBM was made and since then >400 genome‐scale metabolic reconstructions have been made available for the scientific community via the Biomodels database (Juty et al., 2015). Considerable efforts have been made to model various mycoplasma species: *M. genitalium* (Suthers et al., 2009), *M. gallisepticum* (Bautista et al., 2013), *M. pneumoniae* (Wodke et al., 2013), and *M. hyopneumoniae* and other swine pathogens (Ferrarini et al., 2016). These models show in general that the inferred metabolic networks are small compared to other bacteria, linear and have a low redundancy (Yus et al., 2009), which is expected in bacteria with a minimal genome.

CBMs are routinely applied to optimize flux through a chosen reaction and to analyze flux distributions that support this minimal or maximal flux given the measured constraints related to substrate uptake or by‐product formation. Most often, the selected reaction is biomass formation as flux through the biomass reaction represents the organism's growth rate. Accurate growth rate predictions require detailed knowledge of the chemical composition of biomass. The biomass composition has been determined for *M. pneumoniae* (Wodke et al., 2013), which can be grown on defined medium, but it is a challenge to determine the biomass composition for species that cannot be cultured on defined media.

In this study, we developed and deployed a CBM to explore, specifically, energy metabolism. Our goal was to understand and improve a fermentation process used for commercial production of a *M. hyopneumoniae* vaccine. We validated the model with dedicated fermentor experiments in which we measured metabolite profiles and determined gene expression using RNA sequencing. We used the model to improve aerobic fermentation cultures on complex FRIIS medium thereby showing the potential of our modeling framework for further optimization of *M. hyopneumoniae* vaccine production processes.

## Materials and Methods

### Strain Cultivation and Genome and Transcriptome Sequencing

*M. hyopneumoniae* strain 11 cultures were grown using FRIIS medium (Friis, 1975) to which 1.5 g/l glucose was added or in medium to which 1.5 g/l glucose and 2.2 g/l sodium pyruvate was added. Sartorius Stedim Biostat fermentor systems were used with a maximum capacity of 2.0 L. The pH set‐point was controlled at 7.4 using only caustic soda (NaOH, 4N), temperature was controlled at 37°C and the dissolved oxygen concentration was controlled at 5% oxygen saturation using a stirrer speed cascade. Fermentation runs took on average 3–4 days during which 11–14 samples were withdrawn for metabolite and biomass analysis. A flow cytometer (FACSMicroCount, BD) was used to determine the total cell count and based on the titer the biomass concentration was calculated assuming an average cell mass of 0.074 pg/cell (Rosengarten et al., 1988). For genome sequencing, DNA was extracted from a bacterial pellet using the Gentra Puregene bacterial kit (Qiagen, GmbH, Hilden, Germany). A standardized method (supplementary materials) was used to sequence and assemble the genome using Illumina HiSeq sequencing (paired‐end, 50 cycles, 500 mb, 50 bp read length) and PacBio sequencing (1 SMRT cell, 60 mb). RNA was extracted from bacterial pellets using the Qiagen RNeasy mini kit. RNA was sequenced using Illumina HiSeq (single‐end reads, half a lane per sample, 50 cycles) using a standardized method (supplementary materials). Gene expression was analyzed using the R Bioconductor package SCAN_UPC (Piccolo et al., 2012). Changes in expression levels per gene were estimated by comparison of RPKM values (Reads Per Kilobase per Million mapped reads). Raw data of genome sequencing and RNA sequencing was deposited in the NCBI Short Read Archive (SRP101540 and SRP053697).

### Metabolite Analysis Using HPLC

Glycerol, glucose, fructose, mannose, myo‐inositol, and ribose were quantified in culture supernatant using an ion chromatograph ICS‐3000 system with a Dionex CarboPac MA1 (250 × 4.0 mm) column. Injection volume was 5 μL; eluents used were 100 mM NaOH and 5 mM NaOH. Lactate, acetate, formate and pyruvate were determined using the same HPLC system but fitted with a Dionex IonPac AS‐11‐HC column (250 × 4.0 mm) with a 5 μL injection volume and three eluents: 100 mM NaOH, 5 mM NaOH, and purified water. Each fermentation sample was measured once per method.

### Genome Annotation

The genome was annotated using the SAPP annotation pipeline, (Koehorst et al., 2016a). For gene prediction Prodigal version 2.6.2 (Hyatt et al., 2010) with codon table 4 was used. Protein annotation was done using InterProScan version 5.17–56.0 (Jones et al., 2014) as previously described (Kamminga et al., 2017).

### Construction Genome‐Scale Metabolic Model

**Figure 1:**
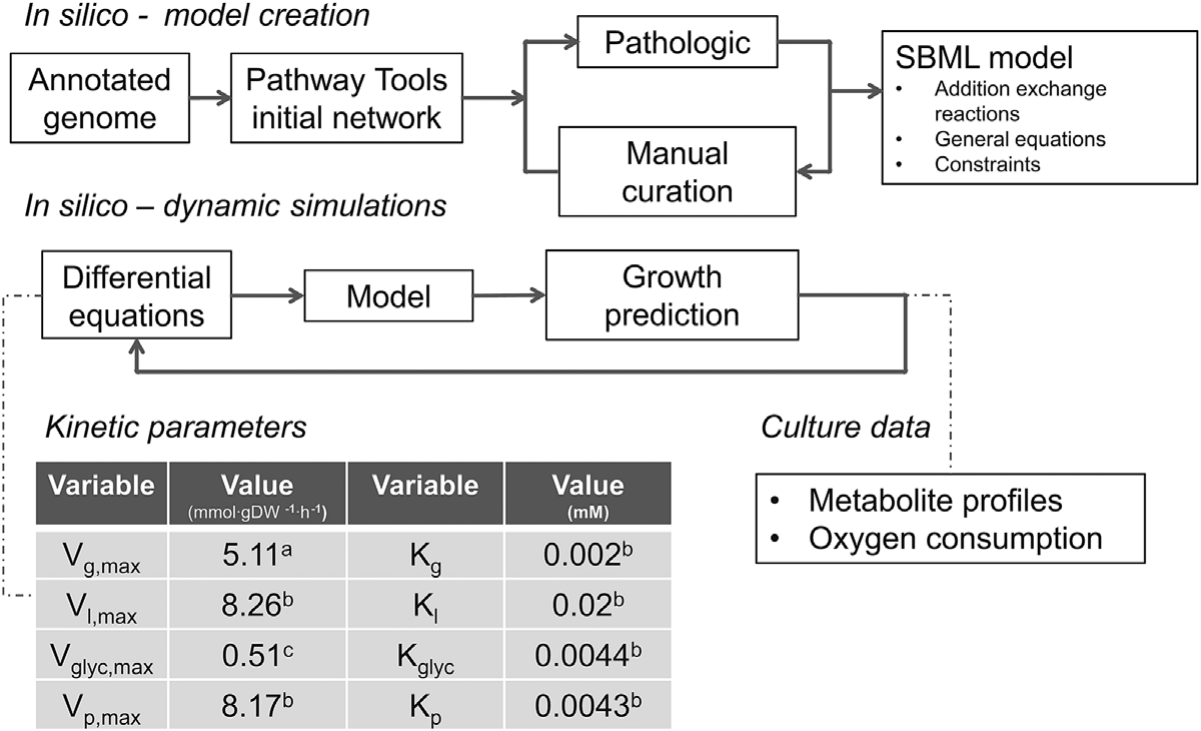
Creation and application of a dynamic flux balance model. Steps needed to create the dynamic model from the annotated genome of *M. hyopneumoniae*. Kinetic parameters were obtained from *M. pneumoniae* (a) (Wodke et al., 2013), *M. mycoides* (b) (Miles et al., 1985) or fitted from *M. hypneumoniae* HPLC data (c).

The annotated genome sequence was imported into Pathway tools (Karp et al., 2016). The PathoLogic tool v. 17.0 was used to create a draft metabolic map (Fig. 1 and supplementary methods), specifically the modules to assign possible enzymatic reactions, protein complexes and to predict presence or absence of metabolic pathways (Karp et al., 2011). Manual curation (supplementary materials) was performed using a decision scheme based on the presence or absence of functional protein domains and comparison to annotated genes in the *M. hyopneumoniae* reference genomes (strains: 232, J, 168, 168L, 7422, and 7448) and to the gene content of the *M. pneumoniae* metabolic model (Wodke et al., 2013). In line with previous studies (Pollack, 2002; Yus et al., 2009), we assumed broad substrate specificity for a number of enzymes in nucleotide metabolism. For instance, pyruvate kinase and the 5′‐nucleotidase were assumed to function with 8 nucleotide diphosphates as substrate. The entire Pathway Tools database was exported to flat files and with a BASH script converted to SBML. Cobrapy v0.2.1 (Ebrahim et al., 2013) was used to create a SBML version compatible with MATLAB v.R2016b and the Cobra Toolbox v.2.0.6 (Schellenberger et al., 2011) with LibSBML v.5.13.0 package enabled (Bornstein et al., 2008). Molecular formulas were added for macro‐molecules and cellular components when needed (supplementary materials). Additionally, transport reactions were added for all amino acids, nucleotides, vitamins, fatty acids, phosphatidylcholine, and general metabolic inputs and by‐products for which no annotated transporter existed.

### Constraint‐Based Modeling

Reactions in the model were represented as a matrix describing reaction substrates, products and their stoichiometries (Orth et al., 2010). We assumed biomass consisted of: 62% protein, 20% lipids, 5% DNA, 6.5% RNA, 1.5% free amino acids, and 0.1% acyl carrier protein (cofactors and vitamins were not included) (Razin et al., 1963; Wodke et al., 2013). Bounds were set for the reaction rates to represent reaction directionality. For several reactions (glucose, glycerol, and glycerol‐3‐phosphate uptake, Table S1), the bounds were set to match physiologically known constraints. Protein and RNA degradation were simulated by imposing positive lower bounds to the protein and RNA degradation reactions; values were taken from the *M. pneumoniae* model (Wodke et al., 2013). Flux balance analysis (FBA) was used to maximize the flux towards the chosen objective reaction, assuming a steady‐state system which mimics exponential growth and to identify combinations of fluxes supporting this maximal flux using the Cobra Toolbox v.2.0.6 and Gurobi solver v.6.5.2 (Gurobi Optimization, Houston, TX). Gene essentiality was assessed by setting the reaction rate(s) coupled to a gene of interest to zero and analyzing if the model still supported growth; that is if the model supported flux through the biomass synthesis reaction. Alternative flux distributions were identified through flux variability analysis (Mahadevan and Schilling, 2003). Briefly, the maximum value for the objective function was calculated; a new constraint was set for the objective function, so that the flux remains higher than 99.99% (or 99.9% in the second run) of the original value; subsequently each reaction in the model was set as objective function of two FBA rounds maximizing and minimizing the corresponding reaction. The model was deposited in the BioModels database (Chelliah et al., 2015) and assigned the identifier MODEL1704250001.

### Dynamic Model

Differential equations (supplementary materials) were used to model glucose, lactate, glycerol, oxygen, and pyruvate transport using first‐order Michaelis–Menten kinetics (Hjersted and Henson, 2006). Kinetic parameters were obtained from *M. mycoides* (Miles et al., 1985), *M. pneumoniae* (Wodke et al., 2013) or from batch data of *M. hyopneumoniae* fermentations (Fig. 1). Time profiles for bacterial cultivations were simulated by combining the set of dynamic equations and the genome‐scale metabolic model. The initial substrate, biomass and by‐product concentrations were used to calculate the growth rate, substrate and by‐product consumption/production rates at *t* = 0 h. With the set of differential equations the changes in substrate and by‐product concentrations were calculated using the ode45 solver in MATLAB v.R2016b. In each iterative cycle, the genome‐scale model was used to re‐calculate the growth rate and substrate and by‐product consumption/production rates (Varma and Palsson, 1994). Oxygen concentration in the liquid phase was fitted using a second order polynomial equation derived from the experimental data (Fig. S1).

## Results

### *M. hyopneumoniae* Strain 11 Genome Sequencing and Comparison to Published Genomes

Genome assembly including scaffolding and gap‐filling resulted in a genome sequence of length 898,877 base‐pairs (bp), consisting of 5 scaffolds, of lengths 341707, 244821, 176273, 32027, and 4049 bp, with an average GC content of 28.6% (NCBI accession number MWWN00000000). We identified 681 protein coding sequences, 466 proteins with at least one protein domain, 855 unique protein domains, and 77 EC numbers using InterProScan (Table I). The genome of strain 11 was compared to the currently published *M. hyopneumoniae* genomes (strains: 232, J, 168L, 168, 7422, and 7448) based on the protein domain repertoire (Kamminga et al., 2017; Koehorst et al., 2016b). We identified a pan‐domainome size of 866 protein domains, of which 846 (98%) were present in all strains (core domainome). We conclude from this comparative analysis that the metabolic capabilities of *M. hyopneumoniae* strains were predicted to be highly similar, as was also found by Ferrarini et al. (Ferrarini et al., 2016).

**Table I:** *M. hyopneumoniae* genome characteristics

Strain	Genome size (bp)	Total amount of proteins^a^	Proteins with domains^b^	Total unique domains^c^	Total unique EC#s^d^
11	898877	681	466	855	77
232	892758	681	468	856	78
J	897405	692	479	859	78
168	925576	698	470	857	78
168L	921093	700	471	857	78
7422	898495	703	477	857	78
7448	920079	690	476	862	78

^a^ Amount of proteins based on prodigal gene calling.

^b^ Number of proteins which contain at least one protein domain.

^c^ Number of unique domains annotated by InterProScan.

^d^ Number of unique EC numbers annotated by InterProScan.

### Genome‐Scale Metabolic Model

**Figure 2:**
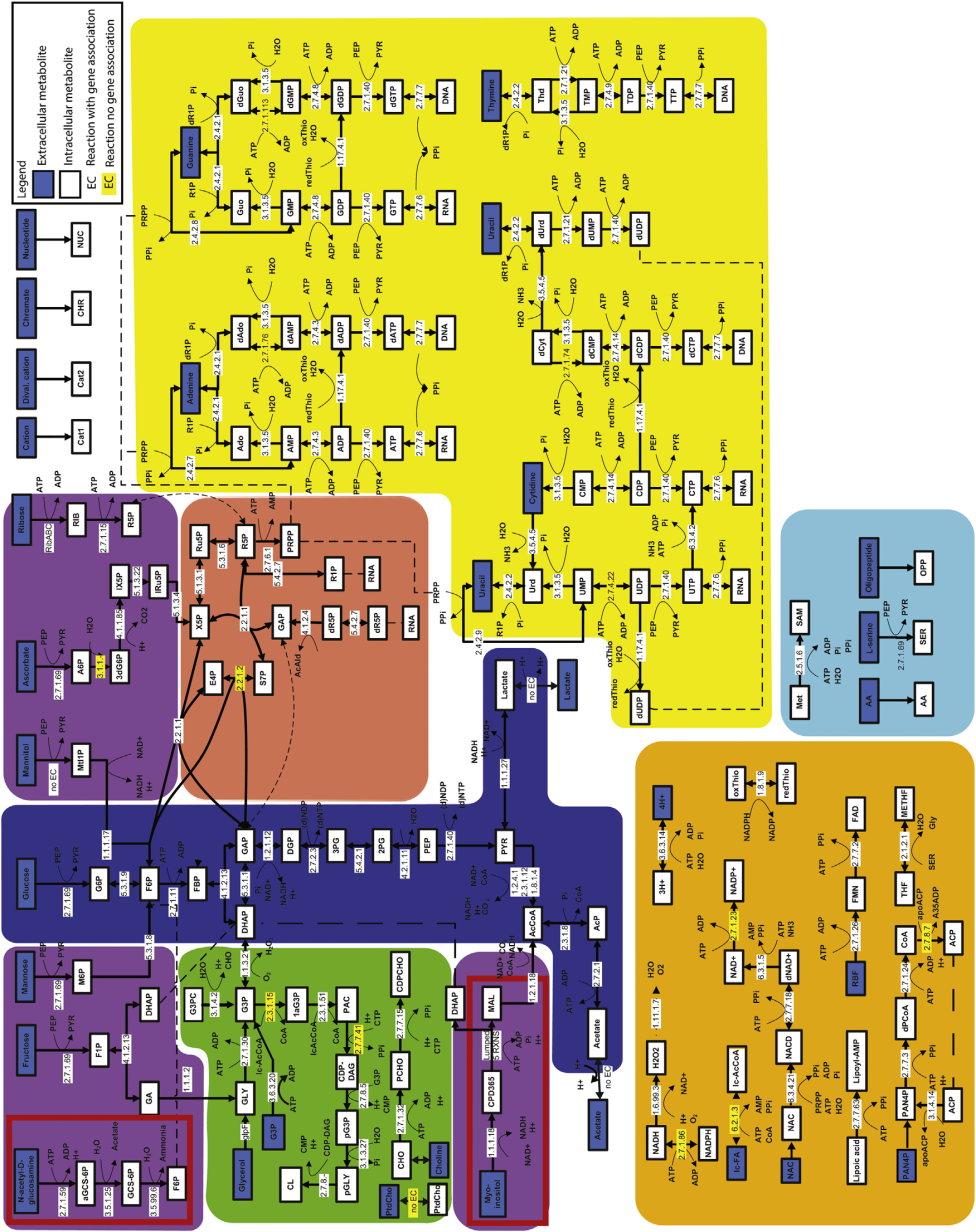
Metabolic map of *M. hyopneumoniae*. Pathways are indicated by background color: glycolysis (blue), alternative sugar metabolism (purple), lipid metabolism (green), vitamin and co‐factor metabolism (orange), nucleotide metabolism (yellow), Pentose Phosphate Pathway (PPP) (red), and amino acid metabolism (blue). Pathways highlighted in red are absent from *M. pneumoniae*. Abbreviations are explained in Table S11.

Based on the genome sequence of *M. hyopneumoniae* strain 11, we created a CBM which consisted of 284 reactions and 298 metabolites (Table II, Fig. 2). The model was termed TK284‐MHyo11. On average, the number of reactions catalyzed per enzyme was 1.39 ± 1.05; this value is close to other bacteria with a low number of protein coding genes (Yus et al., 2009). Analysis of RNA seq. data using SCAN_UPC (Piccolo et al., 2012, 2013) showed that the genes encoding enzymes underlying the reactions in the model were expressed under aerobic conditions on FRIIS medium (Fig. S2).

**Table II:** Model characteristics

	Model		
Model characteristic	TK284‐MHyo11	*M. hyopneumoniae* 232	*M. pneumoniae* M129
Total reactions	284^a^	426	306
Number of genes in the model	133	170	145
Number of gene‐protein reactions (GPR's)^b^	185	233	197
Orphan reactions^c^	82^a^	97	92
Exchange reactions^d^	52	97	57
Transport reactions	53	111	75
Metabolites	298	397	266

^a^ Excluded were 9 conversion reactions from mol to gram.

^b^ Number of gene protein reactions = number of reactions with an associated gene.

^c^ Orphan reactions = reactions without an associated gene, not including exchange reactions

^d^ Exchange reactions = reactions needed to exchange consumed or secreted compounds.

### Comparison to Existing Mycoplasma Models

The number of reactions in our model was lower than the number found in the model of *M. pneumoniae* or the reference model of *M. hyopneumoniae* (encompassing 306 and 426 reactions, respectively, Table II). The functional annotation of our model on the other hand was compared to the reference model of *M. hyopneumoniae* strain 232 and was found to be better aligned with the genome information as it contained less (orphan) reactions unlinked to genome encoded functions. This was a direct result of the approach used during manual curation, where we assigned functionality based on the presence of protein domains associated to a metabolic reaction and, in general, only added reactions for which associated protein domains where found in the genome, which resulted in a reduction in the number of orphan reactions. For example, we did not find protein domains related to fumarate production, succinate production, folate metabolism, phosphatidylcholine synthesis, and synthesis of glycolipids and therefore did not incorporate these reactions in our model although they were present in the reference model as orphan reactions. Interestingly, in the reference model of *M. hyopneumoniae* strain 232 there was no annotated gene for alcohol dehydrogenase (EC 1.1.1.1), while other *M. hyopneumoniae* strains did have this functionality annotated. In strain 11 we did not find a gene for alcohol dehydrogenase either. Comparison of our *M. hyopneumoniae* metabolic model to the *M. pneumoniae* metabolic model showed notable differences such as the presence (in our model) of a myo‐inositol degradation pathway, the presence of a N‐acetyl‐D‐glucosamine degradation pathway and a likely absence of folate metabolism as only a single gene related to folate metabolism could be found in the strain 11 genome: EC 2.1.2.1: glycine hydroxymethyltransferase. Also absent in our model when compared to *M. pneumoniae* were alcohol dehydrogenase, arginine fermentation, glycolipid metabolism, and thymidylate synthase.

### Parameterization and Validation

We measured the initial substrate concentrations in several medium lots to determine starting concentrations of metabolites (Table S2). The average growth rate reached in FRIIS medium was 0.0177 h^−1^ (duplicate batches 0.0219 and 0.0135 h^−1^, respectively). Part of the growth rate variation between batches is most likely caused by variations in the medium composition due to the use of components from animal origin. For initial model simulations the only carbon sources for which consumption was allowed were glucose, glycerol and glycerol‐3‐phosphate. We had no analytical method to measure glycerol‐3‐phosphate in the medium and therefore copied the consumption rate for this compound from the *M. pneumoniae* model. Additionally, we allowed in silico consumption of other components such as amino acids, nucleotides and lipids as listed in Table S3. We used the CBM to estimate (and subsequently fix in the model) an energy expenditure of 18.4 mmol · gDW^−1^ · h^−1^ as non‐growth associated maintenance rate (NGAM). The NGAM was estimated by adding a non‐specific energy consumption reaction to the model. The lower limit of this reaction was then gradually increased until agreement was found between the measured and the model predicted growth rate.

**Figure 3:**
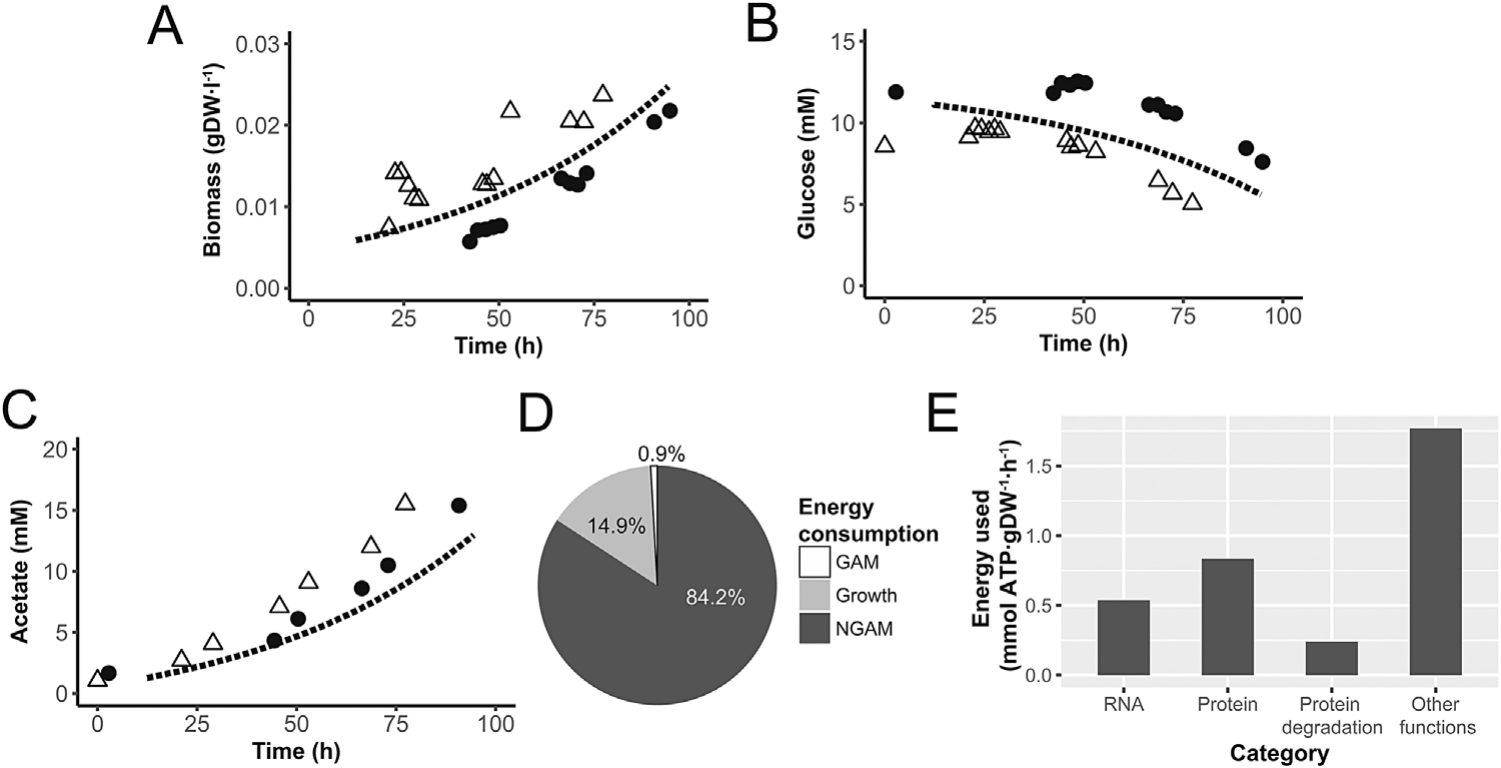
Metabolite profiles and energy balances in standard *M. hyopneumoniae* fermentations. Comparison of model predicted aerobic batch profiles (dashed line) to measured profiles in fermentation batches (*N* = 2, dots and pyramids) for: (**A**) biomass, (**B**) glucose, (**C**) acetate concentration, (**D**) distribution of energy consumption, and (**E**) major growth related energy consumption.

The parameterized CBM was used to build the integrated dynamic model, which was validated by simulation of time profiles in aerobic fermentor cultures. We assumed glycerol to be a non‐limiting component in these cultures since it could be obtained from complex medium components. Based on oxygen consumption profiles we calculated an average lag‐phase of 12.5 h for the duplicate fermentation cultures and started model simulations with a biomass concentration of 5.89 · 10^−3^ gDW · l^−1^. Measured biomass and glucose concentrations were in agreement with predicted values (Fig. 3A and B). The predicted acetate concentration was slightly lower than measured (Fig. 3C), indicating possible consumption of additional carbon sources from the complex medium.

### Analysis of In Silico Flux Distributions and Energy Balances

Up to 124 enzymatic and transport reactions were able to carry flux while growing on glucose and glycerol under aerobic conditions, 44% of the total amount of reactions in the model (excluding conversion reactions, Table II). NGAM required 84.2% of total cellular energy, 0.9% was used for growth‐associated maintenance and 14.9% for reactions needed for growth (Fig. 3D, Table S4). In *M. pneumoniae* cultures 71–88% of cellular energy was used for NGAM and 12–29% for growth, depending on the culture stage (Wodke et al., 2013). Among the growth related functions, the ones consuming the most energy were protein production, RNA production and protein degradation (Fig. 3E). Energetic costs of DNA production were negligible compared to these other categories. When compared to the growth related energy sinks in *M. pneumoniae*, our model did not contain protein folding as energy sink and had reduced lipid synthesis capability.

### Flux Variability Analysis and Gene Essentiality Analysis

We performed flux variability analysis to explore alternative flux distributions compatible with at least 99.99% of the maximal growth rate. The majority of the reactions showed negligible variability as was also found for *M. pneumoniae* (Wodke et al., 2013). We identified the 20 reactions that showed the largest flux change (Table S5). All these reactions were related to conversion of purine nucleotides; there was some flexibility in the network which allowed for alternative pathways to produce dADP and dGDP (Fig. 2). Reaction directionality was changed for the reactions catalyzed by phosphoglycerate kinase which confirmed that the flexible substrate use assumed for this enzyme improved network flexibility. We also performed flux variability analysis allowing 0.1% (Table S6) variation in the growth rate and we identified additional reactions for which directionality changed: CMP kinase and the asparagine‐tRNA ligase. For the latter function there were two separate reactions in the model, one irreversible and one reversible reaction which provided flexibility in the network and allowed the direction change for the reversible reaction. Finally, gene essentiality analysis showed that 41% of genes in the model were classified as essential for growth (Table S7). There is currently insufficient experimental information on gene essentiality in *M. hyopneumoniae* to validate this result (Maglennon et al., 2013).

### Model Predicts That Pyruvate Addition Increases the Growth Rate

**Figure 4:**
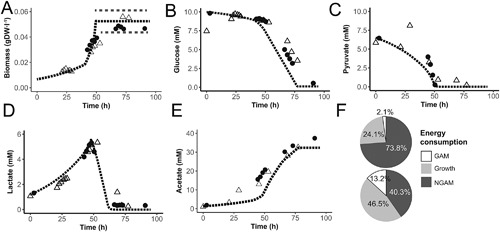
Pyruvate supplementation increases total biomass yield and results in lactate production under aerobic conditions. Comparison of model predicted aerobic batch profiles (dashed line) to measured profiles in fermentation batches (*N* = 2, dots and pyramids) after pyruvate supplementation to medium for: (**A**) biomass, dotted grey lines indicate variation in the final concentration of biomass reached, (**B**) glucose, (**C**) pyruvate, (**D**) lactate, (**E**) acetate concentration, and (**F**) energy distribution early stage (top) and at *t* = 45h.

As was also observed in *M. pneumoniae,* the metabolic pathway that carried the largest amount of flux was glycolysis and pyruvate metabolism. Pyruvate has been mentioned in literature to increase the growth rate of mycoplasma species (Miles et al., 1988). We confirmed that in our model pyruvate addition increases growth rate and ran dedicated fermentor studies to assess the effect on growth and metabolite profiles (Fig. 4A–E). Interestingly, we observed production of lactate under aerobic conditions (Fig. 4D) which is energetically unfavorable. Three metabolic scenarios were simulated with the dynamic integrated model: (i) growth on pyruvate with lactate production and a lowered biomass specific glucose uptake rate; (ii) growth on glucose and pyruvate; and (iii) growth on lactate, glucose and pyruvate. In the initial metabolic condition the flux through the PEP‐PTS transporter for glucose was assumed to be lower as a result of product inhibition. The previously fitted NGAM rate was too high to support growth in the initial metabolic condition when cells were growing on pyruvate and glucose. The growth rate measured in the initial stage of the culture was compatible with a decreased NGAM rate of 10.15 mmol ATP · gDW^−1^ · h^−1^. Based on measured metabolite profiles we increased the flux through the glucose transporter when the pyruvate concentration was less than 2.7 mM. This had a positive influence on the growth rate (Fig. 4A). Near the time of pyruvate exhaustion in the medium, lactate is consumed and growth‐rate further increases. In our simulation, the culture reached stationary phase when a biomass concentration of 0.05 gDW · l^−1^ was reached according to measured biomass data. Energy balances at early and late (*t* = 45 h) exponential growth showed that in the simulations with pyruvate addition, a higher fraction of energy was allocated to growth (Fig. 4F).

### Strain Design

The model was used to study the impact of gene knock‐in mutants on growth assuming growth on FRIIS medium with only glucose added. Our model includes a complete myo‐inositol consumption pathway which was absent in other mycoplasma species. We did not observe consumption of this component in aerobic fermentor studies and in our model the myo‐inositol transporter is an orphan reaction as there was no annotated transporter. A *M. hyopneumoniae* mutant with a knock‐in of a myo‐inositol permease should be able to consume myo‐inositol from the growth medium, for such a mutant the model predicted a doubling of the growth rate (from 0.0177 to 0.0352 h^−1^) upon consumption of 0.25 mmol myo‐inositol · gDW^−1^ · h^−1^ (Table S8). If a knock‐in with an ABC transporter for myo‐inositol is done, the ATP demands of the transporter would enable a slightly lower growth rate (0.0308 h^−1^). An even more challenging knock‐in would be the expression of the complete pathway for arginine fermentation, which is absent in *M. hyopneumoniae*. In *M. pneumoniae* this pathway consists of five genes (MPN304‐307 and MPN560) coupled to three reactions. Simulation of a knock‐in with the five *M. pneumoniae* genes and an additional transporter for one of the by‐products L‐ornithine leads to an increased in silico mutant growth rate of 0.0235 h^−1^ (Table S8), as long as arginine consumption is allowed with the same flux as determined in *M. pneumoniae*.

## Discussion

We have developed and applied a CBM of *M. hyopneumoniae* strain 11 to study and improve biomass yield in fermenter systems. Our aim was to understand cellular energy balances during growth and find methods to increase biomass yield. We found that in the standard cultivation medium used for *M. hyopneumoniae*, 84% of total ATP production by the cell is used for non‐growth associated maintenance and only 14.9% is used for growth. This confirms the results found in *M. pneumoniae*. A possible explanation for the high percentage of energy used for NGAM could be the high surface‐to‐volume ratio of mycoplasma species which means that maintaining cellular homeostasis is energetically expensive (Wodke et al., 2013).

Because our focus for modeling was mainly on understanding cellular energy balances we used a simplified biomass composition in the model. We calculated ATP consumption of reactions related to vitamin and cofactor metabolism in the *M. pneumoniae* model and found that these reactions consumed only 0.003% of total cellular energy (Table S9). This shows that strictly for understanding the cellular energy distribution, the contribution of reactions in co‐factor and vitamin production can be neglected. Moreover, it remains unclear which of these components are consumed directly from the complex medium and which are produced by the bacterium. Our CBM also lacked almost all reactions related to folate metabolism although this component is generally assumed to be essential for growth because formylation of initiator methionyl‐tRNA is needed to start translation in bacteria (de Crécy‐Lagard et al., 2007). However, because formylation of methionyl‐tRNA does not occur in *Pseudomonas aeruginosa* (Newton et al., 1999) and is also not described as part of the minimal bacterial gene set (Gil et al., 2004), we find the assumption that it is absent in *M. hyopneumoniae* reasonable.

Our simulations show that a large part of the metabolic network is not used for growth, in the simulated conditions, and as a result many genes are predicted to be non‐essential. This is an interesting result as mycoplasma genomes are expected to be close to the minimal gene set required for life without a host and therefore a high percentage of genes were expected to be essential. Part of the result can be explained because we applied a simplified biomass equation, for example, addition of NADPH, FAD, and CoA to the biomass equation added 5 essential genes, a 3.8% increase in the percentage of essential genes (Table S7). Also our predictions for gene essentiality were based on growth in complex medium while conditions in the host will be different and gene essentiality might be higher because nutrients are scarce. Though not all genes were found to be essential, our transcriptomics dataset indicated that all genes in the model were expressed and although the transcriptome dataset collected is small, we identified a correlation between gene expression and flux for the glycerol uptake facilitor (glpF) and for the glucose PTS transporter (MHP629) (Table S10).

Given the challenges associated with introducing genetic modification in *M. hyopneumoniae*, the potential of the described knock‐in mutants has to be carefully evaluated. Our model predicted that both the introduction of a myo‐inositol transporter (single gene knock‐in) as well as an arginine fermentation pathway (5 gene knock‐in) potentially increases the growth rate of mutant strains.

To our knowledge, this is the first study where *M. hyopneumoniae* growth is studied in controlled fermenter systems. In these systems pH and oxygen concentration were controlled resulting in a stabilized growth profile and allowing accurate measurement of growth rate, substrate uptake rates and by‐product formation which is essential data for model validation and parameterization. Our stringent approach for model creation, applying annotation based on functional protein domains, resulted in a more condensed model of *M. hyopneumoniae* when compared to the reference model. Following an iterative cycle of model driven experimentation, we were able to double biomass production and reduce total process time by pyruvate addition to aerobic fermentations, which improved the economic potential of the production process. The model we present provides a solid basis to understand the metabolic capability of *M. hyopneumoniae* and further optimize the production process regarding biomass yield and process robustness.

**Nomenclature**
CBM: Constraint‐based model
SBML: Systems Biology Markup Language
COBRA: Constraint Based Reconstruction and Analysis
DW: Dry weight
BASH: Bourne‐again shell; Metabolite abbreviations are explained in Table S11

Authors acknowledge Mark Davids for help with model transformation from Pathwaytools to SBML and further adaptation of the model using the COBRA toolbox in Matlab. Also, we acknowledge the P‐ATS department at MSD‐AH for analytical support. This work was financially supported by MSD Animal Health, Bioprocess Technology and Support, Boxmeer, The Netherlands. VMS, PS, and MS‐D also received funding from the European Union's Horizon 2020 research and innovation program under grant agreement No. 634942.

## Authors’ Contribution

TK, SJS, PS, and VMS contributed to study design. TK, MS‐D, and PS interpreted the results. TK, PS, JB, and MS‐D drafted the manuscript. All authors revised the manuscript and approved the final version. All authors take responsibility for accuracy and integrity of the work.

## Supporting information

Additional supporting information may be found in the online version of this article at the publisher's web‐site.

**Figure S1**. Dissolved oxygen concentration in aerobic fermentations. Dashed lines show measured values and the solid lines shows values assumed during dynamic modeling. (A) Measured dissolved oxygen concentration in aerobic batches grown on glucose. Solid line was assumed for the model based on a second order polynomial fit (−0.0317*t ^ 2–3.1847*t + 81.837). (B) Measured dissolved oxygen concentration in aerobic batches grown on glucose and pyruvate. Solid line was assumed for the model based on a second order polynomial fit (−0.0638*t ^ 2−3.0848*t + 101.83).
**Figure S2**. SCAN_UPC analysis for genes in TK284‐MHyo11. Evaluation of gene expression was done based on read counts per gene present in the genome‐scale model using the package SCAN_UPC (Piccolo et al., 2012; Piccolo, Withers, Francis, Bild, & Johnson, 2013). UPC expression scores are high or 1 for all genes in the model indicating that these genes were expressed. Calculations were done with reads counts obtained from the fermentation with basic FRIIS medium with only glucose added.

**Table S1**. Initial model constraints for simulations in medium with glucose and early growth in medium with glucose and pyruvate. Highlighted in yellow are the model constraints that were changed in the simulations with pyruvate present.

**Table S2**. Metabolite starting concentrations FRIIS medium.

**Table S3**. Components consumed directly from the growth medium.

**Table S4**. Calculation of energy distributions from model predicted reaction fluxes.

**Table S5**. Flux variability analysis at 99.99% of maximal initial growth rate.

**Table S6**. Flux variability analysis at 99.9% of maximal initial growth rate.

**Table S7**. Gene essentiality analysis using the metabolic model. Highlighted genes were found to be essential with a biomass equation containing NADPH, FAD, and CoA

**Table S8**. Flux distributions for knock‐in strains.

**Table S9**. Predicted fluxes for the exponential growth phase.

**Table S10**. Expression level of model genes determined using RNA sequencing in the glucose batch and pyruvate batch. Highlighted genes were mentioned in the manuscript.

**Table S11**. Abbreviations used for metabolite names in the metabolic map.
