# Supplementary material for: Metabolic modeling of energy balances in Mycoplasma hyopneumoniae shows that pyruvate addition increases growth rate
Source: Biotechnol Bioeng. 2017 Jul 27;114(10):2339–47. doi: 10.1002/bit.26347 (PMC6084303; doi:10.1002/bit.26347)
Supplement: Supplementary file 1 — Figure S1. Dissolved oxygen concentration in aerobic fermentations. Dashed lines show measured values and the solid lines shows values assumed during dynamic modeling. (A) Measured dissolved oxygen concentration in aerobic batches grown on glucose. Solid line was assumed for the model based on a second order polynomial fit (−0.0317*t ^ 2–3.1847*t + 81.837). (B) Measured dissolved oxygen concentration in aerobic batches grown on glucose and pyruvate. Solid line was assumed for the model based on a second order polynomial fit (−0.0638*t ^ 2−3.0848*t + 101.83). Figure S2. SCAN_UPC analysis for genes in TK284‐MHyo11. Evaluation of gene expression was done based on read counts per gene present in the genome‐scale model using the package SCAN_UPC (Piccolo et al., 2012; Piccolo, Withers, Francis, Bild, & Johnson, 2013). UPC expression scores are high or 1 for all genes in the model indicating that these genes were expressed. Calculations were done with reads counts obtained from the fermentation with basic FRIIS medium with only glucose added. [file BIT-114-2339-s001.docx]

Supplementary Materials

Metabolic modeling of energy balances in *Mycoplasma hyopneumoniae* shows that pyruvate addition increases growth rate

Tjerko Kamminga^1,2^, Simen-Jan Slagman^2^, Jetta J.E. Bijlsma^3^, Vitor A.P. Martins dos Santos^1^, Maria Suarez-Diez^1^ and Peter J. Schaap^1^*

^1^ Laboratory of Systems and Synthetic Biology, department of Agrotechnology and Food Sciences, Wageningen University and Research, Wageningen, The Netherlands; ^2^ Bioprocess Technology and Support, MSD Animal Health, Boxmeer, The Netherlands; ^3^ Discovery & Technology, MSD Animal Health, Boxmeer, The Netherlands

*Corresponding author: [peter.schaap@wur.nl](mailto:peter.schaap@wur.nl)

Contents

[Materials and Methods 3](#_Toc476735573)

[*Differential equations used in the dynamic flux balance model:* 9](#_Toc476735574)

[Supplementary figures 10](#_Toc476735575)

[References 11](#_Toc476735576)

# Materials and Methods

*Genome sequencing and annotation*

The genome library was created using the TruSeq DNA sample prep kit (Illumina). Illumina HiSeq sequencing (paired-end, 50 cycles, 500 mb, 50 bp read length) and PacBio sequencing (1 SMRT cell, 60 mb) was performed. Illumina FASTQ sequence reads were generated using the Illumina CASAVA pipeline version 1.8.2. Initial quality control was done using the Illumina Chastity filtering. Baseclear applied an in-house developed script to remove (filtered) reads containing adapters and/or a PhiX control signal. Remaining reads were analyzed using the FASTQC quality control tool version 0.10.0. Continuous PacBio long-read data was filtered using the SMRT analysis software suite. Data was filtered by read-length (>50), subread-length (>50) and read-quality (>0.75). Quality of the filtered reads was analyzed using the CLC Genomics workbench version 6.0.1. A de novo hybrid assembly was done using the Illumina and PacBio data using CLC Genomics workbench (v. 5.5.1). Low quality bases were trimmed off using the “Trim sequences” option. Contigs were assembled from the quality filtered reads using “De novo assembly” in CLC Workbench. PacBio CLR reads were used for scaffolding using BLASR (Chaisson & Tesler, 2012). Gaps in the super-scaffolds were (partially) closed using GapFiller v1.1 (Boetzer et al., 2012).

*Transcriptome sequencing*

rRNA was removed using the Ribo-Zero Kit (Epicentre). rRNA-depleted RNA was fragmented with an average length of 100 to 200 base pairs (bp) and converted to double-stranded complementary DNA (cDNA). Library preparation was done using a protocol based on the “dUTP (deoxyuridine triphosphate) method,” to generate strand-specific mRNA-seq libraries including barcoding. The Illumina stranded TruSeq RNA-seq library preparation kit was used. Sequencing of the library was done using the Illumina HiSeq: single-end reads, one lane, 50 cycles, two samples per lane. Illumina sequence filtering and quality trimming was performed as described above. Reads were mapped using Bowtie (Langmead & Salzberg, 2012).

*Creation of the metabolic map*

The draft metabolic map created with Pathway Tools contained 340 reactions, 344 compounds, 16 transporters and only 3 transport reactions. Semi-automatic curation using Pathologic resulted in the assignment of EC numbers to 17 genes in the draft network based on protein domain content or based on annotated functionality in the genome of strain 232. Seven protein complexes were created, three enzymes (ATPase, pyruvate dehydrogenase, ribonucleotide reductase) and four transporters (a PTS transporter for mannitol, an ascorbate transporter, a D-ribose transporter and a glycerol-3-phosphate ABC transporter). An additional 13 transport reactions were assigned semi-automatically which resulted in an updated draft metabolic map containing 349 reactions, 343 compounds, 20 transporters and 16 transport reactions. Extensive manual curation of the metabolic map was done during which we aimed to minimize the amount of orphan reactions in the draft metabolic map. We did not remove dead-end reactions since these could still function if precursors are directly obtained from the medium. The final pathway map contained 222 reactions, 261 compounds, 21 transporters and 18 transport reactions. Only 30 orphan reactions were present. The final map in Pathway Tools was exported and used as basis for the genome-scale metabolic model of *M. hyo* strain 11.

*Model creation using the Cobra Toolbox*

Molecular formulas were added to the tRNA’s, components in lipid metabolism (phosphatidate, long-chain acyl-ACP’s, acyl-sn-glycerol-3-phosphate, long-chain fatty acids, apoACP, cardiolipin, CDP-diacylglycerol, L1-phosphatidylglycerol, L1-phosphatidylglycerolphosphate and long-chain acyl-CoA’s) and co-factors for which no structural formula was defined (oxidized and reduced thioredoxin, electron donors/acceptors in ascorbate metabolism, acyl carrier protein, lipoate intermediated for pyruvate dehydrogenase). All other reactions with metabolites for which no molecular formulas existed were removed from the metabolic model. Reactions were created for the synthesis and degradation of proteins, acyl-carrier protein and RNA (the latter two were copied from the *M. pneumoniae* model (Wodke et al., 2013)). Synthesis reactions were created for DNA, the lipid component of biomass, the unbound amino acids in biomass and for total biomass. Specifically, we assumed that the lipid component in biomass consists of phosphatidylcholine 32:1 (47%), cardiolipin 64:2 (50%) and phosphatidate 32:1 (3%) (Hwang et al., 1986). We did not incorporate vitamins or cofactors in the biomass equation. Other synthesis and degradation reactions were copied from the *M. pneumoniae* model (Wodke et al., 2013). Growth associated maintenance requirements were assumed to be similar to the reference *M. hyopneumoniae* model (Ferrarini et al., 2016). We assumed that oxygen uptake is not a rate limiting factor and applied a sufficiently high maximum oxygen uptake rate to prevent oxygen limitation (27 mmol/gDW/h).

We removed 46 reactions which contained metabolites without molecular formula and their associated genes (total 44 genes). 41 transport reactions were added and in total 52 exchange reactions were added for modeling purposes for all compounds which were directly obtained from medium and for byproducts of metabolism. We defined a basic biomass composition containing DNA, RNA, protein, acyl-carrier protein, lipids and single amino acids present in biomass. *In silico* growth, meaning flux through the biomass formation reaction, was not possible with the initial model based on functionalities in the metabolic map. We needed to add five orphan reactions to enable growth: deoxyguanosine kinase, deoxyadenosine kinase, UMP kinase, NAD kinase and NADH kinase. These reactions were also present in the reference mycoplasma models of *M. hyopneumoniae* and *M. pneumoniae*.

*Manual curation metabolic network M.hyo strain 11*

*Initial pathway and Pathologic improvement*

Genbank annotation from SAPP was loaded into Pathway Tools. Function for 36 genes was determined using the “assign probable enzymes” functionality in Pathologic. To 17 genes an EC number could be assigned. Other genes were not metabolic, non-specific, unknown or flagged for future consideration. After assigning the probable enzymes pathways were rescored. Finally, a PTS transporter for glucose was assigned (gene 93).

*Creation of protein complexes*

Protein complexes were created for the following enzymes. The number of units in the complex was assumed to be 1 for each complex (this is not a critical component in the genome-scale metabolic model).

1. ATPase is a complex of 8 subunits (genes: 208-213, 541 and 542);
2. Pyruvate dehydrogenase (PDH-E1) consists of 2 subunits (genes 429 and 430);
3. Ribonucleotide reductase complex was added consisting of genes 325-327;
4. PTS transporter for mannitol was complex was made (genes 36 and 38);
5. Ascorbate transporter activity was assigned to complex of genes 674-676 based on presence of domain IPR004703;
6. D-ribose transporter functionality was added for the complex of genes 315-317. EC 3.6.3.17 was already assigned but specificity for ribose was added;
7. Glycerol-3-phosphate ABC transporter activity was added to the gene cluster 669-671.

After assigning the protein complexes with associated reactions the “assign probable enzymes” option in Pathologic was re-run. Five new genes were found and ATPase activity (EC3.6.3.14) was assigned to genes 206 and 207. PTS transporter for fructose was added to gene 555. Two unknowns were also present in the list and were not assigned (genes 41 and 53). Pathways were re-scored.

*Pyruvate metabolism*

Reaction EC 2.3.1.12, dihydrolipoyllysine-residue acetyltransferase, was assigned to gene 568 based on the presence of protein domains.

*Myo-inositol metabolism*

The myo-inositol pathway (P562-PWY) was imported. All reactions in this pathway could be assigned to genes. Reaction EC 4.1.2.29 was assigned to gene 171 although definitive proof is lacking. Another reaction related to myo-inositol metabolism, EC 5.3.1.30 glucuronate isomerase, was added to gene 321.

*Ascorbate metabolism*

Ascorbate degradation pathway was completed by assigning reaction EC 5.1.3.22 to gene 504, RXN0-5214 to gene 677 (Ferrarini et al., 2016) and RXN0-705 to gene 505 For two reactions in the pathway no annotated genes are present.

*Lipid and glycerol metabolism*

CDP-diacylglycerol biosynthesis pathway is present. Phosphatidate cytidylyltransferase (EC 2.7.7.41) and glycerol-3-phosphate acyltransferase activity was assumed to be present although there were no relevant protein domains present. Cardiolipin synthase reaction was added to gene 197. Glycerol metabolism was added by assignment of the glycerol uptake facilitor (gene 659) and glycerol-3-phosphate oxidase (EC 1.1.3.21). Insufficient evidence was found to assign a glycerol ABC transporter. EC 2.7.7.15, choline-phosphate cytidylyltransferase, was assigned to gene 539. EC 3.1.4.2, glycerophosphocholine phosphodiesterase, assigned to 603. Triacylglycerol lyase (EC 3.1.1.3) was assumed to have only triacylglycerol as substrate, reactions with other substrates were removed.

*Pentose phosphate pathway*

Transketolase activity was added to gene 495. EC 5.3.1.6, ribose-5-phosphate isomerase, was added to gene 57.

*Alternative sugar metabolism*

Mannose PTS transporter activity was assigned to gene 555. EC 1.1.1.17, mannitol-1-phosphate 5-dehydrogenase, was assigned to gene 37.

*Nucleotide metabolism*

(Deoxy-)Adenylate kinase activity was assigned to gene 373 based on the presence of protein domains (EC2.7.4.3 and EC2.7.4.13). Nucleoside phosphorylase activity was added to gene 463 (EC 2.4.2.1)

For a number of enzymes broad substrate specificity was assumed (Pollack, 2002):

- Pyruvate kinase (EC 2.7.1.40) was assumed to convert: dTDP, dCDP, UDP, CDP, ADP, dGDP, dADP and GDP.
- 5’-nucleotidase (EC 3.1.3.5) was assumed to convert: CMP, dTMP, dUMP, dCMP, UMP, GMP, dGMP and AMP.
- Phosphoglycerate kinase, no annotated gene, was assumed to catalyze reactions EC 2.7.2.3 and 2.7.2.10 and convert the following substrates: GTP, dGTP, ATP and dATP.
- Guanylate kinase (gene 395) was assumed to convert dGMP (EC 2.7.4.13) and GMP (EC 2.7.4.8).
- Ribonucleotide reductase (EC 1.17.4.1) was assumed to convert GDP, UDP, CDP and ADP.
- Purine-nucleoside phosphorylase (EC 2.4.2.1) was assumed to convert adenosine, deoxyadenosine, guanosine and deoxyguanosine.
- Cytidylate kinase (EC 2.7.4.14) was assumed to convert CMP and dCMP.

Thymidylate synthase (EC 2.1.1.45) is assumed not to be present.

*Folate metabolism*

There is just one reaction in folate metabolism: EC 2.1.2.1 glycine hydroxymethyltransferase, folate containing substrates for these reactions will need to be obtained from the medium.

Co-factor metabolism

Gene 539 annotated as a cytidylyltransferase was assumed to catalyze reactions: EC 2.7.7.15 choline-phosphate cytidylyltransferase, EC 2.7.8.18 nicotinate-nucleotide adenylyltransferase and EC 2.7.7.3 pantetheine-phosphate adenylyltransferase. Reaction 2.7.1.24, dephosphocoenzyme A kinase was assigned to gene 651 because of the presence of domain IPR001977. Reaction EC 3.1.4.14 was assigned to genes 516 and 517 based on domain content.

Superpathway removal

Many superpathways were assumed to be present by pathwaytools but lack sufficient evidence for presence in *M. hyopneumoniae*. The following superpathways were removed:

- Mixed acid fermentation
- Glycerol degradation I and II
- PreQ biosynthesis
- Phosphatidylethanolamine biosynthesis
- 6-hydroxymethyl-dihydropterin diphosphate biosynthesis I
- Pyrimidine and ribonucleotide de novo biosynthesis
- Adenosine nucleotide de novo biosynthesis
- Guanosine nucleotides de novo biosynthesis
- Xanthine and xanthosine salvage
- tRNA processing
- UMP biosynthesis
- NAD biosynthesis
- CoA biosynthesis
- 5-dehydro-4-deoxy-glucuronate degradation
- Guanosine nucleotides degradation III
- Triacylglycerol degradation
- Adenine and adenosine salvage III
- Purine ribonucleotides degradation

Reactions directionalities were mainly obtained from the metabolic model of *M. pneumoniae* or from *M. hyopneumoniae* (Ferrarini et al., 2016; Wodke et al., 2013).

*First order Michaelis-Menten equations*

$v_{g}=V_{g,max}\times\frac{[glucose]}{(K_{g}+\left[ glucose \right])}$ 1

$v_{l}=V_{l,max}\times\frac{[lactate]}{(K_{l}+\left[ lactate \right])}$ 2

$v_{p}=V_{p,max}\times\frac{[pyruvate]}{(K_{p}+\left[ pyruvate \right])}$ 3

$v_{glyc}=V_{glyc,max}\times\frac{[glycerol]}{(K_{l}+\left[ glycerol \right])}$ 4

# *Differential equations used in the dynamic flux balance model:*

$\frac{dX}{dt}=\mu\cdot X$ 5

$\frac{dG}{dt}=v_{g}\cdot X$ 6

$\frac{dL}{dt}=v_{l}\cdot X$ 7

$\frac{dP}{dt}=v_{p}\cdot X$ 8

$\frac{dGlyc}{dt}=v_{glyc}\cdot X$ 9

$\frac{dA}{dt}=v_{a}\cdot X$ 10

# Supplementary figures

**Fig. S1. Dissolved oxygen concentration in aerobic fermentations.** Dashed lines show measured values and the solid lines shows values assumed during dynamic modeling. A: Measured dissolved oxygen concentration in aerobic batches grown on glucose. Solid line was assumed for the model based on a second order polynomial fit (-0.0317*t^2-3.1847*t+81.837). B: Measured dissolved oxygen concentration in aerobic batches grown on glucose and pyruvate. Solid line was assumed for the model based on a second order polynomial fit (-0.0638*t^2-3.0848*t+101.83).


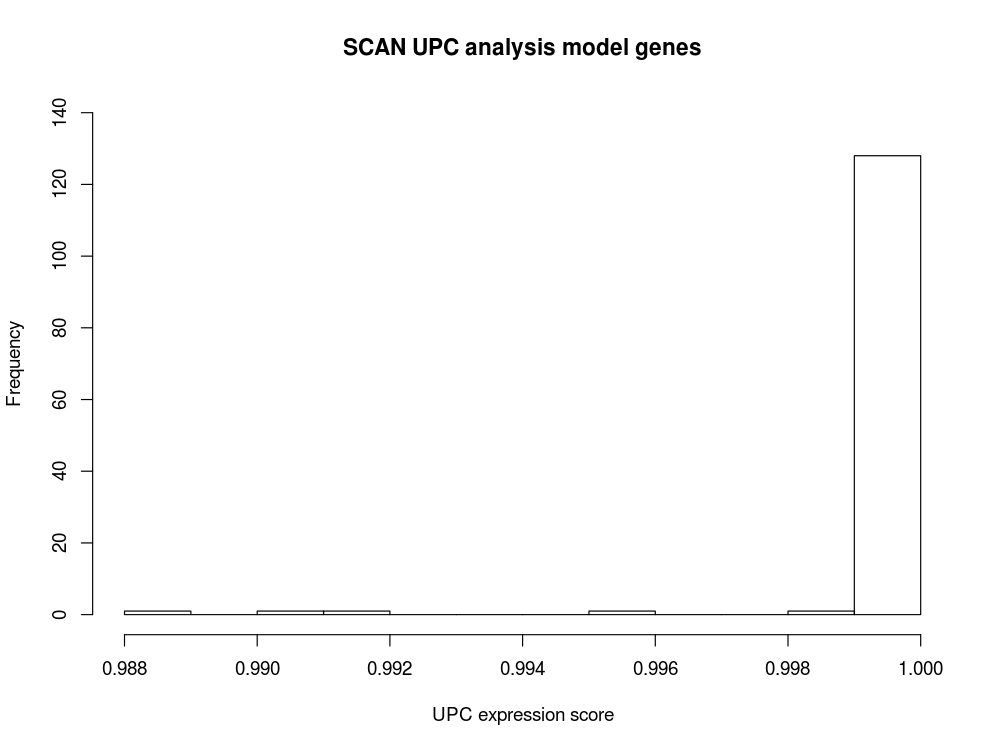


**Fig. S2: SCAN_UPC analysis for genes in TK284-MHyo11.** Evaluation of gene expression was done based on read counts per gene present in the genome-scale model using the package SCAN_UPC (Piccolo et al., 2012; Piccolo, Withers, Francis, Bild, & Johnson, 2013). UPC expression scores are high or 1 for all genes in the model indicating that these genes were expressed. Calculations were done with reads counts obtained from the fermentation with basic FRIIS medium with only glucose added.

# References

Boetzer, M., Pirovano, W., Zerbino, D., Birney, E., Simpson, J., Wong, K., … Jaffe, D. (2012). Toward almost closed genomes with GapFiller. *Genome Biology*, *13*(6), R56. http://doi.org/10.1186/gb-2012-13-6-r56

Chaisson, M. J., & Tesler, G. (2012). Mapping single molecule sequencing reads using basic local alignment with successive refinement (BLASR): application and theory. *BMC Bioinformatics*, *13*, 238. http://doi.org/10.1186/1471-2105-13-238

Ferrarini, M. G., Siqueira, F. M., Mucha, S. G., Palama, T. L., Jobard, É., Elena-Herrmann, B., … Sagot, M.-F. (2016). Insights on the virulence of swine respiratory tract mycoplasmas through genome-scale metabolic modeling. *BMC Genomics*, *17*(1), 353. http://doi.org/10.1186/s12864-016-2644-z

Hwang, F., Wen, D. C., Wu, Y. W., Li, Y. Z., Dong, Q. H., & Wang, S. M. (1986). Studies on the phospholipid composition of pathogenic cell membranes of Mycoplasma hyopneumoniae. *FEBS Letters*, *195*(1–2), 323–6.

Langmead, B., & Salzberg, S. L. (2012). Fast gapped-read alignment with Bowtie 2. *Nat Methods*, *9*(4), 357–359. http://doi.org/10.1038/nmeth.1923

Piccolo, S. R., Sun, Y., Campbell, J. D., Lenburg, M. E., Bild, A. H., & Johnson, W. E. (2012). A single-sample microarray normalization method to facilitate personalized-medicine workflows. *Genomics*, *100*(6), 337–344. http://doi.org/10.1016/j.ygeno.2012.08.003

Piccolo, S. R., Withers, M. R., Francis, O. E., Bild, A. H., & Johnson, W. E. (2013). Multiplatform single-sample estimates of transcriptional activation. *Proceedings of the National Academy of Sciences of the United States of America*, *110*(44), 17778–83. http://doi.org/10.1073/pnas.1305823110

Pollack, J. D. (2002). The necessity of combining genomic and enzymatic data to infer metabolic function and pathways in the smallest bacteria: amino acid, purine and pyrimidine metabolism in Mollicutes. *Frontiers in Bioscience*, *7*, 1762–1781.

Wodke, J. a H., Puchałka, J., Lluch-Senar, M., Marcos, J., Yus, E., Godinho, M., … Maier, T. (2013). Dissecting the energy metabolism in Mycoplasma pneumoniae through genome-scale metabolic modeling. *Molecular Systems Biology*, *9*(653), 653. http://doi.org/10.1038/msb.2013.6
