# Supplementary material for: Metabolic modeling of energy balances in Mycoplasma hyopneumoniae shows that pyruvate addition increases growth rate
Source: Biotechnol Bioeng. 2017 Jul 27;114(10):2339–47. doi: 10.1002/bit.26347 (PMC6084303; doi:10.1002/bit.26347)
Supplement: Supplementary file 2 — Table S1. Initial model constraints for simulations in medium with glucose and early growth in medium with glucose and pyruvate. Highlighted in yellow are the model constraints that were changed in the simulations with pyruvate present. [file BIT-114-2339-s002.docx]

| Table S1: Initial model constraints for simulations in medium with glucose and early growth in medium with glucose and pyruvate. Highlighted in yellow are the model constraints that were changed in the simulations with pyruvate present. | | | | | |
| --- | --- | --- | --- | --- | --- |
|  |  | |  |  |  |
|  | Glucose | | | Glucose + Pyruvate (early growth) | |
| **Reaction** | **Lower bound** | **Upper bound** | | **Lower bound** | **Upper bound** |
| 'RXN01061' | 18.41 | 1000 | | 10.15 | 1000 |
| 'A3__46__6__46__5__46__3RXN' | 0 | 1000 | | 0 | 1000 |
| 'RXN18KM18' | 0 | 1000 | | 0 | 1000 |
| 'URACILPRIBOSYLTRANSRXN' | -1000 | 1000 | | -1000 | 1000 |
| 'TRANSRXN168' | 0 | 1000 | | 0 | 1000 |
| 'DCTPPYROPHOSPHATASERXN' | 0 | 1000 | | 0 | 1000 |
| 'RXN2902' | 0 | 1000 | | 0 | 1000 |
| 'DUTPPYROPRXN' | 0 | 1000 | | 0 | 1000 |
| 'A4__46__1__46__2__46__29RXN' | 0 | 1000 | | 0 | 1000 |
| 'RXN18KM8' | 0 | 1000 | | 0 | 1000 |
| 'MYOINOSITOL2DEHYDROGENASERXN' | 0 | 1000 | | 0 | 1000 |
| 'RXN05292' | 0 | 1000 | | 0 | 1000 |
| 'A2__46__7__46__7__46__15RXN' | 0 | 1000 | | 0 | 1000 |
| 'CDPDIGLYSYNRXN' | 0 | 1000 | | 0 | 1000 |
| 'RXN1381' | 0 | 1000 | | 0 | 1000 |
| 'RXN05375' | -1000 | 1000 | | -1000 | 1000 |
| 'NACETYLGLUCOSAMINEKINASERXN' | 0 | 1000 | | 0 | 1000 |
| 'GTPCYCLOHYDROIRXN' | -1000 | 1000 | | -1000 | 1000 |
| 'CHOLINEKINASERXN' | 0 | 1000 | | 0 | 1000 |
| 'ATPSYNRXN' | -1000 | 1000 | | -1000 | 1000 |
| 'RXN3523' | 0 | 1000 | | 0 | 1000 |
| 'THYMIDYLATE5PHOSPHATASERXN' | 0 | 1000 | | 0 | 1000 |
| 'GAPOXNPHOSPHNRXN' | -1000 | 1000 | | -1000 | 1000 |
| 'R503RXN' | 0 | 1000 | | 0 | 1000 |
| 'GMKALTRXN' | 0 | 1000 | | 0 | 1000 |
| 'RXN05305' | -1000 | 1000 | | -1000 | 1000 |
| 'LACTOSE6PHOSPHATEISOMERASERXN' | -1000 | 1000 | | -1000 | 1000 |
| 'RXN18KM6' | 0 | 1000 | | 0 | 1000 |
| 'GLUCOSAMINE6PDEAMINRXN' | 0 | 1000 | | 0 | 1000 |
| 'RIBULP3EPIMRXN' | -1000 | 1000 | | -1000 | 1000 |
| 'DEOXYADENYLATEKINASERXN' | 0 | 1000 | | 0 | 1000 |
| 'RXN11811' | -1000 | 1000 | | -1000 | 1000 |
| 'RXN05199' | -1000 | 1000 | | -1000 | 1000 |
| 'RXN10981' | 0 | 1000 | | 0 | 1000 |
| 'RXN12149' | -1000 | 1000 | | -1000 | 1000 |
| 'A1TRANSKETORXN' | -1000 | 1000 | | -1000 | 1000 |
| 'PGPPHOSPHARXN' | 0 | 1000 | | 0 | 1000 |
| 'A2TRANSKETORXN' | -1000 | 1000 | | -1000 | 1000 |
| 'RXN12440' | 0 | 1000 | | 0 | 1000 |
| 'RXN1623' | 0 | 1000 | | 0 | 1000 |
| 'RXN12754' | 0 | 1000 | | 0 | 1000 |
| 'DEOXYGUANPHOSPHORRXN' | -1000 | 1000 | | -1000 | 1000 |
| 'DEOXYCYTIDINEKINASERXN' | 0 | 1000 | | 0 | 1000 |
| 'RXN12753' | 0 | 1000 | | 0 | 1000 |
| 'ADENPHOSPHORRXN' | -1000 | 1000 | | -1000 | 1000 |
| 'RXN12862' | 0 | 1000 | | 0 | 1000 |
| 'RXN8141' | -1000 | 1000 | | -1000 | 1000 |
| 'PANTEPADENYLYLTRANRXN' | 0 | 1000 | | 0 | 1000 |
| 'TRANSRXN104' | -1000 | 1000 | | -1000 | 1000 |
| 'RXN18KM2' | 0 | 1000 | | 0 | 1000 |
| 'RXN0705' | 0 | 1000 | | 0 | 1000 |
| 'NADHDEHYDROGENASERXN' | -1000 | 1000 | | -1000 | 1000 |
| 'RIBULPEPIMRXN' | -1000 | 1000 | | -1000 | 1000 |
| 'A1__46__2__46__1__46__27RXN' | -1000 | 1000 | | -1000 | 1000 |
| 'PHOSPHOGLYCERATEKINASEGTPRXN' | -1000 | 1000 | | -1000 | 1000 |
| 'RXN12863' | 0 | 1000 | | 0 | 1000 |
| 'RXN12869' | 0 | 1000 | | 0 | 1000 |
| 'RXN18KM13' | -1000 | 1000 | | -1000 | 1000 |
| 'RXN12861' | 0 | 1000 | | 0 | 1000 |
| 'RXN14143' | 0 | 1000 | | 0 | 1000 |
| 'A5__46__3__46__1__46__17RXN' | -1000 | 1000 | | -1000 | 1000 |
| 'RXN8654' | 0 | 1000 | | 0 | 1000 |
| 'RXN02461' | 0 | 1000 | | 0 | 1000 |
| 'RIBOFLAVINKINRXN' | 0 | 1000 | | 0 | 1000 |
| 'ETHANOLAMINEKINASERXN' | -1000 | 1000 | | -1000 | 1000 |
| 'AMPDEPHOSPHORYLATIONRXN' | 0 | 1000 | | 0 | 1000 |
| 'RXN14142' | 0 | 1000 | | 0 | 1000 |
| 'RXN7609' | 0 | 1000 | | 0 | 1000 |
| 'RXN12872' | 0 | 1000 | | 0 | 1000 |
| 'RXN14025' | 0 | 1000 | | 0 | 1000 |
| 'RXN14026' | 0 | 1000 | | 0 | 1000 |
| 'RXN05214' | -1000 | 1000 | | -1000 | 1000 |
| 'RXN12870' | 0 | 1000 | | 0 | 1000 |
| 'RXN14150' | -1000 | 1000 | | -1000 | 1000 |
| 'GUANPRIBOSYLTRANRXN' | -1000 | 1000 | | -1000 | 1000 |
| 'RXN18KM10' | 0 | 1000 | | 0 | 1000 |
| 'RXN12871' | 0 | 1000 | | 0 | 1000 |
| 'RXN18KM15' | -1000 | 1000 | | -1000 | 1000 |
| 'TRANSALDOLRXN' | -1000 | 1000 | | -1000 | 1000 |
| 'A3__46__1__46__4__46__2RXN' | 0 | 1000 | | 0 | 1000 |
| 'RIB5PISOMRXN' | -1000 | 1000 | | -1000 | 1000 |
| 'RXN18KM7' | 0 | 1000 | | 0 | 1000 |
| 'A3__46__6__46__3__46__20RXN' | -1000 | 1000 | | -1000 | 1000 |
| 'DEOXYADENPHOSPHORRXN' | -1000 | 1000 | | -1000 | 1000 |
| 'MANNPDEHYDROGRXN' | -1000 | 1000 | | -1000 | 1000 |
| 'TRANSRXN156' | 0 | 1000 | | 0 | 1000 |
| 'PHOSACETYLTRANSRXN' | -1000 | 1000 | | -1000 | 1000 |
| 'ACETATEKINRXN' | -1000 | 1000 | | -1000 | 1000 |
| 'A3__46__6__46__3__46__17RXN' | -1000 | 1000 | | -1000 | 1000 |
| 'NICONUCADENYLYLTRANRXN' | -1000 | 1000 | | -1000 | 1000 |
| 'TRANSRXN131' | 0 | 1000 | | 0 | 1000 |
| 'LXULRU5PRXN' | -1000 | 1000 | | -1000 | 1000 |
| 'GLYCEROL3PHOSPHATEOXIDASERXN' | -1000 | 1000 | | -1000 | 1000 |
| 'RXN3715' | 0 | 1000 | | 0 | 1000 |
| 'RXN11832' | -1000 | 1000 | | -1000 | 1000 |
| 'RXN7913' | -1000 | 1000 | | -1000 | 1000 |
| 'RXN8631' | 0 | 1000 | | 0 | 1000 |
| 'F16ALDOLASERXN' | -1000 | 1000 | | -1000 | 1000 |
| 'PRPPSYNRXN' | 0 | 1000 | | 0 | 1000 |
| 'DURIDKIRXN' | 0 | 1000 | | 0 | 1000 |
| 'THYKIRXN' | 0 | 1000 | | 0 | 1000 |
| 'INORGPYROPHOSPHATRXN' | 0 | 1000 | | 0 | 1000 |
| 'A3PGAREARRRXN' | -1000 | 1000 | | -1000 | 1000 |
| 'NAG6PDEACETRXN' | 0 | 1000 | | 0 | 1000 |
| 'DEPHOSPHOCOAKINRXN' | 0 | 1000 | | 0 | 1000 |
| 'RXN13720' | -1000 | 1000 | | -1000 | 1000 |
| 'DEOXYRIBOSEPALDRXN' | 0 | 1000 | | 0 | 1000 |
| 'URAPHOSPHRXN' | -1000 | 1000 | | -1000 | 1000 |
| 'URPHOSRXN' | -1000 | 1000 | | -1000 | 1000 |
| 'THYMPHOSPHRXN' | -1000 | 1000 | | -1000 | 1000 |
| 'TRIOSEPISOMERIZATIONRXN' | -1000 | 1000 | | -1000 | 1000 |
| 'A6PFRUCTPHOSRXN' | 0 | 1000 | | 0 | 1000 |
| 'ADENPRIBOSYLTRANRXN' | -1000 | 1000 | | -1000 | 1000 |
| 'PEPDEPHOSRXN' | 0 | 1000 | | 0 | 1000 |
| 'RXN14207' | 0 | 1000 | | 0 | 1000 |
| 'RXN14192' | 0 | 1000 | | 0 | 1000 |
| 'RXN14117' | 0 | 1000 | | 0 | 1000 |
| 'LLACTATEDEHYDROGENASERXN' | -1000 | 1000 | | -1000 | 1000 |
| 'GUANYLKINRXN' | 0 | 1000 | | 0 | 1000 |
| 'CYTIDEAMRXN' | 0 | 1000 | | 0 | 1000 |
| 'CYTIDEAM2RXN' | 0 | 1000 | | 0 | 1000 |
| 'DPPENTOMUTRXN' | -1000 | 1000 | | -1000 | 1000 |
| 'PPENTOMUTRXN' | -1000 | 1000 | | -1000 | 1000 |
| 'ADENYLKINRXN' | 0 | 1000 | | 0 | 1000 |
| 'GLYOHMETRANSRXN' | -1000 | 1000 | | -1000 | 1000 |
| 'A5DEHYDRO2DEOXYGLUCONOKINASERXN' | 0 | 1000 | | 0 | 1000 |
| 'MYOINOSOSE2DEHYDRATASERXN' | 0 | 1000 | | 0 | 1000 |
| 'A2PGADEHYDRATRXN' | -1000 | 1000 | | -1000 | 1000 |
| 'DTMPKIRXN' | -1000 | 1000 | | -1000 | 1000 |
| 'FADSYNRXN' | 0 | 1000 | | 0 | 1000 |
| 'CTPSYNRXN' | 0 | 1000 | | 0 | 1000 |
| 'PHOSPHAGLYPSYNRXN' | 0 | 1000 | | 0 | 1000 |
| 'DIHYDLIPOXNRXN' | -1000 | 1000 | | -1000 | 1000 |
| 'MANNPISOMRXN' | -1000 | 1000 | | -1000 | 1000 |
| 'PHOSGLYPHOSRXN' | -1000 | 1000 | | -1000 | 1000 |
| 'NADSYNTHNH3RXN' | 0 | 1000 | | 0 | 1000 |
| 'SADENMETSYNRXN' | 0 | 1000 | | 0 | 1000 |
| 'OROTPDECARBRXN' | 0 | 1000 | | 0 | 1000 |
| 'GLYCEROLKINRXN' | 0 | 1000 | | 0 | 1000 |
| 'A6__46__3__46__5__46__7RXN' | 0 | 1000 | | 0 | 1000 |
| 'RXN12460' | -1000 | 1000 | | -1000 | 1000 |
| 'RXN9386' | 0 | 1000 | | 0 | 1000 |
| 'ASPARTATETRNALIGASERXN' | 0 | 1000 | | 0 | 1000 |
| 'GLUTAMINETRNALIGASERXN' | 0 | 1000 | | 0 | 1000 |
| 'TYROSINETRNALIGASERXN' | 0 | 1000 | | 0 | 1000 |
| 'GLYCINETRNALIGASERXN' | 0 | 1000 | | 0 | 1000 |
| 'ISOLEUCINETRNALIGASERXN' | 0 | 1000 | | 0 | 1000 |
| 'ARGININETRNALIGASERXN' | 0 | 1000 | | 0 | 1000 |
| 'VALINETRNALIGASERXN' | 0 | 1000 | | 0 | 1000 |
| 'LEUCINETRNALIGASERXN' | 0 | 1000 | | 0 | 1000 |
| 'CYSTEINETRNALIGASERXN' | 0 | 1000 | | 0 | 1000 |
| 'TRYPTOPHANTRNALIGASERXN' | 0 | 1000 | | 0 | 1000 |
| 'THREONINETRNALIGASERXN' | 0 | 1000 | | 0 | 1000 |
| 'GLURSRXN' | 0 | 1000 | | 0 | 1000 |
| 'ALANINETRNALIGASERXN' | 0 | 1000 | | 0 | 1000 |
| 'LYSINETRNALIGASERXN' | 0 | 1000 | | 0 | 1000 |
| 'HISTIDINETRNALIGASERXN' | 0 | 1000 | | 0 | 1000 |
| 'SERINETRNALIGASERXN' | 0 | 1000 | | 0 | 1000 |
| 'PHENYLALANINETRNALIGASERXN' | 0 | 1000 | | 0 | 1000 |
| 'ASPARAGINETRNALIGASERXN' | 0 | 1000 | | 0 | 1000 |
| 'METHIONINETRNALIGASERXN' | 0 | 1000 | | 0 | 1000 |
| 'PROLINETRNALIGASERXN' | 0 | 1000 | | 0 | 1000 |
| 'THIOREDOXINREDUCTNADPHRXN' | -1000 | 1000 | | -1000 | 1000 |
| 'A3__46__1__46__4__46__14RXN' | 0 | 1000 | | 0 | 1000 |
| 'RXN01134' | 0 | 1000 | | 0 | 1000 |
| 'RXN01132' | -1000 | 1000 | | -1000 | 1000 |
| 'GDPREDUCTRXN' | 0 | 1000 | | 0 | 1000 |
| 'HOLOACPSYNTHRXN' | 0 | 1000 | | 0 | 1000 |
| 'RXN01133' | -1000 | 1000 | | -1000 | 1000 |
| 'CDPREDUCTRXN' | 0 | 1000 | | 0 | 1000 |
| 'UDPREDUCTRXN' | 0 | 1000 | | 0 | 1000 |
| 'ADPREDUCTRXN' | 0 | 1000 | | 0 | 1000 |
| 'RXN18KM3' | -1000 | 1000 | | -1000 | 1000 |
| 'TRANSRXN18KM6' | 0 | 1000 | | 0 | 1000 |
| 'carbonate_co2' | -1000 | 1000 | | -1000 | 1000 |
| 'transport_alanine' | 0 | 1000 | | 0 | 1000 |
| 'transport_arginine' | 0 | 1000 | | 0 | 1000 |
| 'transport_L-asparagine' | 0 | 1000 | | 0 | 1000 |
| 'transport_L-aspartate' | 0 | 1000 | | 0 | 1000 |
| 'transport_L-cysteine' | 0 | 1000 | | 0 | 1000 |
| 'transport_L-glutamate' | 0 | 1000 | | 0 | 1000 |
| 'transport_L-glutamine' | 0 | 1000 | | 0 | 1000 |
| 'transport_L-glycine' | 0 | 1000 | | 0 | 1000 |
| 'transport_L-histidine' | 0 | 1000 | | 0 | 1000 |
| 'transport_L-isoleucine' | 0 | 1000 | | 0 | 1000 |
| 'transport_L-leucine' | 0 | 1000 | | 0 | 1000 |
| 'transport_L-lysine' | 0 | 1000 | | 0 | 1000 |
| 'transport_L-methionine' | 0 | 1000 | | 0 | 1000 |
| 'transport_L-phenylalanine' | 0 | 1000 | | 0 | 1000 |
| 'transport_L-proline' | 0 | 1000 | | 0 | 1000 |
| 'transport_L-serine' | 0 | 1000 | | 0 | 1000 |
| 'transport_L-threonine' | 0 | 1000 | | 0 | 1000 |
| 'transport_L-tryptophan' | 0 | 1000 | | 0 | 1000 |
| 'transport_L-tyrosine' | 0 | 1000 | | 0 | 1000 |
| 'transport_L-valine' | 0 | 1000 | | 0 | 1000 |
| 'transport_guanine' | 0 | 1000 | | 0 | 1000 |
| 'transport_uracil' | 0 | 1000 | | 0 | 1000 |
| 'transport_adenine' | -1000 | 1000 | | -1000 | 1000 |
| 'transport_thymine' | 0 | 1000 | | 0 | 1000 |
| 'transport_cytidine' | 0 | 1000 | | 0 | 1000 |
| 'transport_deoxycytidine' | 0 | 1000 | | 0 | 1000 |
| 'transport_phosphate_in' | 0 | 1000 | | 0 | 1000 |
| 'transport_acetate' | 0 | 1000 | | 0 | 1000 |
| 'transport_pyruvate' | 0 | 1000 | | 0 | 1000 |
| 'transport_CARBONDIOXIDE' | -1000 | 1000 | | -1000 | 1000 |
| 'transport_WATER' | -1000 | 1000 | | -1000 | 1000 |
| 'transport_OXYGENMOLECULE' | -1000 | 1000 | | -1000 | 1000 |
| 'transport_AMMONIA' | -1000 | 1000 | | -1000 | 1000 |
| 'transport_Riboflavin' | 0 | 1000 | | 0 | 1000 |
| 'transport_H2O2' | 0 | 1000 | | 0 | 1000 |
| 'transport_FA' | -1000 | 1000 | | -1000 | 1000 |
| 'transport_panthetheine' | -1000 | 1000 | | -1000 | 1000 |
| 'transport_PHOSPHATIDYL_CHOLINE' | -1000 | 1000 | | -1000 | 1000 |
| 'transport_MYO_INOSITOL' | -1000 | 1000 | | -1000 | 1000 |
| 'transport_phosphate_out' | 0 | 1000 | | 0 | 1000 |
| 'transport_niacine' | 0 | 1000 | | 0 | 1000 |
| 'EX_PROTON_e' | -1000 | 1000 | | -1000 | 1000 |
| 'EX_RIBOSE_e' | -1000 | 1000 | | -1000 | 1000 |
| 'EX_LLACTATE_e' | 0 | 1000 | | 6.5 | 1000 |
| 'EX_FRU_e' | -1000 | 1000 | | -1000 | 1000 |
| 'EX_ASCORBATE_e' | 0 | 0 | | 0 | 0 |
| 'EX_MANNOSE_e' | 0 | 0 | | 0 | 0 |
| 'EX_CPD4422_e' | -1000 | 1000 | | -1000 | 1000 |
| 'EX_GLYCEROL_e' | -0.51 | 0 | | -0.31 | 0 |
| 'EX_SER_e' | -1000 | 1000 | | -1000 | 1000 |
| 'EX_GLYCEROL3P_e' | -0.1277 | 0 | | -0.1277 | 0 |
| 'EX_MANNITOL_e' | 0 | 0 | | 0 | 0 |
| 'EX_DGlucose_e' | -5.11 | 0 | | -2.3 | 0 |
| 'EX_LALPHAALANINE_e' | -1000 | 1000 | | -1000 | 1000 |
| 'EX_ARG_e' | -1000 | 1000 | | -1000 | 1000 |
| 'EX_ASN_e' | -1000 | 1000 | | -1000 | 1000 |
| 'EX_LASPARTATE_e' | -1000 | 1000 | | -1000 | 1000 |
| 'EX_CYS_e' | -1000 | 1000 | | -1000 | 1000 |
| 'EX_GLT_e' | -1000 | 1000 | | -1000 | 1000 |
| 'EX_GLN_e' | -1000 | 1000 | | -1000 | 1000 |
| 'EX_GLY_e' | -1000 | 1000 | | -1000 | 1000 |
| 'EX_HIS_e' | -1000 | 1000 | | -1000 | 1000 |
| 'EX_ILE_e' | -1000 | 1000 | | -1000 | 1000 |
| 'EX_LEU_e' | -1000 | 1000 | | -1000 | 1000 |
| 'EX_LYS_e' | -1000 | 1000 | | -1000 | 1000 |
| 'EX_MET_e' | -1000 | 1000 | | -1000 | 1000 |
| 'EX_PHE_e' | -1000 | 1000 | | -1000 | 1000 |
| 'EX_PRO_e' | -1000 | 1000 | | -1000 | 1000 |
| 'EX_THR_e' | -1000 | 1000 | | -1000 | 1000 |
| 'EX_TRP_e' | -1000 | 1000 | | -1000 | 1000 |
| 'EX_TYR_e' | -1000 | 1000 | | -1000 | 1000 |
| 'EX_VAL_e' | -1000 | 1000 | | -1000 | 1000 |
| 'EX_GUANINE_e' | -1000 | 1000 | | -1000 | 1000 |
| 'EX_URACIL_e' | -1000 | 1000 | | -1000 | 1000 |
| 'EX_ADENINE_e' | -1000 | 1000 | | -1000 | 1000 |
| 'EX_THYMINE_e' | -1000 | 1000 | | -1000 | 1000 |
| 'EX_CYTIDINE_e' | -1000 | 1000 | | -1000 | 1000 |
| 'EX_DEOXYCYTIDINE_e' | -1000 | 1000 | | -1000 | 1000 |
| 'EX_Pi_e' | -1000 | 1000 | | -1000 | 1000 |
| 'EX_ACET_e' | -1000 | 1000 | | -1000 | 1000 |
| 'EX_PYRUVATE_e' | 0 | 0 | | -8.17 | 0 |
| 'EX_CARBONDIOXIDE__e' | -1000 | 1000 | | -1000 | 1000 |
| 'EX_WATER_e' | -1000 | 1000 | | -1000 | 1000 |
| 'EX_OXYGENMOLECULE__e' | -1000 | 1000 | | -1000 | 1000 |
| 'EX_AMMONIA__e' | -1000 | 1000 | | -1000 | 1000 |
| 'EX_RIBOFLAVIN_e' | -1000 | 1000 | | -1000 | 1000 |
| 'EX_HYDROGENPEROXIDE_e' | -1000 | 1000 | | -1000 | 1000 |
| 'EX_LongChainFattyAcids_e' | -1000 | 1000 | | -1000 | 1000 |
| 'EX_PANTETHEINEP_e' | -1000 | 1000 | | -1000 | 1000 |
| 'EX_PHOSPHATIDYL_CHOLINE_e' | -1000 | 1000 | | -1000 | 1000 |
| 'EX_MYOINOSITOL_e' | 0 | 0 | | 0 | 0 |
| 'EX_NIACINE_e' | -1000 | 1000 | | -1000 | 1000 |
| 'Protein_synthesis' | 0 | 1000 | | 0 | 1000 |
| 'Protein_synthesis_gram' | -1000 | 1000 | | -1000 | 1000 |
| 'ACP_synthesis' | 0 | 1000 | | 0 | 1000 |
| 'ACP_synthesis_gram' | -1000 | 1000 | | -1000 | 1000 |
| 'Protein_degradation' | 0.0003489 | 1000 | | 0.0003489 | 1000 |
| 'ACP_degradation' | 0 | 1000 | | 0 | 1000 |
| 'DNA_synthesis' | 0 | 1000 | | 0 | 1000 |
| 'DNA_synthesis_gram' | -1000 | 1000 | | -1000 | 1000 |
| 'RNA_synthesis' | 0 | 1000 | | 0 | 1000 |
| 'RNA_synthesis_gram' | -1000 | 1000 | | -1000 | 1000 |
| 'RNA_degradation' | 0.007741 | 1000 | | 0.007741 | 1000 |
| 'LPHOSPHATIDATE_synthesis_gram' | -1000 | 1000 | | -1000 | 1000 |
| 'CARDIOLIPIN_synthesis_gram' | -1000 | 1000 | | -1000 | 1000 |
| 'PHOSPHATIDYL_CHOLINE_gram' | -1000 | 1000 | | -1000 | 1000 |
| 'LIPID_synthesis' | 0 | 1000 | | 0 | 1000 |
| 'G6P_gram' | -1000 | 1000 | | -1000 | 1000 |
| 'AAbiomass_mol' | 0 | 1000 | | 0 | 1000 |
| 'AAbiomass_gram' | -1000 | 1000 | | -1000 | 1000 |
| 'Biomass_synthesis' | 0 | 1000 | | 0 | 1000 |
| 'EX_BIOMASS_g_c' | -1000 | 1000 | | -1000 | 1000 |
| 'DEOXYGUANOSINEKINASERXN' | -1000 | 1000 | | -1000 | 1000 |
| 'DEOXYADENOSINEKINASERXN' | -1000 | 1000 | | -1000 | 1000 |
| 'UMPKINASERXN' | -1000 | 1000 | | -1000 | 1000 |
| 'ACYLCOASYNTHRXN' | -1000 | 1000 | | -1000 | 1000 |
| 'NADKINRXN' | -1000 | 1000 | | -1000 | 1000 |
| 'NADHKINRXN' | -1000 | 1000 | | -1000 | 1000 |
