# Supplementary material for: Metabolic modeling of energy balances in Mycoplasma hyopneumoniae shows that pyruvate addition increases growth rate
Source: Biotechnol Bioeng. 2017 Jul 27;114(10):2339–47. doi: 10.1002/bit.26347 (PMC6084303; doi:10.1002/bit.26347)
Supplement: Supplementary file 3 — Table S2. Metabolite starting concentrations FRIIS medium. [file BIT-114-2339-s003.docx]

| Table S2: Metabolite starting concentrations FRIIS medium | | | | |  |  |  |  |  |  |  |  |  |  |  |  |
| --- | --- | --- | --- | --- | --- | --- | --- | --- | --- | --- | --- | --- | --- | --- | --- | --- |
|  |  |  |  |  |  |  |  |  |  |  |  |  |  |  |  |  |
| Substrate | Substrate concentration (mM) | | | | | | | | | | | | |  |  |  |
|  | Lot. A | | | | | | | | | Lot. B | Lot. C | Lot. D | | Average (mM) |  | STDEV |
| Glucose | 2.31 | 1.96 | 2.46 | 2.12 | 2.37 | N.A. | N.A. | N.A. | N.A. | 3.59 | 2.90 | N.A. | N.A. | 2.91 | ± | 0.67 |
| Pyruvate | <LOD | <LOD | <LOD | <LOD | <LOD | N.A. | <LOD | <LOD | N.A. | N.D. | N.D. | <LOD | N.A. | <LOD |  |  |
| Lactate | 1.03 | 1.19 | 0.99 | 1.34 | 1.08 | 1.37 | 1.34 | 1.43 | N.A. | 1.58 | 1.73 | 1.39 | 1.39 | 1.46 | ± | 0.20 |
| Acetate | 0.91 | 0.98 | <LOD | <LOD | <LOD | <LOD | 1.06 | 1.09 | 1.35 | N.D. | N.D. | 1.87 | 1.67 | 1.54 | ± | 0.41 |
| Formate | <LOD | <LOD | <LOD | <LOD | <LOD | N.A. | 0.12 | 0.17 | 0.17 | N.D. | N.D. | 0.13 | 0.21 | 0.16 | ± | 0.04 |
| Glycerol | 0.25 | 0.26 | 0.29 | 0.36 | 0.29 | N.A. | 0.28 | 0.29 | 0.31 | N.D. | N.D. | 0.30 | 0.30 | 0.30 | ± | 0.00 |
| Ribose | 0.02 | 0.01 | 0.01 | 0.02 | 0.02 | N.A. | 0.03 | 0.02 | 0.03 | N.D. | N.D. | 0.04 | 0.04 | 0.03 | ± | 0.01 |
| Myo-inositol | 0.18 | 0.20 | 0.16 | 0.22 | 0.21 | N.A. | N.D. | N.D. | N.D. | N.D. | N.D. | N.D. | N.D. | 0.19 | ± | 0.02 |
| Fructose | 0.35 | 0.06 | 0.17 | 0.18 | 0.26 | N.A. | N.A. | N.A. | N.A. | N.D. | N.D. | N.A. | N.A. | 0.20 | ± | 0.11 |
| Mannose | 0.13 | 0.14 | 0.12 | 0.15 | 0.13 | 0.18 | N.D. | N.D. | N.D. | N.D. | N.D. | N.D. | N.D. | 0.14 | ± | 0.02 |
| Mannitol | 0.33 | 0.34 | 0.36 | 0.35 | 0.38 | N.A. | N.D. | N.D. | N.D. | N.D. | N.D. | N.D. | N.D. | 0.35 | ± | 0.02 |
|  |  |  |  |  |  |  |  |  |  |  |  |  |  |  |  |  |
| Mannitol concentration was determined enzymatically (Sigma-Aldrich, kit MAK096-1KT) | | | | | | | |  |  |  |  |  |  |  |  |  |
| N.D. = not determined | |  |  |  |  |  |  |  |  |  |  |  |  |  |  |  |
| N.A. = not applicable | |  |  |  |  |  |  |  |  |  |  |  |  |  |  |  |
| <LOD = below detection limit | | |  |  |  |  |  |  |  |  |  |  |  |  |  |  |
| Substrate levels in lot. B and lot. C were measured enzymatically using the YSI Biochemistry analyzer | | | | | | | | |  |  |  |  |  |  |  |  |
