# Supplementary material for: Metabolic modeling of energy balances in Mycoplasma hyopneumoniae shows that pyruvate addition increases growth rate
Source: Biotechnol Bioeng. 2017 Jul 27;114(10):2339–47. doi: 10.1002/bit.26347 (PMC6084303; doi:10.1002/bit.26347)
Supplement: Supplementary file 4 — Table S3. Components consumed directly from the growth medium. [file BIT-114-2339-s004.docx]

| Table S3: Components consumed directly from the growth medium | |  |
| --- | --- | --- |
|  |  |  |
| **Component** | **Reaction** | **Flux glucose medium (mmol/gDW/h)** |
| Glycerol | 'EX_GLYCEROL_e' | -5.10E-001 |
| Serine | 'EX_SER_e' | -6.13E-003 |
| Glycerol-3-phosphate | 'EX_GLYCEROL3P_e' | -1.28E-001 |
| Glucose | 'EX_DGlucose_e' | -5.11E+000 |
| Alanine | 'EX_LALPHAALANINE_e' | -8.01E-003 |
| Arginine | 'EX_ARG_e' | -3.84E-003 |
| Asparagine | 'EX_ASN_e' | -5.77E-003 |
| Aspartate | 'EX_LASPARTATE_e' | -5.41E-003 |
| Cysteine | 'EX_CYS_e' | -8.65E-004 |
| Glutamate | 'EX_GLT_e' | -6.17E-003 |
| Glutamine | 'EX_GLN_e' | -5.53E-003 |
| Glycine | 'EX_GLY_e' | -6.26E-003 |
| Histidine | 'EX_HIS_e' | -1.79E-003 |
| Isoleucine | 'EX_ILE_e' | -6.39E-003 |
| Leucine | 'EX_LEU_e' | -8.87E-003 |
| Lysine | 'EX_LYS_e' | -9.28E-003 |
| Methionine | 'EX_MET_e' | -1.45E-003 |
| Phenylalanine | 'EX_PHE_e' | -4.45E-003 |
| Proline | 'EX_PRO_e' | -4.19E-003 |
| Threonine | 'EX_THR_e' | -6.11E-003 |
| Tryptophan | 'EX_TRP_e' | -9.07E-004 |
| Tyrosine | 'EX_TYR_e' | -2.91E-003 |
| Valine | 'EX_VAL_e' | -7.27E-003 |
| Guanine | 'EX_GUANINE_e' | -1.37E-003 |
| Adenine | 'EX_ADENINE_e' | -1.99E-003 |
| Thymine | 'EX_THYMINE_e' | -9.96E-004 |
| Deoxycytidine | 'EX_DEOXYCYTIDINE_e' | -1.88E-003 |
| Oxygen | 'EX_OXYGENMOLECULE__e' | -1.15E+001 |
| Long-chain Fatty acids | 'EX_LongChainFattyAcids_e' | -5.53E-003 |
| Phosphatidylcholine | 'EX_PHOSPHATIDYL_CHOLINE_e' | -1.66E-003 |
