# Supplementary material for: Metabolic modeling of energy balances in Mycoplasma hyopneumoniae shows that pyruvate addition increases growth rate
Source: Biotechnol Bioeng. 2017 Jul 27;114(10):2339–47. doi: 10.1002/bit.26347 (PMC6084303; doi:10.1002/bit.26347)
Supplement: Supplementary file 5 — Table S4. Calculation of energy distributions from model predicted reaction fluxes. [file BIT-114-2339-s005.docx]

| Table S4: calculation of energy distributions from model predicted reaction fluxes | | | |  |  |  |  |  |  |
| --- | --- | --- | --- | --- | --- | --- | --- | --- | --- |
|  |  |  |  |  |  |  |  |  |  |
| **Reaction** | **Glucose base** | **Glucose base ATP** | **Pyruvate early** | **Pyruvate early ATP** | **Pyruvate Mid** | **Pyruvate Mid ATP** | **Pyruvate late** | **Pyruvate late ATP** | **Reaction formula** |
| 'RXN01061' | 1.84E+001 | 1.84E+01 | 1.02E+01 | 1.02E+01 | 1.02E+01 | 1.02E+01 | 1.84E+01 | 1.84E+01 | 'WATER_c + ATP_c <=> ADP_c + PROTON_c + Pi_c ' |
| 'A3__46__6__46__5__46__3RXN' | 0.00E+000 |  | 0.00E+00 |  | 0.00E+00 |  | 0.00E+00 |  | 'WATER_c + GTP_c -> PROTON_c + Pi_c + GDP_c ' |
| 'RXN18KM18' | 0.00E+000 | 0.00E+00 | 0.00E+00 | 0.00E+00 | 0.00E+00 | 0.00E+00 | 0.00E+00 | 0.00E+00 | 'WATER_c + PRPP_c + ATP_c + NIACINE_c -> ADP_c + Pi_c + PPI_c + NICOTINATE_NUCLEOTIDE_c ' |
| 'URACILPRIBOSYLTRANSRXN' | -1.48E-003 |  | -2.12E-03 |  | -2.41E-02 |  | -3.31E-02 |  | 'UMP_c + PPI_c <=> PRPP_c + URACIL_c ' |
| 'TRANSRXN168' | 0.00E+000 |  | 0.00E+00 |  | 0.00E+00 |  | 0.00E+00 |  | 'PHOSPHOENOLPYRUVATE_c + MANNOSE_e -> MANNOSE6P_c + PYRUVATE_c ' |
| 'DCTPPYROPHOSPHATASERXN' | 0.00E+000 |  | 0.00E+00 |  | 0.00E+00 |  | 0.00E+00 |  | 'WATER_c + DCTP_c -> PROTON_c + DCMP_c + PPI_c ' |
| 'RXN2902' | 0.00E+000 |  | 0.00E+00 |  | 0.00E+00 |  | 0.00E+00 |  | 'COA_c + NAD_c + MALONATESALD_c -> ACETYLCOA_c + NADH_c + CARBONDIOXIDE_c ' |
| 'DUTPPYROPRXN' | 0.00E+000 |  | 0.00E+00 |  | 0.00E+00 |  | 0.00E+00 |  | 'WATER_c + DUTP_c -> PROTON_c + PPI_c + DUMP_c ' |
| 'A4__46__1__46__2__46__29RXN' | 0.00E+000 |  | 0.00E+00 |  | 0.00E+00 |  | 0.00E+00 |  | 'CPD645_c -> DIHYDROXYACETONEPHOSPHATE_c + MALONATESALD_c ' |
| 'RXN18KM8' | 4.03E-004 |  | 5.76E-04 |  | 6.57E-03 |  | 9.01E-03 |  | 'DCDP_c + PHOSPHOENOLPYRUVATE_c + PROTON_c -> DCTP_c + PYRUVATE_c ' |
| 'MYOINOSITOL2DEHYDROGENASERXN' | 0.00E+000 |  | 0.00E+00 |  | 0.00E+00 |  | 0.00E+00 |  | 'MYOINOSITOL_c + NAD_c -> PROTON_c + CPD365_c + NADH_c ' |
| 'RXN05292' | 0.00E+000 |  | 0.00E+00 |  | 0.00E+00 |  | 0.00E+00 |  | 'WATER_c + DCMP_c -> DEOXYCYTIDINE_c + Pi_c ' |
| 'A2__46__7__46__7__46__15RXN' | 0.00E+000 |  | 0.00E+00 |  | 0.00E+00 |  | 0.00E+00 |  | 'CTP_c + PHOSPHORYLCHOLINE_c + PROTON_c -> CDPCHOLINE_c + PPI_c ' |
| 'CDPDIGLYSYNRXN' | 2.62E-003 |  | 3.75E-03 |  | 4.27E-02 |  | 5.86E-02 |  | 'CTP_c + LPHOSPHATIDATE_c + PROTON_c -> CDPDIACYLGLYCEROL_c + PPI_c ' |
| 'RXN1381' | 2.77E-003 |  | 3.96E-03 |  | 4.51E-02 |  | 6.19E-02 |  | 'GLYCEROL3P_c + LongChainAcylCoAs_c -> ACYLSNGLYCEROL3P_c + COA_c ' |
| 'RXN05375' | 0.00E+000 |  | 0.00E+00 |  | 0.00E+00 |  | 0.00E+00 |  | '2 PROTON_c + CPD01147_c + 2 NADH_c <=> ANTHRANILATE_c + CPD01148_c + 2 NAD_c ' |
| 'NACETYLGLUCOSAMINEKINASERXN' | 0.00E+000 | 0.00E+00 | 0.00E+00 | 0.00E+00 | 0.00E+00 | 0.00E+00 | 0.00E+00 | 0.00E+00 | 'ATP_c + NACETYLDGLUCOSAMINE_c -> ADP_c + PROTON_c + NACETYLDGLUCOSAMINE6P_c ' |
| 'GTPCYCLOHYDROIRXN' | 0.00E+000 |  | 0.00E+00 |  | 0.00E+00 |  | 0.00E+00 |  | 'WATER_c + GTP_c <=> PROTON_c + DIHYDRONEOPTERINP3_c + FORMATE_c ' |
| 'CHOLINEKINASERXN' | 0.00E+000 | 0.00E+00 | 0.00E+00 | 0.00E+00 | 0.00E+00 | 0.00E+00 | 0.00E+00 | 0.00E+00 | 'ATP_c + CHOLINE_c -> ADP_c + PHOSPHORYLCHOLINE_c + PROTON_c ' |
| 'ATPSYNRXN' | -1.11E-001 | -1.11E-01 | -2.17E+00 | -2.17E+00 | -2.94E+00 | -2.94E+00 | -3.24E+00 | -3.24E+00 | 'WATER_c + 3 PROTON_c + ATP_c <=> 4 PROTON_e + ADP_c + Pi_c ' |
| 'RXN3523' | 0.00E+000 |  | 0.00E+00 |  | 0.00E+00 |  | 0.00E+00 |  | '2 CPD318_c -> PROTON_c + ASCORBATE_c + LDEHYDROASCORBATE_c ' |
| 'THYMIDYLATE5PHOSPHATASERXN' | 0.00E+000 |  | 0.00E+00 |  | 0.00E+00 |  | 0.00E+00 |  | 'WATER_c + TMP_c -> THYMIDINE_c + Pi_c ' |
| 'GAPOXNPHOSPHNRXN' | 1.08E+001 |  | 5.02E+00 |  | 1.05E+01 |  | 1.04E+01 |  | 'Pi_c + GAP_c + NAD_c <=> DPG_c + PROTON_c + NADH_c ' |
| 'R503RXN' | 0.00E+000 |  | 0.00E+00 |  | 0.00E+00 |  | 0.00E+00 |  | 'WATER_c + CPD15167_c -> PROTON_c + CPD827_c ' |
| 'GMKALTRXN' | 4.03E-004 | 4.03E-04 | 5.76E-04 | 5.76E-04 | 6.57E-03 | 6.57E-03 | 9.01E-03 | 9.01E-03 | 'ATP_c + DGMP_c -> ADP_c + DGDP_c ' |
| 'RXN05305' | 0.00E+000 |  | 0.00E+00 |  | 0.00E+00 |  | 0.00E+00 |  | 'CPD01108_c <=> RIBOSE_c ' |
| 'LACTOSE6PHOSPHATEISOMERASERXN' | 0.00E+000 |  | 0.00E+00 |  | 0.00E+00 |  | 0.00E+00 |  | 'CPD1241_c <=> TAGATOSE6PHOSPHATE_c ' |
| 'RXN18KM6' | 1.42E-001 |  | 1.43E-01 |  | 1.82E-01 |  | 1.98E-01 |  | 'PHOSPHOENOLPYRUVATE_c + PROTON_c + CDP_c -> CTP_c + PYRUVATE_c ' |
| 'GLUCOSAMINE6PDEAMINRXN' | 0.00E+000 |  | 0.00E+00 |  | 0.00E+00 |  | 0.00E+00 |  | 'WATER_c + DGLUCOSAMINE6P_c -> AMMONIA_c + PROTON_c + FRUCTOSE6P_c ' |
| 'RIBULP3EPIMRXN' | -2.90E-003 |  | -4.15E-03 |  | -4.74E-02 |  | -6.50E-02 |  | 'RIBULOSE5P_c <=> XYLULOSE5PHOSPHATE_c ' |
| 'DEOXYADENYLATEKINASERXN' | 8.04E-005 | 8.04E-05 | 1.15E-04 | 1.15E-04 | 1.31E-03 | 1.31E-03 | 1.80E-03 | 1.80E-03 | 'ATP_c + DAMP_c -> ADP_c + DADP_c ' |
| 'RXN11811' | 0.00E+000 |  | 0.00E+00 |  | 0.00E+00 |  | 0.00E+00 |  | 'AMMONIA_c + PROTON_c <=> AMMONIUM_c ' |
| 'RXN05199' | 0.00E+000 |  | 0.00E+00 |  | 0.00E+00 |  | 0.00E+00 |  | 'Pi_c + GUANOSINE_c <=> RIBOSE1P_c + GUANINE_c ' |
| 'RXN10981' | 0.00E+000 |  | 0.00E+00 |  | 0.00E+00 |  | 0.00E+00 |  | 'Acceptor_c + 2 PROTON_c + ASCORBATE_c -> CPD318_c + DonorH2_c ' |
| 'RXN12149' | 0.00E+000 |  | 0.00E+00 |  | 0.00E+00 |  | 0.00E+00 |  | 'Lrhamnose_c <=> LRHAMNOFURANOSE_c ' |
| 'A1TRANSKETORXN' | 1.45E-003 |  | 2.08E-03 |  | 2.37E-02 |  | 3.25E-02 |  | 'GAP_c + DSEDOHEPTULOSE7P_c <=> XYLULOSE5PHOSPHATE_c + RIBOSE5P_c ' |
| 'PGPPHOSPHARXN' | 1.31E-003 |  | 1.87E-03 |  | 2.14E-02 |  | 2.93E-02 |  | 'WATER_c + L1PHOSPHATIDYLGLYCEROLP_c -> L1PHOSPHATIDYLGLYCEROL_c + Pi_c ' |
| 'A2TRANSKETORXN' | -1.45E-003 |  | -2.08E-03 |  | -2.37E-02 |  | -3.25E-02 |  | 'XYLULOSE5PHOSPHATE_c + ERYTHROSE4P_c <=> GAP_c + FRUCTOSE6P_c ' |
| 'RXN12440' | 0.00E+000 |  | 0.00E+00 |  | 0.00E+00 |  | 0.00E+00 |  | 'HYDROGENPEROXIDE_c + PROTON_c -> 2 WATER_c + ASCORBATE_c + LDEHYDROASCORBATE_c ' |
| 'RXN1623' | 2.77E-003 |  | 3.96E-03 |  | 4.51E-02 |  | 6.19E-02 |  | 'ACYLSNGLYCEROL3P_c + LongChainAcylCoAs_c -> LPHOSPHATIDATE_c + COA_c ' |
| 'RXN12754' | 0.00E+000 |  | 0.00E+00 |  | 0.00E+00 |  | 0.00E+00 |  | 'WATER_c + NADH_c -> CPD02472_c ' |
| 'DEOXYGUANPHOSPHORRXN' | -4.03E-004 |  | -5.76E-04 |  | -6.57E-03 |  | -9.01E-03 |  | 'Pi_c + DEOXYGUANOSINE_c <=> DEOXYDRIBOSE1PHOSPHATE_c + GUANINE_c ' |
| 'DEOXYCYTIDINEKINASERXN' | 4.03E-004 | 4.03E-04 | 5.76E-04 | 5.76E-04 | 6.57E-03 | 6.57E-03 | 9.01E-03 | 9.01E-03 | 'DEOXYCYTIDINE_c + ATP_c -> ADP_c + PROTON_c + DCMP_c ' |
| 'RXN12753' | 0.00E+000 |  | 0.00E+00 |  | 0.00E+00 |  | 0.00E+00 |  | 'WATER_c + NADH_c -> CPD653_c ' |
| 'ADENPHOSPHORRXN' | 0.00E+000 |  | 0.00E+00 |  | 0.00E+00 |  | 0.00E+00 |  | 'ADENOSINE_c + Pi_c <=> RIBOSE1P_c + ADENINE_c ' |
| 'RXN12862' | 0.00E+000 |  | 0.00E+00 |  | 0.00E+00 |  | 0.00E+00 |  | 'WATER_c + LDEHYDROASCORBATE_c -> CPD13907_c ' |
| 'RXN8141' | 1.31E-003 |  | 1.87E-03 |  | 2.14E-02 |  | 2.93E-02 |  | 'L1PHOSPHATIDYLGLYCEROL_c + CDPDIACYLGLYCEROL_c <=> CMP_c + CARDIOLIPIN_c + PROTON_c ' |
| 'PANTEPADENYLYLTRANRXN' | 0.00E+000 | 0.00E+00 | 0.00E+00 | 0.00E+00 | 0.00E+00 | 0.00E+00 | 0.00E+00 | 0.00E+00 | 'PROTON_c + ATP_c + PANTETHEINEP_c -> DEPHOSPHOCOA_c + PPI_c ' |
| 'TRANSRXN104' | 0.00E+000 |  | -6.50E+00 |  | -6.50E+00 |  | 6.41E+00 |  | 'PROTON_e + LLACTATE_e -> PROTON_c + LLACTATE_c ' |
| 'RXN18KM2' | 0.00E+000 |  | 0.00E+00 |  | 0.00E+00 |  | 0.00E+00 |  | 'FRU_e + PHOSPHOENOLPYRUVATE_c -> FRU1P_c + PYRUVATE_c ' |
| 'RXN0705' | 0.00E+000 |  | 0.00E+00 |  | 0.00E+00 |  | 0.00E+00 |  | 'PROTON_c + CPD2343_c -> CARBONDIOXIDE_c + LXYLULOSE5P_c ' |
| 'NADHDEHYDROGENASERXN' | 1.08E+001 |  | 2.60E+00 |  | 8.04E+00 |  | 2.09E+01 |  | '2 PROTON_c + 2 NADH_c + OXYGENMOLECULE_c <=> 2 WATER_c + 2 NAD_c ' |
| 'RIBULPEPIMRXN' | 0.00E+000 |  | 0.00E+00 |  | 0.00E+00 |  | 0.00E+00 |  | 'LRIBULOSE5P_c <=> XYLULOSE5PHOSPHATE_c ' |
| 'A1__46__2__46__1__46__27RXN' | 0.00E+000 |  | 0.00E+00 |  | 0.00E+00 |  | 0.00E+00 |  | 'WATER_c + COA_c + CPD12179_c + NAD_c <=> PROTON_c + PROPIONYLCOA_c + NADH_c + HCO3_c ' |
| 'PHOSPHOGLYCERATEKINASEGTPRXN' | -2.18E-001 |  | -2.18E-01 |  | 4.71E+00 |  | 4.60E+00 |  | 'GTP_c + G3P_c <=> DPG_c + GDP_c ' |
| 'RXN12863' | 0.00E+000 |  | 0.00E+00 |  | 0.00E+00 |  | 0.00E+00 |  | 'HYDROGENPEROXIDE_c + CPD13907_c -> 3 PROTON_c + OXALATE_c + LTHREONATE_c ' |
| 'RXN12869' | 0.00E+000 |  | 0.00E+00 |  | 0.00E+00 |  | 0.00E+00 |  | 'CPD13907_c -> 3 PROTON_c + CPD13914_c ' |
| 'RXN18KM13' | -9.96E-004 |  | -1.42E-03 |  | -1.62E-02 |  | -2.23E-02 |  | 'DATP_c + G3P_c <=> DPG_c + DADP_c ' |
| 'RXN12861' | 0.00E+000 |  | 0.00E+00 |  | 0.00E+00 |  | 0.00E+00 |  | 'CPD13907_c -> CPD334_c + PROTON_c ' |
| 'RXN14143' | 0.00E+000 |  | 0.00E+00 |  | 0.00E+00 |  | 0.00E+00 |  | 'WATER_c + DUMP_c -> Pi_c + DEOXYURIDINE_c ' |
| 'A5__46__3__46__1__46__17RXN' | 0.00E+000 |  | 0.00E+00 |  | 0.00E+00 |  | 0.00E+00 |  | 'CPD37_c <=> CPD343_c ' |
| 'RXN8654' | 0.00E+000 | 0.00E+00 | 0.00E+00 | 0.00E+00 | 0.00E+00 | 0.00E+00 | 0.00E+00 | 0.00E+00 | 'PROTON_c + ATP_c + LIPOICACID_c -> LIPOYLAMP_c + PPI_c ' |
| 'RXN02461' | 0.00E+000 |  | 0.00E+00 |  | 0.00E+00 |  | 0.00E+00 |  | 'PHOSPHOENOLPYRUVATE_c + ASCORBATE_e -> LASCORBATE6PHOSPHATE_c + PYRUVATE_c ' |
| 'RIBOFLAVINKINRXN' | 0.00E+000 | 0.00E+00 | 0.00E+00 | 0.00E+00 | 0.00E+00 | 0.00E+00 | 0.00E+00 | 0.00E+00 | 'RIBOFLAVIN_c + ATP_c -> FMN_c + ADP_c + PROTON_c ' |
| 'ETHANOLAMINEKINASERXN' | 0.00E+000 | 0.00E+00 | 0.00E+00 | 0.00E+00 | 0.00E+00 | 0.00E+00 | 0.00E+00 | 0.00E+00 | 'ATP_c + ETHANOLAMINE_c <=> PHOSPHORYLETHANOLAMINE_c + ADP_c + PROTON_c ' |
| 'AMPDEPHOSPHORYLATIONRXN' | 0.00E+000 |  | 0.00E+00 |  | 0.00E+00 |  | 0.00E+00 |  | 'WATER_c + AMP_c -> ADENOSINE_c + Pi_c ' |
| 'RXN14142' | 0.00E+000 |  | 0.00E+00 |  | 0.00E+00 |  | 0.00E+00 |  | 'WATER_c + DGMP_c -> Pi_c + DEOXYGUANOSINE_c ' |
| 'RXN7609' | 0.00E+000 |  | 0.00E+00 |  | 0.00E+00 |  | 0.00E+00 |  | 'WATER_c + GMP_c -> Pi_c + GUANOSINE_c ' |
| 'RXN12872' | 0.00E+000 |  | 0.00E+00 |  | 0.00E+00 |  | 0.00E+00 |  | 'CPD13914_c -> CPD13910_c ' |
| 'RXN14025' | 0.00E+000 |  | 0.00E+00 |  | 0.00E+00 |  | 0.00E+00 |  | 'WATER_c + UMP_c -> URIDINE_c + Pi_c ' |
| 'RXN14026' | 0.00E+000 |  | 0.00E+00 |  | 0.00E+00 |  | 0.00E+00 |  | 'WATER_c + CMP_c -> Pi_c + CYTIDINE_c ' |
| 'RXN05214' | 0.00E+000 |  | 0.00E+00 |  | 0.00E+00 |  | 0.00E+00 |  | 'WATER_c + LASCORBATE6PHOSPHATE_c <=> CPD2343_c ' |
| 'RXN12870' | 0.00E+000 |  | 0.00E+00 |  | 0.00E+00 |  | 0.00E+00 |  | 'CPD334_c -> CPD13913_c ' |
| 'RXN14150' | 0.00E+000 |  | 0.00E+00 |  | 0.00E+00 |  | 0.00E+00 |  | 'CPD827_c <=> CPD15127_c ' |
| 'GUANPRIBOSYLTRANRXN' | -9.64E-004 |  | -1.38E-03 |  | -1.57E-02 |  | -2.16E-02 |  | 'PPI_c + GMP_c <=> PRPP_c + GUANINE_c ' |
| 'RXN18KM10' | 9.96E-004 |  | 1.42E-03 |  | 1.62E-02 |  | 2.23E-02 |  | 'PHOSPHOENOLPYRUVATE_c + PROTON_c + TDP_c -> TTP_c + PYRUVATE_c ' |
| 'RXN12871' | 0.00E+000 |  | 0.00E+00 |  | 0.00E+00 |  | 0.00E+00 |  | 'WATER_c + CPD13913_c -> PROTON_c + CPD13912_c ' |
| 'RXN18KM15' | 5.40E+000 |  | 2.38E+00 |  | -6.57E-03 |  | -9.01E-03 |  | 'DGTP_c + G3P_c <=> DPG_c + DGDP_c ' |
| 'TRANSALDOLRXN' | -1.45E-003 |  | -2.08E-03 |  | -2.37E-02 |  | -3.25E-02 |  | 'GAP_c + DSEDOHEPTULOSE7P_c <=> ERYTHROSE4P_c + FRUCTOSE6P_c ' |
| 'A3__46__1__46__4__46__2RXN' | 0.00E+000 |  | 0.00E+00 |  | 0.00E+00 |  | 0.00E+00 |  | WATER_c + L1GLYCEROPHOSPHORYLCHOLINE_c -> GLYCEROL3P_c + PROTON_c + CHOLINE_c ' |
| 'RIB5PISOMRXN' | -2.90E-003 |  | -4.15E-03 |  | -4.74E-02 |  | -6.50E-02 |  | 'RIBOSE5P_c <=> RIBULOSE5P_c ' |
| 'RXN18KM7' | 1.95E-001 |  | 1.96E-01 |  | 2.18E-01 |  | 2.27E-01 |  | 'UDP_c + PHOSPHOENOLPYRUVATE_c + PROTON_c -> PYRUVATE_c + UTP_c ' |
| 'A3__46__6__46__3__46__20RXN' | 1.28E-001 | 1.28E-01 | 1.28E-01 | 1.28E-01 | 1.28E-01 | 1.28E-01 | 1.28E-01 | 1.28E-01 | 'WATER_c + ATP_c + GLYCEROL3P_e <=> GLYCEROL3P_c + ADP_c + PROTON_c + Pi_c ' |
| 'DEOXYADENPHOSPHORRXN' | -8.04E-005 |  | -1.15E-04 |  | -1.31E-03 |  | -1.80E-03 |  | 'Pi_c + DEOXYADENOSINE_c <=> DEOXYDRIBOSE1PHOSPHATE_c + ADENINE_c ' |
| 'MANNPDEHYDROGRXN' | 0.00E+000 |  | 0.00E+00 |  | 0.00E+00 |  | 0.00E+00 |  | 'MANNITOL1P_c + NAD_c <=> PROTON_c + NADH_c + FRUCTOSE6P_c ' |
| 'TRANSRXN156' | 0.00E+000 |  | 0.00E+00 |  | 0.00E+00 |  | 0.00E+00 |  | 'PHOSPHOENOLPYRUVATE_c + MANNITOL_e -> MANNITOL1P_c + PYRUVATE_c ' |
| 'PHOSACETYLTRANSRXN' | 1.08E+001 |  | 6.69E+00 |  | 1.21E+01 |  | 2.49E+01 |  | 'ACETYLCOA_c + Pi_c <=> COA_c + ACETYLP_c ' |
| 'ACETATEKINRXN' | -1.08E+001 | -1.08E+01 | -6.69E+00 | -6.69E+00 | -1.21E+01 | -1.21E+01 | -2.49E+01 | -2.49E+01 | 'ACET_c + ATP_c <=> ADP_c + ACETYLP_c ' |
| 'A3__46__6__46__3__46__17RXN' | 0.00E+000 | 0.00E+00 | 0.00E+00 | 0.00E+00 | 0.00E+00 | 0.00E+00 | 0.00E+00 | 0.00E+00 | 'WATER_c + RIBOSE_e + ATP_c <=> RIBOSE_c + ADP_c + PROTON_c + Pi_c ' |
| 'NICONUCADENYLYLTRANRXN' | 0.00E+000 | 0.00E+00 | 0.00E+00 | 0.00E+00 | 0.00E+00 | 0.00E+00 | 0.00E+00 | 0.00E+00 | 'PROTON_c + ATP_c + NICOTINATE_NUCLEOTIDE_c <=> PPI_c + DEAMIDONAD_c ' |
| 'TRANSRXN131' | 5.10E-001 |  | 3.10E-01 |  | 3.10E-01 |  | 3.10E-01 |  | 'GLYCEROL_e -> GLYCEROL_c ' |
| 'LXULRU5PRXN' | 0.00E+000 |  | 0.00E+00 |  | 0.00E+00 |  | 0.00E+00 |  | 'LRIBULOSE5P_c <=> LXYLULOSE5P_c ' |
| 'GLYCEROL3PHOSPHATEOXIDASERXN' | 6.34E-001 |  | 4.31E-01 |  | 3.71E-01 |  | 3.46E-01 |  | 'GLYCEROL3P_c + OXYGENMOLECULE_c <=> HYDROGENPEROXIDE_c + DIHYDROXYACETONEPHOSPHATE_c ' |
| 'RXN3715' | 5.11E+000 |  | 2.30E+00 |  | 5.11E+00 |  | 5.11E+00 |  | 'PHOSPHOENOLPYRUVATE_c + DGlucose_e -> Dglucose6phosphate_c + PYRUVATE_c ' |
| 'RXN11832' | 1.42E-001 | 1.42E-01 | 1.43E-01 | 1.43E-01 | 1.82E-01 | 1.82E-01 | 1.98E-01 | 1.98E-01 | 'CMP_c + ATP_c <=> ADP_c + CDP_c ' |
| 'RXN7913' | 4.03E-004 | 4.03E-04 | 5.76E-04 | 5.76E-04 | 6.57E-03 | 6.57E-03 | 9.01E-03 | 9.01E-03 | 'ATP_c + DCMP_c <=> DCDP_c + ADP_c ' |
| 'RXN8631' | 0.00E+000 |  | 0.00E+00 |  | 0.00E+00 |  | 0.00E+00 |  | 'FRU1P_c -> DIHYDROXYACETONEPHOSPHATE_c + GLYCERALD_c ' |
| 'F16ALDOLASERXN' | 5.11E+000 |  | 2.30E+00 |  | 5.06E+00 |  | 5.04E+00 |  | 'FRUCTOSE16DIPHOSPHATE_c <=> DIHYDROXYACETONEPHOSPHATE_c + GAP_c ' |
| 'PRPPSYNRXN' | 4.36E-003 | 4.36E-03 | 6.23E-03 | 6.23E-03 | 7.10E-02 | 7.10E-02 | 9.75E-02 | 9.75E-02 | 'ATP_c + RIBOSE5P_c -> PRPP_c + PROTON_c + AMP_c ' |
| 'DURIDKIRXN' | 0.00E+000 | 0.00E+00 | 0.00E+00 | 0.00E+00 | 0.00E+00 | 0.00E+00 | 0.00E+00 | 0.00E+00 | 'ATP_c + DEOXYURIDINE_c -> ADP_c + PROTON_c + DUMP_c ' |
| 'THYKIRXN' | 9.96E-004 | 9.96E-04 | 1.42E-03 | 1.42E-03 | 1.62E-02 | 1.62E-02 | 2.23E-02 | 2.23E-02 | 'ATP_c + THYMIDINE_c -> ADP_c + PROTON_c + TMP_c ' |
| 'INORGPYROPHOSPHATRXN' | 9.99E-001 |  | 1.05E+00 |  | 2.72E+00 |  | 3.40E+00 |  | 'WATER_c + PPI_c -> PROTON_c + 2 Pi_c ' |
| 'A3PGAREARRRXN' | 1.08E+001 |  | 5.02E+00 |  | 1.05E+01 |  | 1.04E+01 |  | 'G3P_c <=> A2PG_c ' |
| 'NAG6PDEACETRXN' | 0.00E+000 |  | 0.00E+00 |  | 0.00E+00 |  | 0.00E+00 |  | 'WATER_c + NACETYLDGLUCOSAMINE6P_c -> ACET_c + DGLUCOSAMINE6P_c ' |
| 'DEPHOSPHOCOAKINRXN' | 0.00E+000 | 0.00E+00 | 0.00E+00 | 0.00E+00 | 0.00E+00 | 0.00E+00 | 0.00E+00 | 0.00E+00 | 'ATP_c + DEPHOSPHOCOA_c -> ADP_c + PROTON_c + COA_c ' |
| 'RXN13720' | 5.11E+000 |  | 2.30E+00 |  | 5.11E+00 |  | 5.11E+00 |  | 'Dglucose6phosphate_c <=> FRUCTOSE6P_c ' |
| 'DEOXYRIBOSEPALDRXN' | 0.00E+000 |  | 0.00E+00 |  | 0.00E+00 |  | 0.00E+00 |  | 'DEOXYRIBOSE5P_c -> ACETALD_c + GAP_c ' |
| 'URAPHOSPHRXN' | 1.48E-003 |  | 2.12E-03 |  | 2.41E-02 |  | 3.31E-02 |  | 'Pi_c + DEOXYURIDINE_c <=> URACIL_c + DEOXYDRIBOSE1PHOSPHATE_c ' |
| 'URPHOSRXN' | 0.00E+000 |  | 0.00E+00 |  | 0.00E+00 |  | 0.00E+00 |  | 'URIDINE_c + Pi_c <=> URACIL_c + RIBOSE1P_c ' |
| 'THYMPHOSPHRXN' | -9.96E-004 |  | -1.42E-03 |  | -1.62E-02 |  | -2.23E-02 |  | 'THYMIDINE_c + Pi_c <=> THYMINE_c + DEOXYDRIBOSE1PHOSPHATE_c ' |
| 'TRIOSEPISOMERIZATIONRXN' | -5.74E+000 |  | -2.73E+00 |  | -5.43E+00 |  | -5.39E+00 |  | 'GAP_c <=> DIHYDROXYACETONEPHOSPHATE_c ' |
| 'A6PFRUCTPHOSRXN' | 5.11E+000 | 5.11E+00 | 2.30E+00 | 2.30E+00 | 5.06E+00 | 5.06E+00 | 5.04E+00 | 5.04E+00 | 'ATP_c + FRUCTOSE6P_c -> ADP_c + PROTON_c + FRUCTOSE16DIPHOSPHATE_c ' |
| 'ADENPRIBOSYLTRANRXN' | -1.91E-003 |  | -2.74E-03 |  | -3.12E-02 |  | -4.28E-02 |  | 'PPI_c + AMP_c <=> PRPP_c + ADENINE_c ' |
| 'PEPDEPHOSRXN' | 0.00E+000 | 0.00E+00 | 0.00E+00 | 0.00E+00 | 0.00E+00 | 0.00E+00 | 0.00E+00 | 0.00E+00 | 'ADP_c + PHOSPHOENOLPYRUVATE_c + PROTON_c -> ATP_c + PYRUVATE_c ' |
| 'RXN14207' | 5.40E+000 |  | 2.38E+00 |  | 0.00E+00 |  | 0.00E+00 |  | 'PHOSPHOENOLPYRUVATE_c + PROTON_c + DGDP_c -> DGTP_c + PYRUVATE_c ' |
| 'RXN14192' | 0.00E+000 |  | 0.00E+00 |  | 0.00E+00 |  | 0.00E+00 |  | 'PHOSPHOENOLPYRUVATE_c + PROTON_c + DADP_c -> DATP_c + PYRUVATE_c ' |
| 'RXN14117' | 0.00E+000 |  | 0.00E+00 |  | 4.94E+00 |  | 4.84E+00 |  | 'PHOSPHOENOLPYRUVATE_c + PROTON_c + GDP_c -> GTP_c + PYRUVATE_c ' |
| 'LLACTATEDEHYDROGENASERXN' | 0.00E+000 |  | -6.50E+00 |  | -6.50E+00 |  | 6.41E+00 |  | 'LLACTATE_c + NAD_c <=> PROTON_c + NADH_c + PYRUVATE_c ' |
| 'GUANYLKINRXN' | 2.18E-001 | 2.18E-01 | 2.18E-01 | 2.18E-01 | 2.32E-01 | 2.32E-01 | 2.38E-01 | 2.38E-01 | 'ATP_c + GMP_c -> ADP_c + GDP_c ' |
| 'CYTIDEAMRXN' | 1.48E-003 |  | 2.12E-03 |  | 2.41E-02 |  | 3.31E-02 |  | 'WATER_c + DEOXYCYTIDINE_c -> AMMONIA_c + DEOXYURIDINE_c ' |
| 'CYTIDEAM2RXN' | 0.00E+000 |  | 0.00E+00 |  | 0.00E+00 |  | 0.00E+00 |  | 'WATER_c + CYTIDINE_c -> URIDINE_c + AMMONIA_c ' |
| 'DPPENTOMUTRXN' | 0.00E+000 |  | 0.00E+00 |  | 0.00E+00 |  | 0.00E+00 |  | 'DEOXYDRIBOSE1PHOSPHATE_c <=> DEOXYRIBOSE5P_c ' |
| 'PPENTOMUTRXN' | 0.00E+000 |  | 0.00E+00 |  | 0.00E+00 |  | 0.00E+00 |  | 'RIBOSE1P_c <=> RIBOSE5P_c ' |
| 'ADENYLKINRXN' | 4.43E-001 | 4.43E-01 | 4.88E-01 | 4.88E-01 | 2.05E+00 | 2.05E+00 | 2.69E+00 | 2.69E+00 | 'ATP_c + AMP_c -> 2 ADP_c ' |
| 'GLYOHMETRANSRXN' | 0.00E+000 |  | 0.00E+00 |  | 0.00E+00 |  | 0.00E+00 |  | 'SER_c + THF_c <=> WATER_c + GLY_c + METHYLENETHF_c ' |
| 'A5DEHYDRO2DEOXYGLUCONOKINASERXN' | 0.00E+000 | 0.00E+00 | 0.00E+00 | 0.00E+00 | 0.00E+00 | 0.00E+00 | 0.00E+00 | 0.00E+00 | 'ATP_c + CPD827_c -> ADP_c + PROTON_c + CPD645_c ' |
| 'MYOINOSOSE2DEHYDRATASERXN' | 0.00E+000 |  | 0.00E+00 |  | 0.00E+00 |  | 0.00E+00 |  | 'CPD365_c -> WATER_c + CPD15127_c ' |
| 'A2PGADEHYDRATRXN' | 1.08E+001 |  | 5.02E+00 |  | 1.05E+01 |  | 1.04E+01 |  | 'A2PG_c <=> WATER_c + PHOSPHOENOLPYRUVATE_c ' |
| 'DTMPKIRXN' | 9.96E-004 | 9.96E-04 | 1.42E-03 | 1.42E-03 | 1.62E-02 | 1.62E-02 | 2.23E-02 | 2.23E-02 | 'ATP_c + TMP_c <=> ADP_c + TDP_c ' |
| 'FADSYNRXN' | 0.00E+000 | 0.00E+00 | 0.00E+00 | 0.00E+00 | 0.00E+00 | 0.00E+00 | 0.00E+00 | 0.00E+00 | 'FMN_c + PROTON_c + ATP_c -> PPI_c + FAD_c ' |
| 'CTPSYNRXN' | 6.19E-004 | 6.19E-04 | 8.86E-04 | 8.86E-04 | 1.01E-02 | 1.01E-02 | 1.39E-02 | 1.39E-02 | 'WATER_c + GLN_c + ATP_c + UTP_c <=> CTP_c + GLT_c + ADP_c + 2 PROTON_c + Pi_c ' |
| 'PHOSPHAGLYPSYNRXN' | 1.31E-003 |  | 1.87E-03 |  | 2.14E-02 |  | 2.93E-02 |  | 'GLYCEROL3P_c + CDPDIACYLGLYCEROL_c -> CMP_c + L1PHOSPHATIDYLGLYCEROLP_c + PROTON_c ' |
| 'DIHYDLIPOXNRXN' | 0.00E+000 |  | 0.00E+00 |  | 0.00E+00 |  | 0.00E+00 |  | 'DIHYDROLIPOAMIDE_c + NAD_c <=> LIPOAMIDE_c + PROTON_c + NADH_c ' |
| 'MANNPISOMRXN' | 0.00E+000 |  | 0.00E+00 |  | 0.00E+00 |  | 0.00E+00 |  | 'MANNOSE6P_c <=> FRUCTOSE6P_c ' |
| 'PHOSGLYPHOSRXN' | -1.60E+001 | -1.60E+01 | -7.18E+00 | -7.18E+00 | -1.52E+01 | -1.52E+01 | -1.50E+01 | -1.50E+01 | 'ATP_c + G3P_c <=> DPG_c + ADP_c ' |
| 'NADSYNTHNH3RXN' | 0.00E+000 | 0.00E+00 | 0.00E+00 | 0.00E+00 | 0.00E+00 | 0.00E+00 | 0.00E+00 | 0.00E+00 | 'AMMONIA_c + ATP_c + DEAMIDONAD_c -> PPI_c + NAD_c + AMP_c ' |
| 'SADENMETSYNRXN' | 0.00E+000 | 0.00E+00 | 0.00E+00 | 0.00E+00 | 0.00E+00 | 0.00E+00 | 0.00E+00 | 0.00E+00 | 'WATER_c + ATP_c + MET_c -> Pi_c + PPI_c + SADENOSYLMETHIONINE_c ' |
| 'OROTPDECARBRXN' | 0.00E+000 |  | 0.00E+00 |  | 0.00E+00 |  | 0.00E+00 |  | 'OROTIDINE5PHOSPHATE_c + PROTON_c -> UMP_c + CARBONDIOXIDE_c ' |
| 'GLYCEROLKINRXN' | 5.10E-001 | 5.10E-01 | 3.10E-01 | 3.10E-01 | 3.10E-01 | 3.10E-01 | 3.10E-01 | 3.10E-01 | 'ATP_c + GLYCEROL_c -> GLYCEROL3P_c + ADP_c + PROTON_c ' |
| 'A6__46__3__46__5__46__7RXN' | 0.00E+000 | 0.00E+00 | 0.00E+00 | 0.00E+00 | 0.00E+00 | 0.00E+00 | 0.00E+00 | 0.00E+00 | 'WATER_c + GLN_c + LglutamyltRNAGln_c + ATP_c -> GLT_c + ADP_c + PROTON_c + ChargedGLNtRNAs_c + Pi_c ' |
| 'RXN12460' | -1.27E-002 |  | -1.52E-02 |  | -1.01E-01 |  | -1.36E-01 |  | 'WATER_c + ChargedASNtRNAs_c <=> 2 PROTON_c + ASNtRNAs_c + ASN_c ' |
| 'RXN9386' | 0.00E+000 | 0.00E+00 | 0.00E+00 | 0.00E+00 | 0.00E+00 | 0.00E+00 | 0.00E+00 | -1.36E-01 | 'GLNtRNAs_c + GLT_c + PROTON_c + ATP_c -> LglutamyltRNAGln_c + PPI_c + AMP_c ' |
| 'ASPARTATETRNALIGASERXN' | 1.15E-002 | 1.15E-02 | 1.37E-02 | 1.37E-02 | 9.10E-02 | 9.10E-02 | 1.22E-01 | 0.00E+00 | 'PROTON_c + ATP_c + LASPARTATE_c + ASPtRNAs_c -> PPI_c + AMP_c + ChargedASPtRNAs_c ' |
| 'GLUTAMINETRNALIGASERXN' | 1.08E-002 | 1.08E-02 | 1.29E-02 | 1.29E-02 | 8.58E-02 | 8.58E-02 | 1.16E-01 | 1.22E-01 | 'GLN_c + GLNtRNAs_c + PROTON_c + ATP_c -> ChargedGLNtRNAs_c + PPI_c + AMP_c ' |
| 'TYROSINETRNALIGASERXN' | 6.37E-003 | 6.37E-03 | 7.61E-03 | 7.61E-03 | 5.04E-02 | 5.04E-02 | 6.79E-02 | 1.16E-01 | 'TYR_c + PROTON_c + ATP_c + TYRtRNAs_c -> ChargedTYRtRNAs_c + PPI_c + AMP_c ' |
| 'GLYCINETRNALIGASERXN' | 1.34E-002 | 1.34E-02 | 1.60E-02 | 1.60E-02 | 1.06E-01 | 1.06E-01 | 1.43E-01 | 6.79E-02 | 'GLY_c + GLYtRNAs_c + PROTON_c + ATP_c -> ChargedGLYtRNAs_c + PPI_c + AMP_c ' |
| 'ISOLEUCINETRNALIGASERXN' | 1.40E-002 | 1.40E-02 | 1.68E-02 | 1.68E-02 | 1.11E-01 | 1.11E-01 | 1.50E-01 | 1.43E-01 | 'ILEtRNAs_c + PROTON_c + ATP_c + ILE_c -> ChargedILEtRNAs_c + PPI_c + AMP_c ' |
| 'ARGININETRNALIGASERXN' | 8.28E-003 | 8.28E-03 | 9.89E-03 | 9.89E-03 | 6.56E-02 | 6.56E-02 | 8.83E-02 | 1.50E-01 | 'ARGtRNAs_c + PROTON_c + ATP_c + ARG_c -> ChargedARGtRNAs_c + PPI_c + AMP_c ' |
| 'VALINETRNALIGASERXN' | 1.59E-002 | 1.59E-02 | 1.90E-02 | 1.90E-02 | 1.26E-01 | 1.26E-01 | 1.70E-01 | 8.83E-02 | 'VAL_c + VALtRNAs_c + PROTON_c + ATP_c -> ChargedVALtRNAs_c + PPI_c + AMP_c ' |
| 'LEUCINETRNALIGASERXN' | 1.85E-002 | 1.85E-02 | 2.21E-02 | 2.21E-02 | 1.47E-01 | 1.47E-01 | 1.97E-01 | 1.70E-01 | 'LEUtRNAs_c + PROTON_c + ATP_c + LEU_c -> ChargedLEUtRNAs_c + PPI_c + AMP_c ' |
| 'CYSTEINETRNALIGASERXN' | 1.91E-003 | 1.91E-03 | 2.28E-03 | 2.28E-03 | 1.51E-02 | 1.51E-02 | 2.04E-02 | 1.97E-01 | 'PROTON_c + ATP_c + CYS_c + CYStRNAs_c -> PPI_c + AMP_c + ChargedCYStRNAs_c ' |
| 'TRYPTOPHANTRNALIGASERXN' | 1.91E-003 | 1.91E-03 | 2.28E-03 | 2.28E-03 | 1.51E-02 | 1.51E-02 | 2.04E-02 | 2.04E-02 | 'PROTON_c + ATP_c + TRPtRNAs_c + TRP_c -> ChargedTRPtRNAs_c + PPI_c + AMP_c ' |
| 'THREONINETRNALIGASERXN' | 1.34E-002 | 1.34E-02 | 1.60E-02 | 1.60E-02 | 1.06E-01 | 1.06E-01 | 1.43E-01 | 2.04E-02 | 'THRtRNAs_c + PROTON_c + ATP_c + THR_c -> ChargedTHRtRNAs_c + PPI_c + AMP_c ' |
| 'GLURSRXN' | 1.40E-002 | 1.40E-02 | 1.68E-02 | 1.68E-02 | 1.11E-01 | 1.11E-01 | 1.50E-01 | 1.43E-01 | 'GLT_c + PROTON_c + ATP_c + GLTtRNAs_c -> ChargedGLTtRNAs_c + PPI_c + AMP_c ' |
| 'ALANINETRNALIGASERXN' | 1.72E-002 | 1.72E-02 | 2.05E-02 | 2.05E-02 | 1.36E-01 | 1.36E-01 | 1.83E-01 | 1.50E-01 | 'PROTON_c + ATP_c + LALPHAALANINE_c + ALAtRNAs_c -> ChargedALAtRNAs_c + PPI_c + AMP_c ' |
| 'LYSINETRNALIGASERXN' | 2.04E-002 | 2.04E-02 | 2.44E-02 | 2.44E-02 | 1.62E-01 | 1.62E-01 | 2.18E-01 | 1.83E-01 | 'PROTON_c + ATP_c + LYStRNAs_c + LYS_c -> ChargedLYStRNAs_c + PPI_c + AMP_c ' |
| 'HISTIDINETRNALIGASERXN' | 3.82E-003 | 3.82E-03 | 4.57E-03 | 4.57E-03 | 3.03E-02 | 3.03E-02 | 4.08E-02 | 2.18E-01 | 'HIS_c + PROTON_c + ATP_c + HIStRNAs_c -> ChargedHIStRNAs_c + PPI_c + AMP_c ' |
| 'SERINETRNALIGASERXN' | 1.34E-002 | 1.34E-02 | 1.60E-02 | 1.60E-02 | 1.06E-01 | 1.06E-01 | 1.43E-01 | 4.08E-02 | 'PROTON_c + SERtRNAs_c + ATP_c + SER_c -> ChargedSERtRNAs_c + PPI_c + AMP_c ' |
| 'PHENYLALANINETRNALIGASERXN' | 9.56E-003 | 9.56E-03 | 1.14E-02 | 1.14E-02 | 7.58E-02 | 7.58E-02 | 1.02E-01 | 1.43E-01 | 'PROTON_c + ATP_c + PHE_c + PHEtRNAs_c -> ChargedPHEtRNAs_c + PPI_c + AMP_c ' |
| 'ASPARAGINETRNALIGASERXN' | 0.00E+000 | 0.00E+00 | 0.00E+00 | 0.00E+00 | 0.00E+00 | 0.00E+00 | 0.00E+00 | 1.02E-01 | 'PROTON_c + ASNtRNAs_c + ATP_c + ASN_c -> PPI_c + AMP_c + ChargedASNtRNAs_c ' |
| 'METHIONINETRNALIGASERXN' | 3.19E-003 | 3.19E-03 | 3.81E-03 | 3.81E-03 | 2.53E-02 | 2.53E-02 | 3.41E-02 | 0.00E+00 | 'METtRNAs_c + PROTON_c + ATP_c + MET_c -> PPI_c + AMP_c + ChargedMETtRNAs_c ' |
| 'PROLINETRNALIGASERXN' | 8.92E-003 | 8.92E-03 | 1.07E-02 | 1.07E-02 | 7.06E-02 | 7.06E-02 | 9.51E-02 | 3.41E-02 | 'PRO_c + PROTON_c + ATP_c + PROtRNAs_c -> ChargedPROtRNAs_c + PPI_c + AMP_c ' |
| 'THIOREDOXINREDUCTNADPHRXN' | -9.16E-004 |  | -1.31E-03 |  | -1.49E-02 |  | -2.05E-02 |  | 'RedThioredoxin_c + NADP_c <=> PROTON_c + NADPH_c + OxThioredoxin_c ' |
| 'A3__46__1__46__4__46__14RXN' | 0.00E+000 |  | 0.00E+00 |  | 0.00E+00 |  | 0.00E+00 |  | 'WATER_c + ACP_c -> apoACP_c + PANTETHEINEP_c ' |
| 'RXN01134' | 1.08E+001 |  | 6.69E+00 |  | 1.21E+01 |  | 2.49E+01 |  | 'Pyruvatedehydrogenaselipoate_c + PROTON_c + PYRUVATE_c -> PyruvatedehydrogenaseacetylDHlipoyl_c + CARBONDIOXIDE_c ' |
| 'RXN01132' | 1.08E+001 |  | 6.69E+00 |  | 1.21E+01 |  | 2.49E+01 |  | 'Pyruvatedehydrogenasedihydrolipoate_c + NAD_c <=> Pyruvatedehydrogenaselipoate_c + PROTON_c + NADH_c ' |
| 'GDPREDUCTRXN' | 0.00E+000 |  | 0.00E+00 |  | 0.00E+00 |  | 0.00E+00 |  | 'RedThioredoxin_c + GDP_c -> WATER_c + DGDP_c + OxThioredoxin_c ' |
| 'HOLOACPSYNTHRXN' | 0.00E+000 |  | 0.00E+00 |  | 0.00E+00 |  | 0.00E+00 |  | 'COA_c + apoACP_c -> ACP_c + A35ADP_c ' |
| 'RXN01133' | -1.08E+001 |  | -6.69E+00 |  | -1.21E+01 |  | -2.49E+01 |  | 'Pyruvatedehydrogenasedihydrolipoate_c + ACETYLCOA_c <=> COA_c + PyruvatedehydrogenaseacetylDHlipoyl_c ' |
| 'CDPREDUCTRXN' | 0.00E+000 |  | 0.00E+00 |  | 0.00E+00 |  | 0.00E+00 |  | 'RedThioredoxin_c + CDP_c -> WATER_c + DCDP_c + OxThioredoxin_c ' |
| 'UDPREDUCTRXN' | 0.00E+000 |  | 0.00E+00 |  | 0.00E+00 |  | 0.00E+00 |  | 'UDP_c + RedThioredoxin_c -> WATER_c + DUDP_c + OxThioredoxin_c ' |
| 'ADPREDUCTRXN' | 9.16E-004 |  | 1.31E-03 |  | 1.49E-02 |  | 2.05E-02 |  | 'ADP_c + RedThioredoxin_c -> WATER_c + DADP_c + OxThioredoxin_c ' |
| 'RXN18KM3' | 0.00E+000 |  | 0.00E+00 |  | 0.00E+00 |  | 0.00E+00 |  | 'PHOSPHOENOLPYRUVATE_c + SER_e <=> PYRUVATE_c + A3PSERINE_c ' |
| 'TRANSRXN18KM6' | 0.00E+000 |  | 0.00E+00 |  | 0.00E+00 |  | 0.00E+00 |  | 'CPD4422_e -> CPD4422_c ' |
| 'carbonate_co2' | 0.00E+000 |  | 0.00E+00 |  | 0.00E+00 |  | 0.00E+00 |  | 'PROTON_c + HCO3_c <=> H2CO3_c ' |
| 'transport_alanine' | 8.01E-003 | 8.01E-03 | 1.15E-02 | 1.15E-02 | 1.31E-01 | 1.31E-01 | 1.79E-01 | 0.00E+00 | 'WATER_c + ATP_c + LALPHAALANINE_e -> ADP_c + PROTON_c + Pi_c + LALPHAALANINE_c ' |
| 'transport_arginine' | 3.84E-003 | 3.84E-03 | 5.49E-03 | 5.49E-03 | 6.26E-02 | 6.26E-02 | 8.59E-02 | 1.79E-01 | 'WATER_c + ATP_c + ARG_e -> ADP_c + PROTON_c + Pi_c + ARG_c ' |
| 'transport_L-asparagine' | 5.77E-003 | 5.77E-03 | 8.25E-03 | 8.25E-03 | 9.40E-02 | 9.40E-02 | 1.29E-01 | 8.59E-02 | 'WATER_c + ATP_c + ASN_e -> ADP_c + PROTON_c + ASN_c + Pi_c ' |
| 'transport_L-aspartate' | 5.41E-003 | 5.41E-03 | 7.74E-03 | 7.74E-03 | 8.82E-02 | 8.82E-02 | 1.21E-01 | 1.29E-01 | 'WATER_c + ATP_c + LASPARTATE_e -> ADP_c + PROTON_c + LASPARTATE_c + Pi_c ' |
| 'transport_L-cysteine' | 8.65E-004 | 8.65E-04 | 1.24E-03 | 1.24E-03 | 1.41E-02 | 1.41E-02 | 1.94E-02 | 1.21E-01 | 'WATER_c + ATP_c + CYS_e -> ADP_c + PROTON_c + CYS_c + Pi_c ' |
| 'transport_L-glutamate' | 6.17E-003 | 6.17E-03 | 8.83E-03 | 8.83E-03 | 1.01E-01 | 1.01E-01 | 1.38E-01 | 1.94E-02 | 'WATER_c + ATP_c + GLT_e -> GLT_c + ADP_c + PROTON_c + Pi_c ' |
| 'transport_L-glutamine' | 5.53E-003 | 5.53E-03 | 7.91E-03 | 7.91E-03 | 9.01E-02 | 9.01E-02 | 1.24E-01 | 1.38E-01 | 'WATER_c + ATP_c + GLN_e -> GLN_c + ADP_c + PROTON_c + Pi_c ' |
| 'transport_L-glycine' | 6.26E-003 | 6.26E-03 | 8.96E-03 | 8.96E-03 | 1.02E-01 | 1.02E-01 | 1.40E-01 | 1.24E-01 | 'WATER_c + ATP_c + GLY_e -> GLY_c + ADP_c + PROTON_c + Pi_c ' |
| 'transport_L-histidine' | 1.79E-003 | 1.79E-03 | 2.55E-03 | 2.55E-03 | 2.91E-02 | 2.91E-02 | 3.99E-02 | 1.40E-01 | 'WATER_c + ATP_c + HIS_e -> HIS_c + ADP_c + PROTON_c + Pi_c ' |
| 'transport_L-isoleucine' | 6.39E-003 | 6.39E-03 | 9.14E-03 | 9.14E-03 | 1.04E-01 | 1.04E-01 | 1.43E-01 | 3.99E-02 | 'WATER_c + ATP_c + ILE_e -> ADP_c + PROTON_c + Pi_c + ILE_c ' |
| 'transport_L-leucine' | 8.87E-003 | 8.87E-03 | 1.27E-02 | 1.27E-02 | 1.45E-01 | 1.45E-01 | 1.98E-01 | 1.43E-01 | 'WATER_c + ATP_c + LEU_e -> ADP_c + PROTON_c + Pi_c + LEU_c ' |
| 'transport_L-lysine' | 9.28E-003 | 9.28E-03 | 1.33E-02 | 1.33E-02 | 1.51E-01 | 1.51E-01 | 2.07E-01 | 1.98E-01 | 'WATER_c + ATP_c + LYS_e -> ADP_c + PROTON_c + Pi_c + LYS_c ' |
| 'transport_L-methionine' | 1.45E-003 | 1.45E-03 | 2.07E-03 | 2.07E-03 | 2.36E-02 | 2.36E-02 | 3.24E-02 | 2.07E-01 | 'WATER_c + ATP_c + MET_e -> ADP_c + PROTON_c + MET_c + Pi_c ' |
| 'transport_L-phenylalanine' | 4.45E-003 | 4.45E-03 | 6.36E-03 | 6.36E-03 | 7.25E-02 | 7.25E-02 | 9.95E-02 | 3.24E-02 | 'WATER_c + ATP_c + PHE_e -> ADP_c + PROTON_c + PHE_c + Pi_c ' |
| 'transport_L-proline' | 4.19E-003 | 4.19E-03 | 6.00E-03 | 6.00E-03 | 6.83E-02 | 6.83E-02 | 9.38E-02 | 9.95E-02 | 'WATER_c + ATP_c + PRO_e -> ADP_c + PRO_c + PROTON_c + Pi_c ' |
| 'transport_L-serine' | 6.13E-003 | 6.13E-03 | 8.77E-03 | 8.77E-03 | 9.99E-02 | 9.99E-02 | 1.37E-01 | 9.38E-02 | 'WATER_c + ATP_c + SER_e -> ADP_c + PROTON_c + SER_c + Pi_c ' |
| 'transport_L-threonine' | 6.11E-003 | 6.11E-03 | 8.73E-03 | 8.73E-03 | 9.95E-02 | 9.95E-02 | 1.37E-01 | 1.37E-01 | 'WATER_c + ATP_c + THR_e -> ADP_c + PROTON_c + THR_c + Pi_c ' |
| 'transport_L-tryptophan' | 9.07E-004 | 9.07E-04 | 1.30E-03 | 1.30E-03 | 1.48E-02 | 1.48E-02 | 2.03E-02 | 1.37E-01 | 'WATER_c + ATP_c + TRP_e -> ADP_c + PROTON_c + Pi_c + TRP_c ' |
| 'transport_L-tyrosine' | 2.91E-003 | 2.91E-03 | 4.16E-03 | 4.16E-03 | 4.75E-02 | 4.75E-02 | 6.51E-02 | 2.03E-02 | 'WATER_c + ATP_c + TYR_e -> ADP_c + TYR_c + PROTON_c + Pi_c ' |
| 'transport_L-valine' | 7.27E-003 | 7.27E-03 | 1.04E-02 | 1.04E-02 | 1.19E-01 | 1.19E-01 | 1.63E-01 | 6.51E-02 | 'WATER_c + ATP_c + VAL_e -> VAL_c + ADP_c + PROTON_c + Pi_c ' |
| 'transport_guanine' | 1.37E-003 |  | 1.95E-03 |  | 2.23E-02 |  | 3.06E-02 |  | 'PROTON_e + GUANINE_e <=> PROTON_c + GUANINE_c ' |
| 'transport_uracil' | 0.00E+000 |  | 0.00E+00 |  | 0.00E+00 |  | 0.00E+00 |  | 'PROTON_e + URACIL_e <=> URACIL_c + PROTON_c ' |
| 'transport_adenine' | 1.99E-003 |  | 2.85E-03 |  | 3.25E-02 |  | 4.46E-02 |  | 'PROTON_e + ADENINE_e <=> PROTON_c + ADENINE_c ' |
| 'transport_thymine' | 9.96E-004 |  | 1.42E-03 |  | 1.62E-02 |  | 2.23E-02 |  | 'PROTON_e + THYMINE_e <=> THYMINE_c + PROTON_c ' |
| 'transport_cytidine' | 0.00E+000 |  | 0.00E+00 |  | 0.00E+00 |  | 0.00E+00 |  | 'PROTON_e + CYTIDINE_e <=> PROTON_c + CYTIDINE_c ' |
| 'transport_deoxycytidine' | 1.88E-003 |  | 2.69E-03 |  | 3.07E-02 |  | 4.21E-02 |  | 'PROTON_e + DEOXYCYTIDINE_e <=> DEOXYCYTIDINE_c + PROTON_c ' |
| 'transport_phosphate_in' | 0.00E+000 | 0.00E+00 | 0.00E+00 | 0.00E+00 | 1.91E-02 | 1.91E-02 | 7.38E-02 | 4.21E-02 | 'WATER_c + ATP_c + Pi_e -> ADP_c + PROTON_c + 2 Pi_c ' |
| 'transport_acetate' | 1.08E+001 |  | 6.69E+00 |  | 1.21E+01 |  | 2.49E+01 |  | 'ACET_c + PROTON_c -> PROTON_e + ACET_e ' |
| 'transport_pyruvate' | 0.00E+000 |  | 8.16E+00 |  | 8.15E+00 |  | 8.13E+00 |  | 'PYRUVATE_e -> PYRUVATE_c ' |
| 'transport_CARBONDIOXIDE' | 1.08E+001 |  | 6.69E+00 |  | 1.21E+01 |  | 2.49E+01 |  | 'CARBONDIOXIDE_c <=> CARBONDIOXIDE__e ' |
| 'transport_WATER' | 1.11E+001 |  | -1.17E+00 |  | 7.62E+00 |  | 2.18E+01 |  | 'WATER_c <=> WATER_e ' |
| 'transport_OXYGENMOLECULE' | -1.15E+001 |  | -3.03E+00 |  | -8.41E+00 |  | -2.12E+01 |  | 'OXYGENMOLECULE_c <=> OXYGENMOLECULE__e ' |
| 'transport_AMMONIA' | 1.48E-003 |  | 2.12E-03 |  | 2.41E-02 |  | 3.31E-02 |  | 'AMMONIA_c <=> AMMONIA__e ' |
| 'transport_Riboflavin' | 0.00E+000 |  | 0.00E+00 |  | 0.00E+00 |  | 0.00E+00 |  | 'RIBOFLAVIN_e -> RIBOFLAVIN_c ' |
| 'transport_H2O2' | 6.34E-001 |  | 4.31E-01 |  | 3.71E-01 |  | 3.46E-01 |  | 'HYDROGENPEROXIDE_c -> HYDROGENPEROXIDE_e ' |
| 'transport_FA' | -5.53E-003 |  | -7.92E-03 |  | -9.02E-02 |  | -1.24E-01 |  | 'LongChainFattyAcids_c <=> LongChainFattyAcids_e ' |
| 'transport_panthetheine' | 0.00E+000 |  | 0.00E+00 |  | 0.00E+00 |  | 0.00E+00 |  | 'PANTETHEINEP_c <=> PANTETHEINEP_e ' |
| 'transport_PHOSPHATIDYL_CHOLINE' | -1.66E-003 |  | -2.37E-03 |  | -2.71E-02 |  | -3.71E-02 |  | 'PHOSPHATIDYL_CHOLINE_c <=> PHOSPHATIDYL_CHOLINE_e ' |
| 'transport_MYO_INOSITOL' | 0.00E+000 |  | 0.00E+00 |  | 0.00E+00 |  | 0.00E+00 |  | 'MYOINOSITOL_c <=> MYOINOSITOL_e ' |
| 'transport_phosphate_out' | 1.19E-001 |  | 1.15E-01 |  | 0.00E+00 |  | 0.00E+00 |  | 'Pi_c -> Pi_e ' |
| 'transport_niacine' | 0.00E+000 |  | 0.00E+00 |  | 0.00E+00 |  | 0.00E+00 |  | 'NIACINE_e -> NIACINE_c ' |
| 'EX_PROTON_e' | 1.04E+001 |  | 4.48E+00 |  | 6.77E+00 |  | 5.41E+00 |  | 'PROTON_e <=> ' |
| 'EX_RIBOSE_e' | 0.00E+000 |  | 0.00E+00 |  | 0.00E+00 |  | 0.00E+00 |  | 'RIBOSE_e <=> ' |
| 'EX_LLACTATE_e' | 0.00E+000 |  | 6.50E+00 |  | 6.50E+00 |  | -6.41E+00 |  | 'LLACTATE_e <=> ' |
| 'EX_FRU_e' | 0.00E+000 |  | 0.00E+00 |  | 0.00E+00 |  | 0.00E+00 |  | 'FRU_e <=> ' |
| 'EX_ASCORBATE_e' | 0.00E+000 |  | 0.00E+00 |  | 0.00E+00 |  | 0.00E+00 |  | 'ASCORBATE_e <=> ' |
| 'EX_MANNOSE_e' | 0.00E+000 |  | 0.00E+00 |  | 0.00E+00 |  | 0.00E+00 |  | 'MANNOSE_e <=> ' |
| 'EX_CPD4422_e' | 0.00E+000 |  | 0.00E+00 |  | 0.00E+00 |  | 0.00E+00 |  | 'CPD4422_e <=> ' |
| 'EX_GLYCEROL_e' | -5.10E-001 |  | -3.10E-01 |  | -3.10E-01 |  | -3.10E-01 |  | 'GLYCEROL_e <=> ' |
| 'EX_SER_e' | -6.13E-003 |  | -8.77E-03 |  | -9.99E-02 |  | -1.37E-01 |  | 'SER_e <=> ' |
| 'EX_GLYCEROL3P_e' | -1.28E-001 |  | -1.28E-01 |  | -1.28E-01 |  | -1.28E-01 |  | 'GLYCEROL3P_e <=> ' |
| 'EX_MANNITOL_e' | 0.00E+000 |  | 0.00E+00 |  | 0.00E+00 |  | 0.00E+00 |  | 'MANNITOL_e <=> ' |
| 'EX_DGlucose_e' | -5.11E+000 |  | -2.30E+00 |  | -5.11E+00 |  | -5.11E+00 |  | 'DGlucose_e <=> ' |
| 'EX_LALPHAALANINE_e' | -8.01E-003 |  | -1.15E-02 |  | -1.31E-01 |  | -1.79E-01 |  | 'LALPHAALANINE_e <=> ' |
| 'EX_ARG_e' | -3.84E-003 |  | -5.49E-03 |  | -6.26E-02 |  | -8.59E-02 |  | 'ARG_e <=> ' |
| 'EX_ASN_e' | -5.77E-003 |  | -8.25E-03 |  | -9.40E-02 |  | -1.29E-01 |  | 'ASN_e <=> ' |
| 'EX_LASPARTATE_e' | -5.41E-003 |  | -7.74E-03 |  | -8.82E-02 |  | -1.21E-01 |  | 'LASPARTATE_e <=> ' |
| 'EX_CYS_e' | -8.65E-004 |  | -1.24E-03 |  | -1.41E-02 |  | -1.94E-02 |  | 'CYS_e <=> ' |
| 'EX_GLT_e' | -6.17E-003 |  | -8.83E-03 |  | -1.01E-01 |  | -1.38E-01 |  | 'GLT_e <=> ' |
| 'EX_GLN_e' | -5.53E-003 |  | -7.91E-03 |  | -9.01E-02 |  | -1.24E-01 |  | 'GLN_e <=> ' |
| 'EX_GLY_e' | -6.26E-003 |  | -8.96E-03 |  | -1.02E-01 |  | -1.40E-01 |  | 'GLY_e <=> ' |
| 'EX_HIS_e' | -1.79E-003 |  | -2.55E-03 |  | -2.91E-02 |  | -3.99E-02 |  | 'HIS_e <=> ' |
| 'EX_ILE_e' | -6.39E-003 |  | -9.14E-03 |  | -1.04E-01 |  | -1.43E-01 |  | 'ILE_e <=> ' |
| 'EX_LEU_e' | -8.87E-003 |  | -1.27E-02 |  | -1.45E-01 |  | -1.98E-01 |  | 'LEU_e <=> ' |
| 'EX_LYS_e' | -9.28E-003 |  | -1.33E-02 |  | -1.51E-01 |  | -2.07E-01 |  | 'LYS_e <=> ' |
| 'EX_MET_e' | -1.45E-003 |  | -2.07E-03 |  | -2.36E-02 |  | -3.24E-02 |  | 'MET_e <=> ' |
| 'EX_PHE_e' | -4.45E-003 |  | -6.36E-03 |  | -7.25E-02 |  | -9.95E-02 |  | 'PHE_e <=> ' |
| 'EX_PRO_e' | -4.19E-003 |  | -6.00E-03 |  | -6.83E-02 |  | -9.38E-02 |  | 'PRO_e <=> ' |
| 'EX_THR_e' | -6.11E-003 |  | -8.73E-03 |  | -9.95E-02 |  | -1.37E-01 |  | 'THR_e <=> ' |
| 'EX_TRP_e' | -9.07E-004 |  | -1.30E-03 |  | -1.48E-02 |  | -2.03E-02 |  | 'TRP_e <=> ' |
| 'EX_TYR_e' | -2.91E-003 |  | -4.16E-03 |  | -4.75E-02 |  | -6.51E-02 |  | 'TYR_e <=> ' |
| 'EX_VAL_e' | -7.27E-003 |  | -1.04E-02 |  | -1.19E-01 |  | -1.63E-01 |  | 'VAL_e <=> ' |
| 'EX_GUANINE_e' | -1.37E-003 |  | -1.95E-03 |  | -2.23E-02 |  | -3.06E-02 |  | 'GUANINE_e <=> ' |
| 'EX_URACIL_e' | 0.00E+000 |  | 0.00E+00 |  | 0.00E+00 |  | 0.00E+00 |  | 'URACIL_e <=> ' |
| 'EX_ADENINE_e' | -1.99E-003 |  | -2.85E-03 |  | -3.25E-02 |  | -4.46E-02 |  | 'ADENINE_e <=> ' |
| 'EX_THYMINE_e' | -9.96E-004 |  | -1.42E-03 |  | -1.62E-02 |  | -2.23E-02 |  | 'THYMINE_e <=> ' |
| 'EX_CYTIDINE_e' | 0.00E+000 |  | 0.00E+00 |  | 0.00E+00 |  | 0.00E+00 |  | 'CYTIDINE_e <=> ' |
| 'EX_DEOXYCYTIDINE_e' | -1.88E-003 |  | -2.69E-03 |  | -3.07E-02 |  | -4.21E-02 |  | 'DEOXYCYTIDINE_e <=> ' |
| 'EX_Pi_e' | 1.19E-001 |  | 1.15E-01 |  | -1.91E-02 |  | -7.38E-02 |  | 'Pi_e <=> ' |
| 'EX_ACET_e' | 1.08E+001 |  | 6.69E+00 |  | 1.21E+01 |  | 2.49E+01 |  | 'ACET_e <=> ' |
| 'EX_PYRUVATE_e' | 0.00E+000 |  | -8.16E+00 |  | -8.15E+00 |  | -8.13E+00 |  | 'PYRUVATE_e <=> ' |
| 'EX_CARBONDIOXIDE__e' | 1.08E+001 |  | 6.69E+00 |  | 1.21E+01 |  | 2.49E+01 |  | 'CARBONDIOXIDE__e <=> ' |
| 'EX_WATER_e' | 1.11E+001 |  | -1.17E+00 |  | 7.62E+00 |  | 2.18E+01 |  | 'WATER_e <=> ' |
| 'EX_OXYGENMOLECULE__e' | -1.15E+001 |  | -3.03E+00 |  | -8.41E+00 |  | -2.12E+01 |  | 'OXYGENMOLECULE__e <=> ' |
| 'EX_AMMONIA__e' | 1.48E-003 |  | 2.12E-03 |  | 2.41E-02 |  | 3.31E-02 |  | 'AMMONIA__e <=> ' |
| 'EX_RIBOFLAVIN_e' | 0.00E+000 |  | 0.00E+00 |  | 0.00E+00 |  | 0.00E+00 |  | 'RIBOFLAVIN_e <=> ' |
| 'EX_HYDROGENPEROXIDE_e' | 6.34E-001 |  | 4.31E-01 |  | 3.71E-01 |  | 3.46E-01 |  | 'HYDROGENPEROXIDE_e <=> ' |
| 'EX_LongChainFattyAcids_e' | -5.53E-003 |  | -7.92E-03 |  | -9.02E-02 |  | -1.24E-01 |  | 'LongChainFattyAcids_e <=> ' |
| 'EX_PANTETHEINEP_e' | 0.00E+000 |  | 0.00E+00 |  | 0.00E+00 |  | 0.00E+00 |  | 'PANTETHEINEP_e <=> ' |
| 'EX_PHOSPHATIDYL_CHOLINE_e' | -1.66E-003 |  | -2.37E-03 |  | -2.71E-02 |  | -3.71E-02 |  | 'PHOSPHATIDYL_CHOLINE_e <=> ' |
| 'EX_MYOINOSITOL_e' | 0.00E+000 |  | 0.00E+00 |  | 0.00E+00 |  | 0.00E+00 |  | 'MYOINOSITOL_e <=> ' |
| 'EX_NIACINE_e' | 0.00E+000 |  | 0.00E+00 |  | 0.00E+00 |  | 0.00E+00 |  | 'NIACINE_e <=> ' |
| 'Protein_synthesis' | 6.37E-004 | 5.26E-01 | 7.61E-04 | 6.28E-01 | 5.04E-03 | 4.17E+00 | 6.79E-03 | 0.00E+00 | '482 WATER_c + 22 ChargedGLTtRNAs_c + 21 ChargedTHRtRNAs_c + 21 ChargedGLYtRNAs_c + 32 ChargedLYStRNAs_c + 21 ChargedSERtRNAs_c + 6 ChargedHIStRNAs_c + 826 ATP_c + 13 ChargedARGtRNAs_c + 17 ChargedGLNtRNAs_c + 3 ChargedTRPtRNAs_c + 29 ChargedLEUtRNAs_c + 25 ChargedVALtRNAs_c + 14 ChargedPROtRNAs_c + 15 ChargedPHEtRNAs_c + 22 ChargedILEtRNAs_c + 10 ChargedTYRtRNAs_c + 27 ChargedALAtRNAs_c + 18 ChargedASPtRNAs_c + 20 ChargedASNtRNAs_c + 5 ChargedMETtRNAs_c + 3 ChargedCYStRNAs_c -> 21 THRtRNAs_c + 17 GLNtRNAs_c + 22 ILEtRNAs_c + 29 LEUtRNAs_c + 826 ADP_c + 21 GLYtRNAs_c + 5 METtRNAs_c + 25 VALtRNAs_c + 13 ARGtRNAs_c + 826 PROTON_c + 20 ASNtRNAs_c + 21 SERtRNAs_c + 10 TYRtRNAs_c + 826 Pi_c + 32 LYStRNAs_c + 18 ASPtRNAs_c + 14 PROtRNAs_c + 27 ALAtRNAs_c + 3 TRPtRNAs_c + 22 GLTtRNAs_c + 3 CYStRNAs_c + 6 HIStRNAs_c + 15 PHEtRNAs_c + PROT_mol_c ' |
| 'Protein_synthesis_gram' | 1.09E-002 |  | 1.57E-02 |  | 1.78E-01 |  | 2.45E-01 |  | '0.0263 PROT_mol_c <=> PROT_g_c ' |
| 'ACP_synthesis' | 1.87E-006 | 3.72E-04 | 2.67E-06 | 5.32E-04 | 3.05E-05 | 6.06E-03 | 4.18E-05 | 4.87E+01 | '199 WATER_c + 11 ChargedGLTtRNAs_c + ChargedTHRtRNAs_c + ChargedGLYtRNAs_c + 11 ChargedLYStRNAs_c + 5 ChargedSERtRNAs_c + ChargedHIStRNAs_c + 199 ATP_c + 2 ChargedARGtRNAs_c + 4 ChargedGLNtRNAs_c + 12 ChargedLEUtRNAs_c + 5 ChargedVALtRNAs_c + ChargedPROtRNAs_c + 5 ChargedPHEtRNAs_c + 8 ChargedILEtRNAs_c + 4 ChargedALAtRNAs_c + 6 ChargedASPtRNAs_c + 3 ChargedASNtRNAs_c + 4 ChargedMETtRNAs_c -> THRtRNAs_c + 4 GLNtRNAs_c + 8 ILEtRNAs_c + 12 LEUtRNAs_c + 199 ADP_c + GLYtRNAs_c + 4 METtRNAs_c + 5 VALtRNAs_c + 2 ARGtRNAs_c + 199 PROTON_c + 3 ASNtRNAs_c + 5 SERtRNAs_c + 199 Pi_c + 11 LYStRNAs_c + 6 ASPtRNAs_c + PROtRNAs_c + 4 ALAtRNAs_c + 11 GLTtRNAs_c + HIStRNAs_c + ACP_c + 5 PHEtRNAs_c ' |
| 'ACP_synthesis_gram' | 1.77E-005 |  | 2.53E-05 |  | 2.88E-04 |  | 3.95E-04 |  | '0.10579 ACP_c <=> ACP_g_c ' |
| 'Protein_degradation' | 3.49E-004 | 2.40E-01 | 3.49E-04 | 2.40E-01 | 3.49E-04 | 2.40E-01 | 3.49E-04 | 2.72E-01 | '1032 WATER_c + 688 ATP_c + PROT_mol_c -> 25 VAL_c + 17 GLN_c + 21 GLY_c + 22 GLT_c + 6 HIS_c + 688 ADP_c + 14 PRO_c + 10 TYR_c + 688 PROTON_c + 18 LASPARTATE_c + 21 SER_c + 20 ASN_c + 3 CYS_c + 5 MET_c + 21 THR_c + 15 PHE_c + 688 Pi_c + 27 LALPHAALANINE_c + 13 ARG_c + 22 ILE_c + 32 LYS_c + 3 TRP_c + 29 LEU_c ' |
| 'ACP_degradation' | 0.00E+000 |  | 0.00E+00 |  | 0.00E+00 |  | 0.00E+00 |  | '219 WATER_c + 146 ATP_c + ACP_c -> 5 VAL_c + 4 GLN_c + GLY_c + 11 GLT_c + HIS_c + 146 ADP_c + PRO_c + 146 PROTON_c + 6 LASPARTATE_c + 5 SER_c + 3 ASN_c + 4 MET_c + THR_c + 5 PHE_c + 146 Pi_c + 4 LALPHAALANINE_c + 2 ARG_c + 8 ILE_c + 11 LYS_c + 12 LEU_c ' |
| 'DNA_synthesis' | 1.40E-005 | 1.96E-03 | 2.00E-05 | 2.80E-03 | 2.28E-04 | 3.19E-02 | 3.13E-04 | 0.00E+00 | '140 WATER_c + 71.2 TTP_c + 71.2 DATP_c + 140 ATP_c + 28.8 DGTP_c + 28.8 DCTP_c -> 140 ADP_c + 140 PROTON_c + 140 Pi_c + 200 PPI_c + DNA_mmol_c ' |
| 'DNA_synthesis_gram' | 8.83E-004 |  | 1.26E-03 |  | 1.44E-02 |  | 1.97E-02 |  | '0.015849 DNA_mmol_c <=> DNA_g_c ' |
| 'RNA_synthesis' | 7.78E-003 | 5.37E-01 | 7.79E-03 | 5.38E-01 | 8.30E-03 | 5.73E-01 | 8.51E-03 | 1.36E+00 | '40 WATER_c + 18 CTP_c + 69 ATP_c + 28 GTP_c + 25 UTP_c -> 40 ADP_c + 40 PROTON_c + 40 Pi_c + 100 PPI_c + RNA_mmol_c ' |
| 'RNA_synthesis_gram' | 1.15E-003 |  | 1.64E-03 |  | 1.87E-02 |  | 2.57E-02 |  | '0.029982 RNA_mmol_c <=> RNA_g_c ' |
| 'RNA_degradation' | 7.74E-003 |  | 7.74E-03 |  | 7.74E-03 |  | 7.74E-03 |  | '100 WATER_c + RNA_mmol_c -> 18 CMP_c + 100 PROTON_c + 25 UMP_c + 29 AMP_c + 28 GMP_c ' |
| 'LPHOSPHATIDATE_synthesis_gram' | 1.06E-004 |  | 1.52E-04 |  | 1.73E-03 |  | 2.37E-03 |  | '1.389 LPHOSPHATIDATE_c <=> LPHOSPHATIDATE_g_c ' |
| 'CARDIOLIPIN_synthesis_gram' | 1.77E-003 |  | 2.53E-03 |  | 2.88E-02 |  | 3.95E-02 |  | '0.74195 CARDIOLIPIN_c <=> CARDIOLIPIN_g_c ' |
| 'PHOSPHATIDYL_CHOLINE_gram' | 0.00E+000 |  | 0.00E+00 |  | 0.00E+00 |  | 0.00E+00 |  | '1.3661 PHOSPHATIDYL_CHOLINE_c <=> PHOSPHATIDYL_CHOLINE_g_c ' |
| 'LIPID_synthesis' | 3.53E-003 |  | 5.05E-03 |  | 5.76E-02 |  | 7.90E-02 |  | '0.47 PHOSPHATIDYL_CHOLINE_c + 0.03 LPHOSPHATIDATE_g_c + 0.5 CARDIOLIPIN_g_c -> LIPIDS_c ' |
| 'G6P_gram' | 0.00E+000 |  | 0.00E+00 |  | 0.00E+00 |  | 0.00E+00 |  | '1.3661 Dglucose6phosphate_c <=> Dglucose6phosphate_g_c ' |
| 'AAbiomass_mol' | 2.34E-008 |  | 3.34E-08 |  | 3.81E-07 |  | 5.23E-07 |  | '2793 VAL_c + 294 GLN_c + 9220 GLY_c + 18651 GLT_c + 2422 HIS_c + 6837 PRO_c + 1366 TYR_c + 9318 LASPARTATE_c + 3202 SER_c + 54 ASN_c + 67 CYS_c + 100 MET_c + 2489 THR_c + 5122 PHE_c + 9824 LALPHAALANINE_c + 3913 ARG_c + 1858 ILE_c + 1741 LYS_c + 1864 TRP_c + 21313 LEU_c -> AAbiomass_mol_c ' |
| 'AAbiomass_gram' | 2.65E-004 |  | 3.79E-04 |  | 4.32E-03 |  | 5.92E-03 |  | '8.8228e-05 AAbiomass_mol_c <=> AAbiomass_g_c ' |
| 'Biomass_synthesis' | 1.77E-002 | 2.04E-01 | 2.53E-02 | 2.91E-01 | 2.88E-01 | 3.32E+00 | 3.95E-01 | 6.83E-02 | '11.53 WATER_c + 11.53 ATP_c + 0.62 PROT_g_c + 0.001 ACP_g_c + 0.05 DNA_g_c + 0.065 RNA_g_c + 0.2 LIPIDS_c + 0.015 AAbiomass_g_c -> 11.53 ADP_c + 11.53 PROTON_c + 11.53 Pi_c + BIOMASS_g_c ' |
| 'EX_BIOMASS_g_c' | 1.77E-002 |  | 2.53E-02 |  | 2.88E-01 |  | 3.95E-01 |  | 'BIOMASS_g_c <=> ' |
| 'DEOXYGUANOSINEKINASERXN' | 4.03E-004 | 4.03E-04 | 5.76E-04 | 5.76E-04 | 6.57E-03 | 6.57E-03 | 9.01E-03 | 3.95E-01 | 'ATP_c + DEOXYGUANOSINE_c <=> ADP_c + PROTON_c + DGMP_c ' |
| 'DEOXYADENOSINEKINASERXN' | 8.04E-005 | 8.04E-05 | 1.15E-04 | 1.15E-04 | 1.31E-03 | 1.31E-03 | 1.80E-03 | 9.01E-03 | 'ATP_c + DEOXYADENOSINE_c <=> ADP_c + PROTON_c + DAMP_c ' |
| 'UMPKINASERXN' | 1.95E-001 | 1.95E-01 | 1.96E-01 | 1.96E-01 | 2.18E-01 | 2.18E-01 | 2.27E-01 | 1.80E-03 | 'ATP_c + UMP_c <=> UDP_c + ADP_c ' |
| 'ACYLCOASYNTHRXN' | 5.53E-003 | 5.53E-03 | 7.92E-03 | 7.92E-03 | 9.02E-02 | 9.02E-02 | 1.24E-01 | 2.27E-01 | 'ATP_c + COA_c + LongChainFattyAcids_c <=> PPI_c + AMP_c + LongChainAcylCoAs_c ' |
| 'NADKINRXN' | -9.16E-004 | -9.16E-04 | -1.31E-03 | -1.31E-03 | -1.49E-02 | -1.49E-02 | -2.05E-02 | 1.24E-01 | 'ATP_c + NAD_c <=> ADP_c + PROTON_c + NADP_c ' |
| 'NADHKINRXN' | 9.16E-004 | 9.16E-04 | 1.31E-03 | 1.31E-03 | 1.49E-02 | 1.49E-02 | 2.05E-02 | -2.05E-02 | 'ATP_c + NADH_c <=> ADP_c + PROTON_c + NADPH_c ' |
|  |  |  |  |  |  |  |  |  |  |
|  | **ATP production** | **-21.9** | **ATP production** | **-13.7** | **ATP production** | **-25.2** | **ATP production** | **-38.0** |  |
|  | **ATP consumption** | **21.9** | **ATP consumption** | **13.7** | **ATP consumption** | **25.2** | **ATP consumption** | **77.4** |  |
|  | NGAM (%) | 84.2 | NGAM (%) | 73.8 | NGAM (%) | 40.3 | NGAM (%) | 23.8 |  |
|  | GAM (%) | 0.9 | GAM (%) | 2.1 | GAM (%) | 13.2 | GAM (%) | 0.1 |  |
|  | Growth (%) | 14.9 | Growth (%) | 24.0 | Growth (%) | 46.5 | Growth (%) | 76.1 |  |
