# Supplementary material for: Metabolic modeling of energy balances in Mycoplasma hyopneumoniae shows that pyruvate addition increases growth rate
Source: Biotechnol Bioeng. 2017 Jul 27;114(10):2339–47. doi: 10.1002/bit.26347 (PMC6084303; doi:10.1002/bit.26347)
Supplement: Supplementary file 6 — Table S5. Flux variability analysis at 99.99% of maximal initial growth rate. [file BIT-114-2339-s006.pdf]

Table S5: flux variability analysis at 99.99% of maximal initial growth rate

Name	Basic solution	MIN	MAX	Allowed variation (%)	Variation >0.00001?	Significant	Direction (	Reaction
'RXN01061'	1.84E+001	1.84E+001	1.84E+001	0.000225743	FALSE	FALSE	FALSE	'WATER_c + ATP_c <=> ADP_c + PROTON_c + Pi_c '
'A3__46__6__46__5__46__3RXN'	0.00E+000	0.00E+000	4.16E-005	#DIV/0!	FALSE	FALSE	FALSE	'WATER_c + GTP_c -> PROTON_c + Pi_c + GDP_c '
'RXN18KM18'	0.00E+000	0.00E+000	0.00E+000	#DIV/0!	FALSE	FALSE	FALSE	'WATER_c + PRPP_c + ATP_c + NIACINE_c -> ADP_c + Pi_c + PPI_c + NICOTINATE_NUCLEOTIDE_c '
'URACILPRIBOSYLTRANSRXN'	-1.48E-003	-1.51E-003	-1.48E-003	-2.246884755	FALSE	FALSE	FALSE	'UMP_c + PPI_c <=> PRPP_c + URACIL_c '
'TRANSRXN168'	0.00E+000	0.00E+000	0.00E+000	#DIV/0!	FALSE	FALSE	FALSE	'PHOSPHOENOLPYRUVATE_c + MANNOSE_e -> MANNOSE6P_c + PYRUVATE_c '
'DCTPPYROPHOSPHATASERXN'	0.00E+000	0.00E+000	2.08E-005	#DIV/0!	FALSE	FALSE	FALSE	'WATER_c + DCTP_c -> PROTON_c + DCMP_c + PPI_c '
'RXN2902'	0.00E+000	0.00E+000	0.00E+000	#DIV/0!	FALSE	FALSE	FALSE	'COA_c + NAD_c + MALONATESALD_c -> ACETYLCOA_c + NADH_c + CARBONDIOXIDE_c '
'DUTPPYROPRXN'	0.00E+000	0.00E+000	0.00E+000	#DIV/0!	FALSE	FALSE	FALSE	'WATER_c + DUTP_c -> PROTON_c + PPI_c + DUMP_c '
'A4__46__1__46__2__46__29RXN'	0.00E+000	0.00E+000	0.00E+000	#DIV/0!	FALSE	FALSE	FALSE	'CPD645_c -> DIHYDROXYACETONEPHOSPHATE_c + MALONATESALD_c '
'RXN18KM8'	4.03E-004	4.03E-004	4.24E-004	5.156218728	FALSE	FALSE	FALSE	'DCDP_c + PHOSPHOENOLPYRUVATE_c + PROTON_c -> DCTP_c + PYRUVATE_c '
'MYOINOSITOL2DEHYDROGENASERXN'	0.00E+000	0.00E+000	0.00E+000	#DIV/0!	FALSE	FALSE	FALSE	'MYOINOSITOL_c + NAD_c -> PROTON_c + CPD365_c + NADH_c '
'RXN05292'	0.00E+000	0.00E+000	4.16E-005	#DIV/0!	FALSE	FALSE	FALSE	'WATER_c + DCMP_c -> DEOXYCYTIDINE_c + Pi_c '
'A2__46__7__46__7__46__15RXN'	0.00E+000	0.00E+000	0.00E+000	#DIV/0!	FALSE	FALSE	FALSE	'CTP_c + PHOSPHORYLCHOLINE_c + PROTON_c -> CDPCHOLINE_c + PPI_c '
'CDPDIGLYSYNRXN'	2.62E-003	2.62E-003	2.62E-003	0.005517349	FALSE	FALSE	FALSE	'CTP_c + LPHOSPHATIDATE_c + PROTON_c -> CDPDIACYLGLYCEROL_c + PPI_c '
'RXN1381'	2.77E-003	2.77E-003	2.77E-003	0.005517349	FALSE	FALSE	FALSE	'GLYCEROL3P_c + LongChainAcylCoAs_c -> ACYLSNGLYCEROL3P_c + COA_c
'RXN05375'	0.00E+000	0.00E+000	0.00E+000	#DIV/0!	FALSE	FALSE	FALSE	'2 PROTON_c + CPD01147_c + 2 NADH_c <=> ANTHRANILATE_c + CPD01148_c + 2 NAD_c '
'NACETYLGLUCOSAMINEKINASERXN'	0.00E+000	0.00E+000	0.00E+000	#DIV/0!	FALSE	FALSE	FALSE	'ATP_c + NACETYLDGLUCOSAMINE_c -> ADP_c + PROTON_c + NACETYLDGLUCOSAMINE6P_c '
'GTPCYCLOHYDROIRXN'	0.00E+000	0.00E+000	0.00E+000	#DIV/0!	FALSE	FALSE	FALSE	'WATER_c + GTP_c <=> PROTON_c + DIHYDRONEOPTERINP3_c + FORMATE_c '
'CHOLINEKINASERXN'	0.00E+000	0.00E+000	0.00E+000	#DIV/0!	FALSE	FALSE	FALSE	'ATP_c + CHOLINE_c -> ADP_c + PHOSPHORYLCHOLINE_c + PROTON_c '
'ATPSYNRXN'	-1.11E-001	-1.11E-001	-1.11E-001	-0.010821451	FALSE	FALSE	FALSE	'WATER_c + 3 PROTON_c + ATP_c <=> 4 PROTON_e + ADP_c + Pi_c '
'RXN3523'	0.00E+000	0.00E+000	0.00E+000	#DIV/0!	FALSE	FALSE	FALSE	'2 CPD318_c -> PROTON_c + ASCORBATE_c + LDEHYDROASCORBATE_c '
'THYMIDYLATE5PHOSPHATASERXN'	0.00E+000	0.00E+000	4.16E-005	#DIV/0!	FALSE	FALSE	FALSE	'WATER_c + TMP_c -> THYMIDINE_c + Pi_c '
'GAPOXNPHOSPHNRXN'	1.08E+001	1.08E+001	1.08E+001	0.000191582	FALSE	FALSE	FALSE	'Pi_c + GAP_c + NAD_c <=> DPG_c + PROTON_c + NADH_c '
'R503RXN'	0.00E+000	0.00E+000	0.00E+000	#DIV/0!	FALSE	FALSE	FALSE	'WATER_c + CPD15167_c -> PROTON_c + CPD827_c '
'GMKALTRXN'	4.03E-004	0.00E+000	4.03E-004	100	TRUE	FALSE	FALSE	'ATP_c + DGMP_c -> ADP_c + DGDP_c '
'RXN05305'	0.00E+000	0.00E+000	0.00E+000	#DIV/0!	FALSE	FALSE	FALSE	'CPD01108_c <=> RIBOSE_c '
'LACTOSE6PHOSPHATEISOMERASERXN'	0.00E+000	0.00E+000	0.00E+000	#DIV/0!	FALSE	FALSE	FALSE	'CPD1241_c <=> TAGATOSE6PHOSPHATE_c '
'RXN18KM6'	1.42E-001	1.42E-001	1.42E-001	0.0256163	FALSE	FALSE	FALSE	'PHOSPHOENOLPYRUVATE_c + PROTON_c + CDP_c -> CTP_c + PYRUVATE_c '
'GLUCOSAMINE6PDEAMINRXN'	0.00E+000	0.00E+000	0.00E+000	#DIV/0!	FALSE	FALSE	FALSE	'WATER_c + DGLUCOSAMINE6P_c -> AMMONIA_c + PROTON_c + FRUCTOSE6P_c '
'RIBULP3EPIMRXN'	-2.90E-003	-2.91E-003	-2.90E-003	-0.17884297	FALSE	FALSE	FALSE	'RIBULOSE5P_c <=> XYLULOSE5PHOSPHATE_c '
'DEOXYADENYLATEKINASERXN'	8.04E-005	3.88E-005	5.17E-004	594.2523832	TRUE	FALSE	FALSE	'ATP_c + DAMP_c -> ADP_c + DADP_c '
'RXN11811'	0.00E+000	0.00E+000	0.00E+000	#DIV/0!	FALSE	FALSE	FALSE	'AMMONIA_c + PROTON_c <=> AMMONIUM_c '
'RXN05199'	0.00E+000	0.00E+000	2.08E-005	#DIV/0!	FALSE	FALSE	FALSE	'Pi_c + GUANOSINE_c <=> RIBOSE1P_c + GUANINE_c '
'RXN10981'	0.00E+000	0.00E+000	0.00E+000	#DIV/0!	FALSE	FALSE	FALSE	'Acceptor_c + 2 PROTON_c + ASCORBATE_c -> CPD318_c + DonorH2_c
'RXN12149'	0.00E+000	0.00E+000	0.00E+000	#DIV/0!	FALSE	FALSE	FALSE	'Lrhamnose_c <=> LRHAMNOFURANOSE_c
'A1TRANSKETORXN'	1.45E-003	1.45E-003	1.45E-003	0.178842966	FALSE	FALSE	FALSE	'GAP_c + DSEDOHEPTULOSE7P_c <=> XYLULOSE5PHOSPHATE_c + RIBOSE5P_c '
'PGPPHOSPHARXN'	1.31E-003	1.31E-003	1.31E-003	0.005517349	FALSE	FALSE	FALSE	'WATER_c + L1PHOSPHATIDYLGLYCEROLP_c -> L1PHOSPHATIDYLGLYCEROL_c + Pi_c '
'A2TRANSKETORXN'	-1.45E-003	-1.45E-003	-1.45E-003	-0.178842966	FALSE	FALSE	FALSE	'XYLULOSE5PHOSPHATE_c + ERYTHROSE4P_c <=> GAP_c + FRUCTOSE6P_c '
'RXN12440'	0.00E+000	0.00E+000	0.00E+000	#DIV/0!	FALSE	FALSE	FALSE	'HYDROGENPEROXIDE_c + PROTON_c -> 2 WATER_c + ASCORBATE_c + LDEHYDROASCORBATE_c '
'RXN1623'	2.77E-003	2.77E-003	2.77E-003	0.005517349	FALSE	FALSE	FALSE	'ACYLSNGLYCEROL3P_c + LongChainAcylCoAs_c -> LPHOSPHATIDATE_c + COA_c
'RXN12754'	0.00E+000	0.00E+000	0.00E+000	#DIV/0!	FALSE	FALSE	FALSE	'WATER_c + NADH_c -> CPD02472_c '
'DEOXYGUANPHOSPHORRXN'	-4.03E-004	-4.03E-004	0.00E+000	-100	TRUE	FALSE	FALSE	'Pi_c + DEOXYGUANOSINE_c <=> DEOXYDRIBOSE1PHOSPHATE_c + GUANINE_c '
'DEOXYCYTIDINEKINASERXN'	4.03E-004	3.70E-004	4.45E-004	18.56238747	FALSE	FALSE	FALSE	'DEOXYCYTIDINE_c + ATP_c -> ADP_c + PROTON_c + DCMP_c '
'RXN12753'	0.00E+000	0.00E+000	0.00E+000	#DIV/0!	FALSE	FALSE	FALSE	'WATER_c + NADH_c -> CPD653_c '
'ADENPHOSPHORRXN'	0.00E+000	0.00E+000	2.08E-005	#DIV/0!	FALSE	FALSE	FALSE	'ADENOSINE_c + Pi_c <=> RIBOSE1P_c + ADENINE_c '
'RXN12862'	0.00E+000	0.00E+000	0.00E+000	#DIV/0!	FALSE	FALSE	FALSE	'WATER_c + LDEHYDROASCORBATE_c -> CPD13907_c '
'RXN8141'	1.31E-003	1.31E-003	1.31E-003	0.005517349	FALSE	FALSE	FALSE	'L1PHOSPHATIDYLGLYCEROL_c + CDPDIACYLGLYCEROL_c <=> CMP_c + CARDIOLIPIN_c + PROTON_c '
'PANTEPADENYLYLTRANRXN'	0.00E+000	0.00E+000	0.00E+000	#DIV/0!	FALSE	FALSE	FALSE	'PROTON_c + ATP_c + PANTETHEINEP_c -> DEPHOSPHOCOA_c + PPI_c '
'TRANSRXN104'	0.00E+000	-4.16E-005	0.00E+000	#DIV/0!	FALSE	FALSE	FALSE	'PROTON_e + LLACTATE_e -> PROTON_c + LLACTATE_c '
'RXN18KM2'	0.00E+000	0.00E+000	0.00E+000	#DIV/0!	FALSE	FALSE	FALSE	'FRU_e + PHOSPHOENOLPYRUVATE_c -> FRU1P_c + PYRUVATE_c '
'RXN0705'	0.00E+000	0.00E+000	0.00E+000	#DIV/0!	FALSE	FALSE	FALSE	'PROTON_c + CPD2343_c -> CARBONDIOXIDE_c + LXYLULOSE5P_c '
'NADHDEHYDROGENASERXN'	1.08E+001	1.08E+001	1.08E+001	0.00038318	FALSE	FALSE	FALSE	'2 PROTON_c + 2 NADH_c + OXYGENMOLECULE_c <=> 2 WATER_c + 2 NAD_c '
'RIBULPEPIMRXN'	0.00E+000	0.00E+000	0.00E+000	#DIV/0!	FALSE	FALSE	FALSE	'LRIBULOSE5P_c <=> XYLULOSE5PHOSPHATE_c '
'A1__46__2__46__1__46__27RXN'	0.00E+000	0.00E+000	0.00E+000	#DIV/0!	FALSE	FALSE	FALSE	'WATER_c + COA_c + CPD12179_c + NAD_c <=> PROTON_c + PROPIONYLCOA_c + NADH_c + HCO3_c '
'PHOSPHOGLYCERATEKINASEGTPRXN'	-2.18E-001	-2.18E-001	5.18E+000	-2479.446377	TRUE	FALSE	TRUE	'GTP_c + G3P_c <=> DPG_c + GDP_c '
'RXN12863'	0.00E+000	0.00E+000	0.00E+000	#DIV/0!	FALSE	FALSE	FALSE	'HYDROGENPEROXIDE_c + CPD13907_c -> 3 PROTON_c + OXALATE_c + LTHREONATE_c '
'RXN12869'	0.00E+000	0.00E+000	0.00E+000	#DIV/0!	FALSE	FALSE	FALSE	'CPD13907_c -> 3 PROTON_c + CPD13914_c '
'RXN18KM13'	-9.96E-004	-9.96E-004	5.40E+000	-541798.2693	TRUE	FALSE	TRUE	'DATP_c + G3P_c <=> DPG_c + DADP_c '
'RXN12861'	0.00E+000	0.00E+000	0.00E+000	#DIV/0!	FALSE	FALSE	FALSE	'CPD13907_c -> CPD334_c + PROTON_c '
'RXN14143'	0.00E+000	0.00E+000	4.16E-005	#DIV/0!	FALSE	FALSE	FALSE	'WATER_c + DUMP_c -> Pi_c + DEOXYURIDINE_c '
'A5__46__3__46__1__46__17RXN'	0.00E+000	0.00E+000	0.00E+000	#DIV/0!	FALSE	FALSE	FALSE	'CPD37_c <=> CPD343_c '
'RXN8654'	0.00E+000	0.00E+000	0.00E+000	#DIV/0!	FALSE	FALSE	FALSE	'PROTON_c + ATP_c + LIPOICACID_c -> LIPOYLAMP_c + PPI_c '
'RXN02461'	0.00E+000	0.00E+000	0.00E+000	#DIV/0!	FALSE	FALSE	FALSE	'PHOSPHOENOLPYRUVATE_c + ASCORBATE_e -> LASCORBATE6PHOSPHATE_c + PYRUVATE_c '
'RIBOFLAVINKINRXN'	0.00E+000	0.00E+000	0.00E+000	#DIV/0!	FALSE	FALSE	FALSE	'RIBOFLAVIN_c + ATP_c -> FMN_c + ADP_c + PROTON_c '
'ETHANOLAMINEKINASERXN'	0.00E+000	0.00E+000	0.00E+000	#DIV/0!	FALSE	FALSE	FALSE	'ATP_c + ETHANOLAMINE_c <=> PHOSPHORYLETHANOLAMINE_c + ADP_c + PROTON_c '
'AMPDEPHOSPHORYLATIONRXN'	0.00E+000	0.00E+000	2.08E-005	#DIV/0!	FALSE	FALSE	FALSE	'WATER_c + AMP_c -> ADENOSINE_c + Pi_c '
'RXN14142'	0.00E+000	0.00E+000	4.16E-005	#DIV/0!	FALSE	FALSE	FALSE	'WATER_c + DGMP_c -> Pi_c + DEOXYGUANOSINE_c '
'RXN7609'	0.00E+000	0.00E+000	2.08E-005	#DIV/0!	FALSE	FALSE	FALSE	'WATER_c + GMP_c -> Pi_c + GUANOSINE_c '
'RXN12872'	0.00E+000	0.00E+000	0.00E+000	#DIV/0!	FALSE	FALSE	FALSE	'CPD13914_c -> CPD13910_c '
'RXN14025'	0.00E+000	0.00E+000	2.08E-005	#DIV/0!	FALSE	FALSE	FALSE	'WATER_c + UMP_c -> URIDINE_c + Pi_c '
'RXN14026'	0.00E+000	0.00E+000	1.28E-005	#DIV/0!	FALSE	FALSE	FALSE	'WATER_c + CMP_c -> Pi_c + CYTIDINE_c '
'RXN05214'	0.00E+000	0.00E+000	0.00E+000	#DIV/0!	FALSE	FALSE	FALSE	'WATER_c + LASCORBATE6PHOSPHATE_c <=> CPD2343_c '
'RXN12870'	0.00E+000	0.00E+000	0.00E+000	#DIV/0!	FALSE	FALSE	FALSE	'CPD334_c -> CPD13913_c '
'RXN14150'	0.00E+000	0.00E+000	0.00E+000	#DIV/0!	FALSE	FALSE	FALSE	'CPD827_c <=> CPD15127_c '
'GUANPRIBOSYLTRANRXN'	-9.64E-004	-1.39E-003	-9.63E-004	-43.97968147	TRUE	FALSE	FALSE	'PPI_c + GMP_c <=> PRPP_c + GUANINE_c '
'RXN18KM10'	9.96E-004	9.96E-004	9.96E-004	0.005517349	FALSE	FALSE	FALSE	'PHOSPHOENOLPYRUVATE_c + PROTON_c + TDP_c -> TTP_c + PYRUVATE_c '
'RXN12871'	0.00E+000	0.00E+000	0.00E+000	#DIV/0!	FALSE	FALSE	FALSE	'WATER_c + CPD13913_c -> PROTON_c + CPD13912_c '
'RXN18KM15'	5.40E+000	-4.03E-004	5.40E+000	100.007484	TRUE	FALSE	TRUE	'DGTP_c + G3P_c <=> DPG_c + DGDP_c '
'TRANSALDOLRXN'	-1.45E-003	-1.45E-003	-1.45E-003	-0.178842966	FALSE	FALSE	FALSE	'GAP_c + DSEDOHEPTULOSE7P_c <=> ERYTHROSE4P_c + FRUCTOSE6P_c '
'A3__46__1__46__4__46__2RXN'	0.00E+000	0.00E+000	0.00E+000	#DIV/0!	FALSE	FALSE	FALSE	'WATER_c + L1GLYCEROPHOSPHORYLCHOLINE_c -> GLYCEROL3P_c + PROTON_c + CHOLINE_c '
'RIB5PISOMRXN'	-2.90E-003	-2.91E-003	-2.90E-003	-0.17884297	FALSE	FALSE	FALSE	'RIBOSE5P_c <=> RIBULOSE5P_c '
'RXN18KM7'	1.95E-001	1.95E-001	1.95E-001	0.017049589	FALSE	FALSE	FALSE	'UDP_c + PHOSPHOENOLPYRUVATE_c + PROTON_c -> PYRUVATE_c + UTP_c '
'A3__46__6__46__3__46__20RXN'	1.28E-001	1.28E-001	1.28E-001	0.01627227	FALSE	FALSE	FALSE	'WATER_c + ATP_c + GLYCEROL3P_e <=> GLYCEROL3P_c + ADP_c + PROTON_c + Pi_c '
'DEOXYADENPHOSPHORRXN'	-8.04E-005	-5.17E-004	-3.88E-005	-594.2523829	TRUE	FALSE	FALSE	'Pi_c + DEOXYADENOSINE_c <=> DEOXYDRIBOSE1PHOSPHATE_c + ADENINE_c '
'MANNPDEHYDROGRXN'	0.00E+000	0.00E+000	0.00E+000	#DIV/0!	FALSE	FALSE	FALSE	'MANNITOL1P_c + NAD_c <=> PROTON_c + NADH_c + FRUCTOSE6P_c '
'TRANSRXN156'	0.00E+000	0.00E+000	0.00E+000	#DIV/0!	FALSE	FALSE	FALSE	'PHOSPHOENOLPYRUVATE_c + MANNITOL_e -> MANNITOL1P_c + PYRUVATE_c '
'PHOSACETYLTRANSRXN'	1.08E+001	1.08E+001	1.08E+001	0.000383164	FALSE	FALSE	FALSE	'ACETYLCOA_c + Pi_c <=> COA_c + ACETYLP_c '
'ACETATEKINRXN'	-1.08E+001	-1.08E+001	-1.08E+001	-0.000383164	FALSE	FALSE	FALSE	'ACET_c + ATP_c <=> ADP_c + ACETYLP_c '
'A3__46__6__46__3__46__17RXN'	0.00E+000	0.00E+000	0.00E+000	#DIV/0!	FALSE	FALSE	FALSE	'WATER_c + RIBOSE_e + ATP_c <=> RIBOSE_c + ADP_c + PROTON_c + Pi_c '
'NICONUCADENYLYLTRANRXN'	0.00E+000	0.00E+000	0.00E+000	#DIV/0!	FALSE	FALSE	FALSE	'PROTON_c + ATP_c + NICOTINATE_NUCLEOTIDE_c <=> PPI_c + DEAMIDONAD_c '
'TRANSRXN131'	5.10E-001	5.10E-001	5.10E-001	0.004074449	FALSE	FALSE	FALSE	'GLYCEROL_e -> GLYCEROL_c '
'LXULRU5PRXN'	0.00E+000	0.00E+000	0.00E+000	#DIV/0!	FALSE	FALSE	FALSE	'LRIBULOSE5P_c <=> LXYLULOSE5P_c
'GLYCEROL3PHOSPHATEOXIDASERXN'	6.34E-001	6.34E-001	6.34E-001	0.003279506	FALSE	FALSE	FALSE	'GLYCEROL3P_c + OXYGENMOLECULE_c <=> HYDROGENPEROXIDE_c + DIHYDROXYACETONEPHOSPHATE_c '
'RXN3715'	5.11E+000	5.11E+000	5.11E+000	0.000203324	FALSE	FALSE	FALSE	'PHOSPHOENOLPYRUVATE_c + DGlucose_e -> Dglucose6phosphate_c + PYRUVATE_c
'RXN11832'	1.42E-001	1.42E-001	1.42E-001	0.011203613	FALSE	FALSE	FALSE	'CMP_c + ATP_c <=> ADP_c + CDP_c '
'RXN7913'	4.03E-004	3.70E-004	4.24E-004	13.40616872	FALSE	FALSE	FALSE	'ATP_c + DCMP_c <=> DCDP_c + ADP_c '
'RXN8631'	0.00E+000	0.00E+000	0.00E+000	#DIV/0!	FALSE	FALSE	FALSE	'FRU1P_c -> DIHYDROXYACETONEPHOSPHATE_c + GLYCERALD_c '
'F16ALDOLASERXN'	5.11E+000	5.11E+000	5.11E+000	0.000203439	FALSE	FALSE	FALSE	'FRUCTOSE16DIPHOSPHATE_c <=> DIHYDROXYACETONEPHOSPHATE_c + GAP_c '
'PRPPSYNRXN'	4.36E-003	4.36E-003	4.40E-003	0.953829165	FALSE	FALSE	FALSE	'ATP_c + RIBOSE5P_c -> PRPP_c + PROTON_c + AMP_c '
'DURIDKIRXN'	0.00E+000	0.00E+000	4.16E-005	#DIV/0!	FALSE	FALSE	FALSE	'ATP_c + DEOXYURIDINE_c -> ADP_c + PROTON_c + DUMP_c '
'THYKIRXN'	9.96E-004	9.96E-004	1.04E-003	4.171323021	FALSE	FALSE	FALSE	'ATP_c + THYMIDINE_c -> ADP_c + PROTON_c + TMP_c '
'INORGPYROPHOSPHATRXN'	9.99E-001	9.99E-001	9.99E-001	0.004158697	FALSE	FALSE	FALSE	'WATER_c + PPI_c -> PROTON_c + 2 Pi_c '
'A3PGAREARRRXN'	1.08E+001	1.08E+001	1.08E+001	0.000191582	FALSE	FALSE	FALSE	'G3P_c <=> A2PG_c '
'NAG6PDEACETRXN'	0.00E+000	0.00E+000	0.00E+000	#DIV/0!	FALSE	FALSE	FALSE	'WATER_c + NACETYLDGLUCOSAMINE6P_c -> ACET_c + DGLUCOSAMINE6P_c '
'DEPHOSPHOCOAKINRXN'	0.00E+000	0.00E+000	0.00E+000	#DIV/0!	FALSE	FALSE	FALSE	'ATP_c + DEPHOSPHOCOA_c -> ADP_c + PROTON_c + COA_c '
'RXN13720'	5.11E+000	5.11E+000	5.11E+000	0.000203324	FALSE	FALSE	FALSE	'Dglucose6phosphate_c <=> FRUCTOSE6P_c
'DEOXYRIBOSEPALDRXN'	0.00E+000	0.00E+000	0.00E+000	#DIV/0!	FALSE	FALSE	FALSE	'DEOXYRIBOSE5P_c -> ACETALD_c + GAP_c '
'URAPHOSPHRXN'	1.48E-003	1.44E-003	1.51E-003	5.055490698	FALSE	FALSE	FALSE	'Pi_c + DEOXYURIDINE_c <=> URACIL_c + DEOXYDRIBOSE1PHOSPHATE_c '
'URPHOSRXN'	0.00E+000	0.00E+000	4.16E-005	#DIV/0!	FALSE	FALSE	FALSE	'URIDINE_c + Pi_c <=> URACIL_c + RIBOSE1P_c '
'THYMPHOSPHRXN'	-9.96E-004	-9.96E-004	-9.96E-004	-0.005517349	FALSE	FALSE	FALSE	'THYMIDINE_c + Pi_c <=> THYMINE_c + DEOXYDRIBOSE1PHOSPHATE_c '
'TRIOSEPISOMERIZATIONRXN'	-5.74E+000	-5.74E+000	-5.74E+000	-0.00036197	FALSE	FALSE	FALSE	'GAP_c <=> DIHYDROXYACETONEPHOSPHATE_c '
'A6PFRUCTPHOSRXN'	5.11E+000	5.11E+000	5.11E+000	0.000203439	FALSE	FALSE	FALSE	'ATP_c + FRUCTOSE6P_c -> ADP_c + PROTON_c + FRUCTOSE16DIPHOSPHATE_c '
'ADENPRIBOSYLTRANRXN'	-1.91E-003	-1.96E-003	-1.48E-003	-24.96460692	TRUE	FALSE	FALSE	'PPI_c + AMP_c <=> PRPP_c + ADENINE_c '
'PEPDEPHOSRXN'	0.00E+000	0.00E+000	5.40E+000	#DIV/0!	TRUE	TRUE	FALSE	'ADP_c + PHOSPHOENOLPYRUVATE_c + PROTON_c -> ATP_c + PYRUVATE_c '
'RXN14207'	5.40E+000	0.00E+000	5.40E+000	100.0000172	TRUE	FALSE	FALSE	'PHOSPHOENOLPYRUVATE_c + PROTON_c + DGDP_c -> DGTP_c + PYRUVATE_c '
'RXN14192'	0.00E+000	0.00E+000	5.40E+000	#DIV/0!	TRUE	TRUE	FALSE	'PHOSPHOENOLPYRUVATE_c + PROTON_c + DADP_c -> DATP_c + PYRUVATE_c '
'RXN14117'	0.00E+000	0.00E+000	5.40E+000	#DIV/0!	TRUE	TRUE	FALSE	'PHOSPHOENOLPYRUVATE_c + PROTON_c + GDP_c -> GTP_c + PYRUVATE_c '
'LLACTATEDEHYDROGENASERXN'	0.00E+000	-4.16E-005	0.00E+000	#DIV/0!	FALSE	FALSE	FALSE	'LLACTATE_c + NAD_c <=> PROTON_c + NADH_c + PYRUVATE_c '
'GUANYLKINRXN'	2.18E-001	2.18E-001	2.18E-001	0.187325317	TRUE	FALSE	FALSE	'ATP_c + GMP_c -> ADP_c + GDP_c '
'CYTIDEAMRXN'	1.48E-003	1.44E-003	1.51E-003	5.055490699	FALSE	FALSE	FALSE	'WATER_c + DEOXYCYTIDINE_c -> AMMONIA_c + DEOXYURIDINE_c '
'CYTIDEAM2RXN'	0.00E+000	0.00E+000	4.16E-005	#DIV/0!	FALSE	FALSE	FALSE	'WATER_c + CYTIDINE_c -> URIDINE_c + AMMONIA_c '
'DPPENTOMUTRXN'	0.00E+000	0.00E+000	0.00E+000	#DIV/0!	FALSE	FALSE	FALSE	'DEOXYDRIBOSE1PHOSPHATE_c <=> DEOXYRIBOSE5P_c '
'PPENTOMUTRXN'	0.00E+000	0.00E+000	4.16E-005	#DIV/0!	FALSE	FALSE	FALSE	'RIBOSE1P_c <=> RIBOSE5P_c '
'ADENYLKINRXN'	4.43E-001	4.42E-001	4.43E-001	0.11729239	TRUE	FALSE	FALSE	'ATP_c + AMP_c -> 2 ADP_c '
'GLYOHMETRANSRXN'	0.00E+000	0.00E+000	0.00E+000	#DIV/0!	FALSE	FALSE	FALSE	'SER_c + THF_c <=> WATER_c + GLY_c + METHYLENETHF_c '
'A5DEHYDRO2DEOXYGLUCONOKINASERXN'	0.00E+000	0.00E+000	0.00E+000	#DIV/0!	FALSE	FALSE	FALSE	'ATP_c + CPD827_c -> ADP_c + PROTON_c + CPD645_c '
'MYOINOSOSE2DEHYDRATASERXN'	0.00E+000	0.00E+000	0.00E+000	#DIV/0!	FALSE	FALSE	FALSE	'CPD365_c -> WATER_c + CPD15127_c '
'A2PGADEHYDRATRXN'	1.08E+001	1.08E+001	1.08E+001	0.000191582	FALSE	FALSE	FALSE	'A2PG_c <=> WATER_c + PHOSPHOENOLPYRUVATE_c '
'DTMPKIRXN'	9.96E-004	9.96E-004	9.96E-004	0.005517349	FALSE	FALSE	FALSE	'ATP_c + TMP_c <=> ADP_c + TDP_c '
'FADSYNRXN'	0.00E+000	0.00E+000	0.00E+000	#DIV/0!	FALSE	FALSE	FALSE	'FMN_c + PROTON_c + ATP_c -> PPI_c + FAD_c '

'CTPSYNRXN'	6.19E-004	6.19E-004	6.53E-004	5.367558022	FALSE	FALSE	FALSE	'WATER_c + GLN_c + ATP_c + UTP_c <=> CTP_c + GLT_c + ADP_c + 2 PROTON_c + Pi_c '
'PHOSPHAGLYPSYNRXN'	1.31E-003	1.31E-003	1.31E-003	0.005517349	FALSE	FALSE	FALSE	'GLYCEROL3P_c + CDPDIACYLGLYCEROL_c -> CMP_c + L1PHOSPHATIDYLGLYCEROLP_c + PROTON_c '
'DIHYDLIPOXNRXN'	0.00E+000	0.00E+000	0.00E+000	#DIV/0!	FALSE	FALSE	FALSE	'DIHYDROLIPOAMIDE_c + NAD_c <=> LIPOAMIDE_c + PROTON_c + NADH_c '
'MANNPISOMRXN'	0.00E+000	0.00E+000	0.00E+000	#DIV/0!	FALSE	FALSE	FALSE	'MANNOSE6P_c <=> FRUCTOSE6P_c '
'PHOSGLYPHOSRXN'	-1.60E+001	-1.60E+001	-1.06E+001	-33.68460052	TRUE	FALSE	FALSE	'ATP_c + G3P_c <=> DPG_c + ADP_c '
'NADSYNTHNH3RXN'	0.00E+000	0.00E+000	0.00E+000	#DIV/0!	FALSE	FALSE	FALSE	'AMMONIA_c + ATP_c + DEAMIDONAD_c -> PPI_c + NAD_c + AMP_c '
'SADENMETSYNRXN'	0.00E+000	0.00E+000	0.00E+000	#DIV/0!	FALSE	FALSE	FALSE	'WATER_c + ATP_c + MET_c -> Pi_c + PPI_c + SADENOSYLMETHIONINE_c '
'OROTPDECARBRXN'	0.00E+000	0.00E+000	0.00E+000	#DIV/0!	FALSE	FALSE	FALSE	'OROTIDINE5PHOSPHATE_c + PROTON_c -> UMP_c + CARBONDIOXIDE_c '
'GLYCEROLKINRXN'	5.10E-001	5.10E-001	5.10E-001	0.004074449	FALSE	FALSE	FALSE	'ATP_c + GLYCEROL_c -> GLYCEROL3P_c + ADP_c + PROTON_c '
'A6__46__3__46__5__46__7RXN'	0.00E+000	0.00E+000	4.16E-005	#DIV/0!	FALSE	FALSE	FALSE	'WATER_c + GLN_c + LglutamyltRNAGln_c + ATP_c -> GLT_c + ADP_c + PROTON_c + ChargedGLNtRNAs_c + Pi_c '
'RXN12460'	-1.27E-002	-1.27E-002	-1.27E-002	-0.166355363	FALSE	FALSE	FALSE	'WATER_c + ChargedASNtRNAs_c <=> 2 PROTON_c + ASNtRNAs_c + ASN_c '
'RXN9386'	0.00E+000	0.00E+000	4.16E-005	#DIV/0!	FALSE	FALSE	FALSE	'GLNtRNAs_c + GLT_c + PROTON_c + ATP_c -> LglutamyltRNAGln_c + PPI_c + AMP_c
'ASPARTATETRNALIGASERXN'	1.15E-002	1.15E-002	1.15E-002	0.004673543	FALSE	FALSE	FALSE	'PROTON_c + ATP_c + LASPARTATE_c + ASPtRNAs_c -> PPI_c + AMP_c + ChargedASPtRNAs_c '
'GLUTAMINETRNALIGASERXN'	1.08E-002	1.08E-002	1.08E-002	0.386915571	FALSE	FALSE	FALSE	'GLN_c + GLNtRNAs_c + PROTON_c + ATP_c -> ChargedGLNtRNAs_c + PPI_c + AMP_c '
'TYROSINETRNALIGASERXN'	6.37E-003	6.37E-003	6.37E-003	0.00327938	FALSE	FALSE	FALSE	'TYR_c + PROTON_c + ATP_c + TYRtRNAs_c -> ChargedTYRtRNAs_c + PPI_c + AMP_c '
'GLYCINETRNALIGASERXN'	1.34E-002	1.34E-002	1.34E-002	0.003278922	FALSE	FALSE	FALSE	'GLY_c + GLYtRNAs_c + PROTON_c + ATP_c -> ChargedGLYtRNAs_c + PPI_c + AMP_c
'ISOLEUCINETRNALIGASERXN'	1.40E-002	1.40E-002	1.40E-002	0.005097958	FALSE	FALSE	FALSE	'ILEtRNAs_c + PROTON_c + ATP_c + ILE_c -> ChargedILEtRNAs_c + PPI_c + AMP_c
'ARGININETRNALIGASERXN'	8.28E-003	8.28E-003	8.28E-003	0.003277901	FALSE	FALSE	FALSE	'ARGtRNAs_c + PROTON_c + ATP_c + ARG_c -> ChargedARGtRNAs_c + PPI_c + AMP_c '
'VALINETRNALIGASERXN'	1.59E-002	1.59E-002	1.59E-002	0.003277458	FALSE	FALSE	FALSE	'VAL_c + VALtRNAs_c + PROTON_c + ATP_c -> ChargedVALtRNAs_c + PPI_c + AMP_c
'LEUCINETRNALIGASERXN'	1.85E-002	1.85E-002	1.85E-002	0.005800272	FALSE	FALSE	FALSE	'LEUtRNAs_c + PROTON_c + ATP_c + LEU_c -> ChargedLEUtRNAs_c + PPI_c + AMP_c '
'CYSTEINETRNALIGASERXN'	1.91E-003	1.91E-003	1.91E-003	0.00327938	FALSE	FALSE	FALSE	'PROTON_c + ATP_c + CYS_c + CYStRNAs_c -> PPI_c + AMP_c + ChargedCYStRNAs_c
'TRYPTOPHANTRNALIGASERXN'	1.91E-003	1.91E-003	1.91E-003	0.00327938	FALSE	FALSE	FALSE	'PROTON_c + ATP_c + TRPtRNAs_c + TRP_c -> ChargedTRPtRNAs_c + PPI_c + AMP_c '
'THREONINETRNALIGASERXN'	1.34E-002	1.34E-002	1.34E-002	0.003278922	FALSE	FALSE	FALSE	'THRtRNAs_c + PROTON_c + ATP_c + THR_c -> ChargedTHRtRNAs_c + PPI_c + AMP_c '
'GLURSRXN'	1.40E-002	1.40E-002	1.40E-002	0.007006892	FALSE	FALSE	FALSE	'GLT_c + PROTON_c + ATP_c + GLTtRNAs_c -> ChargedGLTtRNAs_c + PPI_c + AMP_c '
'ALANINETRNALIGASERXN'	1.72E-002	1.72E-002	1.72E-002	0.003277956	FALSE	FALSE	FALSE	'PROTON_c + ATP_c + LALPHAALANINE_c + ALAtRNAs_c -> ChargedALAtRNAs_c + PPI_c + AMP_c
'LYSINETRNALIGASERXN'	2.04E-002	2.04E-002	2.04E-002	0.004819444	FALSE	FALSE	FALSE	'PROTON_c + ATP_c + LYStRNAs_c + LYS_c -> ChargedLYStRNAs_c + PPI_c + AMP_c
'HISTIDINETRNALIGASERXN'	3.82E-003	3.82E-003	3.82E-003	0.003277778	FALSE	FALSE	FALSE	'HIS_c + PROTON_c + ATP_c + HIStRNAs_c -> ChargedHIStRNAs_c + PPI_c + AMP_c '
'SERINETRNALIGASERXN'	1.34E-002	1.34E-002	1.34E-002	0.003339177	FALSE	FALSE	FALSE	'PROTON_c + SERtRNAs_c + ATP_c + SER_c -> ChargedSERtRNAs_c + PPI_c + AMP_c '
'PHENYLALANINETRNALIGASERXN'	9.56E-003	9.56E-003	9.56E-003	0.004673543	FALSE	FALSE	FALSE	'PROTON_c + ATP_c + PHE_c + PHEtRNAs_c -> ChargedPHEtRNAs_c + PPI_c + AMP_c '
'ASPARAGINETRNALIGASERXN'	0.00E+000	0.00E+000	2.08E-005	#DIV/0!	FALSE	FALSE	FALSE	'PROTON_c + ASNtRNAs_c + ATP_c + ASN_c -> PPI_c + AMP_c + ChargedASNtRNAs_c '
'METHIONINETRNALIGASERXN'	3.19E-003	3.19E-003	3.19E-003	0.011201185	FALSE	FALSE	FALSE	'METtRNAs_c + PROTON_c + ATP_c + MET_c -> PPI_c + AMP_c + ChargedMETtRNAs_c '
'PROLINETRNALIGASERXN'	8.92E-003	8.92E-003	8.92E-003	0.003278694	FALSE	FALSE	FALSE	'PRO_c + PROTON_c + ATP_c + PROtRNAs_c -> ChargedPROtRNAs_c + PPI_c + AMP_c '
'THIOREDOXINREDUCTNADPHRXN'	-9.16E-004	-9.57E-004	-9.16E-004	-4.537494406	FALSE	FALSE	FALSE	'RedThioredoxin_c + NADP_c <=> PROTON_c + NADPH_c + OxThioredoxin_c
'A3__46__1__46__4__46__14RXN'	0.00E+000	0.00E+000	0.00E+000	#DIV/0!	FALSE	FALSE	FALSE	'WATER_c + ACP_c -> apoACP_c + PANTETHEINEP_c '
'RXN01134'	1.08E+001	1.08E+001	1.08E+001	0.000383164	FALSE	FALSE	FALSE	'Pyruvatedehydrogenaselipoate_c + PROTON_c + PYRUVATE_c -> PyruvatedehydrogenaseacetylDHlipoyl_c CARBONDIOXIDE_c '
'RXN01132'	1.08E+001	1.08E+001	1.08E+001	0.000383164	FALSE	FALSE	FALSE	'Pyruvatedehydrogenasedihydrolipoate_c + NAD_c <=> Pyruvatedehydrogenaselipoate_c + PROTON_c + NADH_c '
'GDPREDUCTRXN'	0.00E+000	0.00E+000	4.03E-004	#DIV/0!	TRUE	TRUE	FALSE	'RedThioredoxin_c + GDP_c -> WATER_c + DGDP_c + OxThioredoxin_c
'HOLOACPSYNTHRXN'	0.00E+000	0.00E+000	0.00E+000	#DIV/0!	FALSE	FALSE	FALSE	'COA_c + apoACP_c -> ACP_c + A35ADP_c
'RXN01133'	-1.08E+001	-1.08E+001	-1.08E+001	-0.000383164	FALSE	FALSE	FALSE	'Pyruvatedehydrogenasedihydrolipoate_c + ACETYLCOA_c <=> COA_c + PyruvatedehydrogenaseacetylDHlipoyl_c '
'CDPREDUCTRXN'	0.00E+000	0.00E+000	3.32E-005	#DIV/0!	FALSE	FALSE	FALSE	'RedThioredoxin_c + CDP_c -> WATER_c + DCDP_c + OxThioredoxin_c
'UDPREDUCTRXN'	0.00E+000	0.00E+000	0.00E+000	#DIV/0!	FALSE	FALSE	FALSE	'UDP_c + RedThioredoxin_c -> WATER_c + DUDP_c + OxThioredoxin_c
'ADPREDUCTRXN'	9.16E-004	4.80E-004	9.57E-004	52.16527473	TRUE	FALSE	FALSE	'ADP_c + RedThioredoxin_c -> WATER_c + DADP_c + OxThioredoxin_c
'RXN18KM3'	0.00E+000	0.00E+000	0.00E+000	#DIV/0!	FALSE	FALSE	FALSE	'PHOSPHOENOLPYRUVATE_c + SER_e <=> PYRUVATE_c + A3PSERINE_c '
'TRANSRXN18KM6'	0.00E+000	0.00E+000	0.00E+000	#DIV/0!	FALSE	FALSE	FALSE	'CPD4422_e -> CPD4422_c
'carbonate_co2'	0.00E+000	0.00E+000	0.00E+000	#DIV/0!	FALSE	FALSE	FALSE	'PROTON_c + HCO3_c <=> H2CO3_c '
'transport_alanine'	8.01E-003	8.01E-003	8.01E-003	0.005517349	FALSE	FALSE	FALSE	'WATER_c + ATP_c + LALPHAALANINE_e -> ADP_c + PROTON_c + Pi_c + LALPHAALANINE_c '
'transport_arginine'	3.84E-003	3.84E-003	3.84E-003	0.005517349	FALSE	FALSE	FALSE	'WATER_c + ATP_c + ARG_e -> ADP_c + PROTON_c + Pi_c + ARG_c '
'transport_L-asparagine'	5.77E-003	5.77E-003	5.77E-003	0.005517349	FALSE	FALSE	FALSE	'WATER_c + ATP_c + ASN_e -> ADP_c + PROTON_c + ASN_c + Pi_c '
'transport_L-aspartate'	5.41E-003	5.41E-003	5.41E-003	0.005517349	FALSE	FALSE	FALSE	'WATER_c + ATP_c + LASPARTATE_e -> ADP_c + PROTON_c + LASPARTATE_c + Pi_c '
'transport_L-cysteine'	8.65E-004	8.65E-004	8.65E-004	0.005517349	FALSE	FALSE	FALSE	'WATER_c + ATP_c + CYS_e -> ADP_c + PROTON_c + CYS_c + Pi_c '
'transport_L-glutamate'	6.17E-003	6.14E-003	6.17E-003	0.54424672	FALSE	FALSE	FALSE	'WATER_c + ATP_c + GLT_e -> GLT_c + ADP_c + PROTON_c + Pi_c '
'transport_L-glutamine'	5.53E-003	5.53E-003	5.56E-003	0.601374616	FALSE	FALSE	FALSE	'WATER_c + ATP_c + GLN_e -> GLN_c + ADP_c + PROTON_c + Pi_c '
'transport_L-glycine'	6.26E-003	6.26E-003	6.26E-003	0.005517349	FALSE	FALSE	FALSE	'WATER_c + ATP_c + GLY_e -> GLY_c + ADP_c + PROTON_c + Pi_c '
'transport_L-histidine'	1.79E-003	1.79E-003	1.79E-003	0.005517349	FALSE	FALSE	FALSE	'WATER_c + ATP_c + HIS_e -> HIS_c + ADP_c + PROTON_c + Pi_c '
'transport_L-isoleucine'	6.39E-003	6.39E-003	6.39E-003	0.005517349	FALSE	FALSE	FALSE	'WATER_c + ATP_c + ILE_e -> ADP_c + PROTON_c + Pi_c + ILE_c '
'transport_L-leucine'	8.87E-003	8.87E-003	8.87E-003	0.005517349	FALSE	FALSE	FALSE	'WATER_c + ATP_c + LEU_e -> ADP_c + PROTON_c + Pi_c + LEU_c '
'transport_L-lysine'	9.28E-003	9.27E-003	9.28E-003	0.005517349	FALSE	FALSE	FALSE	'WATER_c + ATP_c + LYS_e -> ADP_c + PROTON_c + Pi_c + LYS_c '
'transport_L-methionine'	1.45E-003	1.45E-003	1.45E-003	0.005517349	FALSE	FALSE	FALSE	'WATER_c + ATP_c + MET_e -> ADP_c + PROTON_c + MET_c + Pi_c '
'transport_L-phenylalanine'	4.45E-003	4.45E-003	4.45E-003	0.005517349	FALSE	FALSE	FALSE	'WATER_c + ATP_c + PHE_e -> ADP_c + PROTON_c + PHE_c + Pi_c '
'transport_L-proline'	4.19E-003	4.19E-003	4.19E-003	0.005517349	FALSE	FALSE	FALSE	'WATER_c + ATP_c + PRO_e -> ADP_c + PRO_c + PROTON_c + Pi_c '
'transport_L-serine'	6.13E-003	6.13E-003	6.13E-003	0.005517349	FALSE	FALSE	FALSE	'WATER_c + ATP_c + SER_e -> ADP_c + PROTON_c + SER_c + Pi_c '
'transport_L-threonine'	6.11E-003	6.11E-003	6.11E-003	0.005517349	FALSE	FALSE	FALSE	'WATER_c + ATP_c + THR_e -> ADP_c + PROTON_c + THR_c + Pi_c '
'transport_L-tryptophan'	9.07E-004	9.07E-004	9.07E-004	0.005517349	FALSE	FALSE	FALSE	'WATER_c + ATP_c + TRP_e -> ADP_c + PROTON_c + Pi_c + TRP_c '
'transport_L-tyrosine'	2.91E-003	2.91E-003	2.91E-003	0.005517349	FALSE	FALSE	FALSE	'WATER_c + ATP_c + TYR_e -> ADP_c + TYR_c + PROTON_c + Pi_c '
'transport_L-valine'	7.27E-003	7.27E-003	7.27E-003	0.005517349	FALSE	FALSE	FALSE	'WATER_c + ATP_c + VAL_e -> VAL_c + ADP_c + PROTON_c + Pi_c '
'transport_guanine'	1.37E-003	1.37E-003	1.37E-003	0.005517349	FALSE	FALSE	FALSE	'PROTON_e + GUANINE_e <=> PROTON_c + GUANINE_c '
'transport_uracil'	0.00E+000	0.00E+000	7.79E-006	#DIV/0!	FALSE	FALSE	FALSE	'PROTON_e + URACIL_e <=> URACIL_c + PROTON_c '
'transport_adenine'	1.99E-003	1.99E-003	1.99E-003	0.005517366	FALSE	FALSE	FALSE	'PROTON_e + ADENINE_e <=> PROTON_c + ADENINE_c '
'transport_thymine'	9.96E-004	9.96E-004	9.96E-004	0.005517349	FALSE	FALSE	FALSE	'PROTON_e + THYMINE_e <=> THYMINE_c + PROTON_c '
'transport_cytidine'	0.00E+000	0.00E+000	4.16E-005	#DIV/0!	FALSE	FALSE	FALSE	'PROTON_e + CYTIDINE_e <=> PROTON_c + CYTIDINE_c '
'transport_deoxycytidine'	1.88E-003	1.84E-003	1.88E-003	2.212931217	FALSE	FALSE	FALSE	'PROTON_e + DEOXYCYTIDINE_e <=> DEOXYCYTIDINE_c + PROTON_c
'transport_phosphate_in'	0.00E+000	0.00E+000	4.16E-005	#DIV/0!	FALSE	FALSE	FALSE	'WATER_c + ATP_c + Pi_e -> ADP_c + PROTON_c + 2 Pi_c '
'transport_acetate'	1.08E+001	1.08E+001	1.08E+001	0.000383164	FALSE	FALSE	FALSE	'ACET_c + PROTON_c -> PROTON_e + ACET_e '
'transport_pyruvate'	0.00E+000	0.00E+000	0.00E+000	#DIV/0!	FALSE	FALSE	FALSE	'PYRUVATE_e -> PYRUVATE_c '
'transport_CARBONDIOXIDE'	1.08E+001	1.08E+001	1.08E+001	0.000383164	FALSE	FALSE	FALSE	'CARBONDIOXIDE_c <=> CARBONDIOXIDE__e '
'transport_WATER'	1.11E+001	1.11E+001	1.11E+001	0.000481976	FALSE	FALSE	FALSE	'WATER_c <=> WATER_e '
'transport_OXYGENMOLECULE'	-1.15E+001	-1.15E+001	-1.15E+001	-0.00036203	FALSE	FALSE	FALSE	'OXYGENMOLECULE_c <=> OXYGENMOLECULE__e
'transport_AMMONIA'	1.48E-003	1.47E-003	1.51E-003	2.773498369	FALSE	FALSE	FALSE	'AMMONIA_c <=> AMMONIA__e
'transport_Riboflavin'	0.00E+000	0.00E+000	0.00E+000	#DIV/0!	FALSE	FALSE	FALSE	'RIBOFLAVIN_e -> RIBOFLAVIN_c '
'transport_H2O2'	6.34E-001	6.34E-001	6.34E-001	0.003279506	FALSE	FALSE	FALSE	'HYDROGENPEROXIDE_c -> HYDROGENPEROXIDE_e '
'transport_FA'	-5.53E-003	-5.53E-003	-5.53E-003	-0.005517349	FALSE	FALSE	FALSE	'LongChainFattyAcids_c <=> LongChainFattyAcids_e
'transport_panthetheine'	0.00E+000	0.00E+000	0.00E+000	#DIV/0!	FALSE	FALSE	FALSE	'PANTETHEINEP_c <=> PANTETHEINEP_e '
'transport_PHOSPHATIDYL_CHOLINE'	-1.66E-003	-1.66E-003	-1.66E-003	-0.005517349	FALSE	FALSE	FALSE	'PHOSPHATIDYL_CHOLINE_c <=> PHOSPHATIDYL_CHOLINE_e '
'transport_MYO_INOSITOL'	0.00E+000	0.00E+000	0.00E+000	#DIV/0!	FALSE	FALSE	FALSE	'MYOINOSITOL_c <=> MYOINOSITOL_e '
'transport_phosphate_out'	1.19E-001	1.19E-001	1.19E-001	0.052521379	FALSE	FALSE	FALSE	'Pi_c -> Pi_e '
'transport_niacine'	0.00E+000	0.00E+000	0.00E+000	#DIV/0!	FALSE	FALSE	FALSE	'NIACINE_e -> NIACINE_c '
'EX_PROTON_e'	1.04E+001	1.04E+001	1.04E+001	0.000519786	FALSE	FALSE	FALSE	'PROTON_e <=> '
'EX_RIBOSE_e'	0.00E+000	0.00E+000	0.00E+000	#DIV/0!	FALSE	FALSE	FALSE	'RIBOSE_e <=> '
'EX_LLACTATE_e'	0.00E+000	0.00E+000	4.16E-005	#DIV/0!	FALSE	FALSE	FALSE	'LLACTATE_e <=> '
'EX_FRU_e'	0.00E+000	0.00E+000	0.00E+000	#DIV/0!	FALSE	FALSE	FALSE	'FRU_e <=> '
'EX_ASCORBATE_e'	0.00E+000	0.00E+000	0.00E+000	#DIV/0!	FALSE	FALSE	FALSE	'ASCORBATE_e <=> '
'EX_MANNOSE_e'	0.00E+000	0.00E+000	0.00E+000	#DIV/0!	FALSE	FALSE	FALSE	'MANNOSE_e <=> '
'EX_CPD4422_e'	0.00E+000	0.00E+000	0.00E+000	#DIV/0!	FALSE	FALSE	FALSE	'CPD4422_e <=> '
'EX_GLYCEROL_e'	-5.10E-001	-5.10E-001	-5.10E-001	-0.004074449	FALSE	FALSE	FALSE	'GLYCEROL_e <=> '
'EX_SER_e'	-6.13E-003	-6.13E-003	-6.13E-003	-0.005517349	FALSE	FALSE	FALSE	'SER_e <=> '
'EX_GLYCEROL3P_e'	-1.28E-001	-1.28E-001	-1.28E-001	-0.01627227	FALSE	FALSE	FALSE	'GLYCEROL3P_e <=> '
'EX_MANNITOL_e'	0.00E+000	0.00E+000	0.00E+000	#DIV/0!	FALSE	FALSE	FALSE	'MANNITOL_e <=> '
'EX_DGlucose_e'	-5.11E+000	-5.11E+000	-5.11E+000	-0.000203324	FALSE	FALSE	FALSE	'DGlucose_e <=>
'EX_LALPHAALANINE_e'	-8.01E-003	-8.01E-003	-8.01E-003	-0.005517349	FALSE	FALSE	FALSE	'LALPHAALANINE_e <=> '
'EX_ARG_e'	-3.84E-003	-3.84E-003	-3.84E-003	-0.005517349	FALSE	FALSE	FALSE	'ARG_e <=> '
'EX_ASN_e'	-5.77E-003	-5.77E-003	-5.77E-003	-0.00551735	FALSE	FALSE	FALSE	'ASN_e <=> '
'EX_LASPARTATE_e'	-5.41E-003	-5.41E-003	-5.41E-003	-0.005517349	FALSE	FALSE	FALSE	'LASPARTATE_e <=> '
'EX_CYS_e'	-8.65E-004	-8.65E-004	-8.65E-004	-0.005517349	FALSE	FALSE	FALSE	'CYS_e <=> '
'EX_GLT_e'	-6.17E-003	-6.17E-003	-6.14E-003	-0.544246721	FALSE	FALSE	FALSE	'GLT_e <=> '
'EX_GLN_e'	-5.53E-003	-5.56E-003	-5.53E-003	-0.601374616	FALSE	FALSE	FALSE	'GLN_e <=> '
'EX_GLY_e'	-6.26E-003	-6.26E-003	-6.26E-003	-0.005517349	FALSE	FALSE	FALSE	'GLY_e <=>
'EX_HIS_e'	-1.79E-003	-1.79E-003	-1.79E-003	-0.005517347	FALSE	FALSE	FALSE	'HIS_e <=> '
'EX_ILE_e'	-6.39E-003	-6.39E-003	-6.39E-003	-0.005517349	FALSE	FALSE	FALSE	'ILE_e <=> '
'EX_LEU_e'	-8.87E-003	-8.87E-003	-8.87E-003	-0.005517349	FALSE	FALSE	FALSE	'LEU_e <=> '
'EX_LYS_e'	-9.28E-003	-9.28E-003	-9.27E-003	-0.005517349	FALSE	FALSE	FALSE	'LYS_e <=>
'EX_MET_e'	-1.45E-003	-1.45E-003	-1.45E-003	-0.005517351	FALSE	FALSE	FALSE	'MET_e <=> '
'EX_PHE_e'	-4.45E-003	-4.45E-003	-4.45E-003	-0.005517349	FALSE	FALSE	FALSE	'PHE_e <=> '
'EX_PRO_e'	-4.19E-003	-4.19E-003	-4.19E-003	-0.005517349	FALSE	FALSE	FALSE	'PRO_e <=> '
'EX_THR_e'	-6.11E-003	-6.11E-003	-6.11E-003	-0.005517348	FALSE	FALSE	FALSE	'THR_e <=> '
'EX_TRP_e'	-9.07E-004	-9.07E-004	-9.07E-004	-0.005517346	FALSE	FALSE	FALSE	'TRP_e <=> '
'EX_TYR_e'	-2.91E-003	-2.91E-003	-2.91E-003	-0.005517349	FALSE	FALSE	FALSE	'TYR_e <=> '
'EX_VAL_e'	-7.27E-003	-7.27E-003	-7.27E-003	-0.005517349	FALSE	FALSE	FALSE	'VAL_e <=> '
'EX_GUANINE_e'	-1.37E-003	-1.37E-003	-1.37E-003	-0.005517353	FALSE	FALSE	FALSE	'GUANINE_e <=> '
'EX_URACIL_e'	0.00E+000	-7.79E-006	0.00E+000	#DIV/0!	FALSE	FALSE	FALSE	'URACIL_e <=> '
'EX_ADENINE_e'	-1.99E-003	-1.99E-003	-1.99E-003	-0.005517366	FALSE	FALSE	FALSE	'ADENINE_e <=> '
'EX_THYMINE_e'	-9.96E-004	-9.96E-004	-9.96E-004	-0.005517349	FALSE	FALSE	FALSE	'THYMINE_e <=> '
'EX_CYTIDINE_e'	0.00E+000	-4.16E-005	0.00E+000	#DIV/0!	FALSE	FALSE	FALSE	'CYTIDINE_e <=> '
'EX_DEOXYCYTIDINE_e'	-1.88E-003	-1.88E-003	-1.84E-003	-2.212931214	FALSE	FALSE	FALSE	'DEOXYCYTIDINE_e <=>
'EX_Pi_e'	1.19E-001	1.19E-001	1.19E-001	0.017507126	FALSE	FALSE	FALSE	'Pi_e <=> '
'EX_ACET_e'	1.08E+001	1.08E+001	1.08E+001	0.000383164	FALSE	FALSE	FALSE	'ACET_e <=> '
'EX_PYRUVATE_e'	0.00E+000	0.00E+000	0.00E+000	#DIV/0!	FALSE	FALSE	FALSE	'PYRUVATE_e <=> '
'EX_CARBONDIOXIDE__e'	1.08E+001	1.08E+001	1.08E+001	0.000383164	FALSE	FALSE	FALSE	'CARBONDIOXIDE__e <=> '
'EX_WATER_e'	1.11E+001	1.11E+001	1.11E+001	0.000481976	FALSE	FALSE	FALSE	'WATER_e <=> '
'EX_OXYGENMOLECULE__e'	-1.15E+001	-1.15E+001	-1.15E+001	-0.00036203	FALSE	FALSE	FALSE	'OXYGENMOLECULE__e <=>
'EX_AMMONIA__e'	1.48E-003	1.47E-003	1.51E-003	2.773498369	FALSE	FALSE	FALSE	'AMMONIA__e <=> '
'EX_RIBOFLAVIN_e'	0.00E+000	0.00E+000	0.00E+000	#DIV/0!	FALSE	FALSE	FALSE	'RIBOFLAVIN_e <=> '
'EX_HYDROGENPEROXIDE_e'	6.34E-001	6.34E-001	6.34E-001	0.003279506	FALSE	FALSE	FALSE	'HYDROGENPEROXIDE_e <=> '

'EX_LongChainFattyAcids_e'	-5.53E-003	-5.53E-003	-5.53E-003	-0.005517349	FALSE	FALSE	FALSE	'LongChainFattyAcids_e <=>
'EX_PANTETHEINEP_e'	0.00E+000	0.00E+000	0.00E+000	#DIV/0!	FALSE	FALSE	FALSE	'PANTETHEINEP_e <=> '
'EX_PHOSPHATIDYL_CHOLINE_e'	-1.66E-003	-1.66E-003	-1.66E-003	-0.005517349	FALSE	FALSE	FALSE	'PHOSPHATIDYL_CHOLINE_e <=> '
'EX_MYOINOSITOL_e'	0.00E+000	0.00E+000	0.00E+000	#DIV/0!	FALSE	FALSE	FALSE	'MYOINOSITOL_e <=> '
'EX_NIACINE_e'	0.00E+000	0.00E+000	0.00E+000	#DIV/0!	FALSE	FALSE	FALSE	'NIACINE_e <=> '
'Protein_synthesis'	6.37E-004	6.37E-004	6.37E-004	0.00327938	FALSE	FALSE	FALSE	'482 WATER_c + 22 ChargedGLTtRNAs_c + 21 ChargedTHRtRNAs_c + 21 ChargedGLYtRNAs_c + 32 ChargedLYStRNAs_c + 21 ChargedSERtRNAs_c + 6 ChargedHIStRNAs_c + 826 ATP_c + 13 ChargedARGtRNAs_c + 17 ChargedGLNtRNAs_c + 3 ChargedTRPtRNAs_c + 29 ChargedLEUtRNAs_c + 25 ChargedVALtRNAs_c + 14 ChargedPROtRNAs_c + 15 ChargedPHEtRNAs_c + 22 ChargedILEtRNAs_c + 10 ChargedTYRtRNAs_c + 27 ChargedALAtRNAs_c + 18 ChargedASPtRNAs_c + 20 ChargedASNtRNAs_c + 5 ChargedMETtRNAs_c + 3 ChargedCYStRNAs_c -> 21 THRtRNAs_c + 17 GLNtRNAs_c + 22 ILEtRNAs_c + 29 LEUtRNAs_c + 826 ADP_c + 21 GLYtRNAs_c + 5 METtRNAs_c + 25 VALtRNAs_c + 13 ARGtRNAs_c + 826 PROTON_c + 20 ASNtRNAs_c + 21 SERtRNAs_c + 10 TYRtRNAs_c + 826 Pi_c + 32 LYStRNAs_c + 18 ASPtRNAs_c + 14 PROtRNAs_c + 27 ALAtRNAs_c + 3 TRPtRNAs_c + 22 GLTtRNAs_c + 3 CYStRNAs_c + 6 HIStRNAs_c + 15 PHEtRNAs_c + PROT_mol_c '
'Protein_synthesis_gram'	1.09E-002	1.09E-002	1.09E-002	0.005517349	FALSE	FALSE	FALSE	'0.0263 PROT_mol_c <=> PROT_g_c '
'ACP_synthesis'	1.87E-006	1.87E-006	1.96E-006	4.784434157	FALSE	FALSE	FALSE	'199 WATER_c + 11 ChargedGLTtRNAs_c + ChargedTHRtRNAs_c + ChargedGLYtRNAs_c + 11 ChargedLYStRNAs_c + 5 ChargedSERtRNAs_c + ChargedHIStRNAs_c + 199 ATP_c + 2 ChargedARGtRNAs_c + 4 ChargedGLNtRNAs_c + 12 ChargedLEUtRNAs_c + 5 ChargedVALtRNAs_c + ChargedPROtRNAs_c + 5 ChargedPHEtRNAs_c + 8 ChargedILEtRNAs_c + 4 ChargedALAtRNAs_c + 6 ChargedASPtRNAs_c + 3 ChargedASNtRNAs_c + 4 ChargedMETtRNAs_c -> THRtRNAs_c + 4 GLNtRNAs_c + 8 ILEtRNAs_c + 12 LEUtRNAs_c + 199 ADP_c + GLYtRNAs_c + 4 METtRNAs_c + 5 VALtRNAs_c + 2 ARGtRNAs_c + 199 PROTON_c + 3 ASNtRNAs_c + 5 SERtRNAs_c + 199 Pi_c + 11 LYStRNAs_c + 6 ASPtRNAs_c + PROtRNAs_c + 4 ALAtRNAs_c + 11 GLTtRNAs_c + HIStRNAs_c + ACP_c + 5 PHEtRNAs_c '
'ACP_synthesis_gram'	1.77E-005	1.77E-005	1.77E-005	0.005517666	FALSE	FALSE	FALSE	'0.10579 ACP_c <=> ACP_g_c '
'Protein_degradation'	3.49E-004	3.49E-004	3.49E-004	0.005985701	FALSE	FALSE	FALSE	'1032 WATER_c + 688 ATP_c + PROT_mol_c -> 25 VAL_c + 17 GLN_c + 21 GLY_c + 22 GLT_c + 6 HIS_c + 688 ADP_c + 14 PRO_c + 10 TYR_c + 688 PROTON_c + 18 LASPARTATE_c + 21 SER_c + 20 ASN_c + 3 CYS_c + 5 MET_c + 21 THR_c + 15 PHE_c + 688 Pi_c + 27 LALPHAALANINE_c + 13 ARG_c + 22 ILE_c + 32 LYS_c + 3 TRP_c + 29 LEU_c '
'ACP_degradation'	0.00E+000	0.00E+000	8.94E-008	#DIV/0!	FALSE	FALSE	FALSE	'219 WATER_c + 146 ATP_c + ACP_c -> 5 VAL_c + 4 GLN_c + GLY_c + 11 GLT_c + HIS_c + 146 ADP_c + PRO_c + 146 PROTON_c + 6 LASPARTATE_c + 5 SER_c + 3 ASN_c + 4 MET_c + THR_c + 5 PHE_c + 146 Pi_c + 4 LALPHAALANINE_c + 2 ARG_c + 8 ILE_c + 11 LYS_c + 12 LEU_c '
'DNA_synthesis'	1.40E-005	1.40E-005	1.40E-005	0.005517349	FALSE	FALSE	FALSE	'71.2 TTP_c + 71.2 DATP_c + 28.8 DGTP_c + 28.8 DCTP_c -> 200 PPI_c + DNA_mmol_c
'DNA_synthesis_gram'	8.83E-004	8.83E-004	8.83E-004	0.005517344	FALSE	FALSE	FALSE	'0.015849 DNA_mmol_c <=> DNA_g_c
'RNA_synthesis'	7.78E-003	7.78E-003	7.78E-003	0.002227073	FALSE	FALSE	FALSE	'18 CTP_c + 29 ATP_c + 28 GTP_c + 25 UTP_c -> 100 PPI_c + RNA_mmol_c
'RNA_synthesis_gram'	1.15E-003	1.15E-003	1.15E-003	0.005517349	FALSE	FALSE	FALSE	'0.029982 RNA_mmol_c <=> RNA_g_c
'RNA_degradation'	7.74E-003	7.74E-003	7.74E-003	0.002236973	FALSE	FALSE	FALSE	'100 WATER_c + RNA_mmol_c -> 18 CMP_c + 100 PROTON_c + 25 UMP_c + 29 AMP_c + 28 GMP_c
'LPHOSPHATIDATE_synthesis_gram'	1.06E-004	1.06E-004	1.06E-004	0.005517349	FALSE	FALSE	FALSE	'1.389 LPHOSPHATIDATE_c <=> LPHOSPHATIDATE_g_c '
'CARDIOLIPIN_synthesis_gram'	1.77E-003	1.77E-003	1.77E-003	0.005517351	FALSE	FALSE	FALSE	'0.74195 CARDIOLIPIN_c <=> CARDIOLIPIN_g_c '
'PHOSPHATIDYL_CHOLINE_gram'	0.00E+000	0.00E+000	0.00E+000	#DIV/0!	FALSE	FALSE	FALSE	'1.3661 PHOSPHATIDYL_CHOLINE_c <=> PHOSPHATIDYL_CHOLINE_g_c '
'LIPID_synthesis'	3.53E-003	3.53E-003	3.53E-003	0.005517349	FALSE	FALSE	FALSE	'0.47 PHOSPHATIDYL_CHOLINE_c + 0.03 LPHOSPHATIDATE_g_c + 0.5 CARDIOLIPIN_g_c -> LIPIDS_c
'G6P_gram'	0.00E+000	0.00E+000	0.00E+000	#DIV/0!	FALSE	FALSE	FALSE	'1.3661 Dglucose6phosphate_c <=> Dglucose6phosphate_g_c
'AAbiomass_mol'	2.34E-008	2.34E-008	2.34E-008	0.005517349	FALSE	FALSE	FALSE	'2793 VAL_c + 294 GLN_c + 9220 GLY_c + 18651 GLT_c + 2422 HIS_c + 6837 PRO_c + 1366 TYR_c + 9318 LASPARTATE_c + 3202 SER_c + 54 ASN_c + 67 CYS_c + 100 MET_c + 2489 THR_c + 5122 PHE_c + 9824 LALPHAALANINE_c + 3913 ARG_c + 1858 ILE_c + 1741 LYS_c + 1864 TRP_c + 21313 LEU_c -> AAbiomass_mol_c '
'AAbiomass_gram'	2.65E-004	2.65E-004	2.65E-004	0.005517366	FALSE	FALSE	FALSE	'8.8228e-05 AAbiomass_mol_c <=> AAbiomass_g_c
'Biomass_synthesis'	1.77E-002	1.77E-002	1.77E-002	0.005517349	FALSE	FALSE	FALSE	'11.53 WATER_c + 11.53 ATP_c + 0.62 PROT_g_c + 0.001 ACP_g_c + 0.05 DNA_g_c + 0.065 RNA_g_c + 0.2 LIPIDS_c + 0.015 AAbiomass_g_c -> 11.53 ADP_c + 11.53 PROTON_c + 11.53 Pi_c + BIOMASS_g_c '
'EX_BIOMASS_g_c'	1.77E-002	1.77E-002	1.77E-002	0.005517349	FALSE	FALSE	FALSE	'BIOMASS_g_c <=> '
'DEOXYGUANOSINEKINASERXN'	4.03E-004	0.00E+000	4.45E-004	110.3069201	TRUE	FALSE	FALSE	'ATP_c + DEOXYGUANOSINE_c <=> ADP_c + PROTON_c + DGMP_c '
'DEOXYADENOSINEKINASERXN'	8.04E-005	3.88E-005	5.17E-004	594.2523829	TRUE	FALSE	FALSE	'ATP_c + DEOXYADENOSINE_c <=> ADP_c + PROTON_c + DAMP_c '
'UMPKINASERXN'	1.95E-001	1.95E-001	1.95E-001	0.017049589	FALSE	FALSE	FALSE	'ATP_c + UMP_c <=> UDP_c + ADP_c '
'ACYLCOASYNTHRXN'	5.53E-003	5.53E-003	5.53E-003	0.005517349	FALSE	FALSE	FALSE	'ATP_c + COA_c + LongChainFattyAcids_c <=> PPI_c + AMP_c + LongChainAcylCoAs_c
'NADKINRXN'	-9.16E-004	-9.57E-004	-9.16E-004	-4.537494406	FALSE	FALSE	FALSE	'ATP_c + NAD_c <=> ADP_c + PROTON_c + NADP_c '
'NADHKINRXN'	9.16E-004	9.16E-004	9.57E-004	4.537494406	FALSE	FALSE	FALSE	'ATP_c + NADH_c <=> ADP_c + PROTON_c + NADPH_c '
				Reactions that did not carry flux can be used		4	3	
				Reactions for which flux varies	20			
