# Supplementary material for: Metabolic modeling of energy balances in Mycoplasma hyopneumoniae shows that pyruvate addition increases growth rate
Source: Biotechnol Bioeng. 2017 Jul 27;114(10):2339–47. doi: 10.1002/bit.26347 (PMC6084303; doi:10.1002/bit.26347)
Supplement: Supplementary file 8 — Table S7. Gene essentiality analysis using the metabolic model. Highlighted genes were found to be essential with a biomass equation containing NADPH, FAD, and CoA [file BIT-114-2339-s008.docx]

| Tabel S7: gene essentiality analysis using the metabolic model. Highlighted genes were found to be essential with a biomass equation containing NADPH, FAD and CoA | | | | | | | | |
| --- | --- | --- | --- | --- | --- | --- | --- | --- |
|  |  |  |  |  |  |  |  |  |
| **No** | **Perc** | **Gene** | **Growth rate** | **Rel. GR** | **Phenotype** | **Ratio** | **Reaction** | **Reaction formula** |
| 1 | 1 | G18KM-1152 | 0.018 | 100 | Growth | 1 | 'A3__46__6__46__5__46__3RXN' | 'WATER_c + GTP_c -> PROTON_c + Pi_c + GDP_c ' |
| 2 | 1 | G18KM-1167 | 0.018 | 100 | Growth | 1 | 'RXN18KM18' | 'WATER_c + PRPP_c + ATP_c + NIACINE_c -> ADP_c + Pi_c + PPI_c + NICOTINATE_NUCLEOTIDE_c ' |
| 3 | 2 | G18KM-707 | 0 | 0 | No growth | 0 | 'URACILPRIBOSYLTRANSRXN' | 'UMP_c + PPI_c <=> PRPP_c + URACIL_c ' |
| 4 | 3 | G18KM-1248 | 0.018 | 100 | Growth | 1 | 'TRANSRXN168' | 'PHOSPHOENOLPYRUVATE_c + MANNOSE_e -> MANNOSE6P_c + PYRUVATE_c ' |
|  |  | G18KM-1248 |  |  |  |  | 'RXN18KM2' | 'FRU_e + PHOSPHOENOLPYRUVATE_c -> FRU1P_c + PYRUVATE_c ' |
| 5 | 4 | G18KM-1142 | 0.018 | 100 | Growth | 1 | 'DCTPPYROPHOSPHATASERXN' | 'WATER_c + DCTP_c -> PROTON_c + DCMP_c + PPI_c ' |
|  |  | G18KM-1142 |  |  |  |  | 'DUTPPYROPRXN' | 'WATER_c + DUTP_c -> PROTON_c + PPI_c + DUMP_c ' |
| 6 | 4 | G18KM-1015 | 0.018 | 100 | Growth | 1 | 'RXN2902' | 'COA_c + NAD_c + MALONATESALD_c -> ACETYLCOA_c + NADH_c + CARBONDIOXIDE_c ' |
|  |  | G18KM-1015 |  |  |  |  | 'A5DEHYDRO2DEOXYGLUCONOKINASERXN' | 'ATP_c + CPD827_c -> ADP_c + PROTON_c + CPD645_c ' |
| 7 | 5 | G18KM-864 | 0.018 | 100 | Growth | 1 | 'A4__46__1__46__2__46__29RXN' | 'CPD645_c -> DIHYDROXYACETONEPHOSPHATE_c + MALONATESALD_c ' |
|  |  | G18KM-864 |  |  |  |  | 'RXN8631' | 'FRU1P_c -> DIHYDROXYACETONEPHOSPHATE_c + GLYCERALD_c ' |
|  |  | G18KM-864 |  |  |  |  | 'F16ALDOLASERXN' | 'FRUCTOSE16DIPHOSPHATE_c <=> DIHYDROXYACETONEPHOSPHATE_c + GAP_c ' |
| 8 | 6 | G18KM-1114 | NaN | NaN | Infeasible | NaN | 'RXN18KM8' | 'DCDP_c + PHOSPHOENOLPYRUVATE_c + PROTON_c -> DCTP_c + PYRUVATE_c ' |
|  |  | G18KM-1114 |  |  |  |  | 'RXN18KM6' | 'PHOSPHOENOLPYRUVATE_c + PROTON_c + CDP_c -> CTP_c + PYRUVATE_c ' |
|  |  | G18KM-1114 |  |  |  |  | 'RXN18KM10' | 'PHOSPHOENOLPYRUVATE_c + PROTON_c + TDP_c -> TTP_c + PYRUVATE_c ' |
|  |  | G18KM-1114 |  |  |  |  | 'RXN18KM7' | 'UDP_c + PHOSPHOENOLPYRUVATE_c + PROTON_c -> PYRUVATE_c + UTP_c ' |
|  |  | G18KM-1114 |  |  |  |  | 'PEPDEPHOSRXN' | 'ADP_c + PHOSPHOENOLPYRUVATE_c + PROTON_c -> ATP_c + PYRUVATE_c ' |
|  |  | G18KM-1114 |  |  |  |  | 'RXN14207' | 'PHOSPHOENOLPYRUVATE_c + PROTON_c + DGDP_c -> DGTP_c + PYRUVATE_c ' |
|  |  | G18KM-1114 |  |  |  |  | 'RXN14192' | 'PHOSPHOENOLPYRUVATE_c + PROTON_c + DADP_c -> DATP_c + PYRUVATE_c ' |
|  |  | G18KM-1114 |  |  |  |  | 'RXN14117' | 'PHOSPHOENOLPYRUVATE_c + PROTON_c + GDP_c -> GTP_c + PYRUVATE_c ' |
| 9 | 7 | G18KM-1013 | 0.018 | 100 | Growth | 1 | 'MYOINOSITOL2DEHYDROGENASERXN' | 'MYOINOSITOL_c + NAD_c -> PROTON_c + CPD365_c + NADH_c ' |
| 10 | 7 | G18KM-806 | 0.018 | 100 | Growth | 1 | 'RXN05292' | 'WATER_c + DCMP_c -> DEOXYCYTIDINE_c + Pi_c ' |
|  |  | G18KM-806 |  |  |  |  | 'THYMIDYLATE5PHOSPHATASERXN' | 'WATER_c + TMP_c -> THYMIDINE_c + Pi_c ' |
|  |  | G18KM-806 |  |  |  |  | 'RXN14143' | 'WATER_c + DUMP_c -> Pi_c + DEOXYURIDINE_c ' |
|  |  | G18KM-806 |  |  |  |  | 'AMPDEPHOSPHORYLATIONRXN' | 'WATER_c + AMP_c -> ADENOSINE_c + Pi_c ' |
|  |  | G18KM-806 |  |  |  |  | 'RXN14142' | 'WATER_c + DGMP_c -> Pi_c + DEOXYGUANOSINE_c ' |
|  |  | G18KM-806 |  |  |  |  | 'RXN7609' | 'WATER_c + GMP_c -> Pi_c + GUANOSINE_c ' |
|  |  | G18KM-806 |  |  |  |  | 'RXN14025' | 'WATER_c + UMP_c -> URIDINE_c + Pi_c ' |
|  |  | G18KM-806 |  |  |  |  | 'RXN14026' | 'WATER_c + CMP_c -> Pi_c + CYTIDINE_c ' |
| 11 | 8 | G18KM-1232 | 0.018 | 100 | Growth | 1 | 'A2__46__7__46__7__46__15RXN' | 'CTP_c + PHOSPHORYLCHOLINE_c + PROTON_c -> CDPCHOLINE_c + PPI_c ' |
|  |  | G18KM-1232 |  |  |  |  | 'PANTEPADENYLYLTRANRXN' | 'PROTON_c + ATP_c + PANTETHEINEP_c -> DEPHOSPHOCOA_c + PPI_c ' |
|  |  | G18KM-1232 |  |  |  |  | 'NICONUCADENYLYLTRANRXN' | 'PROTON_c + ATP_c + NICOTINATE_NUCLEOTIDE_c <=> PPI_c + DEAMIDONAD_c ' |
| 12 | 9 | G18KM-1210 | 0.018 | 100 | Growth | 1 | 'RXN05375' | '2 PROTON_c + CPD01147_c + 2 NADH_c <=> ANTHRANILATE_c + CPD01148_c + 2 NAD_c ' |
|  |  | G18KM-1210 |  |  |  |  | 'A3__46__1__46__4__46__14RXN' | 'WATER_c + ACP_c -> apoACP_c + PANTETHEINEP_c ' |
| 13 | 10 | G18KM-914 | 0.018 | 100 | Growth | 1 | 'GTPCYCLOHYDROIRXN' | 'WATER_c + GTP_c <=> PROTON_c + DIHYDRONEOPTERINP3_c + FORMATE_c ' |
| 14 | 10 | G18KM-752 | 0.018 | 100 | Growth | 1 | 'CHOLINEKINASERXN' | 'ATP_c + CHOLINE_c -> ADP_c + PHOSPHORYLCHOLINE_c + PROTON_c ' |
|  |  | G18KM-752 |  |  |  |  | 'ETHANOLAMINEKINASERXN' | 'ATP_c + ETHANOLAMINE_c <=> PHOSPHORYLETHANOLAMINE_c + ADP_c + PROTON_c ' |
| 15 | 11 | G18KM-892 | 0.018 | 100 | Growth | 1 | 'CHOLINEKINASERXN' | 'ATP_c + CHOLINE_c -> ADP_c + PHOSPHORYLCHOLINE_c + PROTON_c ' |
|  |  | G18KM-892 |  |  |  |  | 'ETHANOLAMINEKINASERXN' | 'ATP_c + ETHANOLAMINE_c <=> PHOSPHORYLETHANOLAMINE_c + ADP_c + PROTON_c ' |
| 16 | 12 | G18KM-900 | 0.018 | 100 | Growth | 1 | 'ATPSYNRXN' | 'WATER_c + 3 PROTON_c + ATP_c <=> 4 PROTON_e + ADP_c + Pi_c ' |
| 17 | 13 | G18KM-899 | 0.018 | 100 | Growth | 1 | 'ATPSYNRXN' | 'WATER_c + 3 PROTON_c + ATP_c <=> 4 PROTON_e + ADP_c + Pi_c ' |
| 18 | 13 | G18KM-1234 | 0.018 | 100 | Growth | 1 | 'ATPSYNRXN' | 'WATER_c + 3 PROTON_c + ATP_c <=> 4 PROTON_e + ADP_c + Pi_c ' |
| 19 | 14 | G18KM-1235 | 0.018 | 100 | Growth | 1 | 'ATPSYNRXN' | 'WATER_c + 3 PROTON_c + ATP_c <=> 4 PROTON_e + ADP_c + Pi_c ' |
| 20 | 15 | G18KM-901 | 0.018 | 100 | Growth | 1 | 'ATPSYNRXN' | 'WATER_c + 3 PROTON_c + ATP_c <=> 4 PROTON_e + ADP_c + Pi_c ' |
| 21 | 16 | G18KM-902 | 0.018 | 100 | Growth | 1 | 'ATPSYNRXN' | 'WATER_c + 3 PROTON_c + ATP_c <=> 4 PROTON_e + ADP_c + Pi_c ' |
| 22 | 16 | G18KM-903 | 0.018 | 100 | Growth | 1 | 'ATPSYNRXN' | 'WATER_c + 3 PROTON_c + ATP_c <=> 4 PROTON_e + ADP_c + Pi_c ' |
| 23 | 17 | G18KM-904 | 0.018 | 100 | Growth | 1 | 'ATPSYNRXN' | 'WATER_c + 3 PROTON_c + ATP_c <=> 4 PROTON_e + ADP_c + Pi_c ' |
| 24 | 18 | G18KM-905 | 0.018 | 100 | Growth | 1 | 'ATPSYNRXN' | 'WATER_c + 3 PROTON_c + ATP_c <=> 4 PROTON_e + ADP_c + Pi_c ' |
| 25 | 19 | G18KM-906 | 0.018 | 100 | Growth | 1 | 'ATPSYNRXN' | 'WATER_c + 3 PROTON_c + ATP_c <=> 4 PROTON_e + ADP_c + Pi_c ' |
| 26 | 19 | G18KM-886 | NaN | NaN | Infeasible | NaN | 'GAPOXNPHOSPHNRXN' | 'Pi_c + GAP_c + NAD_c <=> DPG_c + PROTON_c + NADH_c ' |
| 27 | 20 | G18KM-1012 | 0.018 | 100 | Growth | 1 | 'R503RXN' | 'WATER_c + CPD15167_c -> PROTON_c + CPD827_c ' |
| 28 | 21 | G18KM-1088 | NaN | NaN | Infeasible | NaN | 'GMKALTRXN' | 'ATP_c + DGMP_c -> ADP_c + DGDP_c ' |
|  |  | G18KM-1088 |  |  |  |  | 'GUANYLKINRXN' | 'ATP_c + GMP_c -> ADP_c + GDP_c ' |
| 29 | 21 | G18KM-750 | 0 | 0 | No growth | 0 | 'LACTOSE6PHOSPHATEISOMERASERXN' | 'CPD1241_c <=> TAGATOSE6PHOSPHATE_c ' |
|  |  | G18KM-750 |  |  |  |  | 'RIB5PISOMRXN' | 'RIBOSE5P_c <=> RIBULOSE5P_c ' |
| 30 | 22 | G18KM-747 | 0.018 | 100 | Growth | 1 | 'GLUCOSAMINE6PDEAMINRXN' | 'WATER_c + DGLUCOSAMINE6P_c -> AMMONIA_c + PROTON_c + FRUCTOSE6P_c ' |
| 31 | 23 | G18KM-1090 | 0.018 | 100 | Growth | 1 | 'RIBULP3EPIMRXN' | 'RIBULOSE5P_c <=> XYLULOSE5PHOSPHATE_c ' |
| 32 | 24 | G18KM-739 | 0.018 | 100 | Growth | 1 | 'RIBULP3EPIMRXN' | 'RIBULOSE5P_c <=> XYLULOSE5PHOSPHATE_c ' |
| 33 | 24 | G18KM-1066 | NaN | NaN | Infeasible | NaN | 'DEOXYADENYLATEKINASERXN' | 'ATP_c + DAMP_c -> ADP_c + DADP_c ' |
|  |  | G18KM-1066 |  |  |  |  | 'ADENYLKINRXN' | 'ATP_c + AMP_c -> 2 ADP_c ' |
| 34 | 25 | G18KM-1156 | 0.018 | 99.936 | Growth | 0.9993587 | 'RXN05199' | 'Pi_c + GUANOSINE_c <=> RIBOSE1P_c + GUANINE_c ' |
|  |  | G18KM-1156 |  |  |  |  | 'DEOXYGUANPHOSPHORRXN' | 'Pi_c + DEOXYGUANOSINE_c <=> DEOXYDRIBOSE1PHOSPHATE_c + GUANINE_c ' |
|  |  | G18KM-1156 |  |  |  |  | 'ADENPHOSPHORRXN' | 'ADENOSINE_c + Pi_c <=> RIBOSE1P_c + ADENINE_c ' |
|  |  | G18KM-1156 |  |  |  |  | 'DEOXYADENPHOSPHORRXN' | 'Pi_c + DEOXYADENOSINE_c <=> DEOXYDRIBOSE1PHOSPHATE_c + ADENINE_c ' |
| 35 | 26 | G18KM-1188 | 0 | 0 | No growth | 0 | 'A1TRANSKETORXN' | 'GAP_c + DSEDOHEPTULOSE7P_c <=> XYLULOSE5PHOSPHATE_c + RIBOSE5P_c ' |
|  |  | G18KM-1188 |  |  |  |  | 'A2TRANSKETORXN' | 'XYLULOSE5PHOSPHATE_c + ERYTHROSE4P_c <=> GAP_c + FRUCTOSE6P_c ' |
| 36 | 27 | G18KM-807 | 0 | 0 | No growth | 0 | 'RXN1623' | 'ACYLSNGLYCEROL3P_c + LongChainAcylCoAs_c -> LPHOSPHATIDATE_c + COA_c ' |
| 37 | 27 | G18KM-890 | 0 | 0 | No growth | 0 | 'RXN8141' | 'L1PHOSPHATIDYLGLYCEROL_c + CDPDIACYLGLYCEROL_c <=> CMP_c + CARDIOLIPIN_c + PROTON_c ' |
| 38 | 29 | G18KM-1198 | 0.018 | 100 | Growth | 1 | 'RXN0705' | 'PROTON_c + CPD2343_c -> CARBONDIOXIDE_c + LXYLULOSE5P_c ' |
|  |  | G18KM-1198 |  |  |  |  | 'OROTPDECARBRXN' | 'OROTIDINE5PHOSPHATE_c + PROTON_c -> UMP_c + CARBONDIOXIDE_c ' |
| 39 | 30 | G18KM-1158 | NaN | NaN | Infeasible | NaN | 'NADHDEHYDROGENASERXN' | '2 PROTON_c + 2 NADH_c + OXYGENMOLECULE_c <=> 2 WATER_c + 2 NAD_c ' |
| 40 | 30 | G18KM-1196 | 0.018 | 100 | Growth | 1 | 'RIBULPEPIMRXN' | 'LRIBULOSE5P_c <=> XYLULOSE5PHOSPHATE_c ' |
| 41 | 31 | G18KM-1016 | 0.018 | 100 | Growth | 1 | 'A1__46__2__46__1__46__27RXN' | 'WATER_c + COA_c + CPD12179_c + NAD_c <=> PROTON_c + PROPIONYLCOA_c + NADH_c + HCO3_c ' |
| 42 | 32 | G18KM-1246 | NaN | NaN | Infeasible | NaN | 'PHOSPHOGLYCERATEKINASEGTPRXN' | 'GTP_c + G3P_c <=> DPG_c + GDP_c ' |
|  |  | G18KM-1246 |  |  |  |  | 'RXN18KM13' | 'DATP_c + G3P_c <=> DPG_c + DADP_c ' |
|  |  | G18KM-1246 |  |  |  |  | 'RXN18KM15' | 'DGTP_c + G3P_c <=> DPG_c + DGDP_c ' |
|  |  | G18KM-1246 |  |  |  |  | 'PHOSGLYPHOSRXN' | 'ATP_c + G3P_c <=> DPG_c + ADP_c ' |
| 43 | 33 | G18KM-1014 | 0.018 | 100 | Growth | 1 | 'A5__46__3__46__1__46__17RXN' | 'CPD37_c <=> CPD343_c ' |
|  |  | G18KM-1014 |  |  |  |  | 'RXN14150' | 'CPD827_c <=> CPD15127_c ' |
| 44 | 33 | G18KM-1302 | 0.018 | 100 | Growth | 1 | 'RXN8654' | 'PROTON_c + ATP_c + LIPOICACID_c -> LIPOYLAMP_c + PPI_c ' |
| 45 | 34 | G18KM-988 | 0.018 | 100 | Growth | 1 | 'RXN8654' | 'PROTON_c + ATP_c + LIPOICACID_c -> LIPOYLAMP_c + PPI_c ' |
| 46 | 35 | G18KM-1367 | 0.018 | 100 | Growth | 1 | 'RXN02461' | 'PHOSPHOENOLPYRUVATE_c + ASCORBATE_e -> LASCORBATE6PHOSPHATE_c + PYRUVATE_c ' |
| 47 | 36 | G18KM-1368 | 0.018 | 100 | Growth | 1 | 'RXN02461' | 'PHOSPHOENOLPYRUVATE_c + ASCORBATE_e -> LASCORBATE6PHOSPHATE_c + PYRUVATE_c ' |
| 48 | 36 | G18KM-1369 | 0.018 | 100 | Growth | 1 | 'RXN02461' | 'PHOSPHOENOLPYRUVATE_c + ASCORBATE_e -> LASCORBATE6PHOSPHATE_c + PYRUVATE_c ' |
| 49 | 37 | G18KM-964 | 0.018 | 100 | Growth | 1 | 'RIBOFLAVINKINRXN' | 'RIBOFLAVIN_c + ATP_c -> FMN_c + ADP_c + PROTON_c ' |
|  |  | G18KM-964 |  |  |  |  | 'FADSYNRXN' | 'FMN_c + PROTON_c + ATP_c -> PPI_c + FAD_c ' |
| 50 | 38 | G18KM-1370 | 0.018 | 100 | Growth | 1 | 'RXN05214' | 'WATER_c + LASCORBATE6PHOSPHATE_c <=> CPD2343_c ' |
| 51 | 39 | G18KM-980 | 0 | 0 | No growth | 0 | 'GUANPRIBOSYLTRANRXN' | 'PPI_c + GMP_c <=> PRPP_c + GUANINE_c ' |
| 52 | 39 | G18KM-1296 | 0.018 | 100 | Growth | 1 | 'A3__46__1__46__4__46__2RXN' | 'WATER_c + L1GLYCEROPHOSPHORYLCHOLINE_c -> GLYCEROL3P_c + PROTON_c + CHOLINE_c ' |
| 53 | 40 | G18KM-1362 | 0.012 | 65.313 | Growth | 0.6531255 | 'A3__46__6__46__3__46__20RXN' | 'WATER_c + ATP_c + GLYCEROL3P_e <=> GLYCEROL3P_c + ADP_c + PROTON_c + Pi_c ' |
| 54 | 41 | G18KM-1363 | 0.012 | 65.313 | Growth | 0.6531255 | 'A3__46__6__46__3__46__20RXN' | 'WATER_c + ATP_c + GLYCEROL3P_e <=> GLYCEROL3P_c + ADP_c + PROTON_c + Pi_c ' |
| 55 | 41 | G18KM-1364 | 0.012 | 65.313 | Growth | 0.6531255 | 'A3__46__6__46__3__46__20RXN' | 'WATER_c + ATP_c + GLYCEROL3P_e <=> GLYCEROL3P_c + ADP_c + PROTON_c + Pi_c ' |
| 56 | 42 | G18KM-730 | 0.018 | 100 | Growth | 1 | 'MANNPDEHYDROGRXN' | 'MANNITOL1P_c + NAD_c <=> PROTON_c + NADH_c + FRUCTOSE6P_c ' |
| 57 | 43 | G18KM-729 | 0.018 | 100 | Growth | 1 | 'TRANSRXN156' | 'PHOSPHOENOLPYRUVATE_c + MANNITOL_e -> MANNITOL1P_c + PYRUVATE_c ' |
| 58 | 44 | G18KM-731 | 0.018 | 100 | Growth | 1 | 'TRANSRXN156' | 'PHOSPHOENOLPYRUVATE_c + MANNITOL_e -> MANNITOL1P_c + PYRUVATE_c ' |
| 59 | 44 | G18KM-1264 | NaN | NaN | Infeasible | NaN | 'PHOSACETYLTRANSRXN' | 'ACETYLCOA_c + Pi_c <=> COA_c + ACETYLP_c ' |
| 60 | 45 | G18KM-1263 | NaN | NaN | Infeasible | NaN | 'ACETATEKINRXN' | 'ACET_c + ATP_c <=> ADP_c + ACETYLP_c ' |
| 61 | 46 | G18KM-1008 | 0.018 | 100 | Growth | 1 | 'A3__46__6__46__3__46__17RXN' | 'WATER_c + RIBOSE_e + ATP_c <=> RIBOSE_c + ADP_c + PROTON_c + Pi_c ' |
| 62 | 47 | G18KM-1009 | 0.018 | 100 | Growth | 1 | 'A3__46__6__46__3__46__17RXN' | 'WATER_c + RIBOSE_e + ATP_c <=> RIBOSE_c + ADP_c + PROTON_c + Pi_c ' |
| 63 | 47 | G18KM-1010 | 0.018 | 100 | Growth | 1 | 'A3__46__6__46__3__46__17RXN' | 'WATER_c + RIBOSE_e + ATP_c <=> RIBOSE_c + ADP_c + PROTON_c + Pi_c ' |
| 64 | 48 | G18KM-1352 | NaN | NaN | Infeasible | NaN | 'TRANSRXN131' | 'GLYCEROL_e -> GLYCEROL_c ' |
| 65 | 49 | G18KM-1197 | 0.018 | 100 | Growth | 1 | 'LXULRU5PRXN' | 'LRIBULOSE5P_c <=> LXYLULOSE5P_c ' |
| 66 | 50 | G18KM-762 | NaN | NaN | Infeasible | NaN | 'GLYCEROL3PHOSPHATEOXIDASERXN' | 'GLYCEROL3P_c + OXYGENMOLECULE_c <=> HYDROGENPEROXIDE_c + DIHYDROXYACETONEPHOSPHATE_c ' |
| 67 | 50 | G18KM-786 | NaN | NaN | Infeasible | NaN | 'RXN3715' | 'PHOSPHOENOLPYRUVATE_c + DGlucose_e -> Dglucose6phosphate_c + PYRUVATE_c ' |
| 68 | 51 | G18KM-923 | NaN | NaN | Infeasible | NaN | 'RXN11832' | 'CMP_c + ATP_c <=> ADP_c + CDP_c ' |
|  |  | G18KM-923 |  |  |  |  | 'RXN7913' | 'ATP_c + DCMP_c <=> DCDP_c + ADP_c ' |
| 69 | 52 | G18KM-745 | 0.018 | 100 | Growth | 1 | 'RXN8631' | 'FRU1P_c -> DIHYDROXYACETONEPHOSPHATE_c + GLYCERALD_c ' |
|  |  | G18KM-745 |  |  |  |  | 'F16ALDOLASERXN' | 'FRUCTOSE16DIPHOSPHATE_c <=> DIHYDROXYACETONEPHOSPHATE_c + GAP_c ' |
| 70 | 53 | G18KM-828 | 0 | 0 | No growth | 0 | 'PRPPSYNRXN' | 'ATP_c + RIBOSE5P_c -> PRPP_c + PROTON_c + AMP_c ' |
| 71 | 53 | G18KM-784 | 0 | 0 | No growth | 0 | 'DURIDKIRXN' | 'ATP_c + DEOXYURIDINE_c -> ADP_c + PROTON_c + DUMP_c ' |
|  |  | G18KM-784 |  |  |  |  | 'THYKIRXN' | 'ATP_c + THYMIDINE_c -> ADP_c + PROTON_c + TMP_c ' |
| 72 | 54 | G18KM-779 | NaN | NaN | Infeasible | NaN | 'INORGPYROPHOSPHATRXN' | 'WATER_c + PPI_c -> PROTON_c + 2 Pi_c ' |
| 73 | 55 | G18KM-770 | NaN | NaN | Infeasible | NaN | 'A3PGAREARRRXN' | 'G3P_c <=> A2PG_c ' |
| 74 | 56 | G18KM-744 | 0.018 | 100 | Growth | 1 | 'NAG6PDEACETRXN' | 'WATER_c + NACETYLDGLUCOSAMINE6P_c -> ACET_c + DGLUCOSAMINE6P_c ' |
| 75 | 56 | G18KM-1344 | 0.018 | 100 | Growth | 1 | 'DEPHOSPHOCOAKINRXN' | 'ATP_c + DEPHOSPHOCOA_c -> ADP_c + PROTON_c + COA_c ' |
| 76 | 57 | G18KM-712 | NaN | NaN | Infeasible | NaN | 'RXN13720' | 'Dglucose6phosphate_c <=> FRUCTOSE6P_c ' |
| 77 | 58 | G18KM-708 | 0.018 | 100 | Growth | 1 | 'DEOXYRIBOSEPALDRXN' | 'DEOXYRIBOSE5P_c -> ACETALD_c + GAP_c ' |
| 78 | 59 | G18KM-1157 | 0 | 0 | No growth | 0 | 'URAPHOSPHRXN' | 'Pi_c + DEOXYURIDINE_c <=> URACIL_c + DEOXYDRIBOSE1PHOSPHATE_c ' |
|  |  | G18KM-1157 |  |  |  |  | 'URPHOSRXN' | 'URIDINE_c + Pi_c <=> URACIL_c + RIBOSE1P_c ' |
|  |  | G18KM-1157 |  |  |  |  | 'THYMPHOSPHRXN' | 'THYMIDINE_c + Pi_c <=> THYMINE_c + DEOXYDRIBOSE1PHOSPHATE_c ' |
| 79 | 59 | G18KM-1135 | NaN | NaN | Infeasible | NaN | 'TRIOSEPISOMERIZATIONRXN' | 'GAP_c <=> DIHYDROXYACETONEPHOSPHATE_c ' |
| 80 | 60 | G18KM-1127 | NaN | NaN | Infeasible | NaN | 'A6PFRUCTPHOSRXN' | 'ATP_c + FRUCTOSE6P_c -> ADP_c + PROTON_c + FRUCTOSE16DIPHOSPHATE_c ' |
| 81 | 61 | G18KM-1124 | 0 | 0 | No growth | 0 | 'ADENPRIBOSYLTRANRXN' | 'PPI_c + AMP_c <=> PRPP_c + ADENINE_c ' |
| 82 | 61 | G18KM-1104 | 0.018 | 100 | Growth | 1 | 'LLACTATEDEHYDROGENASERXN' | 'LLACTATE_c + NAD_c <=> PROTON_c + NADH_c + PYRUVATE_c ' |
| 83 | 62 | G18KM-1084 | 0 | 0 | No growth | 0 | 'CYTIDEAMRXN' | 'WATER_c + DEOXYCYTIDINE_c -> AMMONIA_c + DEOXYURIDINE_c ' |
|  |  | G18KM-1084 |  |  |  |  | 'CYTIDEAM2RXN' | 'WATER_c + CYTIDINE_c -> URIDINE_c + AMMONIA_c ' |
| 84 | 63 | G18KM-1080 | 0.018 | 100 | Growth | 1 | 'DPPENTOMUTRXN' | 'DEOXYDRIBOSE1PHOSPHATE_c <=> DEOXYRIBOSE5P_c ' |
|  |  | G18KM-1080 |  |  |  |  | 'PPENTOMUTRXN' | 'RIBOSE1P_c <=> RIBOSE5P_c ' |
| 85 | 64 | G18KM-1017 | 0.018 | 100 | Growth | 1 | 'GLYOHMETRANSRXN' | 'SER_c + THF_c <=> WATER_c + GLY_c + METHYLENETHF_c ' |
| 86 | 64 | G18KM-1011 | 0.018 | 100 | Growth | 1 | 'MYOINOSOSE2DEHYDRATASERXN' | 'CPD365_c -> WATER_c + CPD15127_c ' |
| 87 | 65 | G18KM-992 | NaN | NaN | Infeasible | NaN | 'A2PGADEHYDRATRXN' | 'A2PG_c <=> WATER_c + PHOSPHOENOLPYRUVATE_c ' |
| 88 | 66 | G18KM-983 | 0 | 0 | No growth | 0 | 'DTMPKIRXN' | 'ATP_c + TMP_c <=> ADP_c + TDP_c ' |
| 89 | 67 | G18KM-962 | 0 | 0 | No growth | 0 | 'CTPSYNRXN' | 'WATER_c + GLN_c + ATP_c + UTP_c <=> CTP_c + GLT_c + ADP_c + 2 PROTON_c + Pi_c ' |
| 90 | 67 | G18KM-961 | 0 | 0 | No growth | 0 | 'PHOSPHAGLYPSYNRXN' | 'GLYCEROL3P_c + CDPDIACYLGLYCEROL_c -> CMP_c + L1PHOSPHATIDYLGLYCEROLP_c + PROTON_c ' |
| 91 | 68 | G18KM-743 | 0.018 | 100 | Growth | 1 | 'DIHYDLIPOXNRXN' | 'DIHYDROLIPOAMIDE_c + NAD_c <=> LIPOAMIDE_c + PROTON_c + NADH_c ' |
| 92 | 69 | G18KM-1262 | NaN | NaN | Infeasible | NaN | 'DIHYDLIPOXNRXN' | 'DIHYDROLIPOAMIDE_c + NAD_c <=> LIPOAMIDE_c + PROTON_c + NADH_c ' |
|  |  | G18KM-1262 |  |  |  |  | 'RXN01132' | 'Pyruvatedehydrogenasedihydrolipoate_c + NAD_c <=> Pyruvatedehydrogenaselipoate_c + PROTON_c + NADH_c ' |
| 93 | 70 | G18KM-1250 | 0.018 | 100 | Growth | 1 | 'MANNPISOMRXN' | 'MANNOSE6P_c <=> FRUCTOSE6P_c ' |
| 94 | 70 | G18KM-1229 | 0.018 | 100 | Growth | 1 | 'NADSYNTHNH3RXN' | 'AMMONIA_c + ATP_c + DEAMIDONAD_c -> PPI_c + NAD_c + AMP_c ' |
| 95 | 71 | G18KM-1206 | 0.018 | 100 | Growth | 1 | 'SADENMETSYNRXN' | 'WATER_c + ATP_c + MET_c -> Pi_c + PPI_c + SADENOSYLMETHIONINE_c ' |
| 96 | 72 | G18KM-1353 | NaN | NaN | Infeasible | NaN | 'GLYCEROLKINRXN' | 'ATP_c + GLYCEROL_c -> GLYCEROL3P_c + ADP_c + PROTON_c ' |
| 97 | 73 | G18KM-880 | 0.018 | 100 | Growth | 1 | 'A6__46__3__46__5__46__7RXN' | 'WATER_c + GLN_c + LglutamyltRNAGln_c + ATP_c -> GLT_c + ADP_c + PROTON_c + ChargedGLNtRNAs_c + Pi_c ' |
| 98 | 74 | G18KM-879 | 0.018 | 100 | Growth | 1 | 'A6__46__3__46__5__46__7RXN' | 'WATER_c + GLN_c + LglutamyltRNAGln_c + ATP_c -> GLT_c + ADP_c + PROTON_c + ChargedGLNtRNAs_c + Pi_c ' |
| 99 | 75 | G18KM-1037 | 0.017 | 96.668 | Growth | 0.9666773 | 'RXN12460' | 'WATER_c + ChargedASNtRNAs_c <=> 2 PROTON_c + ASNtRNAs_c + ASN_c ' |
| 100 | 76 | G18KM-1000 | NaN | NaN | Infeasible | NaN | 'ASPARTATETRNALIGASERXN' | 'PROTON_c + ATP_c + LASPARTATE_c + ASPtRNAs_c -> PPI_c + AMP_c + ChargedASPtRNAs_c ' |
| 101 | 76 | G18KM-1100 | NaN | NaN | Infeasible | NaN | 'GLUTAMINETRNALIGASERXN' | 'GLN_c + GLNtRNAs_c + PROTON_c + ATP_c -> ChargedGLNtRNAs_c + PPI_c + AMP_c ' |
|  |  | G18KM-1100 |  |  |  |  | 'GLURSRXN' | 'GLT_c + PROTON_c + ATP_c + GLTtRNAs_c -> ChargedGLTtRNAs_c + PPI_c + AMP_c ' |
| 102 | 77 | G18KM-916 | NaN | NaN | Infeasible | NaN | 'TYROSINETRNALIGASERXN' | 'TYR_c + PROTON_c + ATP_c + TYRtRNAs_c -> ChargedTYRtRNAs_c + PPI_c + AMP_c ' |
| 103 | 78 | G18KM-911 | NaN | NaN | Infeasible | NaN | 'GLYCINETRNALIGASERXN' | 'GLY_c + GLYtRNAs_c + PROTON_c + ATP_c -> ChargedGLYtRNAs_c + PPI_c + AMP_c ' |
| 104 | 79 | G18KM-883 | NaN | NaN | Infeasible | NaN | 'ISOLEUCINETRNALIGASERXN' | 'ILEtRNAs_c + PROTON_c + ATP_c + ILE_c -> ChargedILEtRNAs_c + PPI_c + AMP_c ' |
| 105 | 79 | G18KM-862 | NaN | NaN | Infeasible | NaN | 'ARGININETRNALIGASERXN' | 'ARGtRNAs_c + PROTON_c + ATP_c + ARG_c -> ChargedARGtRNAs_c + PPI_c + AMP_c ' |
| 106 | 80 | G18KM-845 | NaN | NaN | Infeasible | NaN | 'VALINETRNALIGASERXN' | 'VAL_c + VALtRNAs_c + PROTON_c + ATP_c -> ChargedVALtRNAs_c + PPI_c + AMP_c ' |
| 107 | 81 | G18KM-821 | NaN | NaN | Infeasible | NaN | 'LEUCINETRNALIGASERXN' | 'LEUtRNAs_c + PROTON_c + ATP_c + LEU_c -> ChargedLEUtRNAs_c + PPI_c + AMP_c ' |
| 108 | 81 | G18KM-815 | NaN | NaN | Infeasible | NaN | 'CYSTEINETRNALIGASERXN' | 'PROTON_c + ATP_c + CYS_c + CYStRNAs_c -> PPI_c + AMP_c + ChargedCYStRNAs_c ' |
| 109 | 82 | G18KM-765 | NaN | NaN | Infeasible | NaN | 'TRYPTOPHANTRNALIGASERXN' | 'PROTON_c + ATP_c + TRPtRNAs_c + TRP_c -> ChargedTRPtRNAs_c + PPI_c + AMP_c ' |
| 110 | 83 | G18KM-1283 | 0.018 | 100 | Growth | 1 | 'THREONINETRNALIGASERXN' | 'THRtRNAs_c + PROTON_c + ATP_c + THR_c -> ChargedTHRtRNAs_c + PPI_c + AMP_c ' |
| 111 | 84 | G18KM-764 | 0.018 | 100 | Growth | 1 | 'THREONINETRNALIGASERXN' | 'THRtRNAs_c + PROTON_c + ATP_c + THR_c -> ChargedTHRtRNAs_c + PPI_c + AMP_c ' |
| 112 | 84 | G18KM-1040 | 0.018 | 100 | Growth | 1 | 'ALANINETRNALIGASERXN' | 'PROTON_c + ATP_c + LALPHAALANINE_c + ALAtRNAs_c -> ChargedALAtRNAs_c + PPI_c + AMP_c ' |
| 113 | 85 | G18KM-855 | 0.018 | 100 | Growth | 1 | 'ALANINETRNALIGASERXN' | 'PROTON_c + ATP_c + LALPHAALANINE_c + ALAtRNAs_c -> ChargedALAtRNAs_c + PPI_c + AMP_c ' |
| 114 | 86 | G18KM-856 | 0.018 | 100 | Growth | 1 | 'ALANINETRNALIGASERXN' | 'PROTON_c + ATP_c + LALPHAALANINE_c + ALAtRNAs_c -> ChargedALAtRNAs_c + PPI_c + AMP_c ' |
| 115 | 87 | G18KM-1034 | NaN | NaN | Infeasible | NaN | 'LYSINETRNALIGASERXN' | 'PROTON_c + ATP_c + LYStRNAs_c + LYS_c -> ChargedLYStRNAs_c + PPI_c + AMP_c ' |
| 116 | 87 | G18KM-1001 | NaN | NaN | Infeasible | NaN | 'HISTIDINETRNALIGASERXN' | 'HIS_c + PROTON_c + ATP_c + HIStRNAs_c -> ChargedHIStRNAs_c + PPI_c + AMP_c ' |
| 117 | 88 | G18KM-991 | NaN | NaN | Infeasible | NaN | 'SERINETRNALIGASERXN' | 'PROTON_c + SERtRNAs_c + ATP_c + SER_c -> ChargedSERtRNAs_c + PPI_c + AMP_c ' |
| 118 | 89 | G18KM-968 | 0.018 | 100 | Growth | 1 | 'PHENYLALANINETRNALIGASERXN' | 'PROTON_c + ATP_c + PHE_c + PHEtRNAs_c -> ChargedPHEtRNAs_c + PPI_c + AMP_c ' |
| 119 | 90 | G18KM-969 | 0.018 | 100 | Growth | 1 | 'PHENYLALANINETRNALIGASERXN' | 'PROTON_c + ATP_c + PHE_c + PHEtRNAs_c -> ChargedPHEtRNAs_c + PPI_c + AMP_c ' |
| 120 | 90 | G18KM-1255 | 0.018 | 100 | Growth | 1 | 'ASPARAGINETRNALIGASERXN' | 'PROTON_c + ASNtRNAs_c + ATP_c + ASN_c -> PPI_c + AMP_c + ChargedASNtRNAs_c ' |
| 121 | 91 | G18KM-1174 | 0.018 | 100 | Growth | 1 | 'ASPARAGINETRNALIGASERXN' | 'PROTON_c + ASNtRNAs_c + ATP_c + ASN_c -> PPI_c + AMP_c + ChargedASNtRNAs_c ' |
| 122 | 92 | G18KM-1169 | NaN | NaN | Infeasible | NaN | 'METHIONINETRNALIGASERXN' | 'METtRNAs_c + PROTON_c + ATP_c + MET_c -> PPI_c + AMP_c + ChargedMETtRNAs_c ' |
| 123 | 93 | G18KM-1378 | NaN | NaN | Infeasible | NaN | 'PROLINETRNALIGASERXN' | 'PRO_c + PROTON_c + ATP_c + PROtRNAs_c -> ChargedPROtRNAs_c + PPI_c + AMP_c ' |
| 124 | 93 | G18KM-1139 | 0 | 0 | No growth | 0 | 'THIOREDOXINREDUCTNADPHRXN' | 'RedThioredoxin_c + NADP_c <=> PROTON_c + NADPH_c + OxThioredoxin_c ' |
| 125 | 94 | G18KM-1209 | 0.018 | 100 | Growth | 1 | 'A3__46__1__46__4__46__14RXN' | 'WATER_c + ACP_c -> apoACP_c + PANTETHEINEP_c ' |
| 126 | 95 | G18KM-1122 | NaN | NaN | Infeasible | NaN | 'RXN01134' | 'Pyruvatedehydrogenaselipoate_c + PROTON_c + PYRUVATE_c -> PyruvatedehydrogenaseacetylDHlipoyl_c + CARBONDIOXIDE_c ' |
| 127 | 96 | G18KM-1123 | NaN | NaN | Infeasible | NaN | 'RXN01134' | 'Pyruvatedehydrogenaselipoate_c + PROTON_c + PYRUVATE_c -> PyruvatedehydrogenaseacetylDHlipoyl_c + CARBONDIOXIDE_c ' |
| 128 | 96 | G18KM-1018 | 0 | 0 | No growth | 0 | 'GDPREDUCTRXN' | 'RedThioredoxin_c + GDP_c -> WATER_c + DGDP_c + OxThioredoxin_c ' |
|  |  | G18KM-1018 |  |  |  |  | 'CDPREDUCTRXN' | 'RedThioredoxin_c + CDP_c -> WATER_c + DCDP_c + OxThioredoxin_c ' |
|  |  | G18KM-1018 |  |  |  |  | 'UDPREDUCTRXN' | 'UDP_c + RedThioredoxin_c -> WATER_c + DUDP_c + OxThioredoxin_c ' |
|  |  | G18KM-1018 |  |  |  |  | 'ADPREDUCTRXN' | 'ADP_c + RedThioredoxin_c -> WATER_c + DADP_c + OxThioredoxin_c ' |
| 129 | 97 | G18KM-1020 | 0 | 0 | No growth | 0 | 'GDPREDUCTRXN' | 'RedThioredoxin_c + GDP_c -> WATER_c + DGDP_c + OxThioredoxin_c ' |
|  |  | G18KM-1020 |  |  |  |  | 'CDPREDUCTRXN' | 'RedThioredoxin_c + CDP_c -> WATER_c + DCDP_c + OxThioredoxin_c ' |
|  |  | G18KM-1020 |  |  |  |  | 'UDPREDUCTRXN' | 'UDP_c + RedThioredoxin_c -> WATER_c + DUDP_c + OxThioredoxin_c ' |
|  |  | G18KM-1020 |  |  |  |  | 'ADPREDUCTRXN' | 'ADP_c + RedThioredoxin_c -> WATER_c + DADP_c + OxThioredoxin_c ' |
| 130 | 98 | G18KM-1261 | NaN | NaN | Infeasible | NaN | 'RXN01133' | 'Pyruvatedehydrogenasedihydrolipoate_c + ACETYLCOA_c <=> COA_c + PyruvatedehydrogenaseacetylDHlipoyl_c ' |
| 131 | 99 | G18KM-785 | 0.018 | 100 | Growth | 1 | 'RXN18KM3' | 'PHOSPHOENOLPYRUVATE_c + SER_e <=> PYRUVATE_c + A3PSERINE_c ' |
| 132 | 99 | G18KM-1095 | 0.018 | 100 | Growth | 1 | 'TRANSRXN18KM6' | 'CPD4422_e -> CPD4422_c ' |
| 133 | 100 | G18KM-1096 | 0.018 | 100 | Growth | 1 | 'TRANSRXN18KM6' | 'CPD4422_e -> CPD4422_c ' |
|  |  |  |  |  |  |  |  |  |
|  |  |  |  | Growth | 78 |  |  |  |
|  |  |  |  | No growth | 17 |  |  |  |
|  |  |  |  | Infeasible | 38 |  |  |  |
|  |  |  |  |  | 55 | Essential genes | |  |
|  |  |  |  |  | 60 | Essential genes if NADP, FAD and CoA would have been added to the biomass equation | | |
|  |  |  |  |  | 41.4 | Percentage essential genes | |  |
|  |  |  |  |  | 45.1 | Percentage essential genes with more complex biomass | |  |
|  |  |  |  |  | 156.4 | Number of genes with one or more reactions coupled | |  |
|  |  |  |  |  |  |  |  |  |
|  |  |  |  |  | GPRs | 185 |  |  |
|  |  |  |  |  | Average reactions per gene | 1.39 |  |  |
