# Supplementary material for: Metabolic modeling of energy balances in Mycoplasma hyopneumoniae shows that pyruvate addition increases growth rate
Source: Biotechnol Bioeng. 2017 Jul 27;114(10):2339–47. doi: 10.1002/bit.26347 (PMC6084303; doi:10.1002/bit.26347)
Supplement: Supplementary file 9 — Table S8. Flux distributions for knock‐in strains. [file BIT-114-2339-s009.pdf]

Table S8: Flux distributions for knock-in strains

Reaction	Myo-inositol knock-in	Myo-inositol knock-in (ABC)	Arginine knock-in	Reaction formula
'RXN01061'	18.41	18.41	18.41	'WATER_c + ATP_c <=> ADP_c + PROTON_c + Pi_c '
'A3__46__6__46__5__46__3RXN'	0	0	0	'WATER_c + GTP_c -> PROTON_c + Pi_c + GDP_c '
'RXN18KM18'	0	0	0	'WATER_c + PRPP_c + ATP_c + NIACINE_c -> ADP_c + Pi_c + PPI_c + NICOTINATE_NUCLEOTIDE_c '
'URACILPRIBOSYLTRANSRXN'	-0.003075826	-0.002707493	-0.001970826	'UMP_c + PPI_c <=> PRPP_c + URACIL_c '
'TRANSRXN168'	0	0	0	'PHOSPHOENOLPYRUVATE_c + MANNOSE_e -> MANNOSE6P_c + PYRUVATE_c '
'DCTPPYROPHOSPHATASERXN'	0	0	0	'WATER_c + DCTP_c -> PROTON_c + DCMP_c + PPI_c '
'RXN2902'	0.25	0.25	0	'COA_c + NAD_c + MALONATESALD_c -> ACETYLCOA_c + NADH_c + CARBONDIOXIDE_c '
'DUTPPYROPRXN'	0	0	0	'WATER_c + DUTP_c -> PROTON_c + PPI_c + DUMP_c '
'A4__46__1__46__2__46__29RXN'	0.25	0.25	0	'CPD645_c -> DIHYDROXYACETONEPHOSPHATE_c + MALONATESALD_c '
'RXN18KM8'	0.000837705	0.000737389	0.000536757	'DCDP_c + PHOSPHOENOLPYRUVATE_c + PROTON_c -> DCTP_c + PYRUVATE_c '
'MYOINOSITOL2DEHYDROGENASERXN'	0.25	0.25	0	'MYOINOSITOL_c + NAD_c -> PROTON_c + CPD365_c + NADH_c '
'RXN05292'	0	0	0	'WATER_c + DCMP_c -> DEOXYCYTIDINE_c + Pi_c '
'A2__46__7__46__7__46__15RXN'	0	0	0	'CTP_c + PHOSPHORYLCHOLINE_c + PROTON_c -> CDPCHOLINE_c + PPI_c '
'CDPDIGLYSYNRXN'	0.005446644	0.004794403	0.003489921	'CTP_c + LPHOSPHATIDATE_c + PROTON_c -> CDPDIACYLGLYCEROL_c + PPI_c '
'RXN1381'	0.005752544	0.005063671	0.003685925	'GLYCEROL3P_c + LongChainAcylCoAs_c -> ACYLSNGLYCEROL3P_c + COA_c '
'RXN05375'	0	0	0	'2 PROTON_c + CPD01147_c + 2 NADH_c <=> ANTHRANILATE_c + CPD01148_c + 2 NAD_c '
'NACETYLGLUCOSAMINEKINASERXN'	0	0	0	'ATP_c + NACETYLDGLUCOSAMINE_c -> ADP_c + PROTON_c + NACETYLDGLUCOSAMINE6P_c '
'GTPCYCLOHYDROIRXN'	0	0	0	'WATER_c + GTP_c <=> PROTON_c + DIHYDRONEOPTERINP3_c + FORMATE_c '
'CHOLINEKINASERXN'	0	0	0	'ATP_c + CHOLINE_c -> ADP_c + PHOSPHORYLCHOLINE_c + PROTON_c '
'ATPSYNRXN'	-0.229533766	-0.279217659	-0.128585446	'WATER_c + 3 PROTON_c + ATP_c <=> 4 PROTON_e + ADP_c + Pi_c '
'RXN3523'	0	0	0	'2 CPD318_c -> PROTON_c + ASCORBATE_c + LDEHYDROASCORBATE_c '
'THYMIDYLATE5PHOSPHATASERXN'	0	0	0	'WATER_c + TMP_c -> THYMIDINE_c + Pi_c '
'GAPOXNPHOSPHNRXN'	11.08412922	11.08695184	10.84259709	'Pi_c + GAP_c + NAD_c <=> DPG_c + PROTON_c + NADH_c '
'R503RXN'	0	0	0	'WATER_c + CPD15167_c -> PROTON_c + CPD827_c '
'GMKALTRXN'	0.000837705	0.000737389	0.000536757	'ATP_c + DGMP_c -> ADP_c + DGDP_c '
'RXN05305'	0	0	0	'CPD01108_c <=> RIBOSE_c '
'LACTOSE6PHOSPHATEISOMERASERXN'	0	0	0	'CPD1241_c <=> TAGATOSE6PHOSPHATE_c '
'RXN18KM6'	0.144784644	0.144132403	0.142827921	'PHOSPHOENOLPYRUVATE_c + PROTON_c + CDP_c -> CTP_c + PYRUVATE_c '
'GLUCOSAMINE6PDEAMINRXN'	0	0	0	'WATER_c + DGLUCOSAMINE6P_c -> AMMONIA_c + PROTON_c + FRUCTOSE6P_c '
'RIBULP3EPIMRXN'	-0.006037967	-0.005314915	-0.00386881	'RIBULOSE5P_c <=> XYLULOSE5PHOSPHATE_c '
'DEOXYADENYLATEKINASERXN'	0.000167127	0.000147114	0.000107086	'ATP_c + DAMP_c -> ADP_c + DADP_c '
'RXN11811'	0	0	0	'AMMONIA_c + PROTON_c <=> AMMONIUM_c '
'RXN05199'	0	0	0	'Pi_c + GUANOSINE_c <=> RIBOSE1P_c + GUANINE_c '
'RXN10981'	0	0	0	'Acceptor_c + 2 PROTON_c + ASCORBATE_c -> CPD318_c + DonorH2_c '
'RXN12149'	0	0	0	'Lrhamnose_c <=> LRHAMNOFURANOSE_c '
'A1TRANSKETORXN'	0.003018984	0.002657457	0.001934405	'GAP_c + DSEDOHEPTULOSE7P_c <=> XYLULOSE5PHOSPHATE_c + RIBOSE5P_c '
'PGPPHOSPHARXN'	0.002723322	0.002397201	0.00174496	'WATER_c + L1PHOSPHATIDYLGLYCEROLP_c -> L1PHOSPHATIDYLGLYCEROL_c + Pi_c '
'A2TRANSKETORXN'	-0.003018984	-0.002657457	-0.001934405	'XYLULOSE5PHOSPHATE_c + ERYTHROSE4P_c <=> GAP_c + FRUCTOSE6P_c '
'RXN12440'	0	0	0	'HYDROGENPEROXIDE_c + PROTON_c -> 2 WATER_c + ASCORBATE_c + LDEHYDROASCORBATE_c '
'RXN1623'	0.005752544	0.005063671	0.003685925	'ACYLSNGLYCEROL3P_c + LongChainAcylCoAs_c -> LPHOSPHATIDATE_c + COA_c '
'RXN12754'	0	0	0	'WATER_c + NADH_c -> CPD02472_c '
'DEOXYGUANPHOSPHORRXN'	-0.000837705	-0.000737389	-0.000536757	'Pi_c + DEOXYGUANOSINE_c <=> DEOXYDRIBOSE1PHOSPHATE_c + GUANINE_c '
'DEOXYCYTIDINEKINASERXN'	0.000837705	0.000737389	0.000536757	'DEOXYCYTIDINE_c + ATP_c -> ADP_c + PROTON_c + DCMP_c '
'RXN12753'	0	0	0	'WATER_c + NADH_c -> CPD653_c '
'ADENPHOSPHORRXN'	0	0	0	'ADENOSINE_c + Pi_c <=> RIBOSE1P_c + ADENINE_c '
'RXN12862'	0	0	0	'WATER_c + LDEHYDROASCORBATE_c -> CPD13907_c '
'RXN8141'	0.002723322	0.002397201	0.00174496	'L1PHOSPHATIDYLGLYCEROL_c + CDPDIACYLGLYCEROL_c <=> CMP_c + CARDIOLIPIN_c + PROTON_c '
'PANTEPADENYLYLTRANRXN'	0	0	0	'PROTON_c + ATP_c + PANTETHEINEP_c -> DEPHOSPHOCOA_c + PPI_c '
'TRANSRXN104'	0	0	0	'PROTON_e + LLACTATE_e -> PROTON_c + LLACTATE_c '
'RXN18KM2'	0	0	0	'FRU_e + PHOSPHOENOLPYRUVATE_c -> FRU1P_c + PYRUVATE_c '
'RXN0705'	0	0	0	'PROTON_c + CPD2343_c -> CARBONDIOXIDE_c + LXYLULOSE5P_c '
'NADHDEHYDROGENASERXN'	11.33317728	11.3361139	10.84198714	'2 PROTON_c + 2 NADH_c + OXYGENMOLECULE_c <=> 2 WATER_c + 2 NAD_c '
'RIBULPEPIMRXN'	0	0	0	'LRIBULOSE5P_c <=> XYLULOSE5PHOSPHATE_c '
'A1__46__2__46__1__46__27RXN'	0	0	0	'WATER_c + COA_c + CPD12179_c + NAD_c <=> PROTON_c + PROPIONYLCOA_c + NADH_c + HCO3_c '
'PHOSPHOGLYCERATEKINASEGTPRXN'	5.411084183	5.415515547	-0.218031329	'GTP_c + G3P_c <=> DPG_c + GDP_c '
'RXN12863'	0	0	0	'HYDROGENPEROXIDE_c + CPD13907_c -> 3 PROTON_c + OXALATE_c + LTHREONATE_c '
'RXN12869'	0	0	0	'CPD13907_c -> 3 PROTON_c + CPD13914_c '
'RXN18KM13'	-0.002070994	-0.00182299	-0.001326983	'DATP_c + G3P_c <=> DPG_c + DADP_c '
'RXN12861'	0	0	0	'CPD13907_c -> CPD334_c + PROTON_c '
'RXN14143'	0	0	0	'WATER_c + DUMP_c -> Pi_c + DEOXYURIDINE_c '
'A5__46__3__46__1__46__17RXN'	0	0	0	'CPD37_c <=> CPD343_c '
'RXN8654'	0	0	0	'PROTON_c + ATP_c + LIPOICACID_c -> LIPOYLAMP_c + PPI_c '
'RXN02461'	0	0	0	'PHOSPHOENOLPYRUVATE_c + ASCORBATE_e -> LASCORBATE6PHOSPHATE_c + PYRUVATE_c '
'RIBOFLAVINKINRXN'	0	0	0	'RIBOFLAVIN_c + ATP_c -> FMN_c + ADP_c + PROTON_c '
'ETHANOLAMINEKINASERXN'	0	0	0	'ATP_c + ETHANOLAMINE_c <=> PHOSPHORYLETHANOLAMINE_c + ADP_c + PROTON_c '
'AMPDEPHOSPHORYLATIONRXN'	0	0	0	'WATER_c + AMP_c -> ADENOSINE_c + Pi_c '
'RXN14142'	0	0	0	'WATER_c + DGMP_c -> Pi_c + DEOXYGUANOSINE_c '
'RXN7609'	0	0	0	'WATER_c + GMP_c -> Pi_c + GUANOSINE_c '
'RXN12872'	0	0	0	'CPD13914_c -> CPD13910_c '
'RXN14025'	0	0	0	'WATER_c + UMP_c -> URIDINE_c + Pi_c '
'RXN14026'	0	0	0	'WATER_c + CMP_c -> Pi_c + CYTIDINE_c '
'RXN05214'	0	0	0	'WATER_c + LASCORBATE6PHOSPHATE_c <=> CPD2343_c '
'RXN12870'	0	0	0	'CPD334_c -> CPD13913_c '
'RXN14150'	-0.25	-0.25	0	'CPD827_c <=> CPD15127_c '
'GUANPRIBOSYLTRANRXN'	-0.002002864	-0.001763019	-0.001283329	'PPI_c + GMP_c <=> PRPP_c + GUANINE_c '
'RXN18KM10'	0.002070994	0.00182299	0.001326983	'PHOSPHOENOLPYRUVATE_c + PROTON_c + TDP_c -> TTP_c + PYRUVATE_c '
'RXN12871'	0	0	0	'WATER_c + CPD13913_c -> PROTON_c + CPD13912_c '
'RXN18KM15'	-0.000837705	-0.000737389	5.391872846	'DGTP_c + G3P_c <=> DPG_c + DGDP_c '
'TRANSALDOLRXN'	-0.003018984	-0.002657457	-0.001934405	'GAP_c + DSEDOHEPTULOSE7P_c <=> ERYTHROSE4P_c + FRUCTOSE6P_c '
'A3__46__1__46__4__46__2RXN'	0	0	0	'WATER_c + L1GLYCEROPHOSPHORYLCHOLINE_c -> GLYCEROL3P_c + PROTON_c + CHOLINE_c '
'RIB5PISOMRXN'	-0.006037967	-0.005314915	-0.00386881	'RIBOSE5P_c <=> RIBULOSE5P_c '
'RXN18KM7'	0.196600826	0.196232493	0.195495826	'UDP_c + PHOSPHOENOLPYRUVATE_c + PROTON_c -> PYRUVATE_c + UTP_c '
'A3__46__6__46__3__46__20RXN'	0.1277	0.1277	0.1277	'WATER_c + ATP_c + GLYCEROL3P_e <=> GLYCEROL3P_c + ADP_c + PROTON_c + Pi_c '
'DEOXYADENPHOSPHORRXN'	-0.000167127	-0.000147114	-0.000107086	'Pi_c + DEOXYADENOSINE_c <=> DEOXYDRIBOSE1PHOSPHATE_c + ADENINE_c '
'MANNPDEHYDROGRXN'	0	0	0	'MANNITOL1P_c + NAD_c <=> PROTON_c + NADH_c + FRUCTOSE6P_c '
'TRANSRXN156'	0	0	0	'PHOSPHOENOLPYRUVATE_c + MANNITOL_e -> MANNITOL1P_c + PYRUVATE_c '
'PHOSACETYLTRANSRXN'	11.33412922	11.33695184	10.84259709	'ACETYLCOA_c + Pi_c <=> COA_c + ACETYLP_c '
'ACETATEKINRXN'	-11.33412922	-11.33695184	-10.84259709	'ACET_c + ATP_c <=> ADP_c + ACETYLP_c '
'A3__46__6__46__3__46__17RXN'	0	0	0	'WATER_c + RIBOSE_e + ATP_c <=> RIBOSE_c + ADP_c + PROTON_c + Pi_c '
'NICONUCADENYLYLTRANRXN'	0	0	0	'PROTON_c + ATP_c + NICOTINATE_NUCLEOTIDE_c <=> PPI_c + DEAMIDONAD_c '
'TRANSRXN131'	0.51	0.51	0.51	'GLYCEROL_e -> GLYCEROL_c '
'LXULRU5PRXN'	0	0	0	'LRIBULOSE5P_c <=> LXYLULOSE5P_c '
'GLYCEROL3PHOSPHATEOXIDASERXN'	0.629224134	0.630239128	0.632269114	'GLYCEROL3P_c + OXYGENMOLECULE_c <=> HYDROGENPEROXIDE_c + DIHYDROXYACETONEPHOSPHATE_c '
'RXN3715'	5.11	5.11	5.11	'PHOSPHOENOLPYRUVATE_c + DGlucose_e -> Dglucose6phosphate_c + PYRUVATE_c '
'RXN11832'	0.144784644	0.144132403	0.142827921	'CMP_c + ATP_c <=> ADP_c + CDP_c '
'RXN7913'	0.000837705	0.000737389	0.000536757	'ATP_c + DCMP_c <=> DCDP_c + ADP_c '
'RXN8631'	0	0	0	'FRU1P_c -> DIHYDROXYACETONEPHOSPHATE_c + GLYCERALD_c '
'F16ALDOLASERXN'	5.103962033	5.104685085	5.10613119	'FRUCTOSE16DIPHOSPHATE_c <=> DIHYDROXYACETONEPHOSPHATE_c + GAP_c '
'PRPPSYNRXN'	0.009056951	0.007972372	0.005803214	'ATP_c + RIBOSE5P_c -> PRPP_c + PROTON_c + AMP_c '

'DURIDKIRXN'	0	0	0	'ATP_c + DEOXYURIDINE_c -> ADP_c + PROTON_c + DUMP_c '
'THYKIRXN'	0.002070994	0.00182299	0.001326983	'ATP_c + THYMIDINE_c -> ADP_c + PROTON_c + TMP_c '
'INORGPYROPHOSPHATRXN'	1.120354746	1.092427479	1.036572946	'WATER_c + PPI_c -> PROTON_c + 2 Pi_c '
'A3PGAREARRRXN'	11.08412922	11.08695184	10.84259709	'G3P_c <=> A2PG_c '
'NAG6PDEACETRXN'	0	0	0	'WATER_c + NACETYLDGLUCOSAMINE6P_c -> ACET_c + DGLUCOSAMINE6P_c '
'DEPHOSPHOCOAKINRXN'	0	0	0	'ATP_c + DEPHOSPHOCOA_c -> ADP_c + PROTON_c + COA_c '
'RXN13720'	5.11	5.11	5.11	'Dglucose6phosphate_c <=> FRUCTOSE6P_c '
'DEOXYRIBOSEPALDRXN'	0	0	0	'DEOXYRIBOSE5P_c -> ACETALD_c + GAP_c '
'URAPHOSPHRXN'	0.003075826	0.002707493	0.001970826	'Pi_c + DEOXYURIDINE_c <=> URACIL_c + DEOXYDRIBOSE1PHOSPHATE_c '
'URPHOSRXN'	0	0	0	'URIDINE_c + Pi_c <=> URACIL_c + RIBOSE1P_c '
'THYMPHOSPHRXN'	-0.002070994	-0.00182299	-0.001326983	'THYMIDINE_c + Pi_c <=> THYMINE_c + DEOXYDRIBOSE1PHOSPHATE_c '
'TRIOSEPISOMERIZATIONRXN'	-5.983186167	-5.984924213	-5.738400305	'GAP_c <=> DIHYDROXYACETONEPHOSPHATE_c '
'A6PFRUCTPHOSRXN'	5.103962033	5.104685085	5.10613119	'ATP_c + FRUCTOSE6P_c -> ADP_c + PROTON_c + FRUCTOSE16DIPHOSPHATE_c '
'ADENPRIBOSYLTRANRXN'	-0.003978261	-0.00350186	-0.002549059	'PPI_c + AMP_c <=> PRPP_c + ADENINE_c '
'PEPDEPHOSRXN'	0	0	0	'ADP_c + PHOSPHOENOLPYRUVATE_c + PROTON_c -> ATP_c + PYRUVATE_c '
'RXN14207'	0	0	5.392409603	'PHOSPHOENOLPYRUVATE_c + PROTON_c + DGDP_c -> DGTP_c + PYRUVATE_c '
'RXN14192'	0	0	0	'PHOSPHOENOLPYRUVATE_c + PROTON_c + DADP_c -> DATP_c + PYRUVATE_c '
'RXN14117'	5.629835047	5.634026566	0	'PHOSPHOENOLPYRUVATE_c + PROTON_c + GDP_c -> GTP_c + PYRUVATE_c '
'LLACTATEDEHYDROGENASERXN'	0	0	0	'LLACTATE_c + NAD_c <=> PROTON_c + NADH_c + PYRUVATE_c '
'GUANYLKINRXN'	0.218750864	0.218511019	0.218031329	'ATP_c + GMP_c -> ADP_c + GDP_c '
'CYTIDEAMRXN'	0.003075826	0.002707493	0.001970826	'WATER_c + DEOXYCYTIDINE_c -> AMMONIA_c + DEOXYURIDINE_c '
'CYTIDEAM2RXN'	0	0	0	'WATER_c + CYTIDINE_c -> URIDINE_c + AMMONIA_c '
'DPPENTOMUTRXN'	0	0	0	'DEOXYDRIBOSE1PHOSPHATE_c <=> DEOXYRIBOSE5P_c '
'PPENTOMUTRXN'	0	0	0	'RIBOSE1P_c <=> RIBOSE5P_c '
'ADENYLKINRXN'	0.556304881	0.530106682	0.477710286	'ATP_c + AMP_c -> 2 ADP_c '
'GLYOHMETRANSRXN'	0	0	0	'SER_c + THF_c <=> WATER_c + GLY_c + METHYLENETHF_c '
'A5DEHYDRO2DEOXYGLUCONOKINASERXN'	0.25	0.25	0	'ATP_c + CPD827_c -> ADP_c + PROTON_c + CPD645_c '
'MYOINOSOSE2DEHYDRATASERXN'	0.25	0.25	0	'CPD365_c -> WATER_c + CPD15127_c '
'A2PGADEHYDRATRXN'	11.08412922	11.08695184	10.84259709	'A2PG_c <=> WATER_c + PHOSPHOENOLPYRUVATE_c '
'DTMPKIRXN'	0.002070994	0.00182299	0.001326983	'ATP_c + TMP_c <=> ADP_c + TDP_c '
'FADSYNRXN'	0	0	0	'FMN_c + PROTON_c + ATP_c -> PPI_c + FAD_c '
'CTPSYNRXN'	0.001287555	0.001133369	0.000824997	'WATER_c + GLN_c + ATP_c + UTP_c <=> CTP_c + GLT_c + ADP_c + 2 PROTON_c + Pi_c '
'PHOSPHAGLYPSYNRXN'	0.002723322	0.002397201	0.00174496	'GLYCEROL3P_c + CDPDIACYLGLYCEROL_c -> CMP_c + L1PHOSPHATIDYLGLYCEROLP_c + PROTON_c '
'DIHYDLIPOXNRXN'	0	0	0	'DIHYDROLIPOAMIDE_c + NAD_c <=> LIPOAMIDE_c + PROTON_c + NADH_c '
'MANNPISOMRXN'	0	0	0	'MANNOSE6P_c <=> FRUCTOSE6P_c '
'PHOSGLYPHOSRXN'	-16.4923047	-16.49990701	-16.01511162	'ATP_c + G3P_c <=> DPG_c + ADP_c '
'NADSYNTHNH3RXN'	0	0	0	'AMMONIA_c + ATP_c + DEAMIDONAD_c -> PPI_c + NAD_c + AMP_c '
'SADENMETSYNRXN'	0	0	0	'WATER_c + ATP_c + MET_c -> Pi_c + PPI_c + SADENOSYLMETHIONINE_c '
'OROTPDECARBRXN'	0	0	0	'OROTIDINE5PHOSPHATE_c + PROTON_c -> UMP_c + CARBONDIOXIDE_c '
'GLYCEROLKINRXN'	0.51	0.51	0.51	'ATP_c + GLYCEROL_c -> GLYCEROL3P_c + ADP_c + PROTON_c '
'A6__46__3__46__5__46__7RXN'	0	0	0	'WATER_c + GLN_c + LglutamyltRNAGln_c + ATP_c -> GLT_c + ADP_c + PROTON_c + ChargedGLNtRNAs_c + Pi_c '
'RXN12460'	-0.018959862	-0.017525022	-0.014655343	'WATER_c + ChargedASNtRNAs_c <=> 2 PROTON_c + ASNtRNAs_c + ASN_c '
'RXN9386'	0	0	0	'GLNtRNAs_c + GLT_c + PROTON_c + ATP_c -> LglutamyltRNAGln_c + PPI_c + AMP_c '
'ASPARTATETRNALIGASERXN'	0.01707669	0.0157838	0.013198019	'PROTON_c + ATP_c + LASPARTATE_c + ASPtRNAs_c -> PPI_c + AMP_c + ChargedASPtRNAs_c '
'GLUTAMINETRNALIGASERXN'	0.016121513	0.014901225	0.012460649	'GLN_c + GLNtRNAs_c + PROTON_c + ATP_c -> ChargedGLNtRNAs_c + PPI_c + AMP_c '
'TYROSINETRNALIGASERXN'	0.009474107	0.008757384	0.007323939	'TYR_c + PROTON_c + ATP_c + TYRtRNAs_c -> ChargedTYRtRNAs_c + PPI_c + AMP_c '
'GLYCINETRNALIGASERXN'	0.019899507	0.018393925	0.01538276	'GLY_c + GLYtRNAs_c + PROTON_c + ATP_c -> ChargedGLYtRNAs_c + PPI_c + AMP_c '
'ISOLEUCINETRNALIGASERXN'	0.020874099	0.019293589	0.016132571	'ILEtRNAs_c + PROTON_c + ATP_c + ILE_c -> ChargedILEtRNAs_c + PPI_c + AMP_c '
'ARGININETRNALIGASERXN'	0.012324105	0.011391436	0.009526097	'ARGtRNAs_c + PROTON_c + ATP_c + ARG_c -> ChargedARGtRNAs_c + PPI_c + AMP_c '
'VALINETRNALIGASERXN'	0.023704682	0.021910551	0.018322288	'VAL_c + VALtRNAs_c + PROTON_c + ATP_c -> ChargedVALtRNAs_c + PPI_c + AMP_c '
'LEUCINETRNALIGASERXN'	0.027521506	0.02543743	0.02126928	'LEUtRNAs_c + PROTON_c + ATP_c + LEU_c -> ChargedLEUtRNAs_c + PPI_c + AMP_c '
'CYSTEINETRNALIGASERXN'	0.002842232	0.002627215	0.002197182	'PROTON_c + ATP_c + CYS_c + CYStRNAs_c -> PPI_c + AMP_c + ChargedCYStRNAs_c '
'TRYPTOPHANTRNALIGASERXN'	0.002842232	0.002627215	0.002197182	'PROTON_c + ATP_c + TRPtRNAs_c + TRP_c -> ChargedTRPtRNAs_c + PPI_c + AMP_c '
'THREONINETRNALIGASERXN'	0.019899507	0.018393925	0.01538276	'THRtRNAs_c + PROTON_c + ATP_c + THR_c -> ChargedTHRtRNAs_c + PPI_c + AMP_c '
'GLURSRXN'	0.020885748	0.019303843	0.016140035	'GLT_c + PROTON_c + ATP_c + GLTtRNAs_c -> ChargedGLTtRNAs_c + PPI_c + AMP_c '
'ALANINETRNALIGASERXN'	0.02559562	0.023658609	0.019784588	'PROTON_c + ATP_c + LALPHAALANINE_c + ALAtRNAs_c -> ChargedALAtRNAs_c + PPI_c + AMP_c '
'LYSINETRNALIGASERXN'	0.030359855	0.028061228	0.023463974	'PROTON_c + ATP_c + LYStRNAs_c + LYS_c -> ChargedLYStRNAs_c + PPI_c + AMP_c '
'HISTIDINETRNALIGASERXN'	0.005688347	0.005257849	0.004396852	'HIS_c + PROTON_c + ATP_c + HIStRNAs_c -> ChargedHIStRNAs_c + PPI_c + AMP_c '
'SERINETRNALIGASERXN'	0.019915039	0.018407597	0.015392713	'PROTON_c + SERtRNAs_c + ATP_c + SER_c -> ChargedSERtRNAs_c + PPI_c + AMP_c '
'PHENYLALANINETRNALIGASERXN'	0.014230575	0.013153166	0.010998349	'PROTON_c + ATP_c + PHE_c + PHEtRNAs_c -> ChargedPHEtRNAs_c + PPI_c + AMP_c '
'ASPARAGINETRNALIGASERXN'	0	0	0	'PROTON_c + ASNtRNAs_c + ATP_c + ASN_c -> PPI_c + AMP_c + ChargedASNtRNAs_c '
'METHIONINETRNALIGASERXN'	0.004752585	0.004392364	0.003671922	'METtRNAs_c + PROTON_c + ATP_c + MET_c -> PPI_c + AMP_c + ChargedMETtRNAs_c '
'PROLINETRNALIGASERXN'	0.013267632	0.012263756	0.010256003	'PRO_c + PROTON_c + ATP_c + PROtRNAs_c -> ChargedPROtRNAs_c + PPI_c + AMP_c '
'THIOREDOXINREDUCTNADPHRXN'	-0.001903867	-0.001675877	-0.001219897	'RedThioredoxin_c + NADP_c <=> PROTON_c + NADPH_c + OxThioredoxin_c '
'A3__46__1__46__4__46__14RXN'	0	0	0	'WATER_c + ACP_c -> apoACP_c + PANTETHEINEP_c '
'RXN01134'	11.08412922	11.08695184	10.84259709	'Pyruvatedehydrogenaselipoate_c + PROTON_c + PYRUVATE_c -> PyruvatedehydrogenaseacetylDHlipoyl_c + CARBONDIOXIDE_c '
'RXN01132'	11.08412922	11.08695184	10.84259709	'Pyruvatedehydrogenasedihydrolipoate_c + NAD_c <=> Pyruvatedehydrogenaselipoate_c + PROTON_c + NADH_c '
'GDPREDUCTRXN'	0	0	0	'RedThioredoxin_c + GDP_c -> WATER_c + DGDP_c + OxThioredoxin_c '
'HOLOACPSYNTHRXN'	0	0	0	'COA_c + apoACP_c -> ACP_c + A35ADP_c '
'RXN01133'	-11.08412922	-11.08695184	-10.84259709	'Pyruvatedehydrogenasedihydrolipoate_c + ACETYLCOA_c <=> COA_c + PyruvatedehydrogenaseacetylDHlipoyl_c '
'CDPREDUCTRXN'	0	0	0	'RedThioredoxin_c + CDP_c -> WATER_c + DCDP_c + OxThioredoxin_c '
'UDPREDUCTRXN'	0	0	0	'UDP_c + RedThioredoxin_c -> WATER_c + DUDP_c + OxThioredoxin_c '
'ADPREDUCTRXN'	0.001903867	0.001675877	0.001219897	'ADP_c + RedThioredoxin_c -> WATER_c + DADP_c + OxThioredoxin_c '
'RXN18KM3'	0	0	0	'PHOSPHOENOLPYRUVATE_c + SER_e <=> PYRUVATE_c + A3PSERINE_c '
'TRANSRXN18KM6'	0	0	0	'CPD4422_e -> CPD4422_c '
'carbonate_co2'	0	0	0	'PROTON_c + HCO3_c <=> H2CO3_c '
'transport_alanine'	0.016652531	0.014658374	0.010670059	'WATER_c + ATP_c + LALPHAALANINE_e -> ADP_c + PROTON_c + Pi_c + LALPHAALANINE_c '
'transport_arginine'	0.007978483	0.007023051	0.005112189	'WATER_c + ATP_c + ARG_e -> ADP_c + PROTON_c + Pi_c + ARG_c '
'transport_L-asparagine'	0.011984486	0.010549331	0.007679023	'WATER_c + ATP_c + ASN_e -> ADP_c + PROTON_c + ASN_c + Pi_c '
'transport_L-aspartate'	0.011249121	0.009902028	0.007207841	'WATER_c + ATP_c + LASPARTATE_e -> ADP_c + PROTON_c + LASPARTATE_c + Pi_c '
'transport_L-cysteine'	0.001798787	0.00158338	0.001152567	'WATER_c + ATP_c + CYS_e -> ADP_c + PROTON_c + CYS_c + Pi_c '
'transport_L-glutamate'	0.012828384	0.011292172	0.008219749	'WATER_c + ATP_c + GLT_e -> GLT_c + ADP_c + PROTON_c + Pi_c '
'transport_L-glutamine'	0.01149205	0.010115866	0.007363497	'WATER_c + ATP_c + GLN_e -> GLN_c + ADP_c + PROTON_c + Pi_c '
'transport_L-glycine'	0.013020478	0.011461263	0.008342832	'WATER_c + ATP_c + GLY_e -> GLY_c + ADP_c + PROTON_c + Pi_c '
'transport_L-histidine'	0.003712598	0.003268011	0.002378836	'WATER_c + ATP_c + HIS_e -> HIS_c + ADP_c + PROTON_c + Pi_c '
'transport_L-isoleucine'	0.013288553	0.011697236	0.008514601	'WATER_c + ATP_c + ILE_e -> ADP_c + PROTON_c + Pi_c + ILE_c '
'transport_L-leucine'	0.018438706	0.016230653	0.011814546	'WATER_c + ATP_c + LEU_e -> ADP_c + PROTON_c + Pi_c + LEU_c '
'transport_L-lysine'	0.019279625	0.016970871	0.012353362	'WATER_c + ATP_c + LYS_e -> ADP_c + PROTON_c + Pi_c + LYS_c '
'transport_L-methionine'	0.003012943	0.00265214	0.001930534	'WATER_c + ATP_c + MET_e -> ADP_c + PROTON_c + MET_c + Pi_c '
'transport_L-phenylalanine'	0.009245881	0.008138678	0.005924271	'WATER_c + ATP_c + PHE_e -> ADP_c + PROTON_c + PHE_c + Pi_c '
'transport_L-proline'	0.008715147	0.007671499	0.005584204	'WATER_c + ATP_c + PRO_e -> ADP_c + PRO_c + PROTON_c + Pi_c '
'transport_L-serine'	0.012743679	0.011217611	0.008165475	'WATER_c + ATP_c + SER_e -> ADP_c + PROTON_c + SER_c + Pi_c '
'transport_L-threonine'	0.012693513	0.011173452	0.00813333	'WATER_c + ATP_c + THR_e -> ADP_c + PROTON_c + THR_c + Pi_c '
'transport_L-tryptophan'	0.001886078	0.001660218	0.001208499	'WATER_c + ATP_c + TRP_e -> ADP_c + PROTON_c + Pi_c + TRP_c '
'transport_L-tyrosine'	0.006051462	0.005326793	0.003877456	'WATER_c + ATP_c + TYR_e -> ADP_c + TYR_c + PROTON_c + Pi_c '
'transport_L-valine'	0.015117855	0.013307476	0.00968672	'WATER_c + ATP_c + VAL_e -> VAL_c + ADP_c + PROTON_c + Pi_c '
'transport_guanine'	0.002840569	0.002500408	0.001820086	'PROTON_e + GUANINE_e <=> PROTON_c + GUANINE_c '
'transport_uracil'	0	0	0	'PROTON_e + URACIL_e <=> URACIL_c + PROTON_c '
'transport_adenine'	0.004145388	0.003648974	0.002656145	'PROTON_e + ADENINE_e <=> PROTON_c + ADENINE_c '
'transport_thymine'	0.002070994	0.00182299	0.001326983	'PROTON_e + THYMINE_e <=> THYMINE_c + PROTON_c '
'transport_cytidine'	0	0	0	'PROTON_e + CYTIDINE_e <=> PROTON_c + CYTIDINE_c '
'transport_deoxycytidine'	0.003913532	0.003444882	0.002507584	'PROTON_e + DEOXYCYTIDINE_e <=> DEOXYCYTIDINE_c + PROTON_c '
'transport_phosphate_in'	0	0	0	'WATER_c + ATP_c + Pi_e -> ADP_c + PROTON_c + 2 Pi_c '
'transport_acetate'	11.33412922	11.33695184	10.84259709	'ACET_c + PROTON_c -> PROTON_e + ACET_e '

'transport_pyruvate'	0	0	0	'PYRUVATE_e -> PYRUVATE_c '
'transport_CARBONDIOXIDE'	11.33412922	11.33695184	11.09259709	'CARBONDIOXIDE_c <=> CARBONDIOXIDE__e '
'transport_WATER'	12.04317869	11.99011019	10.63397319	'WATER_c <=> WATER_e '
'transport_OXYGENMOLECULE'	-11.96240142	-11.96635303	-11.47425626	'OXYGENMOLECULE_c <=> OXYGENMOLECULE__e '
'transport_AMMONIA'	0.003075826	0.002707493	0.501970826	'AMMONIA_c <=> AMMONIA__e '
'transport_Riboflavin'	0	0	0	'RIBOFLAVIN_e -> RIBOFLAVIN_c '
'transport_H2O2'	0.629224134	0.630239128	0.632269114	'HYDROGENPEROXIDE_c -> HYDROGENPEROXIDE_e '
'transport_FA'	-0.011505088	-0.010127342	-0.007371851	'LongChainFattyAcids_c <=> LongChainFattyAcids_e '
'transport_panthetheine'	0	0	0	'PANTETHEINEP_c <=> PANTETHEINEP_e '
'transport_PHOSPHATIDYL_CHOLINE'	-0.003450264	-0.003037091	-0.002210746	'PHOSPHATIDYL_CHOLINE_c <=> PHOSPHATIDYL_CHOLINE_e '
'transport_MYO_INOSITOL'	-0.25	0	0	'MYOINOSITOL_c <=> MYOINOSITOL_e '
'transport_phosphate_out'	0.108976974	0.111219075	0.115703277	'Pi_c -> Pi_e '
'transport_niacine'	0	0	0	'NIACINE_e -> NIACINE_c '
'EX_PROTON_e'	10.40302367	10.20866395	10.31994451	'PROTON_e <=> '
'EX_RIBOSE_e'	0	0	0	'RIBOSE_e <=> '
'EX_LLACTATE_e'	0	0	0	'LLACTATE_e <=> '
'EX_FRU_e'	0	0	0	'FRU_e <=> '
'EX_ASCORBATE_e'	0	0	0	'ASCORBATE_e <=> '
'EX_MANNOSE_e'	0	0	0	'MANNOSE_e <=> '
'EX_CPD4422_e'	0	0	0	'CPD4422_e <=> '
'EX_GLYCEROL_e'	-0.51	-0.51	-0.51	'GLYCEROL_e <=> '
'EX_SER_e'	-0.012743679	-0.011217611	-0.008165475	'SER_e <=> '
'EX_GLYCEROL3P_e'	-0.1277	-0.1277	-0.1277	'GLYCEROL3P_e <=> '
'EX_MANNITOL_e'	0	0	0	'MANNITOL_e <=> '
'EX_DGlucose_e'	-5.11	-5.11	-5.11	'DGlucose_e <=> '
'EX_LALPHAALANINE_e'	-0.016652531	-0.014658374	-0.010670059	'LALPHAALANINE_e <=> '
'EX_ARG_e'	-0.007978483	-0.007023051	-0.255112189	'ARG_e <=> '
'EX_ASN_e'	-0.011984486	-0.010549331	-0.007679023	'ASN_e <=> '
'EX_LASPARTATE_e'	-0.011249121	-0.009902028	-0.007207841	'LASPARTATE_e <=> '
'EX_CYS_e'	-0.001798787	-0.00158338	-0.001152567	'CYS_e <=> '
'EX_GLT_e'	-0.012828384	-0.011292172	-0.008219749	'GLT_e <=> '
'EX_GLN_e'	-0.01149205	-0.010115866	-0.007363497	'GLN_e <=> '
'EX_GLY_e'	-0.013020478	-0.011461263	-0.008342832	'GLY_e <=> '
'EX_HIS_e'	-0.003712598	-0.003268011	-0.002378836	'HIS_e <=> '
'EX_ILE_e'	-0.013288553	-0.011697236	-0.008514601	'ILE_e <=> '
'EX_LEU_e'	-0.018438706	-0.016230653	-0.011814546	'LEU_e <=> '
'EX_LYS_e'	-0.019279625	-0.016970871	-0.012353362	'LYS_e <=> '
'EX_MET_e'	-0.003012943	-0.00265214	-0.001930534	'MET_e <=> '
'EX_PHE_e'	-0.009245881	-0.008138678	-0.005924271	'PHE_e <=> '
'EX_PRO_e'	-0.008715147	-0.007671499	-0.005584204	'PRO_e <=> '
'EX_THR_e'	-0.012693513	-0.011173452	-0.00813333	'THR_e <=> '
'EX_TRP_e'	-0.001886078	-0.001660218	-0.001208499	'TRP_e <=> '
'EX_TYR_e'	-0.006051462	-0.005326793	-0.003877456	'TYR_e <=> '
'EX_VAL_e'	-0.015117855	-0.013307476	-0.00968672	'VAL_e <=> '
'EX_GUANINE_e'	-0.002840569	-0.002500408	-0.001820086	'GUANINE_e <=> '
'EX_URACIL_e'	0	0	0	'URACIL_e <=> '
'EX_ADENINE_e'	-0.004145388	-0.003648974	-0.002656145	'ADENINE_e <=> '
'EX_THYMINE_e'	-0.002070994	-0.00182299	-0.001326983	'THYMINE_e <=> '
'EX_CYTIDINE_e'	0	0	0	'CYTIDINE_e <=> '
'EX_DEOXYCYTIDINE_e'	-0.003913532	-0.003444882	-0.002507584	'DEOXYCYTIDINE_e <=> '
'EX_Pi_e'	0.108976974	0.111219075	0.115703277	'Pi_e <=> '
'EX_ACET_e'	11.33412922	11.33695184	10.84259709	'ACET_e <=> '
'EX_PYRUVATE_e'	0	0	0	'PYRUVATE_e <=> '
'EX_CARBONDIOXIDE__e'	11.33412922	11.33695184	11.09259709	'CARBONDIOXIDE__e <=> '
'EX_WATER_e'	12.04317869	11.99011019	10.63397319	'WATER_e <=> '
'EX_OXYGENMOLECULE__e'	-11.96240142	-11.96635303	-11.47425626	'OXYGENMOLECULE__e <=> '
'EX_AMMONIA__e'	0.003075826	0.002707493	0.501970826	'AMMONIA__e <=> '
'EX_RIBOFLAVIN_e'	0	0	0	'RIBOFLAVIN_e <=> '
'EX_HYDROGENPEROXIDE_e'	0.629224134	0.630239128	0.632269114	'HYDROGENPEROXIDE_e <=> '
'EX_LongChainFattyAcids_e'	-0.011505088	-0.010127342	-0.007371851	'LongChainFattyAcids_e <=> '
'EX_PANTETHEINEP_e'	0	0	0	'PANTETHEINEP_e <=> '
'EX_PHOSPHATIDYL_CHOLINE_e'	-0.003450264	-0.003037091	-0.002210746	'PHOSPHATIDYL_CHOLINE_e <=> '
'EX_MYOINOSITOL_e'	-0.25	-0.25	0	'MYOINOSITOL_e <=> '
'EX_NIACINE_e'	0	0	0	'NIACINE_e <=> '
'Protein_synthesis'	0.000947411	0.000875738	0.000732394	'482 WATER_c + 22 ChargedGLTtRNAs_c + 21 ChargedTHRtRNAs_c + 21 ChargedGLYtRNAs_c + 32 ChargedLYStRNAs_c + 21 ChargedSERtRNAs_c + 6 ChargedHIStRNAs_c + 826 ATP_c + 13 ChargedARGtRNAs_c + 17 ChargedGLNtRNAs_c + 3 ChargedTRPtRNAs_c + 29 ChargedLEUtRNAs_c + 25 ChargedVALtRNAs_c + 14 ChargedPROtRNAs_c + 15 ChargedPHEtRNAs_c + 22 ChargedILEtRNAs_c + 10 ChargedTYRtRNAs_c + 27 ChargedALAtRNAs_c + 18 ChargedASPtRNAs_c + 20 ChargedASNtRNAs_c + 5 ChargedMETtRNAs_c + 3 ChargedCYStRNAs_c -> 21 THRtRNAs_c + 17 GLNtRNAs_c + 22 ILEtRNAs_c + 29 LEUtRNAs_c + 826 ADP_c + 21 GLYtRNAs_c + 5 METtRNAs_c + 25 VALtRNAs_c + 13 ARGtRNAs_c + 826 PROTON_c + 20 ASNtRNAs_c + 21 SERtRNAs_c + 10 TYRtRNAs_c + 826 Pi_c + 32 LYStRNAs_c + 18 ASPtRNAs_c + 14 PROtRNAs_c + 27 ALAtRNAs_c + 3 TRPtRNAs_c + 22 GLTtRNAs_c + 3 CYStRNAs_c + 6 HIStRNAs_c + 15 PHEtRNAs_c + PROT_mol_c '
'Protein_synthesis_gram'	0.02275706	0.020031879	0.014581518	'0.0263 PROT_mol_c <=> PROT_g_c '
'ACP_synthesis'	3.88301506570	3.41802014964229	2.4880303175302	'199 WATER_c + 11 ChargedGLTtRNAs_c + ChargedTHRtRNAs_c + ChargedGLYtRNAs_c + 11 ChargedLYStRNAs_c + 5 ChargedSERtRNAs_c + ChargedHIStRNAs_c + 199 ATP_c + 2 ChargedARGtRNAs_c + 4 ChargedGLNtRNAs_c + 12 ChargedLEUtRNAs_c + 5 ChargedVALtRNAs_c + ChargedPROtRNAs_c + 5 ChargedPHEtRNAs_c + 8 ChargedILEtRNAs_c + 4 ChargedALAtRNAs_c + 6 ChargedASPtRNAs_c + 3 ChargedASNtRNAs_c + 4 ChargedMETtRNAs_c -> THRtRNAs_c + 4 GLNtRNAs_c + 8 ILEtRNAs_c + 12 LEUtRNAs_c + 199 ADP_c + GLYtRNAs_c + 4 METtRNAs_c + 5 VALtRNAs_c + 2 ARGtRNAs_c + 199 PROTON_c + 3 ASNtRNAs_c + 5 SERtRNAs_c + 199 Pi_c + 11 LYStRNAs_c + 6 ASPtRNAs_c + PROtRNAs_c + 4 ALAtRNAs_c + 11 GLTtRNAs_c + HIStRNAs_c + ACP_c + 5 PHEtRNAs_c '
'ACP_synthesis_gram'	3.67049349279	3.23094824352665	2.3518577563663	'0.10579 ACP_c <=> ACP_g_c '
'Protein_degradation'	0.0003489	0.0003489	0.0003489	'1032 WATER_c + 688 ATP_c + PROT_mol_c -> 25 VAL_c + 17 GLN_c + 21 GLY_c + 22 GLT_c + 6 HIS_c + 688 ADP_c + 14 PRO_c + 10 TYR_c + 688 PROTON_c + 18 LASPARTATE_c + 21 SER_c + 20 ASN_c + 3 CYS_c + 5 MET_c + 21 THR_c + 15 PHE_c + 688 Pi_c + 27 LALPHAALANINE_c + 13 ARG_c + 22 ILE_c + 32 LYS_c + 3 TRP_c + 29 LEU_c '
'ACP_degradation'	0	0	0	'219 WATER_c + 146 ATP_c + ACP_c -> 5 VAL_c + 4 GLN_c + GLY_c + 11 GLT_c + HIS_c + 146 ADP_c + PRO_c + 146 PROTON_c + 6 LASPARTATE_c + 5 SER_c + 3 ASN_c + 4 MET_c + THR_c + 5 PHE_c + 146 Pi_c + 4 LALPHAALANINE_c + 2 ARG_c + 8 ILE_c + 11 LYS_c + 12 LEU_c '
'DNA_synthesis'	2.90869908534	2.56037947695879	1.8637402601978	'140 WATER_c + 71.2 TTP_c + 71.2 DATP_c + 140 ATP_c + 28.8 DGTP_c + 28.8 DCTP_c -> 140 ADP_c + 140 PROTON_c + 140 Pi_c + 200 PPI_c + DNA_mmol_c '
'DNA_synthesis_gram'	0.001835247	0.001615474	0.001175929	'0.015849 DNA_mmol_c <=> DNA_g_c '
'RNA_synthesis'	0.007812531	0.007803965	0.007786833	'40 WATER_c + 18 CTP_c + 69 ATP_c + 28 GTP_c + 25 UTP_c -> 40 ADP_c + 40 PROTON_c + 40 Pi_c + 100 PPI_c + RNA_mmol_c '
'RNA_synthesis_gram'	0.002385821	0.002100116	0.001528708	'0.029982 RNA_mmol_c <=> RNA_g_c '
'RNA_degradation'	0.007741	0.007741	0.007741	'100 WATER_c + RNA_mmol_c -> 18 CMP_c + 100 PROTON_c + 25 UMP_c + 29 AMP_c + 28 GMP_c '
'LPHOSPHATIDATE_synthesis_gram'	0.00022023	0.000193857	0.000141111	'1.389 LPHOSPHATIDATE_c <=> LPHOSPHATIDATE_g_c '
'CARDIOLIPIN_synthesis_gram'	0.003670493	0.003230948	0.002351858	'0.74195 CARDIOLIPIN_c <=> CARDIOLIPIN_g_c '
'PHOSPHATIDYL_CHOLINE_gram'	0	0	0	'1.3661 PHOSPHATIDYL_CHOLINE_c <=> PHOSPHATIDYL_CHOLINE_g_c '
'LIPID_synthesis'	0.007340987	0.006461896	0.004703716	'0.47 PHOSPHATIDYL_CHOLINE_c + 0.03 LPHOSPHATIDATE_g_c + 0.5 CARDIOLIPIN_g_c -> LIPIDS_c '
'G6P_gram'	0	0	0	'1.3661 Dglucose6phosphate_c <=> Dglucose6phosphate_g_c

Table S8

'AAbiomass_mol'	4.85760064380	4.27589813547447	3.1124931188146	'2793 VAL_c + 294 GLN_c + 9220 GLY_c + 18651 GLT_c + 2422 HIS_c + 6837 PRO_c + 1366 TYR_c + 9318 LASPARTATE_c + 3202 SER_c + 54 ASN_c + 67 CYS_c + 100 MET_c + 2489 THR_c + 5122 PHE_c + 9824 LALPHAALANINE_c + 3913 ARG_c + 1858 ILE_c + 1741 LYS_c + 1864 TRP_c + 21313 LEU_c -> AAbiomass_mol_c '
'AAbiomass_gram'	0.000550574	0.000484642	0.000352779	'8.8228e-05 AAbiomass_mol_c <=> AAbiomass_g_c '
'Biomass_synthesis'	0.036704935	0.032309482	0.023518578	'11.53 WATER_c + 11.53 ATP_c + 0.62 PROT_g_c + 0.001 ACP_g_c + 0.05 DNA_g_c + 0.065 RNA_g_c + 0.2 LIPIDS_c + 0.015 AAbiomass_g_c -> 11.53 ADP_c + 11.53 PROTON_c + 11.53 Pi_c + BIOMASS_g_c '
'EX_BIOMASS_g_c'	0.036704935	0.032309482	0.023518578	'BIOMASS_g_c <=> '
'DEOXYGUANOSINEKINASERXN'	0.000837705	0.000737389	0.000536757	'ATP_c + DEOXYGUANOSINE_c <=> ADP_c + PROTON_c + DGMP_c '
'DEOXYADENOSINEKINASERXN'	0.000167127	0.000147114	0.000107086	'ATP_c + DEOXYADENOSINE_c <=> ADP_c + PROTON_c + DAMP_c '
'UMPKINASERXN'	0.196600826	0.196232493	0.195495826	'ATP_c + UMP_c <=> UDP_c + ADP_c '
'ACYLCOASYNTHRXN'	0.011505088	0.010127342	0.007371851	'ATP_c + COA_c + LongChainFattyAcids_c <=> PPI_c + AMP_c + LongChainAcylCoAs_c '
'NADKINRXN'	-0.001903867	-0.001675877	-0.001219897	'ATP_c + NAD_c <=> ADP_c + PROTON_c + NADP_c '
'NADHKINRXN'	0.001903867	0.001675877	0.001219897	'ATP_c + NADH_c <=> ADP_c + PROTON_c + NADPH_c '
'ABC_myoInositol'	NA	0.25	NA	'WATER_c + ATP_c + MYOINOSITOL_e -> ADP_c + Pi_c + MYOINOSITOL_c '
'ArcA'	NA	NA	0.25	'WATER_c + ARG_c -> AMMONIA_c + PROTON_c + LCITRULLINE_c '
'OTC'	NA	NA	0.25	'PROTON_c + Pi_c + LCITRULLINE_c <=> LORNITHINE_c + carbamoyl_phosphate_c '
'Ckase'	NA	NA	0.25	'ADP_c + PROTON_c + carbamoyl_phosphate_c <=> AMMONIA_c + ATP_c + CARBONDIOXIDE_c '
'Ornithine_transport'	NA	NA	0.25	'ARG_e + LORNITHINE_c <=> ARG_c + LORNITHINE_e '
'EX_LORNITHINE_e'	NA	NA	0.25	'LORNITHINE_e <=> '
