# Supplementary material for: Metabolic modeling of energy balances in Mycoplasma hyopneumoniae shows that pyruvate addition increases growth rate
Source: Biotechnol Bioeng. 2017 Jul 27;114(10):2339–47. doi: 10.1002/bit.26347 (PMC6084303; doi:10.1002/bit.26347)
Supplement: Supplementary file 10 — Table S9. Predicted fluxes for the exponential growth phase. [file BIT-114-2339-s010.pdf]

Table S9: Predicted Fluxes for the Exponential Growth Phase

Table copied from: Wodke J a H, Puchałka J, Lluch-Senar M, Marcos J, Yus E, Godinho M, Gutiérrez-Gallego R, dos Santos V a PM, Serrano L, Klipp E, Maier T. 2013. Dissecting the energy metabolism in Mycoplasma pneumoniae through genome-scale metabolic modeling. Mol. Syst. Biol. 9:653. http://www.pubmedcentral.nih.gov/articlerender.fcgi?artid=3658275&tool=pmcentrez&rendertype=abstract.

reaction ID	24h	36h	48h	60h	24H flux	ATP in reaction?	
M001	5.10836	7.369	9.69714	12.3776		False	D-glucose[e] + phosphoenolpyruvate[c] --> D-glucose 6-phosphate[c] + pyruvate[c]
M002	5.0835	7.34078	9.67749	12.3683		False	[c]: D-glucose 6-phosphate <==> D-fructose 6-phosphate
M003	5.16243	7.45648	9.8343	12.5723	-5.16243	TRUE	[c]: D-fructose 6-phosphate + ATP --> ADP + D-fructose 1,6-bisphosphate + H+
M004	5.16243	7.45648	9.8343	12.5723		False	[c]: D-fructose 1,6-bisphosphate <==> dihydroxyacetone phosphate + D-glyceraldehyde 3-phosphate
M005	-5.40839	-7.81419	-10.3117	-13.1877		False	[c]: D-glyceraldehyde 3-phosphate <==> dihydroxyacetone phosphate
M006	10.6103	15.3285	20.2244	25.862		False	[c]: NAD+ + orthophosphate + D-glyceraldehyde 3-phosphate <==> NADH + H+ + 1,3-bisphospho-D-glycerate
M007	-10.6103	-15.3285	-20.2244	-25.862	10.6103	TRUE	[c]: 3-phospho-D-glycerate + ATP <==> ADP + 1,3-bisphospho-D-glycerate
M008	-10.6103	-15.3285	-20.2244	-25.862		False	[c]: 2-phospho-D-glycerate <==> 3-phospho-D-glycerate
M009	10.6103	15.3285	20.2244	25.862		False	[c]: 2-phospho-D-glycerate <==> phosphoenolpyruvate + H2O
M010	4.9216	7.37505	9.95335	12.9233	4.9216	TRUE	[c]: ADP + phosphoenolpyruvate + H+ --> ATP + pyruvate
M011	-3.16668	-8.39742	-15.6053	-23.1843		False	[c]: (S)-lactate + NAD+ <==> NADH + H+ + pyruvate
M012	7.4436	6.9311	4.6191	2.6777		False	[c]: lipoamide + H+ + pyruvate --> S-acetyldihydrolipoamide + CO2
M013	-7.4436	-6.9311	-4.6191	-2.6777		False	[c]: acetyl-CoA + dihydrolipoamide <==> S-acetyldihydrolipoamide + CoA
M014	7.4436	6.9311	4.6191	2.6777		False	[c]: dihydrolipoamide + NAD+ <==> lipoamide + NADH + H+
M015	7.4436	6.9311	4.6191	2.6777		False	[c]: acetyl-CoA + orthophosphate <==> acetyl phosphate + CoA
M016	-7.4436	-6.9311	-4.6191	-2.6777	7.4436	TRUE	[c]: acetate + ATP <==> ADP + acetyl phosphate
M017	7.44291	6.93027	4.61863	2.67759		False	[c]: (2) NADH + oxygen + (2) H+ <==> (2) H2O + (2) NAD+
M018	0.0922169	0.100645	0.0791637	0.0533939	0.0922169	TRUE	(4) H+[e] + ADP[c] + orthophosphate[c] <==> H2O[c] + (3) H+[c] + ATP[c]
M019	0	0	0	0		False	D-fructose[e] + phosphoenolpyruvate[c] --> D-fructose 1-phosphate[c] + pyruvate[c]
M020	0	0	0	0		TRUE	[c]: D-fructose 1-phosphate + ATP --> ADP + D-fructose 1,6-bisphosphate + H+
M021	0	0	0	0		False	[c]: D-fructose 1-phosphate --> D-glyceraldehyde + dihydroxyacetone phosphate
M022	0	0	0	0		False	[c]: glycerol + NAD+ <==> D-glyceraldehyde + NADH + H+
M023	0	0	0	0		False	D-mannose[e] + phosphoenolpyruvate[c] --> D-mannose 6-phosphate[c] + pyruvate[c]
M024	0	0	0	0		False	[c]: D-mannose 6-phosphate <==> D-fructose 6-phosphate
M025	0	0	0	0		False	mannitol[e] + phosphoenolpyruvate[c] --> pyruvate[c] + D-mannitol 1-phosphate[c]
M026	0	0	0	0		False	[c]: NAD+ + D-mannitol 1-phosphate <==> NADH + D-fructose 6-phosphate + H+
M027	0	0	0	0		False	L-ascorbate[e] + phosphoenolpyruvate[c] + (2) H+[c] --> L-ascorbate 6-phosphate[c] + pyruvate[c]
M028	0	0	0	0		False	[c]: H2O + L-ascorbate 6-phosphate <==> 3-keto-L-gulonate 6-phosphate + (3) H+
M029	0	0	0	0		False	[c]: 3-keto-L-gulonate 6-phosphate + H+ --> CO2 + L-xylulose 5-phosphate
M030	0	0	0	0		False	[c]: L-ribulose 5-phosphate <==> L-xylulose 5-phosphate
M031	0	0	0	0		False	[c]: L-ribulose 5-phosphate <==> D-xylulose 5-phosphate
M032	0.127709	0.184225	0.242428	0.30944		False	D-ribose[e] --> D-ribose[c]
M033	0.127709	0.184225	0.242428	0.30944	-0.127709	TRUE	[c]: D-ribose + ATP --> ADP + D-ribose 5-phosphate + H+
M034	-0.0394621	-0.0578489	-0.0784016	-0.102028		False	[c]: D-fructose 6-phosphate + D-glyceraldehyde 3-phosphate <==> D-xylulose 5-phosphate + D-erythrose 4-phosphate
M035	0.0394621	0.0578489	0.0784016	0.102028		False	[c]: sedoheptulose 7-phosphate + D-glyceraldehyde 3-phosphate <==> D-erythrose 4-phosphate + D-fructose 6-phosphate
M036	-0.0394621	-0.0578489	-0.0784016	-0.102028		False	[c]: sedoheptulose 7-phosphate + D-glyceraldehyde 3-phosphate <==> D-xylulose 5-phosphate + D-ribose 5-phosphate
M037	0.0789242	0.115698	0.156803	0.204056		False	[c]: D-ribulose 5-phosphate <==> D-xylulose 5-phosphate
M038	0.0789242	0.115698	0.156803	0.204056		False	[c]: D-ribose 5-phosphate <==> D-ribulose 5-phosphate
M039	0	0	0	0		TRUE	[c]: D-ribose 5-phosphate + ATP --> AMP + 5-phospho-alpha-D-ribose 1-diphosphate + H+
M040	-0.0093227	-0.0106783	-0.0072231	-0.00335526		False	[c]: D-ribose 1-phosphate <==> D-ribose 5-phosphate + (2) H+
M041	0	0	0	0		False	[c]: 2-deoxy-D-ribose 1-phosphate <==> 2-deoxy-D-ribose 5-phosphate
M042	0	0	0	0		False	[c]: 2-deoxy-D-ribose 5-phosphate --> acetaldehyde + D-glyceraldehyde 3-phosphate
M043	0	0	0	0		False	[c]: acetaldehyde + NAD+ + CoA <==> acetyl-CoA + NADH + H+
M044	-0.127709	-0.184225	-0.242428	-0.30944		False	glycerol[c] <==> glycerol[e]
M045	0.127709	0.184225	0.242428	0.30944	-0.127709	TRUE	[c]: glycerol + ATP --> ADP + sn-glycerol 3-phosphate + H+
M046	0.127709	0.184225	0.242428	0.30944	-0.127709	TRUE	sn-glycerol 3-phosphate[e] + H2O[c] + ATP[c] --> ADP[c] + orthophosphate[c] + H+[c] + sn-glycerol 3-phosphate[c]
M047	0.245959	0.357712	0.477379	0.615333		False	[c]: oxygen + sn-glycerol 3-phosphate <==> H2O2 + dihydroxyacetone phosphate
M048	0.01	0.01	0.01	0.01		False	[c]: H2O + dihydroxyacetone phosphate --> dihydroxyacetone + orthophosphate
M049	0.01	0.01	0.01	0.01	-0.01	TRUE	[c]: dihydroxyacetone + ATP --> ADP + dihydroxyacetone phosphate + H+
M050	0.00945854	0.0107382	0.00747673	0.00354742		False	[c]: ACP-R (Mpn) + sn-glycerol 3-phosphate --> 1-acyl-glycerol 3-phosphate (Mpn) + acyl carrier protein
M051	0.00945854	0.0107382	0.00747673	0.00354742		False	[c]: ACP-R (Mpn) + 1-acyl-glycerol 3-phosphate (Mpn) --> phosphatidic acid (Mpn) + acyl carrier protein
M052	0	0	0	0		False	[c]: phosphatidic acid (Mpn) + CTP + H+ --> CDP-diacylglycerol (Mpn) + pyrophosphate
M053	0	0	0	0		False	[c]: CDP-diacylglycerol (Mpn) + sn-glycerol 3-phosphate --> CMP + phosphatidylglycerol 3-phosphate (Mpn) + H+
M054	0	0	0	0		False	[c]: H2O + phosphatidylglycerol 3-phosphate (Mpn) --> orthophosphate + phosphatidylglycerol (Mpn)
M055	0	0	0	0		False	[c]: CDP-diacylglycerol (Mpn) + phosphatidylglycerol (Mpn) --> cardiolipin (Mpn) + CMP + H+
M056	0.00284386	0.0032286	0.00224799	0.00106659		False	[c]: H2O + phosphatidic acid (Mpn) --> diacylglycerol (Mpn) + orthophosphate
M057	-0.0170631	-0.0193716	-0.013488	-0.00639952		False	[c]: D-glucose 1-phosphate <==> D-glucose 6-phosphate
M058	0.0170631	0.0193716	0.013488	0.00639952		False	[c]: D-glucose 1-phosphate + UTP + H+ <==> UDP-glucose + pyrophosphate
M059	0.00853157	0.00968581	0.00674398	0.00319976		False	[c]: UDP-glucose <==> UDP-galactose
M060	0.00284386	0.0032286	0.00224799	0.00106659		False	[c]: diacylglycerol (Mpn) + (3) UDP-galactose + (3) UDP-glucose --> glycolipid (Mpn) + (6) H+ + (6) UDP
M061	4.46E-06	5.07E-06	3.53E-06	1.67E-06	-4.46E-06	TRUE	[c]: choline + ATP --> ADP + H+ + choline phosphate
M062	4.46E-06	5.07E-06	3.53E-06	1.67E-06		False	[c]: CTP + H+ + choline phosphate --> CDP-choline + pyrophosphate

M063	0	0	0	0		False	[c]: sn-glycero-3-phosphocholine + H2O <==> choline + sn-glycerol 3-phosphate + H+
M064	0	0	0	0		False	[c]: phosphatidylcholine + (2) H2O <==> sn-glycero-3-phosphocholine + (2) fatty acid (Mpn)
M065	-0.0028193	-0.0046814	-0.0031536	-0.00146034		False	[c]: AMP + pyrophosphate <==> adenine + 5-phospho-alpha-D-ribose 1-diphosphate
M066	-8.84E-05	-0.0001004	-6.99E-05	-3.32E-05		False	[c]: adenosine + orthophosphate + (2) H+ <==> D-ribose 1-phosphate + adenine
M067	0	0	0	0		False	[c]: H2O + AMP --> adenosine + orthophosphate
M068	0.613989	0.651928	0.558451	0.446129	-0.613989	TRUE	[c]: AMP + ATP <==> (2) ADP
M069	0	0.00143352	0.00099812	0.00047357		False	[c]: ADP + reduced thioredoxin --> dADP + H2O + oxidized thioredoxin
M070	0.00126269	0.00143352	0.00099812	0.000621014		False	[c]: oxidized thioredoxin + H+ + NADPH <==> NADP+ + reduced thioredoxin
M071	0.00783096	0.00784313	0.00781211	0.00777474	-0.5403362	TRUE	[c]: (28) GTP + (25) UTP + (40) H2O + (18) CTP + (69) ATP --> (40) ADP + RNA (Mpn) + (40) orthophosphate + (40) H+ + (100) pyrophosphate
M072	1.1943	1.23496	1.13134	1.00678		False	[c]: H2O + pyrophosphate --> (2) orthophosphate + H+
M073	-0.002175	-0.0010358	-0.0007212	-0.000342168		False	[c]: deoxyadenosine + orthophosphate <==> 2-deoxy-D-ribose 1-phosphate + adenine
M074	0.00217501	0.00103576	0.00072117	0.000342168	-0.002175	TRUE	[c]: deoxyadenosine + ATP <==> ADP + H+ + dAMP
M075	0.00217501	0.00103576	0.00072117	0.000342168	-0.002175	TRUE	[c]: dAMP + ATP <==> dADP + ADP
M076	0.00217501	0.00246927	0.00171929	0.000815738		False	[c]: phosphoenolpyruvate + dADP + H+ --> dATP + pyruvate
M077	0	0	0	0			[c]: 3-phospho-D-glycerate + dATP <==> dADP + 1,3-bisphospho-D-glycerate
M078	7.25E-05	8.23E-05	5.73E-05	2.72E-05	-1.02E-02	TRUE	[c]: (20) dGTP + (140) H2O + (30) dTTP + (20) dCTP + (140) ATP + (30) dATP -> (140) ADP + DNA (Mpn) + (140) orthophosphate + (140) H+ + (100) pyrophosphate
M079	-0.0037815	-0.0028595	-0.001991	-0.000944662		False	[c]: GMP + pyrophosphate <==> 5-phospho-alpha-D-ribose 1-diphosphate + guanine
M080	-7.40E-05	-8.40E-05	-5.85E-05	-2.77E-05		False	[c]: guanosine + orthophosphate + (2) H+ <==> D-ribose 1-phosphate + guanine
M081	0	0	0	0		False	[c]: GMP + H2O --> guanosine + orthophosphate
M082	0.220529	0.219608	0.218739	0.217693	-0.220529	TRUE	[c]: GMP + ATP <==> ADP + GDP
M083	0.00126269	0	0	0		False	[c]: reduced thioredoxin + GDP --> H2O + oxidized thioredoxin + dGDP
M084	0.219267	0.219608	0.218739	0.217693		False	[c]: phosphoenolpyruvate + H+ + GDP --> GTP + pyruvate
M085	0	0	0	0		False	[c]: GTP + 3-phospho-D-glycerate <==> GDP + 1,3-bisphospho-D-glycerate
M086	-0.0001873	-0.0016462	-0.0011462	-0.000543825		False	[c]: deoxyguanosine + orthophosphate <==> 2-deoxy-D-ribose 1-phosphate + guanine
M087	0.00018732	0.00164618	0.00114619	0.000543825	-0.0001873	TRUE	[c]: deoxyguanosine + ATP <==> ADP + H+ + dGMP
M088	0.00018732	0.00164618	0.00114619	0.000543825	-0.0001873	TRUE	[c]: ATP + dGMP <==> ADP + dGDP
M089	0.00145001	0.00164618	0.00114619	0.000543825		False	[c]: phosphoenolpyruvate + dGDP + H+ --> dGTP + pyruvate
M090	0	0	0	0		False	[c]: 3-phospho-D-glycerate + dGTP <==> dGDP + 1,3-bisphospho-D-glycerate
M091	0.00679791	0.00781201	0.00522737	0.00240835		False	[c]: UMP + pyrophosphate <==> uracil + 5-phospho-alpha-D-ribose 1-diphosphate
M092	-0.0091603	-0.010494	-0.0070947	-0.00329435		False	[c]: orthophosphate + (2) H+ + uridine <==> uracil + D-ribose 1-phosphate
M093	0	0	0	0		False	[c]: H2O + UMP --> orthophosphate + uridine
M094	0.00904681	0.0103652	0.00700506	0.0032518	-0.0090468	TRUE	[c]: ATP + uridine --> ADP + UMP + H+
M095	0.195774	0.196078	0.195303	0.194368	-0.195774	TRUE	[c]: UMP + ATP <==> ADP + UDP
M096	0.212837	0.21545	0.208791	0.200768		False	[c]: phosphoenolpyruvate + H+ + UDP --> UTP + pyruvate
M097	0	0	0	0		False	[c]: reduced thioredoxin + UDP --> H2O + oxidized thioredoxin + dUDP
M098	0	0	0	0		False	[c]: cytidine + H2O --> NH3 + uridine
M099	0.00162367	0.00184334	0.00128347	0.000756401	-0.0016237	TRUE	[c]: cytidine + ATP --> ADP + CMP + H+
M100	0	0	0	0		False	[c]: H2O + CMP --> cytidine + orthophosphate
M101	0.140962	0.141181	0.140621	0.140094	-0.140962	TRUE	[c]: CMP + ATP <==> ADP + CDP
M102	0.140962	0.141181	0.140621	0.139947		False	[c]: phosphoenolpyruvate + CDP + H+ --> CTP + pyruvate
M103	0	0	0	0.000147444		False	[c]: CDP + reduced thioredoxin --> H2O + oxidized thioredoxin + dCDP
M104	0.00145001	0.00164618	0.00114619	0.000396382	-0.00145	TRUE	[c]: deoxycytidine + ATP --> ADP + dCMP + H+
M105	0	0	0	0		False	[c]: H2O + dCMP --> deoxycytidine + orthophosphate
M106	0.00145001	0.00164618	0.00114619	0.000396382	-0.00145	TRUE	[c]: dCMP + ATP <==> ADP + dCDP
M107	0.00145001	0.00164618	0.00114619	0.000543825		False	[c]: phosphoenolpyruvate + dCDP + H+ --> dCTP + pyruvate
M108	0.00459279	0.00521416	0.00363049	0.00172253		False	[c]: H2O + deoxycytidine --> deoxyuridine + NH3
M109	0.00236234	0.00268194	0.00186737	0.000885993		False	[c]: deoxyuridine + orthophosphate <==> uracil + 2-deoxy-D-ribose 1-phosphate
M110	0.00223046	0.00253222	0.00176312	0.000836533	-0.0022305	TRUE	[c]: deoxyuridine + ATP --> ADP + dUMP + H+
M111	0	0	0	0		False	[c]: H2O + dUMP --> deoxyuridine + orthophosphate
M112	0	0	0	0		TRUE	[c]: dUMP + ATP <==> ADP + dUDP
M113	0.00223046	0.00253222	0.00176312	0.000836533		False	[c]: 5,10-methylenetetrahydrofolate + dUMP <==> dTMP + dihydrofolate
M114	0	0	0	0		False	[c]: thymidine + orthophosphate <==> 2-deoxy-D-ribose 1-phosphate + thymine
M115	0	0	0	0		TRUE	[c]: thymidine + ATP --> ADP + dTMP + H+
M116	5.54E-05	6.29E-05	4.38E-05	2.08E-05		False	[c]: H2O + dTMP --> thymidine + orthophosphate
M117	0.00217501	0.00246927	0.00171929	0.000815738	-0.002175	TRUE	[c]: dTMP + ATP <==> ADP + dTDP
M118	0.00217501	0.00246927	0.00171929	0.000815738		False	[c]: phosphoenolpyruvate + dTDP + H+ --> dTTP + pyruvate
M119	0.240511	0.239227	0.242499	0.246441		False	[c]: L-arginine + H2O --> NH3 + L-citrulline + H+
M120	-0.240511	-0.239227	-0.242499	-0.246441		False	[c]: carbamoyl phosphate + L-ornithine <==> orthophosphate + H+ + L-citrulline
M121	-0.240511	-0.239227	-0.242499	-0.246441	0.240511	TRUE	[c]: CO2 + NH3 + ATP <==> ADP + carbamoyl phosphate + H+
M122	-4.46E-06	-5.07E-06	-3.53E-06	-1.67E-06		False	[c]: NAD+ + dihydrofolate <==> NADH + folic acid
M123	-0.0022349	-0.0025373	-0.0017667	-0.000838208		False	[c]: tetrahydrofolate + NAD+ <==> NADH + dihydrofolate + H+
M124	0	0	0	0		TRUE	[c]: tetrahydrofolate + ATP + formate --> ADP + orthophosphate + 10-formyltetrahydrofolate
M125	0.00106551	0.00116246	0.00091536	0.000617665		False	[c]: H2O + 5,10-methenyltetrahydrofolate <==> 10-formyltetrahydrofolate
M126	0.00106551	0.00116246	0.00091536	0.000617665		False	[c]: L-methionyl-tRNA(Met) + 10-formyltetrahydrofolate --> tetrahydrofolate + N-formylmethionyl-tRNA(Met)
M127	-0.0033004	-0.0036998	-0.002682	-0.00145587		False	[c]: glycine + H2O + 5,10-methylenetetrahydrofolate <==> tetrahydrofolate + L-serine
M128	0.00106998	0.00116753	0.00091889	0.000619339		False	[c]: NADP+ + 5,10-methylenetetrahydrofolate <==> H+ + 5,10-methenyltetrahydrofolate + NADPH
M129	0.00999554	0.00999493	0.00999647	0.00999833	-0.0099955	TRUE	[c]: ATP + 5-formyltetrahydrofolate --> ADP + orthophosphate + (2) H+ + 5,10-methenyltetrahydrofolate
M130	0.01	0.01	0.01	0.01		False	[c]: H2O + H+ + 5,10-methenyltetrahydrofolate --> 5-formyltetrahydrofolate
M131	0.00106538	0.00116231	0.00091526	0.000617615		False	[c]: NAD+ + formate <==> CO2 + NADH
M132	4.46E-06	5.07E-06	3.53E-06	1.67E-06	-4.46E-06	TRUE	[c]: H2O + ATP + L-methionine --> orthophosphate + pyrophosphate + S-adenosyl-L-methionine
M133	0	0	0	0		False	[c]: DNA (Mpn) + S-adenosyl-L-methionine --> 5mcDNA (Mpn) + S-adenosyl-L-homocysteine
M134	0	0	0	0		False	[c]: H2O + S-adenosyl-L-homocysteine --> L-homocysteine + adenosine
M135	-0.0001972	-0.0002711	-8.28E-05	-3.35E-06		False	[c]: nicotinate D-ribonucleotide + pyrophosphate <==> nicotinate + 5-phospho-alpha-D-ribose 1-diphosphate + H+

M136	0.00019718	0.00027105	8.28E-05	3.35E-06	-0.0001972	TRUE	[c]: nicotinate D-ribonucleotide + H+ + ATP <==> deamino-NAD+ + pyrophosphate
M137	0.00019718	0.00027105	8.28E-05	3.35E-06	-0.0001972	TRUE	[c]: deamino-NAD+ + NH3 + ATP --> AMP + NAD+ + pyrophosphate
M138	0	0	0	0		TRUE	[c]: NAD+ + ATP --> ADP + NADP+ + H+
M139	0.00019718	0.00027105	8.28E-05	3.35E-06	-0.0001972	TRUE	[c]: NADH + ATP --> ADP + H+ + NADPH
M140	4.46E-06	5.07E-06	3.53E-06	1.67E-06	-4.464E-06	TRUE	[c]: riboflavin + ATP --> ADP + FMN + H+
M141	4.46E-06	5.07E-06	3.53E-06	1.67E-06	-4.464E-06	TRUE	[c]: FMN + H+ + ATP --> pyrophosphate + FAD
M142	4.46E-06	5.07E-06	3.53E-06	1.67E-06	-4.464E-06	TRUE	[c]: pyridoxal + ATP --> ADP + pyridoxal phosphate + H+
M143	4.46E-06	5.07E-06	3.53E-06	1.67E-06	-4.464E-06	TRUE	[c]: ATP + thiamin --> ADP + H+ + thiamin monophosphate
M144	4.46E-06	5.07E-06	3.53E-06	1.67E-06	-4.464E-06	TRUE	[c]: thiamin monophosphate + ATP --> ADP + thiamin diphosphate
M145	4.60E-06	5.22E-06	3.63E-06	1.72E-06	-4.598E-06	TRUE	[c]: pantetheine + ATP --> ADP + H+ + pantetheine 4-phosphate
M146	4.60E-06	5.22E-06	3.63E-06	1.72E-06	-4.598E-06	TRUE	[c]: H+ + ATP + pantetheine 4-phosphate --> pyrophosphate + dephospho-CoA
M147	4.60E-06	5.22E-06	3.63E-06	1.72E-06	-4.598E-06	TRUE	[c]: ATP + dephospho-CoA --> ADP + CoA + H+
M148	1.34E-07	1.52E-07	1.06E-07	5.02E-08		False	[c]: apoprotein [acyl carrier protein] + CoA --> adenosine 3',5'-bisphosphate + acyl carrier protein + H+
M149	0	0	0	0		False	[c]: H2O + acyl carrier protein --> apoprotein [acyl carrier protein] + H+ + pantetheine 4-phosphate
M150	1.34E-07	1.52E-07	1.06E-07	5.02E-08		False	[c]: H2O + adenosine 3',5'-bisphosphate --> AMP + orthophosphate
M151	0.0189171	0.0214764	0.0149535	0.00709484	-0.0189171	TRUE	[c]: fatty acid (Mpn) + acyl carrier protein + ATP --> ACP-R (Mpn) + AMP + H+ + pyrophosphate
M152	0	0	0	0	0	TRUE	[c]: H2O + L-glutamyl-tRNA(Gln) + L-asparagine + ATP --> L-glutaminyl-tRNA(Gln) + ADP + orthophosphate + H+ + L-aspartate
M153	0.018112	0.0197599	0.0155598	0.0104997	-0.018112	TRUE	[c]: L-glutamine + H2O + L-glutamyl-tRNA(Gln) + ATP --> L-glutaminyl-tRNA(Gln) + ADP + L-glutamate + orthophosphate + H+
M154	0.0063928	0.00697447	0.00549196	0.00370589	-0.0063928	TRUE	[c]: tRNA(Met) + L-methionine + ATP --> L-methionyl-tRNA(Met) + AMP + pyrophosphate
M155	0.0234394	0.025572	0.0201365	0.0135879	-0.0234394	TRUE	[c]: tRNA(Ile) + L-isoleucine + ATP --> L-isoleucyl-tRNA(Ile) + AMP + pyrophosphate
M156	0.0266351	0.0290585	0.022882	0.0154406	-0.0266351	TRUE	[c]: tRNA(Val) + L-valine + ATP --> L-valyl-tRNA(Val) + AMP + pyrophosphate
M157	0.0308976	0.0337088	0.0265437	0.0179114	-0.0308976	TRUE	[c]: tRNA(Leu) + L-leucine + ATP --> AMP + L-leucyl-tRNA(Leu) + pyrophosphate
M158	0.00319613	0.00348693	0.00274577	0.00185284	-0.0031961	TRUE	[c]: tRNA(Cys) + ATP + L-cysteine --> AMP + pyrophosphate + L-cysteinyl-tRNA(Cys)
M159	0.0234398	0.0255725	0.0201368	0.0135881	-0.0234398	TRUE	[c]: tRNA(Glu) + L-glutamate + ATP --> L-glutamyl-tRNA(Glu) + AMP + pyrophosphate
M160	0.018112	0.0197599	0.0155598	0.0104997	-0.018112	TRUE	[c]: L-glutamate + ATP + tRNA(Gln) --> L-glutamyl-tRNA(Gln) + AMP + pyrophosphate
M161	0.0138502	0.0151103	0.0118986	0.00802909	-0.0138502	TRUE	[c]: L-arginine + tRNA(Arg) + ATP --> AMP + L-arginyl-tRNA(Arg) + pyrophosphate
M162	0.0106538	0.0116231	0.00915257	0.00617615	-0.0106538	TRUE	[c]: L-tyrosine + tRNA(Tyr) + ATP --> AMP + L-tyrosyl-tRNA(Tyr) + pyrophosphate
M163	0.00319613	0.00348693	0.00274577	0.00185284	-0.0031961	TRUE	[c]: tRNA(Trp) + L-tryptophan + ATP --> AMP + L-tryptophanyl-tRNA(Trp) + pyrophosphate
M164	0.0223736	0.0244093	0.0192209	0.0129702	-0.0223736	TRUE	[c]: tRNA(Ser) + L-serine + ATP --> L-seryl-tRNA(Ser) + AMP + pyrophosphate
M165	0.0223731	0.0244087	0.0192205	0.01297	-0.0223731	TRUE	[c]: L-threonine + tRNA(Thr) + ATP --> AMP + L-threonyl-tRNA(Thr) + pyrophosphate
M166	0.0149154	0.0162725	0.0128137	0.00864666	-0.0149154	TRUE	[c]: tRNA(Pro) + ATP + L-proline --> L-prolyl-tRNA(Pro) + AMP + pyrophosphate
M167	0.0191776	0.0209225	0.0164753	0.0111174	-0.0191776	TRUE	[c]: tRNA(Asp) + L-aspartate + ATP --> AMP + L-aspartyl-tRNA(Asp) + pyrophosphate
M168	0.0213079	0.0232467	0.0183055	0.0123524	-0.0213079	TRUE	[c]: tRNA(Asn) + L-asparagine + ATP --> AMP + L-asparaginyl-tRNA(Asn) + pyrophosphate
M169	0.0340935	0.0371956	0.0292894	0.0197642	-0.0340935	TRUE	[c]: tRNA(Lys) + L-lysine + ATP --> AMP + pyrophosphate + L-lysyl-tRNA(Lys)
M170	0.0063924	0.00697401	0.00549165	0.00370574	-0.0063924	TRUE	[c]: L-histidine + ATP + tRNA(His) --> AMP + L-histidyl-tRNA(His) + pyrophosphate
M171	0.0159813	0.0174354	0.0137294	0.00926447	-0.0159813	TRUE	[c]: tRNA(Phe) + L-phenylalanine + ATP --> AMP + L-phenylalanyl-tRNA(Phe) + pyrophosphate
M172	0.0287657	0.031383	0.0247124	0.0166758	-0.0287657	TRUE	[c]: L-alanine + tRNA(Ala) + ATP --> L-alanyl-tRNA(Ala) + AMP + pyrophosphate
M173	0.0223731	0.0244087	0.0192205	0.01297	-0.0223731	TRUE	[c]: glycine + tRNA(Gly) + ATP --> glycyl-tRNA(Gly) + AMP + pyrophosphate
M174	0.007741	0.007741	0.007741	0.007741		False	[c]: (100) H2O + RNA (Mpn) --> (28) GMP + (29) AMP + (18) CMP + (25) UMP + (100) H+
M175	-7.68518	-7.17149	-4.86251	-2.92476		False	CO2[e] <==> CO2[c]
M176	0	0	0	0		False	uracil[e] + H+[e] <==> uracil[c] + H+[c]
M177	0	0	0	0		False	thymine[e] + H+[e] --> thymine[c] + H+[c]
M178	3.16668	8.39742	15.6053	23.1843		False	(S)-lactate[c] + H+[c] --> (S)-lactate[e] + H+[e]
M179	7.68887	7.28799	5.09601	3.29292		False	oxygen[e] <==> oxygen[c]
M180	4.46E-06	5.07E-06	3.53E-06	1.67E-06		False	riboflavin[e] --> riboflavin[c]
M181	0.00164613	0.00186883	0.00130122	0.000764823		False	cytidine[e] + H+[e] <==> cytidine[c] + H+[c]
M182	0	0	0	0		False	L-homocysteine[c] --> L-homocysteine[e]
M183	0.00404275	0.00458969	0.00319569	0.00151623		False	guanine[e] + H+[e] --> guanine[c] + H+[c]
M184	-0.245959	-0.357712	-0.477379	-0.615333		False	H2O2[e] <==> H2O2[c]
M185	4.46E-06	5.07E-06	3.53E-06	1.67E-06		False	pyridoxal[e] --> pyridoxal[c]
M186	4.46E-06	5.07E-06	3.53E-06	1.67E-06		False	folic acid[e] --> folic acid[c]
M187	0.0189171	0.0214764	0.0149535	0.00709484		False	fatty acid (Mpn)[e] <==> fatty acid (Mpn)[c]
M188	-7.53968	-7.05254	-4.47367	-2.21489		False	H2O[e] <==> H2O[c]
M189	0.00508274	0.00581759	0.00394467	0.00183568		False	adenine[c] + H+[c] <==> adenine[e] + H+[e]
M190	0.00019718	0.00027105	8.28E-05	3.35E-06		False	nicotinate[e] --> nicotinate[c]
M191	4.60E-06	5.22E-06	3.63E-06	1.72E-06		False	pantetheine[e] --> pantetheine[c]
M192	0.0184901	0.0209444	0.014689	0.00715274	-0.0184901	TRUE	L-serine[e] + H2O[c] + ATP[c] --> ADP[c] + L-serine[c] + orthophosphate[c] + H+[c]
M193	0.00436503	0.00495557	0.00345044	0.0016371	-0.004365	TRUE	L-methionine[e] + H2O[c] + ATP[c] --> ADP[c] + orthophosphate[c] + L-methionine[c] + H+[c]
M194	0.485417	0.483397	0.488546	0.494601		False	NH3[c] <==> NH3[e]
M195	4.46E-06	5.07E-06	3.53E-06	1.67E-06	-4.46E-06	TRUE	thiamin[e] + H2O[c] + ATP[c] --> ADP[c] + orthophosphate[c] + H+[c] + thiamin[c]
M196	4.46E-06	5.07E-06	3.53E-06	1.67E-06		False	H+[e] + choline[e] <==> H+[c] + choline[c]
M197	0.240511	0.239227	0.242499	0.246441		False	L-arginine[e] + L-ornithine[c] <==> L-ornithine[e] + L-arginine[c]
M198	7.4436	6.9311	4.6191	2.6777		False	H+[c] + acetate[c] --> H+[e] + acetate[e]
M199	0	0	0	0		TRUE	phosphatidylcholine[e] + ATP[c] + H2O[c] --> phosphatidylcholine[c] + ADP[c] + orthophosphate[c] + H+[c]
M200	0	0	0	0		False	L-alanine[c] + H+[c] --> L-alanine[e] + H+[e]
M201	0	0	0	0		False	L-arginine[c] + H+[c] --> L-arginine[e] + H+[e]
M202	0	0	0	0		False	L-asparagine[c] + H+[c] --> L-asparagine[e] + H+[e]
M203	0	0	0	0		False	H+[c] + L-aspartate[c] --> H+[e] + L-aspartate[e]
M204	0	0	0	0		False	H+[c] + L-cysteine[c] --> H+[e] + L-cysteine[e]

M205	0	0	0	0		False	L-glutamate[c] + H+[c] --> L-glutamate[e] + H+[e]
M206	0	0	0	0		False	L-glutamine[c] + H+[c] --> L-glutamine[e] + H+[e]
M207	0	0	0	0		False	glycine[c] + H+[c] --> glycine[e] + H+[e]
M208	0	0	0	0		False	L-histidine[c] + H+[c] --> L-histidine[e] + H+[e]
M209	0	0	0	0		False	L-isoleucine[c] + H+[c] --> L-isoleucine[e] + H+[e]
M210	0	0	0	0		False	L-leucine[c] + H+[c] --> H+[e] + L-leucine[e]
M211	0	0	0	0		False	L-lysine[c] + H+[c] --> L-lysine[e] + H+[e]
M212	0	0	0	0		False	H+[c] + L-methionine[c] --> H+[e] + L-methionine[e]
M213	0	0	0	0		False	L-phenylalanine[c] + H+[c] --> L-phenylalanine[e] + H+[e]
M214	0	0	0	0		False	H+[c] + L-proline[c] --> H+[e] + L-proline[e]
M215	0	0	0	0		False	L-serine[c] + H+[c] --> L-serine[e] + H+[e]
M216	0	0	0	0		False	L-threonine[c] + H+[c] --> L-threonine[e] + H+[e]
M217	0	0	0	0		False	L-tryptophan[c] + H+[c] --> L-tryptophan[e] + H+[e]
M218	0	0	0	0		False	L-tyrosine[c] + H+[c] --> L-tyrosine[e] + H+[e]
M219	0	0	0	0		False	L-valine[c] + H+[c] --> L-valine[e] + H+[e]
M220	0.0060428	0.00686034	0.00477668	0.00211891		False	deoxycytidine[e] + H+[e] --> deoxycytidine[c] + H+[c]
M221	0.00948916	0.010773	0.00750094	0.00355891	-0.0094892	TRUE	L-arginine[e] + H2O[c] + ATP[c] --> ADP[c] + L-arginine[c] + orthophosphate[c] + H+[c]
M222	0.0133134	0.0151146	0.0105239	0.00499318	-0.0133134	TRUE	L-aspartate[e] + H2O[c] + ATP[c] --> ADP[c] + orthophosphate[c] + H+[c] + L-aspartate[c]
M223	0.00215242	0.00244363	0.00170144	0.000807266	-0.0021524	TRUE	L-cysteine[e] + H2O[c] + ATP[c] --> ADP[c] + orthophosphate[c] + H+[c] + L-cysteine[c]
M224	0.0165966	0.018842	0.0131192	0.00622456	-0.0165966	TRUE	L-glutamate[e] + H2O[c] + ATP[c] --> ADP[c] + orthophosphate[c] + L-glutamate[c] + H+[c]
M225	0.0121573	0.0138493	0.00953696	0.00434156	-0.0121573	TRUE	glycine[e] + H2O[c] + ATP[c] --> ADP[c] + glycine[c] + orthophosphate[c] + H+[c]
M226	0.0158465	0.0179904	0.0125263	0.00594323	-0.0158465	TRUE	L-isoleucine[e] + H2O[c] + ATP[c] --> ADP[c] + L-isoleucine[c] + orthophosphate[c] + H+[c]
M227	0.019784	0.0224606	0.0156387	0.00741998	-0.019784	TRUE	L-alanine[e] + H2O[c] + ATP[c] --> L-alanine[c] + ADP[c] + orthophosphate[c] + H+[c]
M228	0.0143324	0.0162714	0.0113294	0.00537535	-0.0143324	TRUE	L-asparagine[e] + H2O[c] + ATP[c] --> ADP[c] + orthophosphate[c] + L-asparagine[c] + H+[c]
M229	0.0217309	0.0246709	0.0171777	0.00815018	-0.0217309	TRUE	L-leucine[e] + H2O[c] + ATP[c] --> ADP[c] + orthophosphate[c] + L-leucine[c] + H+[c]
M230	0.0121938	0.0138435	0.00963887	0.00457327	-0.0121938	TRUE	L-glutamine[e] + H2O[c] + ATP[c] --> L-glutamine[c] + ADP[c] + orthophosphate[c] + H+[c]
M231	0.00440712	0.00500337	0.00348372	0.00165289	-0.0044071	TRUE	L-histidine[e] + H2O[c] + ATP[c] --> ADP[c] + L-histidine[c] + orthophosphate[c] + H+[c]
M232	0.0230065	0.026119	0.018186	0.00862857	-0.0230065	TRUE	L-lysine[e] + H2O[c] + ATP[c] --> ADP[c] + L-lysine[c] + orthophosphate[c] + H+[c]
M233	0.010336	0.0117344	0.00817038	0.00387653	-0.010336	TRUE	L-proline[e] + H2O[c] + ATP[c] --> ADP[c] + orthophosphate[c] + H+[c] + L-proline[c]
M234	0.0109765	0.0124615	0.00867663	0.00411673	-0.0109765	TRUE	L-phenylalanine[e] + H2O[c] + ATP[c] --> ADP[c] + L-phenylalanine[c] + orthophosphate[c] + H+[c]
M235	0.0151573	0.0172079	0.0119814	0.00568473	-0.0151573	TRUE	L-threonine[e] + H2O[c] + ATP[c] --> ADP[c] + L-threonine[c] + orthophosphate[c] + H+[c]
M236	0.00223265	0.0025347	0.00176485	0.000837354	-0.0022327	TRUE	L-tryptophan[e] + H2O[c] + ATP[c] --> ADP[c] + L-tryptophan[c] + orthophosphate[c] + H+[c]
M237	0.00722576	0.00820333	0.00571177	0.00271002	-0.0072258	TRUE	L-tyrosine[e] + H2O[c] + ATP[c] --> ADP[c] + L-tyrosine[c] + orthophosphate[c] + H+[c]
M238	0.0180373	0.0204776	0.014258	0.00676488	-0.0180373	TRUE	L-valine[e] + H2O[c] + ATP[c] --> ADP[c] + L-valine[c] + orthophosphate[c] + H+[c]
M239	0	0	0	0	0	TRUE	orthophosphate[e] + H2O[c] + ATP[c] --> ADP[c] + (2) orthophosphate[c] + H+[c]
M240	0.0945652	0.146456	0.216448	0.297221		False	orthophosphate[c] --> orthophosphate[e]
M241	3.16668	8.39742	15.6053	23.1843		False	[e]: (S)-lactate -->
M242	0	0	0	0		False	[c]: 5mcDNA (Mpn) -->
M243	7.4436	6.9311	4.6191	2.6777		False	[e]: acetate -->
M244	-0.0050827	-0.0058176	-0.0039447	-0.00183568		False	[e]: adenine <==>
M245	0	0	0	0		False	[c]: adenosine 3',5'-bisphosphate -->
M246	0.0446431	0.0506829	0.0352892	0.0167434		False	[e]: biomass -->
M247	7.68518	7.17149	4.86251	2.92476		False	[e]: CO2 <==>
M248	-0.0121573	-0.0138493	-0.009537	-0.00434156		False	[e]: glycine <==>
M249	0.245959	0.357712	0.477379	0.615333		False	[e]: H2O2 -->
M250	-0.019784	-0.0224606	-0.0156387	-0.00741998		False	[e]: L-alanine <==>
M251	-0.25	-0.25	-0.25	-0.25		False	[e]: L-arginine <==>
M252	-0.0143324	-0.0162714	-0.0113294	-0.00537535		False	[e]: L-asparagine <==>
M253	-0.0133134	-0.0151146	-0.0105239	-0.00499318		False	[e]: L-aspartate <==>
M254	-0.0021524	-0.0024436	-0.0017014	-0.000807266		False	[e]: L-cysteine <==>
M255	-0.0165966	-0.018842	-0.0131192	-0.00622456		False	[e]: L-glutamate <==>
M256	-0.0121938	-0.0138435	-0.0096389	-0.00457327		False	[e]: L-glutamine <==>
M257	-0.0044071	-0.0050034	-0.0034837	-0.00165289		False	[e]: L-histidine <==>
M258	0	0	0	0		False	[e]: L-homocysteine -->
M259	-0.0158465	-0.0179904	-0.0125263	-0.00594323		False	[e]: L-isoleucine <==>
M260	-0.0217309	-0.0246709	-0.0171777	-0.00815018		False	[e]: L-leucine <==>
M261	-0.0230065	-0.026119	-0.018186	-0.00862857		False	[e]: L-lysine <==>
M262	-0.004365	-0.0049556	-0.0034504	-0.0016371		False	[e]: L-methionine <==>
M263	0.240511	0.239227	0.242499	0.246441		False	[e]: L-ornithine -->
M264	-0.0109765	-0.0124615	-0.0086766	-0.00411673		False	[e]: L-phenylalanine <==>
M265	-0.010336	-0.0117344	-0.0081704	-0.00387653		False	[e]: L-proline <==>
M266	-0.0184901	-0.0209444	-0.014689	-0.00715274		False	[e]: L-serine <==>
M267	-0.0151573	-0.0172079	-0.0119814	-0.00568473		False	[e]: L-threonine <==>
M268	-0.0022327	-0.0025347	-0.0017649	-0.000837354		False	[e]: L-tryptophan <==>
M269	-0.0072258	-0.0082033	-0.0057118	-0.00271002		False	[e]: L-tyrosine <==>
M270	-0.0180373	-0.0204776	-0.014258	-0.00676488		False	[e]: L-valine <==>
M271	0.00018825	0.00026092	7.57E-05	0		False	[c]: NADP+ -->
M272	0.485417	0.483397	0.488546	0.494601		False	[e]: NH3 <==>
M273	0	0	0	0		False	[e]: --> ascorbate
M274	-4.46E-06	-5.07E-06	-3.53E-06	-1.67E-06		False	[e]: <==> choline
M275	0.00164613	0.00186883	0.00130122	0.000764823		False	[e]: --> cytidine
M276	0.0060428	0.00686034	0.00477668	0.00211891		False	[e]: --> deoxycytidine
M277	0	0	0	0		False	[e]: --> D-fructose
M278	0	0	0	0		False	[e]: --> D-mannose
M279	0.127709	0.184225	0.242428	0.30944		False	[e]: --> D-ribose
M280	0.0189171	0.0214764	0.0149535	0.00709484		False	[e]: <==> fatty acid (Mpn)
M281	4.46E-06	5.07E-06	3.53E-06	1.67E-06		False	[e]: --> folic acid
M282	5.10836	7.369	9.69714	12.3776		False	[e]: --> D-glucose
M283	0.127709	0.184225	0.242428	0.30944		False	[e]: --> glycerol
M284	0.00404275	0.00458969	0.00319569	0.00151623		False	[e]: --> guanine

M285	-10.2246	-14.9068	-19.8945	-25.6422		False	[e]: <==> H+
M286	0	0	0	0		False	[e]: --> mannitol
M287	0.00019718	0.00027105	8.28E-05	3.35E-06		False	[e]: --> nicotinate
M288	0.0945652	0.146456	0.216448	0.297221		False	[e]: <==> orthophosphate
M289	7.68887	7.28799	5.09601	3.29292		False	[e]: --> oxygen
M290	4.60E-06	5.22E-06	3.63E-06	1.72E-06		False	[e]: --> pantetheine
M291	0	0	0	0		False	[e]: --> phosphatidylcholine
M292	4.46E-06	5.07E-06	3.53E-06	1.67E-06		False	[e]: --> pyridoxal
M293	4.46E-06	5.07E-06	3.53E-06	1.67E-06		False	[e]: --> riboflavin
M294	0.127709	0.184225	0.242428	0.30944		False	[e]: --> sn-glycerol 3-phosphate
M295	4.46E-06	5.07E-06	3.53E-06	1.67E-06		False	[e]: --> thiamin
M296	0	0	0	0		False	[e]: --> thymine
M297	0	0	0	0		False	[e]: --> uracil
M298	-7.53968	-7.05254	-4.47367	-2.21489		False	[e]: <==> H2O
M299	0.00106538	0.00116231	0.00091526	0.000617615	-0.8800039	TRUE	[c]: (25) L-valyl-tRNA(Val) + (18) L-aspartyl-tRNA(Asp) + (13) L-arginyl-tRNA(Arg) + N-formylmethionyl-tRNA(Met) + (826) ATP + (32) L-lysyl-tRNA(Lys) + (17) L-glutaminyl-tRNA(Gln) + (22) L-isoleucyl-tRNA(Ile) + (482) H2O + (21) L-seryl-tRNA(Ser) + (14) L-prolyl-tRNA(Pro) + (21) glycyl-tRNA(Gly) + (27) L-alanyl-tRNA(Ala) + (10) L-tyrosyl-tRNA(Tyr) + (21) L-threonyl-tRNA(Thr) + (20) L-asparaginyl-tRNA(Asn) + (5) L-methionyl-tRNA(Met) + (6) L-histidyl-tRNA(His) + (3) L-cysteinyl-tRNA(Cys) + (22) L-glutamyl-tRNA(Glu) + (3) L-tryptophanyl-tRNA(Trp) + (15) L-phenylalanyl-tRNA(Phe) + (29) L-leucyl-tRNA(Leu) --> (3) tRNA(Trp) + (14) tRNA(Pro) + (10) tRNA(Tyr) + (29) tRNA(Leu) + (6) tRNA(Met) + (13) tRNA(Arg) + (22) tRNA(Glu) + (3) tRNA(Cys) + (27) tRNA(Ala) + (6) tRNA(His) + (32) tRNA(Lys) + (18) tRNA(Asp) + (25) tRNA(Val) + (826) H+ + (22) tRNA(Ile) + (21) tRNA(Ser) + (826) orthophosphate + (17) tRNA(Gln) + (826) ADP + (15) tRNA(Phe) + (21) tRNA(Gly) + (20) tRNA(Asn) + protein (Mpn) + (21) tRNA(Thr)
M300	0	0	0	0		TRUE	[c]: H2O + protein (Mpn) + ATP --> ADP + orthophosphate + DnaK-folded protein (Mpn) + H+
M301	0	0	0	0		TRUE	[c]: (7) H2O + protein (Mpn) + (7) ATP --> (7) ADP + GroEL-folded protein (Mpn) + (7) orthophosphate + (7) H+
M302	0.0003489	0.0003489	0.0003489	0.0003489	-0.2400432	TRUE	[c]: (1032) H2O + protein (Mpn) + (688) ATP --> (27) L-alanine + (10) L-tyrosine + (21) glycine + (3) L-tryptophan + (21) L-serine + (20) L-asparagine + (22) L-glutamate + (29) L-leucine + (688) H+ + (14) L-proline + (3) L-cysteine + (13) L-arginine + (32) L-lysine + (21) L-threonine + (15) L-phenylalanine + (688) orthophosphate + (18) L-aspartate + (6) L-methionine + (688) ADP + (17) L-glutamine + (22) L-isoleucine + (25) L-valine + (6) L-histidine
M303	1.34E-07	1.52E-07	1.06E-07	5.02E-08	-2.67E-05	TRUE	[c]: (5) L-valyl-tRNA(Val) + (6) L-aspartyl-tRNA(Asp) + (2) L-arginyl-tRNA(Arg) + N-formylmethionyl-tRNA(Met) + (199) ATP + (11) L-lysyl-tRNA(Lys) + (4) L-glutaminyl-tRNA(Gln) + (8) L-isoleucyl-tRNA(Ile) + (199) H2O + (5) L-seryl-tRNA(Ser) + L-prolyl-tRNA(Pro) + glycyl-tRNA(Gly) + (4) L-alanyl-tRNA(Ala) + L-threonyl-tRNA(Thr) + (3) L-asparaginyl-tRNA(Asn) + (3) L-methionyl-tRNA(Met) + L-histidyl-tRNA(His) + (11) L-glutamyl-tRNA(Glu) + (5) L-phenylalanyl-tRNA(Phe) + (12) L-leucyl-tRNA(Leu) --> tRNA(Pro) + (12) tRNA(Leu) + (4) tRNA(Met) + (2) tRNA(Arg) + (11) tRNA(Glu) + (4) tRNA(Ala) + tRNA(His) + (11) tRNA(Lys) + (6) tRNA(Asp) + (5) tRNA(Val) + (282) H+ + (8) tRNA(Ile) + apoprotein [acyl carrier protein] + (5) tRNA(Ser) + (199) orthophosphate + (4) tRNA(Gln) + (199) ADP + (5) tRNA(Phe) + tRNA(Gly) + (3) tRNA(Asn) + tRNA(Thr)
M304	13.105	16.9618	20.435	25.2015	-13.105	TRUE	[c]: H2O + ATP --> ADP + orthophosphate + H+
M305	4.46E-08	5.07E-08	3.53E-08	1.67E-08		False	[c]: (1657) guanosine + (1366) L-tyrosine + (9220) glycine + (1864) L-tryptophan + (54) L-asparagine + (100) S-adenosyl-L-methionine + (100) pyridoxal phosphate + (67) L-cysteine + a100) thiamin diphosphate + (2489) L-threonine + (100) 5-formyltetrahydrofolate + (1242) thymidine + (2793) L-valine + (1624) DNA (Mpn) + (1981) adenosine + (100) CoA + (9824) L-alanine + (3202) L-serine + (2015) RNA (Mpn) + (63702) glycolipid (Mpn) + (503) cytidine + (190279) D-glucose 6-phosphate + (3) acyl carrier protein + (18651) L-glutamate + (21313) L-leucine + (6837) L-proline + (100) NADP+ + (3913) L-arginine + (1741) L-lysine + (5122) L-phenylalanine + (1370) L-methionine + (9318) L-aspartate + (100) FAD + (294) L-glutamine + (1858) L-isoleucine + (148168) phosphatidic acid (Mpn) + (54847) protein (Mpn) + (2422) L-histidine + (100) NADPH + (2541) uridine --> (1000000) biomass
M306	0.0446431	0.0506829	0.0352892	0.0167434	-1.1160775	TRUE	biomass[c] + (25) H2O[c] + (25) ATP[c] --> biomass[e] + (25) ADP[c] + (25) orthophosphate[c] + (25) H+[c]

Table S9: Predicted Fluxes for the Exponential Growth Phase: The fluxes for all model reactions in mmol per gram of cells and hour as predicted for different time points of the exponential growth phase of batch culture growth are shown. Negative fluxes indicate that the flux direction of the respective reaction is opposed to the reaction annotation in table S1.

Energy balances

Total consumption	-23.31	mmol/gDW/h
Total production	23.31	mmol/gDW/h
NGAM (%)	56.22	
GAM (%)	4.79	

Vitamin and cofactor metabolism 0.003 %
