# Supplementary material for: Metabolic modeling of energy balances in Mycoplasma hyopneumoniae shows that pyruvate addition increases growth rate
Source: Biotechnol Bioeng. 2017 Jul 27;114(10):2339–47. doi: 10.1002/bit.26347 (PMC6084303; doi:10.1002/bit.26347)
Supplement: Supplementary file 11 — Table S10. Expression level of model genes determined using RNA sequencing in the glucose batch and pyruvate batch. Highlighted genes were mentioned in the manuscript. [file BIT-114-2339-s011.docx]

| Table S10 Expression level of model genes determined using RNA sequencing in the glucose batch and pyruvate batch. Highlighted genes were mentioned in the manuscript | | | | | |  |  |
| --- | --- | --- | --- | --- | --- | --- | --- |
|  |  |  |  |  |  |  |  |
| **Model gene ID** | **Gene ID** | **Annotated function strain 232** | **Gene_ID_232** | **RPKM_glucose batch** | **RPKM_pyruvate batch** | **Flux Glucose batch  (mmmol/gDW/h)** | **Flux Pyruvate batch (mmol/gDW/h)** |
| G18KM-1261 | _568 | Hypothetical protein | aceF | 1400.393068 | 1581.563644 | -1.08E+001 | -6.69E+000 |
| G18KM-1263 | _570 | aliphatic acid kinase short-chain | ackA | 1574.946403 | 1721.698498 | -1.08E+001 | -6.69E+000 |
| G18KM-1209 | _516 | Hypothetical protein | acpD\|(NC_006360\|563951..564535) | 676.7253338 | 740.8002167 | 0.00E+000 | 0.00E+000 |
| G18KM-1210 | _517 | nadh-azoreductase fmn-dependent | acpD\|(NC_006360\|564477..565154) | 486.7222763 | 542.3963717 | 0.00E+000 | 0.00E+000 |
| G18KM-1066 | _373 | adenylate kinase/ump-cmp kinase | adk | 545.3831173 | 574.4650893 | 8.04E-005 | 1.15E-004 |
| G18KM-1066 | _373 | adenylate kinase/ump-cmp kinase | adk | 545.3831173 | 574.4650893 | 4.43E-001 | 4.88E-001 |
| G18KM-1124 | _431 | adenine phosphoribosyl transferase | apt | 3209.52026 | 6906.97161 | -1.91E-003 | -2.74E-003 |
| G18KM-1196 | _503 | Hypothetical protein | araD | 1194.795442 | 1010.704494 | 0.00E+000 | 0.00E+000 |
| G18KM-862 | _169 | aminoacyl-transfer rna synthetases class-i signature. | argS | 365.6601684 | 421.2270784 | 8.28E-003 | 9.89E-003 |
| G18KM-1174 | _481 | aspartyl/asparaginyl-trna synthetase class iib | asnC | 984.1088538 | 1240.818117 | 0.00E+000 | 0.00E+000 |
| G18KM-1000 | _307 | aspartyl/asparaginyl-trna synthetase class iib | aspS | 164.5173725 | 208.4088476 | 1.15E-002 | 1.37E-002 |
| G18KM-1084 | _391 | cytidine deaminase homotetrameric | cdd | 160.8233667 | 184.3384332 | 1.48E-003 | 2.12E-003 |
| G18KM-1084 | _391 | cytidine deaminase homotetrameric | cdd | 160.8233667 | 184.3384332 | 0.00E+000 | 0.00E+000 |
| G18KM-890 | _197 | phospholipase d/transphosphatidylase | cls | 86.29550359 | 155.9996621 | 1.31E-003 | 1.87E-003 |
| G18KM-923 | _230 | cytidylate kinase | cmk | 255.8092454 | 264.6663289 | 1.42E-001 | 1.43E-001 |
| G18KM-923 | _230 | cytidylate kinase | cmk | 255.8092454 | 264.6663289 | 4.03E-004 | 5.76E-004 |
| G18KM-815 | _122 | cysteinyl-trna synthetase/mycothiol ligase | cysS | 41.78783405 | 80.82461861 | 1.91E-003 | 2.28E-003 |
| G18KM-1157 | _464 | thymidine and pyrimidine-nucleoside phosphorylases signature. | deoA | 1146.735833 | 987.8622659 | 1.48E-003 | 2.12E-003 |
| G18KM-1157 | _464 | thymidine and pyrimidine-nucleoside phosphorylases signature. | deoA | 1146.735833 | 987.8622659 | 0.00E+000 | 0.00E+000 |
| G18KM-1157 | _464 | thymidine and pyrimidine-nucleoside phosphorylases signature. | deoA | 1146.735833 | 987.8622659 | -9.96E-004 | -1.42E-003 |
| G18KM-1080 | _387 | phosphopentomutase | DeoB | 154.5668246 | 209.5080936 | 0.00E+000 | 0.00E+000 |
| G18KM-1080 | _387 | phosphopentomutase | DeoB | 154.5668246 | 209.5080936 | 0.00E+000 | 0.00E+000 |
| G18KM-708 | _15 | deoxyribose-phosphate aldolase | deoC | 278.9180686 | 387.914022 | 0.00E+000 | 0.00E+000 |
| G18KM-1156 | _463 | Hypothetical protein | deoD | 1338.962122 | 1107.35855 | 0.00E+000 | 0.00E+000 |
| G18KM-1156 | _463 | Hypothetical protein | deoD | 1338.962122 | 1107.35855 | -4.03E-004 | -5.76E-004 |
| G18KM-1156 | _463 | Hypothetical protein | deoD | 1338.962122 | 1107.35855 | 0.00E+000 | 0.00E+000 |
| G18KM-1156 | _463 | Hypothetical protein | deoD | 1338.962122 | 1107.35855 | -8.04E-005 | -1.15E-004 |
| G18KM-992 | _299 | enolase | eno | 1738.895798 | 1326.080465 | 1.08E+001 | 5.02E+000 |
| G18KM-1264 | _571 | phosphate acetyl/butaryl transferase | eutD | 1246.922408 | 1478.598414 | 1.08E+001 | 6.69E+000 |
| G18KM-864 | _171 | ketose-bisphosphate aldolase class-ii | fba | 881.1605121 | 1001.660703 | 0.00E+000 | 0.00E+000 |
| G18KM-886 | _193 | glyceraldehyde 3-phosphate dehydrogenase active site | gap | 3182.105276 | 2248.947625 | 1.08E+001 | 5.02E+000 |
| G18KM-879 | _186 | amidase | gatA | 280.0059481 | 343.8373242 | 0.00E+000 | 0.00E+000 |
| G18KM-762 | _69 | fad dependent oxidoreductase | glpD | 219.2379828 | 303.1675464 | 6.34E-001 | 4.31E-001 |
| G18KM-1352 | _659 | major intrinsic protein | glpF | 571.4403507 | 42.61073162 | 5.10E-001 | 3.10E-001 |
| G18KM-1353 | _660 | glycerol kinase | glpK | 658.8205562 | 49.40050339 | 5.10E-001 | 3.10E-001 |
| G18KM-1296 | _603 | Hypothetical protein | glpQ | 122.2404122 | 82.14201827 | 0.00E+000 | 0.00E+000 |
| G18KM-1100 | _407 | glutamyl/glutaminyl-trna synthetase | gltX | 111.2511371 | 164.3750898 | 1.08E-002 | 1.29E-002 |
| G18KM-1100 | _407 | glutamyl/glutaminyl-trna synthetase | gltX | 111.2511371 | 164.3750898 | 1.40E-002 | 1.68E-002 |
| G18KM-1017 | _324 | serine hydroxymethyltransferase pyridoxal-phosphate attachment site. | glyA | 766.2076913 | 1379.969835 | 0.00E+000 | 0.00E+000 |
| G18KM-911 | _218 | glycyl-trna synthetase | glyS | 194.8415851 | 264.189497 | 1.34E-002 | 1.60E-002 |
| G18KM-1088 | _395 | guanylate kinase | gmk | 101.0736534 | 128.844285 | 4.03E-004 | 5.76E-004 |
| G18KM-1088 | _395 | guanylate kinase | gmk | 101.0736534 | 128.844285 | 2.18E-001 | 2.18E-001 |
| G18KM-1001 | _308 | histidyl-trna synthetase | hisS | 102.837863 | 159.9455013 | 3.82E-003 | 4.57E-003 |
| G18KM-980 | _287 | Hypothetical protein | hpt | 1236.860057 | 764.3032585 | -9.64E-004 | -1.38E-003 |
| G18KM-1104 | _411 | l-lactate dehydrogenase active site | IctD | 16763.88193 | 4127.772512 | 0.00E+000 | -6.50E+000 |
| G18KM-883 | _190 | isoleucine-trna ligase | ileS | 335.1345175 | 235.9460265 | 1.40E-002 | 1.68E-002 |
| G18KM-1015 | _322 | carbohydrate kinase pfkb | iolC | 901.4892711 | 138.0709558 | 0.00E+000 | 0.00E+000 |
| G18KM-1015 | _322 | carbohydrate kinase pfkb | iolC | 901.4892711 | 138.0709558 | 0.00E+000 | 0.00E+000 |
| G18KM-1012 | _319 | 3 5/4-trihydroxycyclohexa-1 2-dione hydrolase | iolD | 1148.473763 | 190.6659619 | 0.00E+000 | 0.00E+000 |
| G18KM-821 | _128 | leucine-trna ligase | leuS | 261.9910291 | 426.275427 | 1.85E-002 | 2.21E-002 |
| G18KM-892 | _199 | choline/ethanolamine kinase | licA | 238.7876231 | 413.3302266 | 0.00E+000 | 0.00E+000 |
| G18KM-892 | _199 | choline/ethanolamine kinase | licA | 238.7876231 | 413.3302266 | 0.00E+000 | 0.00E+000 |
| G18KM-988 | _295 | bacterial lipoate protein ligase c-terminus | lplA\|(NC_006360\|145867..146877) | 202.1393512 | 213.3717647 | 0.00E+000 | 0.00E+000 |
| G18KM-1034 | _341 | lysyl-trna synthetase signature | lysS | 281.6550445 | 321.7299403 | 2.04E-002 | 2.44E-002 |
| G18KM-1169 | _476 | methionyl-trna synthetase | metG | 128.3791884 | 147.6328157 | 3.19E-003 | 3.81E-003 |
| G18KM-1206 | _513 | s-adenosylmethionine synthase signature 2. | metK | 240.7169047 | 239.5258143 | 0.00E+000 | 0.00E+000 |
| G18KM-856 | _163 | Hypothetical protein | mhp006 | 167.91415 | 148.7352484 | 1.72E-002 | 2.05E-002 |
| G18KM-914 | _221 | gtp cyclohydrolase 1 type 2/nif3 | mhp064 | 107.2524203 | 175.4123497 | 0.00E+000 | 0.00E+000 |
| G18KM-1011 | _318 | myo inos iole: myo-inosose-2 dehydratase | mhp148 | 1286.94888 | 255.9095099 | 0.00E+000 | 0.00E+000 |
| G18KM-1013 | _320 | inositol 2-dehydrogenase | mhp150 | 1042.954253 | 163.9090817 | 0.00E+000 | 0.00E+000 |
| G18KM-1014 | _321 | kdui/iolb isomerase | mhp151 | 864.7506693 | 142.0741633 | 0.00E+000 | 0.00E+000 |
| G18KM-1014 | _321 | kdui/iolb isomerase | mhp151 | 864.7506693 | 142.0741633 | 0.00E+000 | 0.00E+000 |
| G18KM-1096 | _403 | chromate transporter | mhp237 | 28.40531461 | 41.55478872 | 0.00E+000 | 0.00E+000 |
| G18KM-1142 | _449 | dutpase/dctp pyrophosphatase | mhp284 | 206.081262 | 395.2909412 | 0.00E+000 | 0.00E+000 |
| G18KM-1142 | _449 | dutpase/dctp pyrophosphatase | mhp284 | 206.081262 | 395.2909412 | 0.00E+000 | 0.00E+000 |
| G18KM-1344 | _651 | Hypothetical protein | mhp362 | 23.07098398 | 37.01550014 | 0.00E+000 | 0.00E+000 |
| G18KM-1370 | _677 | phosphotriesterase | mhp389 | 4834.170738 | 7971.573904 | 0.00E+000 | 0.00E+000 |
| G18KM-1167 | _474 | aldolase-type tim barrel | mhp408 | 770.1287925 | 681.0241078 | 0.00E+000 | 0.00E+000 |
| G18KM-1248 | _555 | phosphotransferase system fructose-specific iib subunit | mhp490 | 1456.660852 | 919.1820973 | 0.00E+000 | 0.00E+000 |
| G18KM-1248 | _555 | phosphotransferase system fructose-specific iib subunit | mhp490 | 1456.660852 | 919.1820973 | 0.00E+000 | 0.00E+000 |
| G18KM-745 | _52 | ketose-bisphosphate aldolase class-ii | mhp589 | 144.7913548 | 80.16983297 | 0.00E+000 | 0.00E+000 |
| G18KM-745 | _52 | ketose-bisphosphate aldolase class-ii | mhp589 | 144.7913548 | 80.16983297 | 5.11E+000 | 2.30E+000 |
| G18KM-750 | _57 | ribose/galactose isomerase | mhp594 | 709.8582117 | 1000.586587 | 0.00E+000 | 0.00E+000 |
| G18KM-750 | _57 | ribose/galactose isomerase | mhp594 | 709.8582117 | 1000.586587 | -2.90E-003 | -4.15E-003 |
| G18KM-786 | _93 | phosphotransferase system sugar-specific permease eiia type 1 | mhp629 | 506.1752609 | 155.3366957 | 5.11E+000 | 2.30E+000 |
| G18KM-807 | _114 | phospholipid/glycerol acyltransferase | mhp652 | 92.45522319 | 89.06753015 | 2.77E-003 | 3.96E-003 |
| G18KM-1016 | _323 | aldehyde dehydrogenase cysteine active site | mmsA | 1535.204454 | 212.5850778 | 0.00E+000 | 0.00E+000 |
| G18KM-731 | _38 | phosphotransferase system eiib component type 2/3 | mtlA | 12.10372856 | 13.91634236 | 0.00E+000 | 0.00E+000 |
| G18KM-730 | _37 | Hypothetical protein | mtlD | 4.183232309 | 10.82540139 | 0.00E+000 | 0.00E+000 |
| G18KM-1232 | _539 | cytidylyltransferase | nadD | 202.7101683 | 223.162332 | 0.00E+000 | 0.00E+000 |
| G18KM-1232 | _539 | cytidylyltransferase | nadD | 202.7101683 | 223.162332 | 0.00E+000 | 0.00E+000 |
| G18KM-1232 | _539 | cytidylyltransferase | nadD | 202.7101683 | 223.162332 | 0.00E+000 | 0.00E+000 |
| G18KM-1229 | _536 | nad/gmp synthase | nadE | 1149.028311 | 534.1881443 | 0.00E+000 | 0.00E+000 |
| G18KM-744 | _51 | amidohydrolase | nagA | 105.0018388 | 114.5725354 | 0.00E+000 | 0.00E+000 |
| G18KM-747 | _54 | glucosamine/galactosamine-6-phosphate isomerase | nagB | 86.46694127 | 19.70538173 | 0.00E+000 | 0.00E+000 |
| G18KM-1158 | _465 | fad-dependent pyridine nucleotide reductase signature | nox | 2105.796003 | 2931.715301 | 1.08E+001 | 2.60E+000 |
| G18KM-1020 | _327 | ribonucleotide reductase large chain signature | nrdE | 840.471917 | 589.9395369 | 0.00E+000 | 0.00E+000 |
| G18KM-1020 | _327 | ribonucleotide reductase large chain signature | nrdE | 840.471917 | 589.9395369 | 0.00E+000 | 0.00E+000 |
| G18KM-1020 | _327 | ribonucleotide reductase large chain signature | nrdE | 840.471917 | 589.9395369 | 0.00E+000 | 0.00E+000 |
| G18KM-1020 | _327 | ribonucleotide reductase large chain signature | nrdE | 840.471917 | 589.9395369 | 9.16E-004 | 1.31E-003 |
| G18KM-1123 | _430 | dehydrogenase e1 component | pdhA | 2932.064645 | 2096.574361 | 1.08E+001 | 6.69E+000 |
| G18KM-1262 | _569 | pyridine nucleotide disulphide reductase class-i signature | pdhD\|(NC_006360\|635265..637112) | 1741.016224 | 2253.833414 | 0.00E+000 | 0.00E+000 |
| G18KM-1262 | _569 | pyridine nucleotide disulphide reductase class-i signature | pdhD\|(NC_006360\|635265..637112) | 1741.016224 | 2253.833414 | 1.08E+001 | 6.69E+000 |
| G18KM-1127 | _434 | atp-dependent 6-phosphofructokinase | pfkA | 234.5517004 | 366.0791353 | 5.11E+000 | 2.30E+000 |
| G18KM-712 | _19 | phosphoglucose isomerase signature 1. | pgi | 209.8997576 | 274.455383 | 5.11E+000 | 2.30E+000 |
| G18KM-1246 | _553 | phosphoglycerate kinase signature. | pgk | 706.1393275 | 624.4337763 | -2.18E-001 | -2.18E-001 |
| G18KM-1246 | _553 | phosphoglycerate kinase signature. | pgk | 706.1393275 | 624.4337763 | -9.96E-004 | -1.42E-003 |
| G18KM-1246 | _553 | phosphoglycerate kinase signature. | pgk | 706.1393275 | 624.4337763 | 5.40E+000 | 2.38E+000 |
| G18KM-1246 | _553 | phosphoglycerate kinase signature. | pgk | 706.1393275 | 624.4337763 | -1.60E+001 | -7.18E+000 |
| G18KM-770 | _77 | bpg-independent pgam n-terminus (ipgm n) | pgm | 546.7167933 | 407.2424831 | 1.08E+001 | 5.02E+000 |
| G18KM-969 | _276 | aminoacyl-trna synthetase class ii | pheS | 414.3383198 | 402.317846 | 9.56E-003 | 1.14E-002 |
| G18KM-1250 | _557 | mannose-6-phosphate isomerase type i | pmi | 3021.880428 | 1570.125855 | 0.00E+000 | 0.00E+000 |
| G18KM-779 | _86 | inorganic pyrophosphatase | ppa | 2206.219827 | 2151.69255 | 9.99E-001 | 1.05E+000 |
| G18KM-1378 | _685 | proline-trna ligase class iia | proS | 334.3574094 | 495.8747672 | 8.92E-003 | 1.07E-002 |
| G18KM-828 | _135 | ribose-phosphate diphosphokinase | prsA | 316.490689 | 392.6606431 | 4.36E-003 | 6.23E-003 |
| G18KM-961 | _268 | cdp-alcohol phosphatidyltransferase | psgA | 55.2553845 | 88.4594433 | 1.31E-003 | 1.87E-003 |
| G18KM-1037 | _344 | peptidyl-trna hydrolase signature 1. | pth | 443.764712 | 504.8907313 | -1.27E-002 | -1.52E-002 |
| G18KM-785 | _92 | phosphotransferase system hpr serine phosphorylation site | ptsH | 790.5192952 | 674.6423328 | 0.00E+000 | 0.00E+000 |
| G18KM-1114 | _421 | pyruvate kinase | pyk | 579.9923841 | 628.5524292 | 4.03E-004 | 5.76E-004 |
| G18KM-1114 | _421 | pyruvate kinase | pyk | 579.9923841 | 628.5524292 | 1.42E-001 | 1.43E-001 |
| G18KM-1114 | _421 | pyruvate kinase | pyk | 579.9923841 | 628.5524292 | 9.96E-004 | 1.42E-003 |
| G18KM-1114 | _421 | pyruvate kinase | pyk | 579.9923841 | 628.5524292 | 1.95E-001 | 1.96E-001 |
| G18KM-1114 | _421 | pyruvate kinase | pyk | 579.9923841 | 628.5524292 | 0.00E+000 | 0.00E+000 |
| G18KM-1114 | _421 | pyruvate kinase | pyk | 579.9923841 | 628.5524292 | 5.40E+000 | 2.38E+000 |
| G18KM-1114 | _421 | pyruvate kinase | pyk | 579.9923841 | 628.5524292 | 0.00E+000 | 0.00E+000 |
| G18KM-1114 | _421 | pyruvate kinase | pyk | 579.9923841 | 628.5524292 | 0.00E+000 | 0.00E+000 |
| G18KM-962 | _269 | glutamine amidotransferase | pyrG | 321.019284 | 431.2950671 | 6.19E-004 | 8.86E-004 |
| G18KM-1152 | _459 | gtpase mtg1 | rbgA | 144.0845335 | 151.5261133 | 0.00E+000 | 0.00E+000 |
| G18KM-1010 | _317 | abc transporter | rbsA | 894.0240916 | 150.6381697 | 0.00E+000 | 0.00E+000 |
| G18KM-964 | _271 | fad synthetase | ribF | 162.5540888 | 160.2657126 | 0.00E+000 | 0.00E+000 |
| G18KM-964 | _271 | fad synthetase | ribF | 162.5540888 | 160.2657126 | 0.00E+000 | 0.00E+000 |
| G18KM-739 | _46 | aldolase-type tim barrel | rpe\|(NC_006360\|725462..726118) | 121.1076921 | 97.80351703 | -2.90E-003 | -4.15E-003 |
| G18KM-991 | _298 | serine-trna ligase type1 | serS | 938.757545 | 804.9610427 | 1.34E-002 | 1.60E-002 |
| G18KM-1197 | _504 | l-ribulose-5-phosphate 3-epimerase | sgaU | 684.967509 | 579.1955429 | 0.00E+000 | 0.00E+000 |
| G18KM-784 | _91 | thymidine kinase cellular-type signature. | tdk | 408.3407478 | 449.9229463 | 0.00E+000 | 0.00E+000 |
| G18KM-784 | _91 | thymidine kinase cellular-type signature. | tdk | 408.3407478 | 449.9229463 | 9.96E-004 | 1.42E-003 |
| G18KM-764 | _71 | threonine-trna ligase class iia | thrS | 194.205079 | 261.8019571 | 1.34E-002 | 1.60E-002 |
| G18KM-1188 | _495 | transketolase signature 2. | tktA | 925.4172297 | 942.6389151 | 1.45E-003 | 2.08E-003 |
| G18KM-1188 | _495 | transketolase signature 2. | tktA | 925.4172297 | 942.6389151 | -1.45E-003 | -2.08E-003 |
| G18KM-983 | _290 | thymidylate kinase signature. | tmk | 401.3606902 | 330.8933344 | 9.96E-004 | 1.42E-003 |
| G18KM-1135 | _442 | triosephosphate isomerase active site | tpiA | 428.324306 | 593.0411552 | -5.74E+000 | -2.73E+000 |
| G18KM-765 | _72 | tryptophan-trna ligase | trpS | 371.115619 | 460.0107952 | 1.91E-003 | 2.28E-003 |
| G18KM-1139 | _446 | pyridine nucleotide-disulphide oxidoreductase class-ii | trxB | 665.9285515 | 724.7280261 | -9.16E-004 | -1.31E-003 |
| G18KM-916 | _223 | tyrosine-trna ligase | tyrS | 80.27104103 | 155.3324497 | 6.37E-003 | 7.61E-003 |
| G18KM-1364 | _671 | Hypothetical protein | ugpE | 285.4414422 | 171.780624 | 1.28E-001 | 1.28E-001 |
| G18KM-1369 | _676 | phosphotransferase system sugar-specific permease component | ulaA\|(NC_006360\|464573..466342) | 4099.392518 | 6440.745466 | 0.00E+000 | 0.00E+000 |
| G18KM-1198 | _505 | aldolase-type tim barrel | ulaD | 762.4976885 | 844.1792017 | 0.00E+000 | 0.00E+000 |
| G18KM-1198 | _505 | aldolase-type tim barrel | ulaD | 762.4976885 | 844.1792017 | 0.00E+000 | 0.00E+000 |
| G18KM-707 | _14 | uracil phosphoribosyltransferase | upp | 1159.39203 | 853.602283 | -1.48E-003 | -2.12E-003 |
| G18KM-806 | _113 | 5'-nucleotidase signature 2. | ushA | 1747.019786 | 699.058157 | 0.00E+000 | 0.00E+000 |
| G18KM-806 | _113 | 5'-nucleotidase signature 2. | ushA | 1747.019786 | 699.058157 | 0.00E+000 | 0.00E+000 |
| G18KM-806 | _113 | 5'-nucleotidase signature 2. | ushA | 1747.019786 | 699.058157 | 0.00E+000 | 0.00E+000 |
| G18KM-806 | _113 | 5'-nucleotidase signature 2. | ushA | 1747.019786 | 699.058157 | 0.00E+000 | 0.00E+000 |
| G18KM-806 | _113 | 5'-nucleotidase signature 2. | ushA | 1747.019786 | 699.058157 | 0.00E+000 | 0.00E+000 |
| G18KM-806 | _113 | 5'-nucleotidase signature 2. | ushA | 1747.019786 | 699.058157 | 0.00E+000 | 0.00E+000 |
| G18KM-806 | _113 | 5'-nucleotidase signature 2. | ushA | 1747.019786 | 699.058157 | 0.00E+000 | 0.00E+000 |
| G18KM-806 | _113 | 5'-nucleotidase signature 2. | ushA | 1747.019786 | 699.058157 | 0.00E+000 | 0.00E+000 |
| G18KM-845 | _152 | aminoacyl-transfer rna synthetases class-i signature. | valS | 126.1978368 | 139.2154549 | 1.59E-002 | 1.90E-002 |
