# Supplementary material for: Metabolic modeling of energy balances in Mycoplasma hyopneumoniae shows that pyruvate addition increases growth rate
Source: Biotechnol Bioeng. 2017 Jul 27;114(10):2339–47. doi: 10.1002/bit.26347 (PMC6084303; doi:10.1002/bit.26347)
Supplement: Supplementary file 12 — Table S11. Abbreviations used for metabolite names in the metabolic map. [file BIT-114-2339-s012.docx]

| Tabel S11: Abbreviations used for metabolite names in the metabolic map | |
| --- | --- |
|  |  |
| **Abbreviation** | **Metabolite name** |
| 1aG3P | 1-acyl-sn-glycerol 3-phosphate |
| 3dG6P | 3-dehydro-L-gulonate 6-phosphate |
| A35ADP | adenosine 3',5'-bisphosphate |
| A6P | L-ascorbate 6-phosphate |
| aa | amino acid |
| AcAld | acetaldehyde |
| AcCoA | acetyl-CoA |
| ACE | acetate |
| ACP | acyl-carrier-protein, holo[acyl-carrier-protein] |
| AcP | acetyl phosphate |
| Ado | adenosine |
| ADP | adenosine 5'-diphosphate |
| AMP | adenosine 5'-phosphate |
| apoACP | apo-[acyl-carrier protein] |
| ATP | adenosine 5'-triphosphate |
| Cat1 | Monovalent cation |
| Cat2 | Divalent cation |
| CDP | cytidine 5'-diphosphate |
| CDP-CHO | CDP-Choline |
| CDP-DAG | CDP-diacylglycerol |
| CHO | choline |
| CHR | chromate |
| CL | cardiolipin |
| CMP | cytidine 5'-phosphate |
| CoA | coenzyme A |
| CPD365 | scyllo-inosine |
| CTP | cytidine 5'-triphosphate |
| dAdo | deoxyadenosine |
| dADP | 2'-deoxyadenosine 5'-diphosphate |
| DAG | diacylglycerol, 1,2-diacyl-sn-glycerol |
| dAMP | 2'-deoxyadenosine 5'-phosphate |
| dATP | 2'-deoxyadenosine 5'-triphosphate |
| dCDP | 2'-deoxycytidine 5'-diphosphate |
| dCMP | 2'-deoxycytidine 5'-phosphate |
| dCTP | 2'-deoxycytidine 5'-triphosphate |
| dCyt | deoxycytidine |
| dGDP | 2'-deoxyguanosine 5'-diphosphate |
| dGMP | 2'-deoxyguanosine 5'-phosphate |
| DGP | 3-phospho-D-glyceroyl phosphate |
| dGTP | 2'-deoxyguanosine 5'-triphosphate |
| dGuo | deoxyguanosine |
| DHAP | glycerone phosphate, dihydroxyacetone phosphate |
| dNAD | deamido-NAD+ |
| dPCoA | dephospho-CoA |
| dR1P | 2-deoxy-D-ribose 1-phosphate |
| dR5P | 2-deoxy-D-ribose 5-phosphate |
| dRu5P | D-ribulose 5-phosphate |
| dUDP | 2'-deoxyuridine 5'-diphosphate |
| dUMP | 2'-deoxyuridine 5'-phosphate |
| dUrd | deoxyuridine |
| E4P | D-erythrose 4-phosphate |
| F1P | D-fructose 1-phosphate |
| F6P | D-fructose 6-phosphate |
| FA | fatty acid |
| FAD | flavin-adenine dinucleotide |
| FBP | D-fructose 1,6-bisphosphate |
| FMN | flavin mononucleotide |
| G3P | glycerol 3-phosphate |
| G3PC | sn-glycero-3-phosphocholine |
| G6P | D-glucose 6-phosphate |
| GA | glyceraldehyde |
| GAP | D-glyceraldehyde 3-phosphate |
| GDP | guanosine 5'-diphosphate |
| GLY | glycerol |
| Gly | glycine |
| GMP | guanosine 5'-phosphate |
| GTP | guanosine 5'-triphosphate |
| Guo | guanosine |
| H+ | proton |
| H2O | water |
| H2O2 | hydrogen peroxide |
| lc-AcCoA | long-chain acyl CoA |
| lc-FA | long-chain fatty acid |
| lRu5P | L-ribulose 5-phosphate |
| lX5P | L-xylulose 5-phosphate |
| M6P | D-mannose 6-phosphate |
| MAL | methylmalonate semialdehyde |
| Met | L-methionine |
| METHF | 5,10-methenyltetrahydrofolate |
| Mtl1P | D-mannitol 1-phosphate |
| NAC | nicotinate |
| NACD | nicotinate D-ribonucleotide |
| NAD+ | nicotinamide adenine dinucleotide |
| NADH | nicotinamide adenine dinucleotide -reduced |
| NADP+ | nicotinamide adenine dinucleotide phosphate |
| NADPH | nicotinamide adenine dinucleotide phosphate -reduced |
| NUC | Nucleotide |
| OPP | oligopeptide |
| oxThio | thioredoxin disulfide |
| PAC | phosphatidate, 1,2-diacyl-sn-glycerol 3-phosphate |
| PAN4P | pantetheine 4'-phosphate |
| PEP | phosphoenolpyruvate |
| pG3P | phosphatidylglycerophosphate, 3(3-sn-phosphatidyl)-sn-glycerol 1-phosphate |
| pGLY | phosphatidylglycerol Pi phosphate |
| Pi | phosphate |
| PPi | diphosphate |
| PRPP | 5-phospho-D-ribose 1-diphosphate |
| PtdCho | phosphatidylcholine |
| PYR | pyruvate |
| R1P | D-ribose 1-phosphate |
| R5P | D-ribose 5-phosphate |
| RBF | riboflavin |
| redThio | thioredoxin |
| RIB | D-ribose |
| S7P | sedoheptulose 7-phosphate |
| SAM | S-adenosyl-L-methionine |
| SER | L-Serine |
| TDP | thymidine 5'-diphosphate |
| Thd | thymidine |
| THF | tetrahydrofolate |
| TMP | thymidine 5'-phosphate |
| TTP | thymidine 5'-triphosphate |
| UDP | uridine 5'-diphosphate |
| UMP | uridine 5'-phosphate |
| Urd | uridine |
| UTP | uridine 5'-triphosphate |
